# 3‐Deoxy‐3‐Fluoro Mannuronic Acid Alginates: Stereoselective Automated Synthesis and Conformational Behaviour

**DOI:** 10.1002/anie.5914227

**Published:** 2026-05-28

**Authors:** Sean T. Evans, Nishu Yadav, Wouter A. Remmerswaal, Daan Hoogers, Koen N. A. van de Vrande, Sarah Hosking, Ana Poveda, Jeroen D. C. Codée, Jesús Jiménez‐Barbero, Martina Delbianco, Gavin J. Miller

**Affiliations:** ^1^ School of Chemical and Physical Sciences Keele University Keele UK; ^2^ Department of Biomolecular Systems Max Planck Institute of Colloids and Interfaces Potsdam Germany; ^3^ Leiden Institute of Chemistry Leiden University Leiden the Netherlands; ^4^ Unilever Research and Development Wirral UK; ^5^ CICbioGUNE Basque Research and Technology Alliance Derio Spain; ^6^ Ikerbasque Basque Foundation for Science Bilbao Spain; ^7^ Department of Inorganic & Organic Chemistry Faculty of Science and Technology University of the Basque Country Leioa Spain; ^8^ Centro De Investigación Biomedica En Red De Enfermedades Respiratorias Fuencarral‐El Pardo Spain; ^9^ Manchester Institute of Biotechnology & Department of Chemistry University of Manchester Manchester UK

**Keywords:** alginates, β‐mannuronate, conformation, deoxyfluorination, glycosylation

## Abstract

Fluorination is a powerful strategy for the editing of glycans, creating bespoke tools to probe carbohydrate‐protein recognition and processing. Positioning the site for fluorination within a glycan is critical, whether examining bonding interactions or installing benign reporter capability. Herein, we study the effect of C‐3 fluorination of mannuronic acids in the assembly of β‐1,4‐d‐mannuronic acid alginate fragments and the effect of these point mutations on the conformational preference of the generated oligosaccharides. We explore the synthesis and glycosylating properties of a 3‐deoxy‐3‐fluoro d‐mannuronate donor to establish that the C‐3 fluoride does not thwart the high β‐stereoselectivity of the mannuronate system. Subsequent deployment of these building blocks in automated glycan assembly enables the preparation of a small library of β‐d‐mannuronate oligosaccharides containing the C‐3‐F modification. Comprehensive NMR analysis shows that even though the 3‐deoxy‐3‐fluoro units disrupt the native inter‐residue C3‐OH•••O5 hydrogen bonds, the overall conformation remains in line with that of the native β‐1,4 mannuronic acid alginate. This indicates tolerance for this modification in these structural tools and provides an exciting foundation to study self‐assembly and enzymatic processing of β‐1,4 mannuronic acid oligosaccharides.

## Graphical abstract

The impact of C‐3 fluorination on the synthesis and conformation of alginate oligosaccharides is investigated. A C‐3‐fluoro mannuronic acid building block is generated and shown to react with high β‐selectivity. Automated glycan assembly enables access to uniquely fluorinated mannuronic acid alginates where deoxyfluorination is shown not to perturb the native conformational preference of the system.

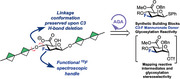

## 1 Introduction

Fluorinated glycans offer an instrumental capability for the analysis of carbohydrate‐protein interactions, principally through isosteric replacement of a sugar ring hydroxyl group that can probe native hydrogen bonding at the binding interface [1, 2, 3, 4, 5]. This can identify critical atomic‐level requirements and energetics between sugars and protein side chains without the need for extensive protein mutant libraries. Furthermore, deoxyfluorination may offer improved hydrolytic glycan profiles and enable analysis of such systems using sensitive ^19^F NMR spectroscopy and other analytical methods, such as FIB‐SIMS [6, 7, 8, 9, 10].

The exact position of sugar ring fluorination within a given glycan is critical. Its incorporation may impact physicochemical properties, as evidenced for deoxyfluorination in tuning the aggregation and assembly of fluorinated cellulose and cellodextrins [11, 12]. To serve as an adequate reporter group in binding studies, the deoxyfluorination should not perturb crucial intramolecular binding interactions and impact the overall structure of the glycan [4]. This was demonstrated recently in screening synthetic, fluorinated Lewis type 2 antigens against glycan binding proteins [13].

**FIGURE 1:**
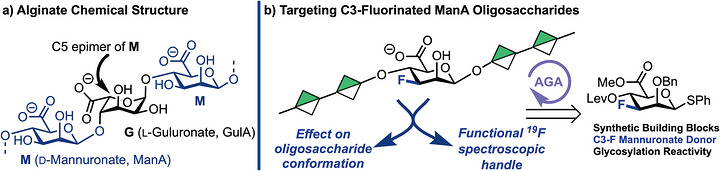
(a) Chemical structure of alginate composed of *cis*‐linked d‐mannuronic and l‐guluronic acids. (b) Introduction of C3‐deoxyfluorination within ManA and derived oligosaccharides.

Herein we explore the insertion and effect of C3 deoxyfluorination within d‐mannuronic acid (ManA) oligomers. ManA is a constituent part of alginate (Figure 1a), an unbranched, linear polysaccharide widely employed as a thickening and gelling agent in the food industry and as a component of wound dressings [14, 15, 16]. Alginate is also produced by two genera of bacteria, *Azobacter* and *Pseudomonas*, the latter contributing deleteriously to the lung biofilm matrices of cystic fibrosis and non‐CF bronchiectasis sufferers [17, 18].

For the synthesis of 3‐F ManA containing alginates, we first probed the effect of 3‐deoxy fluorination on the stereoselectivity of 3‐F ManA donors to find that this point mutation does not negatively affect the stereoselectivity of ManA *cis*‐glycosylations. We next implemented the building blocks in automated glycan assembly (AGA) [19] of a set of 3‐F alginates to deliver fluorinated ManA glycomimetics (Figure 1b). The generated oligosaccharides were used to explore conformational properties using NMR spectroscopy [13], demonstrating that whilst 3‐F precludes the existence of the canonical inter‐residue 3‐OH^…^O5 hydrogen bond, the major conformation around the glycosidic linkage is preserved. This approach completes a de facto site‐directed mutagenesis and benign incorporation of 3‐F ManA within defined alginate sequences, which will enable wider deployment of such tools as mimics of native alginate, to study its self‐assembly and enzymatic processing.

## 2 Results and Discussion

### 2.1 Synthesis and β‐Diastereoselective Glycosylation Capability of a C3‐Fluorinated ManA

Mannuronic acids are amongst the most stereoselective glycosylating agents for the effective installation of *cis*‐mannosidic linkages, and they have been employed in the assembly of long alginate and oligomannose fragments, both in solution and using AGA [19, 20, 21, 22, 23, 24, 25, 26]. However, incorporation of 3‐deoxy‐3‐fluorination for oligomannuronate synthesis is hitherto unexplored. Related studies on the use of 4,6‐*O*‐benzylidene‐3‐deoxy‐3‐fluoro‐d‐mannopyranosyl donors have been reported to show that these donors, in contrast to their C3‐oxy counterparts, showed poor diastereoselectivity in their glycosylations [27]. In addition, C2‐deoxyfluorination has been used in α‐selective mannosylations using AGA [28].

**FIGURE 2:**
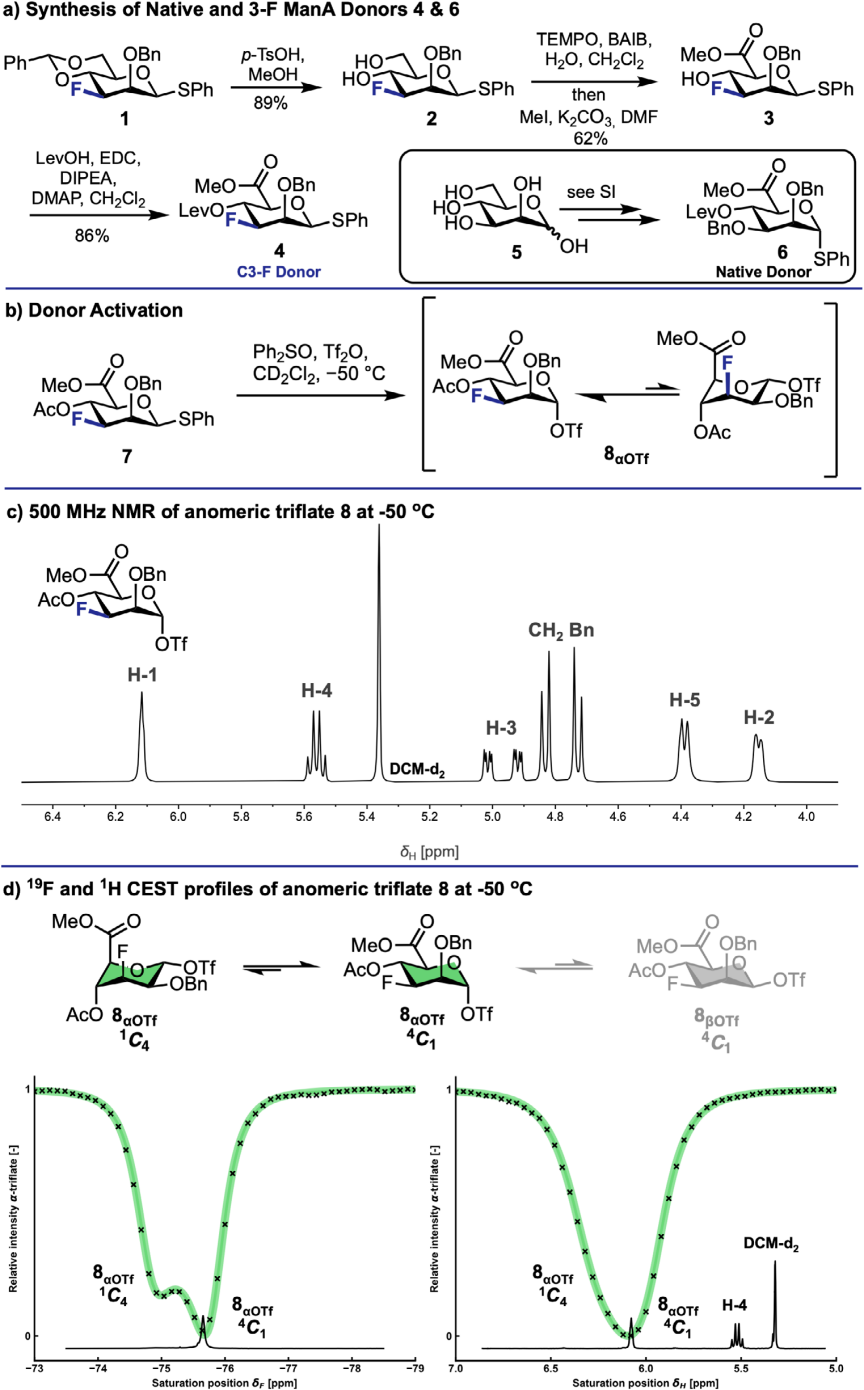
(a) Chemical synthesis of 3‐deoxy‐3‐fluoro‐d‐mannuronate donor **4** and native d‐mannuronate **6** (boxed). (b) Activation of 4‐*O*‐acetyl‐3‐deoxy‐3‐fluoro‐d‐mannuronate **7** cleanly provides the anomeric triflate **8**. (c) Key region of the ^1^H NMR spectrum (500 MHz, CD_2_Cl_2_) of anomeric triflate **8** with ring protons assigned. (d) ^1^H and ^19^F‐CEST‐NMR indicate the presence of the ring‐flipped ^1^C_4_ conformer in the mixture and the rapid interconversion between the ^4^C_1_ and the ^1^C_4_ chairs.

To access an appropriate donor monosaccharide for AGA, we first completed the synthesis of 3‐deoxy‐3‐fluoro‐mannuronate thioglycoside donor **4**, suitably equipped with a 4‐*O*‐Lev group to enable acceptor unmasking during iterative oligosaccharide synthesis (Figure 2a). The synthesis proceeded from intermediate **1**, itself accessed from commercial 1,2:5,6‐di‐*O*‐isopropylidene‐α‐d‐allofuranose (see Supporting Information S1) [27]. 4,6‐*O*‐Benzylidene deprotection was achieved by heating thioglycoside **1** to reflux with *p*‐TsOH in methanol, successfully providing the desired diol **2** in 89% yield, following purification by silica flash column chromatography. C6‐oxidation of diol **2** using TEMPO/BAIB followed by carboxylate methylation afforded C3‐fluoro mannuronic ester **3** in 62% yield over two steps. The synthesis of donor **4** was completed by the protection of the C4‐hydroxyl group as a levulinoyl ester. From the commercial starting material, thioglycoside **4** could be obtained in 9% yield over 13 steps, avoiding the need for chromatography until compound **1**, thus facilitating the provision of multigram quantities of the requisite donor for AGA. To provide an accompanying native d‐mannuronate building block, synthesis of α‐thioglycoside **6** was completed, starting from d‐mannose **5** (Figure 2a boxed, see Supporting Information S1) [19].

Next, we investigated the activation of the 3‐F ManA donors to explore the effect of the fluorine on the stereoselectivity of ManA glycosylations. As introduced above, mannuronic acid donors provide exceptional 1,2‐*cis* stereoselectivity and both the structure of the anomeric triflates, generated upon activation of the parent donors, and the structure of the mannuronic acid oxocarbenium ion have been shown to contribute to the excellent β‐stereoselectivity observed in such glycosylations [29]. The mannuronic acid oxocarbenium ion takes up a ^3^ *H*_4_ conformation, in which all ring substituents work in concert to stabilise the structure. Notably, the mannuronic acid triflates have been shown to preferentially adopt ^1^ *C*_4_ chair forms, which are in rapid equilibrium with their ^4^ *C*_1_ counterparts [30]. Accordingly, activation of thioglycoside **7** using Ph_2_SO/Tf_2_O was monitored using low‐temperature NMR, starting at −70°C in CD_2_Cl_2_ and, upon warming to −50°C, revealed a clean consumption of starting material **7** to provide triflate **8** (Figure 2b,c). Examination of the scalar coupling constants within **8** determined the ^4^ *C*_1_ conformer to be predominantly formed (^3^ *J*_H3‐H4_ = ^3^ *J*_H4‐H5_ = 9.1 Hz).

To further interrogate the nature of the activated 3‐deoxy‐3‐fluoro mannuronic acid donor, we employed exchange NMR techniques, building on recent studies by others [31, 32, 33]. In addition to variable‐temperature NMR experiments, ^1^H and ^19^F Chemical Exchange Saturation Transfer (CEST) NMR measurements were performed to probe the presence of low‐abundance intermediates and rapid exchange processes. Upon visual inspection of the CEST profiles, two exchanging species can be discerned in both ^1^H and ^19^F CEST NMR (Figure 2d), of which the chemical shifts are in accordance with the previously studied ring‐flipped mannuronic acid α‐triflates [32]. Importantly, ^19^F EXSY experiments revealed no exchange pathway involving triflate dissociation, demonstrating that the observed CEST signals originate exclusively from conformational ring‐flipping rather than anomeric interconversion. Therefore, these could be unambiguously assigned to the axial α‐glycosyl triflate and the ring‐flipped equatorial α‐triflate, rather than to an α/β anomeric triflate pair. The exceptionally fast interconversion between the two α‐triflate conformers necessitated cooling to −90°C to observe a discernible CEST signals, underscoring the remarkably low barrier for ring inversion in this system. In order to extract quantitative data from the experiments, the CEST data were fitted to the set of Bloch‐McConnell equations (Supporting Information S3: Equation S3). To this end, CEST profiles were recorded using varying saturation widths (20, 30, 40, and 60 Hz for ^1^H; 50, 7, 100, and 150 Hz for ^19^F) in order to fit these equations (see Supporting Information S3) [34, 35]. The best possible fits corresponded to a ratio of 1.0:0.15 ^4^ *C*_1_:^1^ *C*_4_ and 1.0:0.16 ^4^ *C*_1_:^1^ *C*_4_ for the ^1^H and ^19^F measurements, respectively.

Overall, the strong preference for the ^4^ *C*_1_ conformation stands in contrast to the conformational distribution of the corresponding native ManA derived triflate, which exists as a 1.0:1.3 ^4^ *C*_1_:^1^ *C*_4_ mixture [19], as well as previously reported C2‐modified systems (both C‐2‐azide and C‐2‐fluoride), which favoured a ^1^ *C*_4_ conformation [30]. The departure from this trend in triflate **8** to ^4^ *C*_1_ is presently unclear but may be a summation of different stereoelectronic effects. For example, the substituents at C3 and C5 will lead to destabilising dipoles in the ^1^ *C*_4_ conformer. At the same time, the interaction of the electron poor C‐5 carbonyl by electron donation from the C‐3 substituent will be less stabilising for the C‐3‐F than the C‐3‐*O* mannuronate. Triflate **8** was gradually warmed (with NMR spectra obtained in 10°C increments, see Supporting Information S2) until decomposition was observed at −20°C, notably higher than that reported for a native ManA triflate (−40°C). This was unsurprising as any build‐up of formal positive charge at the anomeric centre would be expected to be destabilised by the C3 fluorine.

To probe the structure of the 3‐F ManA oxocarbenum ion, we turned to the use of computational approaches, as the short lifetime of oxocarbenium ions precludes their characterisation under reaction‐like conditions [31]. The conformational energy landscape map (CEL map) of the mannuronic acid oxocarbenium ion has shown that this species has a very strong preference to adopt a ^3^ *H*_4_ conformation, which places the C‐3‐OR, C4‐OR as well as the C5‐CO_2_R groups in the most stabilising/least destabilising *pseudo*‐axial orientations. Accordingly, CEL maps were generated for the 3F ManA oxocarbenium ion (see Supporting Information S2) showing the *^3^H_4_* half chair conformation was also strongly favoured. Nucleophilic attack on this conformation is predicted to occur from the topside of the oxocarbenium ion, proceeding through a favourable β‐product forming ^1^ *C*_4_‐like transition state. Thus, the high relative stability of the ^3^ *H*_4_ conformation can offer a β‐selective glycosylation outcome for donor **4**, akin to native donor **6**.

Finally, having established the nature of the activated anomeric triflates and the conformational preferences of the corresponding oxocarbenium ion, we sought to experimentally assess the stereochemical outcome for glycosylation by the 3‐deoxy‐3‐fluoro mannuronosyl donor **4**. To test this, model glycosylations were performed with a systematic toolkit of ethanol‐based acceptors of decreasing nucleophilicity (ethanol, monofluoroethanol, difluoroethanol, and trifluoroethanol; see Table S7, Supporting Information S5). In all cases, glycosylation proceeded with exceptional and invariant β‐selectivity regardless of acceptor nucleophilicity. These results demonstrate that, for the 3‐F mannuronosyl donor, glycosylation displays pronounced stereochemical convergence: irrespective of whether bond formation proceeds through a more S_N_2‐like or S_N_1‐like regime as acceptor nucleophilicity is varied, β‐glycoside formation is consistently favoured. Thus, the mechanistic position along the substitution continuum does not translate into divergent stereochemical outcomes, in full agreement with the spectroscopic and computational analyses presented above.

### 2.2 Automated Glycan Assembly Incorporating a 3‐Deoxy‐3‐fluoro ManA Donor

Development of an AGA protocol to access modified ManA oligosaccharides via a 1,2‐*cis* selective glycosylation was investigated next using the commercially available Glyconeer 3.1 synthesiser [36]. Whilst clean activation of thioglycosides **4** and **6** was observed using Ph_2_SO/Tf_2_O (Figure 2), such a pre‐activation strategy cannot currently be applied to AGA and thioglycosides **4** and **6** were thus converted to their corresponding *N*‐phenyltrifluoroacetimidate (NPTFA) donors (see Supporting Information S1).

**FIGURE 3:**
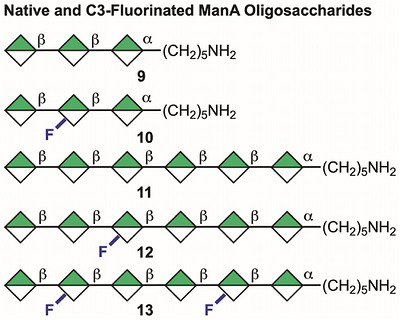
SNFG [38] representations of native and C3‐fluorinated ManA oligosaccharides accessed using AGA.

We first completed the synthesis of trisaccharides **9** and **10**, exploring capability of the fluorinated mannuronate as both a donor and an acceptor compared to the native ManA (Figure 3). We selected a linker‐functionalised Merrifield resin to deliver oligosaccharides containing an anomeric aminopentanol linker. Initial efforts using a traceless linker (ultimately resulting in a free sugar at the reducing terminus) resulted in product mixtures [37], containing small amounts of a GlcA (C2‐epimer) at the reducing‐terminus and were abandoned.

Five equivalents of ManA donor were used per coupling cycle with activation by stoichiometric TMSOTf in CH_2_Cl_2_ (see Supporting Information S3) An initial glycosylation temperature of −35°C was held for 30 min, followed by an increase in temperature to −10°C for 5 min. After each glycosylation a capping step acetylated any unreacted acceptor to ease purification of the final oligosaccharide from deletion sequences. Removal of the levulinoyl ester protecting group with hydrazine acetate was achieved at 40°C to reveal a new acceptor. Following the required AGA cycles and photocleavage of the protected oligosaccharides from the solid support, MALDI‐TOF analysis revealed trisaccharide formation was successful with no observed deletion sequences. Normal phase HPLC purification of the protected trisaccharide was followed by saponification, hydrogenolysis and purification by reverse phase HPLC to afford trisaccharide **9** in 10% overall yield and trisaccharide **10** in 6% overall yield, with respect to the resin loading. HSQC NMR analysis revealed that the reducing‐end linkage to the alkyl linker is α‐configured, while all inter‐saccharide linkages are β‐configured. Importantly, this observation does not match the model glycosylation reactions. We reasoned that this discrepancy could be due to more strenuous conditions required for AGA which could have led to anomerisation of the ManA residue connected to the linker. To probe this mechanistic hypothesis, we exposed ethyl β‐mannuronate **7A** to AGA coupling conditions (1.0 equiv. TMSOTf), while monitoring the mixture with variable‐temperature NMR experiments. We started the reaction at −40°C followed by warming to −10°C to show that the β‐linked glycoside undergoes rapid and clean anomerisation to the thermodynamically favoured α‐anomer (see Supporting Information S7).

Next, the synthesis of longer structures was performed, with hexasaccharides **11–13** containing zero, one or two internal fluorines targeted (Figure 3). AGA and deprotection proceeded smoothly, albeit with some deletion sequences detected by MALDI‐TOF analysis following photocleavage from the resin. Deprotections and purification were then completed to deliver oligosaccharides **11**, **12** and **13** in 12%, 6%, and 2% yields respectively, which were then analysed by NMR spectroscopy. Overall, these results are comparable to those producing GlcA‐ and ManA‐containing oligosaccharides (12%–16% yields for 8‐mers) [19, 39], noting that only one cycle of glycosylation was used here. The assignment of the oligosaccharides was achieved by combining the information recorded in ^1^H homonuclear (COSY, TOCSY, NOESY, ROESY) and heteronuclear ^1^H‐^13^C (HMQC, HMBC) and ^1^H‐^19^F 2D F‐relay‐[H]H‐TOCSY long‐range correlation experiments (see Supporting Information S6). Despite the signal overlap typical of homopolysaccharides, this analysis allowed the terminal residues to be readily identifiable in all cases. The reducing end α‐linkage (to the alkyl linker), and the non‐reducing end terminal residue showed distinct patterns that were repeated across all compounds (see Supporting Information S9). Notwithstanding the reducing end residue, this analysis confirmed the prevalence of all β‐linkages in the remainder of the sequences, and the functional capability of C3‐fluorinated ManA donor **4** in effecting 1,2‐*cis* selective glycosylation.

### 2.3 Characterisation and Conformational NMR Analysis of Fluorinated ManA Oligosaccharides

**FIGURE 4:**
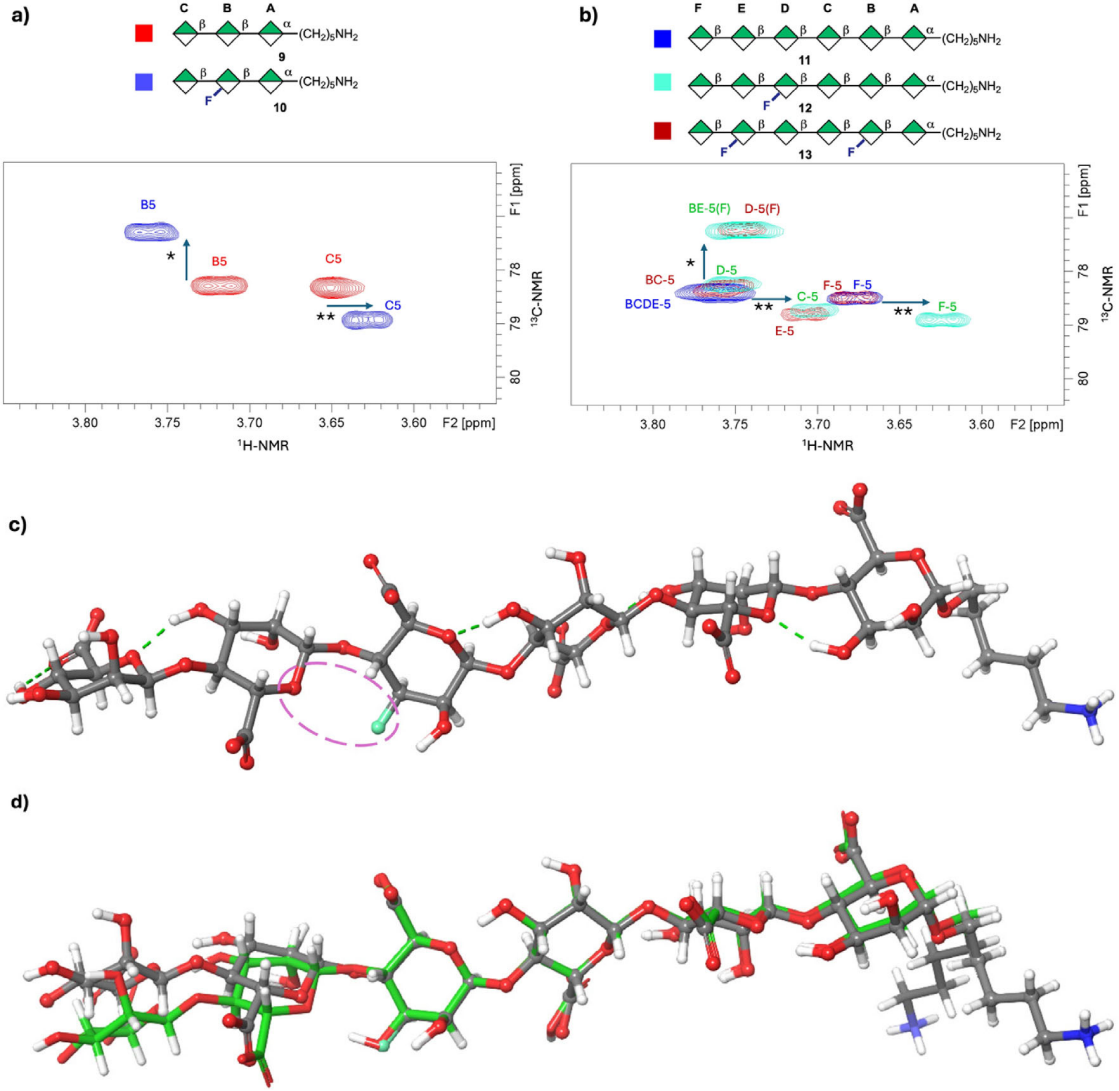
The ^1^H and ^1^ ^3^C chemical shifts at ring position 5 are strongly influenced by the presence of the fluorine atom at position 3. The effects on the same (B, D, or BE) and adjacent (C, E, or CF) residues of **10**, **12**, and **13** are compared to those of **9** and **11**. Superimposition of sections of the ^1^H‐^13^C HSQC spectra of: (a, b) **9** (red) versus **10** (blue) **b) 11** (red), **12** (turquoise), and **13** (blue) spectra are shown. Fluorination at position 3 induces an intra‐residue ^1^ ^3^C shielding effect (*) and a ^1^H shielding and ^13^C deshielding effect at the following residue (**). (c) Molecular model of compound **12** highlighting the canonical 3‐OH_i_.O5_i+1_ hydrogen bond, typical of β‐1‐4 linked disaccharide fragments with equatorial 3‐OH groups. The presence of the fluorine at position C3 (green) precludes the existence of the corresponding hydrogen bond (dashed magenta oval). (d) Overlay of molecular models of non‐fluorinated **11** (green) and monofluorinated **12** (grey) compounds, using D residue for the superimposition.

Conformational analysis was carried out next by combining NMR experiments with molecular modelling protocols. Chemical shifts measured for the fluorinated oligosaccharides were compared to those of the parent analogues without fluorine (**10** vs. **9**, **12** and **13** vs. **11**). Interestingly, fluorination at C3 in ManA exerted ^1^H shielding and ^13^C deshielding effects at C5 in the adjacent residue (**, Figure 4). This experimental evidence can be satisfactorily explained by disruption of the inter‐residue C3‐OH^….^O5 hydrogen bond that is ubiquitous in β‐1,4 linked disaccharide fragments with equatorial C3 hydroxyl groups [40]. An observed shielding effect at C5 of the fluorinated residue (*, Figure 4) can be readily explained by the OH to F substitution within the ring.

The conformational analysis was based on the evaluation of inter‐residue cross peaks measured in NOESY (400–500 ms) and/or ROESY (300 ms) experiments. The isolated spin pair approximation (ISPA) was used to estimate the averaged inter‐proton distances using the intra‐residue (H1‐H3/5) distances (ca. 2.5 Å each) as internal standard. Indeed, for all compounds, strong H1‐H3/5 NOEs were observed for the H1 anomeric protons of the β‐linked residues, which are typical for ^4^ *C*_1_ chair conformations [41]. For the reducing end, with an α‐configuration, a strong H1‐H2 cross peak was observed. In all cases, strong NOEs across the glycosidic linkage between H1 of a given residue and H4 of the preceding one were scrutinised, with similar intensities to the H1/H3,H5 intra‐residue proton pairs. This fact supports that the average H1i‐H4i+1 distances are ca. 2.5 Å. These distances agree with a major *exo*‐anomeric‐*syn* conformation around *Φ* angle and a *syn*‐Ψ geometry, completely in line with the conformation around β‐1,4 glycosidic linkages in natural oligosaccharides. Furthermore, molecular mechanics calculations provided this conformation as the most favourable geometry (see Supporting Information S9 and Figure 4). Overall, this analysis indicates that C3 deoxyfluorination only induces minor differences in the overall conformation, preserving the conformational preference of the native oligomer.

Inter‐residue hydrogen bonds are frequently invoked as key contributors to glycan conformational preferences. However, their role must be considered within a broader framework that includes stereoelectronic effects, steric constraints, and solvation. In many cases, these hydrogen bonds are relatively weak and emerge as a consequence of an already favourable spatial arrangement rather than acting as the primary driving force for conformational selection. A well‐known example is provided by Lewis X systems, where the closed conformation is associated with a non‐classical C–H···O hydrogen bond involving the fucose H5 proton. Importantly, this interaction is enabled by the preorganization of the glycan and coexists with other stabilizing effects, rather than acting in isolation as a conformational determinant [42]. Furthermore, an analogous case for contiguous α‐1,4‐linked Gal/GalN/GalNAc residues has been reported [43].

In this context, the removal of a single hydrogen‐bond donor or acceptor is not necessarily expected to induce a major conformational rearrangement. Instead, such interactions often reinforce a preferred rotamer rather than define it. This concept is supported by recent work which shows that deletion of an inter‐residue hydrogen bond in a disaccharide by deoxygenation does not alter the preferred linkage geometry but rather modulates the stability of an already populated conformational state [44]. Importantly, this behaviour is also consistent with earlier observations. In a previous study on lactose derivatives [45], the HO‐3···O‐5′ inter‐residue hydrogen bond was eliminated by substitution of the hydroxyl group (e.g., by hydrogen or methyl). Despite removal of this interaction, the distribution of low‐energy conformers in solution remained essentially unchanged, indicating that this hydrogen bond was not an essential determinant of the preferred conformation, but rather a stabilising interaction compatible with it. Most recently, in work on selectively deoxyfluorinated LacNAc analogues [46], a similar trend was noted: disruption or modulation of the O5′···H–O3 hydrogen bond does not alter the dominant typical Φ/Ψ conformational preference but instead affects the fine details of the conformational ensemble, such as local flexibility and population distribution.

Taken together, these observations support a general picture in which inter‐residue hydrogen bonds in glycans frequently act as fine‐tuning stabilising interactions, rather than primary conformational determinants. Within this framework, the conformational preservation observed in the present study (upon deoxyfluorination) indicates that the disrupted hydrogen bonds do not alter the intrinsically preferred geometry of the linkage, which is governed by the underlying stereoelectronic and steric features of the whole system.

## 3 Conclusion

We have established an efficient building block assembly for C3‐deoxy‐3‐fluoro mannuronates and completed a primary exploration of their capability in effecting 1,2‐*cis* selective glycosylations. The donors could be readily activated to provide the corresponding anomeric triflates, that showed rapid conformational exchange between ^4^ *C*_1_ and ^1^ *C*_4_ chair conformers. This behaviour mirrors that of the parent 3‐oxy ManA donors, although the exchange is significantly more rapid for 3‐F ManA and this system adopts a strong preference for a ^4^ *C*_1_ conformation. Using computational chemistry, we established that the structure of potential oxocarbenium ion intermediates (or by extension structures that feature strong oxocarbenium ion character) also closely resemble the structure of the ManA oxocarbenium, with the ^3^ *H*_4_ half‐chair conformer being the most favourable oxocarbenium ion structure. This conformation and the preference for a ^4^ *C*_1_ α‐anomeric triflate contribute to the observed exceptional β‐stereoselectivity of these 3‐F ManA donors. This enabled automated, solid‐supported oligosaccharide assembly, and access to a first series of β‐1,4‐d‐mannuronic acid oligosaccharides containing bespoke C3‐fluoro modification of distinct residues within the chain. Detailed NMR analysis of these sequences shows that, importantly, although the presence of the fluorine atom at C3 precludes the existence of the canonical inter‐residue 3‐OH^…^O5 hydrogen bond, the major conformation around the glycosidic linkage is preserved [47]. This finding demonstrates the suitability and potential of fluorine as a benign hydroxyl bioisostere and capability in effecting a means of site‐directed mutagenesis, for carbohydrates.

These fluorinated oligosaccharides will be useful probes to unpick the specificity of enzymes that process alginates. For example, within *Pseudomonas aeruginosa* alginate molecular assembly the acetylation complex enzymes AlgJ and AlgX are poorly understood, and fluorinated motifs should be able to resolve which hydroxyl group they acetylate [48]. Furthermore, fluorine incorporation within oligomannuronate sequences provides a convenient and sensitive spectroscopic tool that will enable reporter capability to quickly screen for interactions between ManA oligomers and cations. This will support fully correlating the relationships between structure and function in alginate cross‐linking interactions that determine the ultimate viscosity of alginate polysaccharide gels.

## Author Contributions

**Sean T. Evans**: writing – original draft, investigation, methodology, writing – review and editing, data curation. **Nishu Yadav**: investigation, writing – original draft, writing – review and editing, methodology, data curation. **Wouter A. Remmerswaal**: investigation, methodology, writing – original draft, writing – review and editing, data curation. **Daan Hoogers**: investigation, data curation, methodology, writing – review and editing, writing – original draft. **Koen N. A. van de Vrande**: methodology, data curation, investigation, writing – review and editing. **Sarah Hosking**: funding acquisition, supervision, writing – review and editing. **Ana Poveda**: methodology, writing – review and editing, investigation, data curation. **Jeroen D. C. Codée**: funding acquisition, methodology, writing – review and editing, supervision. **Jesús Jiménez‐Barbero**: funding acquisition, writing – review and editing, supervision, methodology. **Martina Delbianco**: methodology, funding acquisition, supervision, writing – review and editing, writing – original draft. **Gavin J. Miller**: conceptualization, project administration, supervision, writing – original draft, writing – review and editing, funding acquisition, methodology.

## Conflicts of Interest

The authors declare no conflicts of interest.

## Supporting information




**Supporting File**: anie72871‐sup‐0001‐SuppMat.pdf.

## Data Availability

The data that support the findings of this study are available in the Supporting Information of this article.
