# Supplementary material for: 3‐Deoxy‐3‐Fluoro Mannuronic Acid Alginates: Stereoselective Automated Synthesis and Conformational Behaviour
Source: Angew Chem Int Ed Engl. 2026 May 28;65(31):e5914227. doi: 10.1002/anie.5914227 (PMC13411212; doi:10.1002/anie.5914227)
Supplement: Supplementary file 1 — Supporting File: anie72871‐sup‐0001‐SuppMat.pdf. [file ANIE-65-e5914227-s001.pdf]

Supporting Information

3-Deoxy-3-Fluoro Mannuronic Acid Alginates: Stereoselective Automated Synthesis and Conformational Behaviour

Sean T. Evans,[1] Nishu Yadav,[2] Wouter A. Remmerswaal,[3] Daan Hoogers,[3] Koen N. A. van de Vrande,[3] Sarah Hosking,[4] Ana Poveda,[5-8] Jeroen D. C. Codée,[3] Jesús Jiménez-Barbero,[5-8] Martina Delbianco[2] and Gavin J. Miller[1,9*]

[1] School of Chemical and Physical Sciences & Centre for Glycoscience, Keele University, Keele, Staffordshire, ST5 5BG, U.K.
[2] Department of Biomolecular Systems, Max Planck Institute of Colloids and Interfaces, Am Mühlenberg 1, 14476, Potsdam, Germany
[3] Leiden Institute of Chemistry, Leiden University, 2333 CC Leiden, Netherlands
[4] Unilever Research and Development, Quarry Rd East, Bebington, Birkenhead, Wirral CH63 3JW
[5] CICbioGUNE, Basque Research and Technology Alliance, Parque Científico Tecnológico de Bizkaia Building 801A, E-48160 Derio, Spain.
[6] Ikerbasque, Basque Foundation for Science, Plaza Euskadi 5, E-48009 Bilbao, Spain.
[7] Department of Inorganic & Organic Chemistry, Faculty of Science and Technology, University of the Basque Country, EHU, E-48940 Leioa, Spain.
[8] Centro de Investigación Biomedica En Red de Enfermedades Respiratorias, Fuencarral-El Pardo, E-28029 Madrid, Spain.
[9] Manchester Institute of Biotechnology & Department of Chemistry, University of Manchester, Manchester, M1 7DN, UK

Table of Contents

S1. Synthesis of Building Blocks 2
S2. Anomeric Triflate Formation and Degradation 20
S3. Exchange NMR analysis of triflate **8** 21
S4. Conformational Energy Landscapes 31
S5. Solution-phase glycosylation reactions of donor **7** 33
S6. Automated Glycan Assembly 36
S7. Anomerisation kinetics for compound **7a** 48
S8. 1D NMR spectra 51
S9. 2D NMR Spectra of Oligosaccharides and Conformational Analysis 85
S10. References 96

S1. Synthesis of Building Blocks

1.1 General experimental

All reagents and solvents were purchased from commercial sources (Sigma Aldrich, Alfa Aesar, Fluorochem, Biosynth, Apollo Scientific, TCI, Fisher Scientific) and were used without further purification. Reactions that required anhydrous conditions were conducted under an atmosphere of N_2 using pre-dried glassware. Anhydrous solvents were either obtained from a Pure Solv-400-3-MD solvent purification system (Innovative Technology Inc.) or were purchased with Sure/Seal™ or AcroSeal™ packaging. Reaction monitoring was accomplished by thin-layer chromatography using Merck TLC Silica gel 60 F_{254} aluminimum sheets. Following development, TLC plates were observed under 254 nm UV light and were then coated in 5% (v/v) solution of H_2SO_4 in EtOH and heated until staining occurred. Column chromatography used Aldrich technical grade silica gel, pore size 60 Å, 230-400 mesh particle size, 40-63 µm particle size. The volumes of solvents and solutions reported for work-ups and washes have been estimated and were not accurately measured. Lyophilisation was conducted using a Labconco FreeZone Benchtop freeze dryer. NMR spectra were recorded on a Bruker Ascend 400 NMR spectrometer. ^{1}H NMR spectra were referenced to tetramethylsilane, except for those recorded in D_2O which were referenced to the residual solvent peak. The multiplicity of signals is indicated by the following abbreviations: s (singlet), d (doublet), t (triplet), q (quartet), dd (doublet of doublets), ddd (doublet of doublets of doublets), dddd (doublet of doublets of doublets of doublets), dt (doublet of triplets), td (triplet of doublets), m (multiplet), br (broad). In **^{13}C {^{1}H} NMR** assignments, (CH) refers to an aromatic carbon bonded to hydrogen and (C_q) refers to a quaternary aromatic carbon. *J* coupling values in **^{1}H NMR** spectra are provided to one decimal place. High resolution mass spectrometry data was provided either by the Keele University Faculty of Natural Sciences Mass Spectrometry facility or by the National Mass Spectrometry Facility at Swansea University. Optical rotation measurements were conducted using a Bellingham+Stanley digital polarimeter (specific rotation, tube length: 50 mm, concentrations in g per 100 mL). Melting point measurements were conducted using a Cole-Palmer Stuart SMP10 digital melting point apparatus.

1.2 Synthesis of 3-F-ManA Thioglycosides 4 and 7

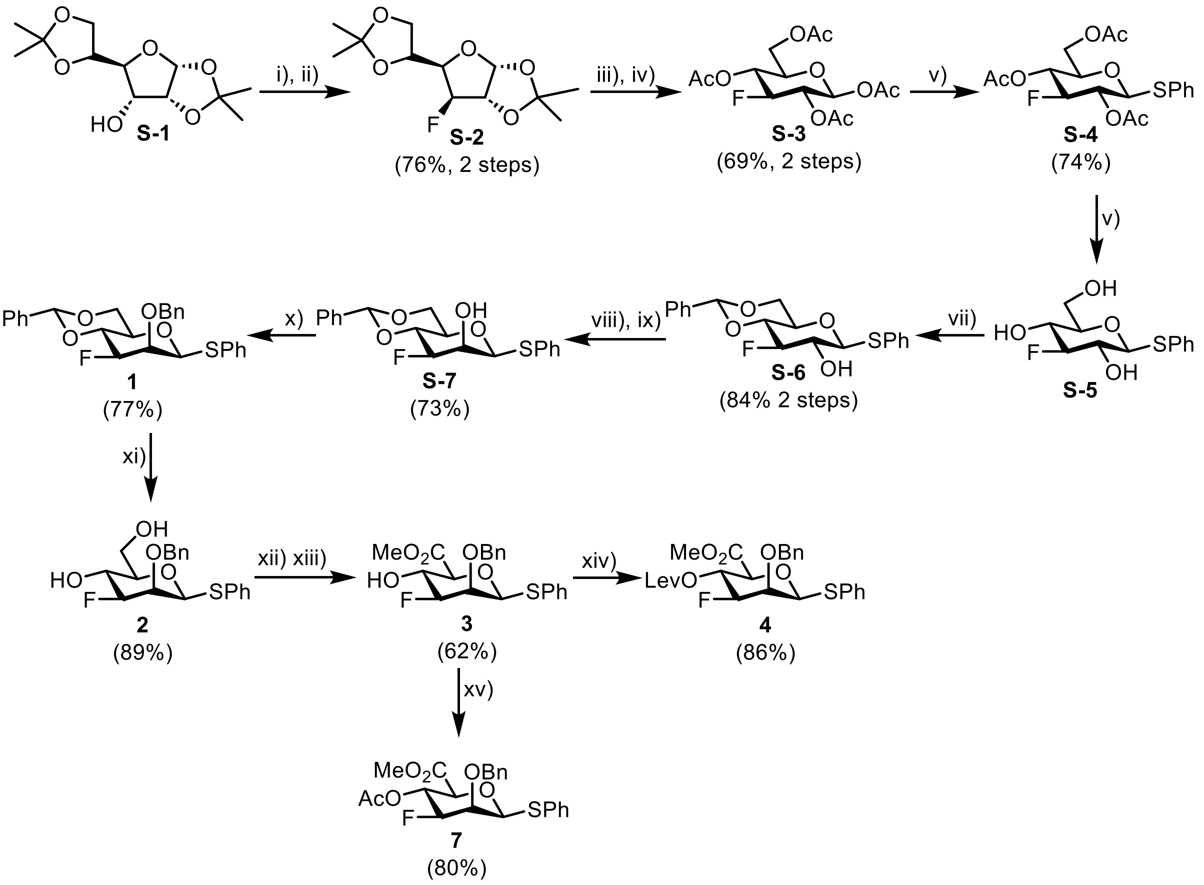

Reaction conditions: ***i)*** *Tf_2O, pyridine;* ***ii)*** *CsF, t-BuOH;* ***iii)*** *0.1M H_2SO_4;* ***iv)*** *NaOAc, Ac_2O;* ***v)*** *PhSH, $BF_3 \cdot Et_2O$, CH_2Cl_2;* ***vi)*** *Na_2CO_3, MeOH;* ***vii)*** *$PhCH(OMe)_2$, (±) CSA, MeCN;* ***viii)*** *DMP, CH_2Cl_2;* ***ix)*** *L-selectride, THF;* ***x)*** *BnBr, NaH, DMF;* ***xi)*** *p-TsOH·H_2O, MeOH;* ***xii)*** *TEMPO, BAIB, H_2O, CH_2Cl_2;* ***xiii)*** *MeI, K_2CO_3, DMF;* ***xiv)*** *LevOH, EDC·HCl, DIPEA, DMAP, CH_2Cl_2;* ***xv)*** *Ac_2O, pyridine, DMAP, CH_2Cl_2.*

3-deoxy-3-fluoro-1,2:5,6-diisopropylidene-α-D-glucofuranose (S-2)

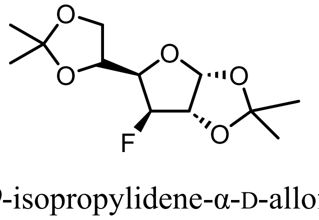

To a stirred solution of 1,2:5,6-di-*O*-isopropylidene-α-D-allofuranose (**S-1**) (20.0 g, 76.8 mmol) in pyridine (154 mL) at 0 °C was added Tf_2O (14.9 mL, 88.5 mmol). The mixture was stirred at 0 °C for one hour, then concentrated *in vacuo*. The resulting residue was diluted with EtOAc (250 mL) and washed with saturated aqueous $NaHCO_3$ (125 mL), brine (125 mL), dried

($MgSO_4$) and concentrated in vacuo. The crude triflate was dissolved in *t*-butanol (256 mL). Caesium fluoride (35.0 g, 230 mmol) was added, and the reaction was heated to reflux for 22 h. The mixture was then cooled to room temperature, concentrated *in vacuo* and dissolved in ethyl acetate (250 mL). The organic layer was washed with saturated aqueous $NaHCO_3$ (125 mL), water (125 mL), brine (125 mL), dried ($MgSO_4$) and concentrated *in vacuo*. The crude product was dissolved in diethyl ether (250 mL) and filtered through a plug of silica to afford the title compound **S-2** (30.6 g, 11.7 mmol, 76%) as an orange oil. R_f = 0.4 (2.5% MeOH/CH_2Cl_2); **^{1}H NMR** (400 MHz; $CDCl_3$); 5.95 (1H, d, J = 3.7 Hz, H-1), 5.01 (1H, dd, J = 49.8, 2.3 Hz, H-3), 4.70 (1H, dd, J = 10.5, 4.7 Hz, H-2), 4.29 (1H, ddd, J = 8.3, 6.1, 4.7 Hz, H-5), 4.12 (1H, dd, J = 8.6, 5.9 Hz, H-6), 4.11 (1H. ddd. J = 29.2, 8.1, 2.3 Hz, H-4), 4.03 (1H, dd, J = 8.7, 4.8 Hz, H-6'), 1.50 (3H, s, CH_3), 1.45 (3H, s, CH_3), 1.37 (3H, s, CH_3), 1.33 (3H, s, CH_3);); **^{13}C {^{1}H} NMR** (101 MHz; $CDCl_3$) δ 112.4 ($C(CH_3)_2$), 109.5 ($C(CH_3)_2$), 105.2 (C1), 93.9 (d, $^1J_{C\text{-}F}$ = 183.8 Hz, C3), 82.6 (d, $^2J_{C\text{-}F}$ = 32.8 Hz, C2), 80.7 (d, $^2J_{C\text{-}F}$ = 19.0 Hz, C4), 71.9 (d, $^3J_{C\text{-}F}$ = 7.1 Hz, C5), 67.2 (d, $^4J_{C\text{-}F}$ = 1.4 Hz, C6), 26.9 (CH_3), 26.7 (CH_3), 26.2 (CH_3), 25.2 (CH_3); **^{19}F NMR** (376 MHz; $CDCl_3$) δ −207.57 (ddd, J = 50.0, 29.2, 10.7 Hz); **HRMS** (ESI) *m/z* found: $(M+Na)^+$ 285.1114, $C_{12}H_{19}FO_5Na$ requires 285.1114. Data matched those previously reported.[1]

1,2,4,6-Tetra-*O*-acetyl-3-deoxy-3-fluoro-β-D-glucopyranose (S-3)

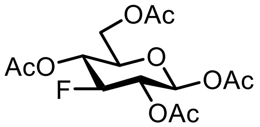

A stirred suspension of compound **S-2** (36.8 g, 141 mmol, 1 eq.) in 0.1 M sulfuric acid (450 mL) was heated to 60 °C for 16 h. The reaction mixture was then cooled to 0 °C, neutralised with saturated aqueous $NaHCO_3$, concentrated and dried under vacuum. To the resulting solid was added sodium acetate (5.77 g, 70.3 mmol. 0.5 eq.) and acetic anhydride (132 mL). The mixture was heated to reflux for 5 min then was allowed to cool to room temperature. The reaction mixture was then poured over ice and stirred until the product precipitated as a brown solid. The crude product was filtered and recrystallised from methanol to afford the title compound **S-3** (35.3 g, 96.7 mmol, 69%) as a white crystalline solid. R_f= 0.8 (60% EtOAc/*n*-hexane); m.p. 119-121 °C; **^{1}H NMR** (400 MHz; $CDCl_3$) δ 5.66 (1H, d, J = 8.4 Hz, H-1), 5.32-5.22 (2H, m, H-2, H-4), 4.61 (1H, ddd, J = 52.0, 9.1, 9.1 Hz, H-3), 4.28 (1H, dd, J = 12.5, 4.6 Hz, H-6a), 4.13 (1H, ddd, J = 12.5, 2.0, 2.0 Hz, H-6b), 3.75 (1H, dddd, J = 10.1, 4.5, 2.2, 1.2 Hz, H-5), 2.13 (1H, s, CH_3 Ac), 2.12 (1H, s, CH_3 Ac), 2.12 (1H, s, CH_3 Ac), 2.10 (1H, s, CH_3 Ac); **^{13}C {^{1}H} NMR** (101 MHz; $CDCl_3$) δ 169.6 (C=O Ac), 168.1 (C=O Ac), 168.0 (C=O Ac),

168.0 (C=O Ac), 90.6 (d, $^1J_{C\text{-}F}$ = 191.8 Hz, C3), 90.1 (d, $^3J_{C\text{-}F}$ = 11.3 Hz, C1), 70.9 (d, $^3J_{C\text{-}F}$ = 7.7 Hz, C5), 69.2 (d, $^2J_{C\text{-}F}$ = 19.1 Hz, C2), 66.6 (d, $^2J_{C\text{-}F}$ = 18.7 Hz, C4), 60.2 (d, $^4J_{C\text{-}F}$ = 1.8 Hz, C6), 19.8 (CH_3 Ac), 19.7 (CH_3 Ac), 19.6 (CH_3 Ac), 19.6 (CH_3 Ac); **^{19}F NMR** (376 MHz; $CDCl_3$) δ −196.0 (ddd, *J* = 52.0, 12.6, 12.6 Hz); **HRMS** (ESI) *m/z* found: $(M+Na)^+$ 373.0901, $C_{14}H_{19}FO_9Na$ requires 373.0911. Data matched those previously reported.[2]

Phenyl 2,4,6-tri-*O*-acetyl-3-deoxy-3-fluoro-1-thio-β-D-glucopyranoside (S-4)

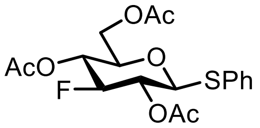

To a stirred solution of compound **S-3** (28.4 g, 81.2 mmol, 1.0 eq.) in CH_2Cl_2 (91 mL) at 0 °C was added thiophenol (12.4 mL, 121 mmol, 1.5 eq.) followed by dropwise addition of $BF_3{\cdot}Et_2O$ (24.8 mL, 200 mmol, 2.5 eq.). The mixture was stirred at 0 °C for 8 h and was then quenched with saturated aqueous sodium bicarbonate. The mixture was diluted with CH_2Cl_2 (300 mL) and the organic layer was separated. The organic layer was washed with water (100 mL), dried ($MgSO_4$) and concentrated *in vacuo* to afford a brown solid. The crude product was purified by recrystallisation from MeOH to afford the title compound **S-4** (24.0 g, 59.8 mmol, 74%) as a white crystalline solid. R_f = 0.3 (30% EtOAc/*n*-hexane); m.p. 122-124 °C; **^{1}H NMR** (400 MHz; $CDCl_3$) δ 7.54-7.47 (2H, m, Ar-H), 7.35-7.28 (3H, m, Ar-H), 5.22-5.04 (2H, m, H-2, H-4), 4.63 (1H, dd, *J* = 10.2, 1.0 Hz, H-1), 4.57 (1H, ddd, *J* = 52.2, 9.0. 8.9 Hz, H-3), 4.22-4.19 (2H, m, H-6a, H-6b), 2.16 (3H, s, CH_3 Ac), 2.10 (3H, s, CH_3 Ac), 2.09 (3H, s, CH_3 Ac); **^{13}C {^{1}H} NMR** (101 MHz; $CDCl_3$) δ 170.6 (C=O Ac), 169.2 (C=O Ac), 169.0 (C=O Ac), 133.1 (CH), 131.7 (C_q), 129.0 (CH), 128.5 (CH), 92.6 (d, *J* = 193.3 Hz, C3), 85.3 (d, $^3J_{C\text{-}F}$ = 7.8 Hz, C1), 75.2 (d, $^3J_{C\text{-}F}$ = 7.3 Hz, C5), 69.7 (d, $^2J_{C\text{-}F}$ = 18. 5 Hz, C2), 68.2 (d, $^2J_{C\text{-}F}$ = 18.4 Hz, C4), 62.1 (d, $^4J_{C\text{-}F}$ = 1.8 Hz, C6), 20.8 (CH_3 Ac), 20.7 (CH_3 Ac), 20.7 (CH_3 Ac); **^{19}F NMR** (376 MHz; $CDCl_3$) δ −192.5 (ddd, *J* = 52.2, 12.2, 12.2 Hz); **HRMS** (ESI) *m/z* found: $(M+NH_4)^+$ 418.1328, $C_{18}H_{25}FO_7SN$ requires 418.1336. Data matched those previously reported.[3]

Phenyl 3-deoxy-3-fluoro-1-thio-β-D-glucopyranoside (S-5) and Phenyl 4,6-*O*-benzylidene-3-deoxy-3-fluoro-1-thio-β-D-glucopyranoside (S-6)

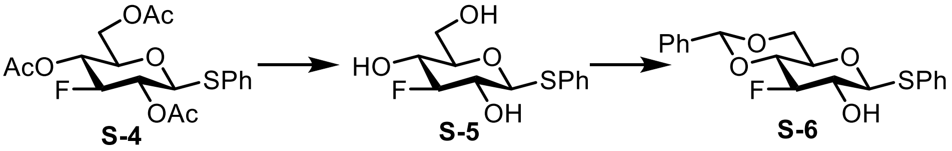

A suspension of compound **S-4** (24.0 g, 59.8 mmol, 1.0 eq.) and sodium carbonate (700 mg, 6.60 mmol, 0.1 eq.) in MeOH (260 mL) was stirred at room temperature for 16 h. The reaction

mixture was neutralised by the addition of Amberlite IR120 (H^+) ion exchange resin, filtered and concentrated *in vacuo* to afford title compound **S-5** as a white solid. R_f = 0.05 (40% EtOAc/*n*-hexane); **^{1}H NMR** (400 MHz; MeOD) δ 7.60-7.53 (2H, m, Ar-H), 7.37-7.21 (3H, m, Ar-H), 4.61 (1H, dd, *J* = 9.8, 0.9 Hz, H-1), 4.27 (1H, ddd, *J* = 52.6, 8.6, 8.6 Hz, H-3), 3.87 (1H, ddd, *J* = 12.1, 2.3, 1.3 Hz, H-6a), 3.69 (1H, dd, *J* = 12.2, 5.4 Hz, H-6b), 3.56 (1H, ddd, *J* = 13.8, 9.9, 8.7 Hz, H-4), 3.43 (1H, ddd, *J* = 13.3, 9.9, 8.5 Hz, H-2), 3.36-3.28 (1H, m, H-5); **^{13}C {^{1}H} NMR** (101 MHz; MeOD) δ 133.3 (C_q), 131.7 (CH), 128.5 (CH), 127.2 (CH), 98.2 (d, $^1J_{C\text{-}F}$ = 184.9 Hz, C3), 87.1 (d, $^3J_{C\text{-}F}$ = 8.8 Hz, C1), 79.6 (d, $^3J_{C\text{-}F}$ = 7.6 Hz, C5), 70.6 (d, $^2J_{C\text{-}F}$ = 18.4 Hz, C2), 68.2 (d, $^2J_{C\text{-}F}$ = 18.3 Hz, C4), 61.0 (d, $^4J_{C\text{-}F}$ = 1.8 Hz, C6); **^{19}F NMR** (376 MHz; MeOD) δ −192.3 (ddd, *J* = 52.7, 13.6, 13.6 Hz); **HRMS** (ESI) *m/z* found: $(M+Na)^+$ 297.0566, $C_{12}H_{15}FO_4SNa$ requires 297.0573. Crude compound **S-5** was dissolved in MeCN (197 mL) and benzaldehyde dimethyl acetal (11.2 mL, 74.4 mmol, 1.24 eq.) and (±) CSA (1.44 g, 62.0 mmol, 1.0 eq.) were added. The mixture was stirred at room temperature for 3 h after which time product **S-6** (~8 g) had precipitated as a white solid. The reaction mixture was filtered, product **S-6** was kept aside, and the filtrate was concentrated *in vacuo* until approximately half of the solvent remained. Fresh MeCN (100 mL) was added and the mixture was stirred at room temperature for 16 h. The reaction was then quenched with NEt_3 and concentrated *in vacuo*. The resulting residue was triturated with Et_2O:pet. ether (1:1, ~500 mL) and the two batches of product were combined to afford the title compound **S-6** (18.2 g, 50.1 mmol, 84%) as a white solid. R_f = 0.6 (30% EtOAc/*n*-hexane); m.p. 105-107 °C; **^{1}H NMR** (400 MHz; $CDCl_3$) δ 7.59-7.52 (2H, m, Ar-H), 7.52-7.45 (2H, m, Ar-H), 7.41-7.32 (6H, m, Ar-H), 5.54 (1H, s, C*H*Ph), 4.64 (1H, ddd, *J* = 53.5, 9.2, 8.2 Hz, H-3), 4.62 (1H, d, *J* = 9.8 Hz, H-1), 4.41 (1H, ddd, *J* = 10.6, 5.0, 2.1 Hz, H-6a), 3.80 (1H, dd, *J* = 10.3, 10.3, Hz, H-6b), 3.73 (1H, ddd, *J* = 11.3, 9.5, 9.5 Hz, H-4), 3.65 (1H, ddd, *J* = 14.1, 10.0, 8.4 Hz, H-2), 3.50 (1H, dddd, *J* = 9.8, 9.8, 5.0, 1.4 Hz), 2.70 (1H, br. s, H); **^{13}C {^{1}H} NMR** (101 MHz; $CDCl_3$) δ 136.6 (C_q), 133.4 (CH), 130.7 (C_q), 129.3 (CH), 129.3 (CH), 128.8 (CH), 128.4 (CH), 126.2 (CH), 101.7 (*C*HPh), 93.3 (d, $^1J_{C\text{-}F}$ = 190.1 Hz, C3), 88.3 (d, $^3J_{C\text{-}F}$ = 7.7 Hz, C1), 78.7 (d, $^2J_{C\text{-}F}$ = 17.2 Hz, C4), 71.4 (d, *J* = 18.3 Hz, C2), 69.8 (d, $^3J_{C\text{-}F}$ = 7.8 Hz, C5), 68.5 (d, $^4J_{C\text{-}F}$ = 1.4 Hz, C6); **^{19}F NMR** (376 MHz; $CDCl_3$) δ −192.7 (ddd, *J* = 53.3, 12.5, 12.5 Hz); **HRMS** (ESI) *m/z* found: $(M+Na)^+$ 385.0880, $C_{19}H_{19}FO_4SNa$ requires 385.0886. Data matched those previously reported.[3]

Phenyl 4,6-*O*-benzylidene-3-deoxy-3-fluoro-1-thio-β-D-mannopyranoside (S-7)

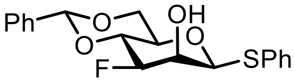

To a stirred solution of compound **S-6** (260 mg, 0.717 mmol, 1.0 eq.) in CH_2Cl_2 (2.6 mL) was added Dess-Martin periodinane (365 mg, 0.860 mmol, 1.2 eq.). The mixture was stirred at room temperature for 2 h. The reaction mixture was then diluted with CH_2Cl_2 (5 mL) and washed with saturated aqueous $Na_2S_2O_3$ (5 mL), brine (5 mL), dried ($MgSO_4$) and concentrated *in vacuo*. The resulting residue was dissolved in THF (2.6 mL) and cooled to −78 °C. L-selectride (1.0 M in THF, 1.43 mL, 1.43 mmol, 2.0 eq.) was added and the mixture was stirred at −78 °C for 20 min. The reaction was then quenched with water (5 mL) and allowed to warm to room temperature. The mixture was diluted with CH_2Cl_2 (5 mL) and washed with saturated aqueous sodium bicarbonate (5 mL), brine (5 mL), dried ($MgSO_4$) and concentrated *in vacuo*. The crude product was purified by column chromatography on silica gel (15% EtOAc/n-Hexane) to afford compound **S-7** (191 mg, 0.527 mmol, 73%) as an off-white solid. R_f = 0.3 (30% EtOAc/*n*-hexane); m.p. 186-188 °C; **^{1}H NMR** (400 MHz; $CDCl_3$) δ 7.54-7.45 (4H, m, Ar-H), 7.40-7.27 (6H, m, Ar-H), 5.59 (1H, s, C*H*Ph), 4.91 (1H, dd, *J* = 1.5, 1.5 Hz, H-1), 4.68 (1H, ddd, *J* = 49.8, 9.5, 3.5 Hz, H-3), 4.52-4.45 (1H, m, H-2), 4.36 (1H, ddd, *J* = 10.6, 5.0, 2.4 Hz, H-6a), 4.28 (1H, ddd, *J* = 10.9, 9.5, 9.5 Hz, H-4), 3.94 (1H, dd, *J* = 10.3, 10.3 Hz, H-6b), 3.44 (1H, dddd, *J* = 9.9, 9.9, 5.0, 1.6 Hz, H-5), 2.67 (1H, s, O*H*); **^{13}C {^{1}H} NMR** (101 MHz; $CDCl_3$) δ 136.9 (C_q), 133.7 (C_q), 131.8 (CH), 129.3 (CH), 129.2 (CH), 128.4 (CH), 128.1 (CH), 126.2 (CH), 101.9 (*C*HPh), 90.5 (d, $^1J_{C-F}$ = 190.7 Hz, C3), 87.5 (d, $^3J_{C-F}$ = 5.7 Hz, C1), 76.2 (d, $^2J_{C-F}$ = 17.8 Hz, C4), 71.2 (d, $^2J_{C-F}$ = 17.6 Hz, C2), 70.5 (d, $^3J_{C-F}$ = 7.6 Hz, C5), 68.3 (d, $^4J_{C-F}$ = 1.8 Hz, C6); **^{19}F NMR** (376 MHz; $CDCl_3$) δ −197.7 (ddd, *J* = 49.9, 10.8, 6.2 Hz); **HRMS** (ESI) *m/z* found: $(M+H)^+$ 363.1060, $C_{19}H_{20}FO_4S$ requires 363.1066. Data matched those previously reported.[3]

Phenyl 2-*O*-benzyl-4,6-*O*-benzylidene-3-deoxy-3-fluoro-1-thio-β-D-mannopyranoside (1)

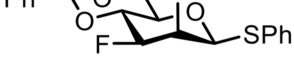

To a stirred solution of compound **S-7** (2.46 g, 6.78 mmol, 1.0 eq.) in anhydrous DMF (24 mL) at 0 °C was added NaH (60% in mineral oil, 298 mg, 7.46 mmol, 1.1 eq.). The mixture was stirred at room temperature for 10 min, after which time benzyl bromide (0.97 mL, 8.14 mmol, 1.2 eq.) was added. The mixture was stirred at room temperature overnight. The mixture was

then concentrated *in vacuo*, dissolved in EtOAc (50 mL) and washed with saturated aqueous $NaHCO_3$ (50 mL). The aqueous layer was extracted with EtOAc (3 × 50 mL). The combined organic extracts were washed with water (50 mL), brine (50 mL), dried ($MgSO_4$) and concentrated *in vacuo*. The crude product was purified by column chromatography on silica gel to afford compound **1** (2.38 g, 5.25 mmol, 77%) as a white solid. R_f = 0.8 (30% EtOAc/n-hexane); m.p. 170-171 °C; **^{1}H NMR** (400 MHz; $CDCl_3$) δ 7.53-7.44 (6H, m, Ar-H), 7.43-7.26 (9H, m, Ar-H), 5.60 (1H, s, C*H*Ph), 5.00 (1H, d, *J* = 11.0 Hz, C*H*H Bn), 4.88 (1H, d, *J* = 1.5 Hz, H-1), 4.80 (1H, d, *J* = 10.8 Hz, C*H*H Bn), 4.76 (1H, ddd, *J* = 49.6, 9.7, 3.5 Hz, H-3), 4.45-4.26 (3H, m, H-2, H-4, H-6a), 3.94 (1H, dd, *J* = 10.3, 10.3 Hz, H-6b), 3.42 (1H, ddd, *J* = 9.5, 9.4, 4.8 Hz, H-5); **^{13}C {^{1}H} NMR** (101 MHz; $CDCl_3$) δ 137.3 (C_q), 137.0 (C_q), 134.4 (C_q), 131.5 (CH), 129.2 (CH), 129.1 (CH), 128.7 (CH), 128.4 (CH), 128.3 (CH), 128.1 (CH), 127.8 (CH), 126.2 (CH), 101.8 (*C*HPh), 91.8 (d, $^1J_{C\text{-}F}$ = 194.6 Hz, C3), 88.4 (d, $^3J_{C\text{-}F}$ = 7.1 Hz, C1), 78.3 (d, $^2J_{C\text{-}F}$ = 15.8 Hz, C2), 76.5 (d, $^2J_{C\text{-}F}$ = 17.7 Hz, C4), 76.1 (d, $^4J_{C\text{-}F}$ = 4.4 Hz, CH_2 Bn), 70.7 (d, $^3J_{C\text{-}F}$ = 7.6 Hz, C5), 68.3 (d, $^4J_{C\text{-}F}$ = 1.8 Hz, C6); **^{19}F NMR** (376 MHz; $CDCl_3$) δ −197.0 (ddd, *J* = 49.5, 7.6, 7.6 Hz); **HRMS** (ESI) *m/z* found: $(M+Na)^+$ 475.1342, $C_{26}H_{25}FO_4SNa$ requires 475.1355. Data matched those previously reported.[3]

Phenyl 2-*O*-benzyl-3-deoxy-3-fluoro-1-thio-β-D-mannopyranoside (2)

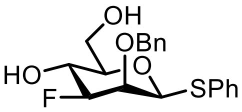

A stirred solution of compound **1** (1.27 g, 2.80 mmol, 1.0 eq.) and *p*-toluenesulfonic acid monohydrate (54 mg, 0.31 mmol, 0.1 eq.) in MeOH (12.5 mL) was heated to reflux for 2 h. The mixture was then cooled to 0 °C, neutralised with NEt_3 and concentrated *in vacuo*. The resulting residue was dissolved in CH_2Cl_2 (100 mL) and washed with water (100 mL). The organic layer was concentrated *in vacuo* and the residue purified by chromatography on silica gel (50% EtOAc/*n*-hexane) to afford compound **2** (904 mg, 2.48 mmol, 89%) as an off-white solid. R_f = 0.1 (40% EtOAc/*n*-hexane); m.p. 98-99 °C; **$[\alpha]_D^{22}$** −66 (c 1.0, $CHCl_3$); **^{1}H NMR** (400 MHz; $CDCl_3$) δ 7.48-7.42 (4H, m, Ar-H), 7.39-7.26 (6H, m, Ar-H), 4.93 (1H, d, *J* = 11.2 Hz, CH*H* Bn), 4.82 (1H, dd, *J* = 1.4, 1.4 Hz, H-1), 4.76 (1H, *J* = 11.2 Hz, CH*H* Bn), 4.52 (1H, ddd, *J* = 48.7, 9.3, 3.3 Hz, H-3), 4.30-4.18 (2H, m, H-2, H-4), 3.97-3.90 (1H, m, H-6a), 3.90-3.81 (1H, m, H-6b), 3.33 (1H, dddd, *J* = 9.7, 4.9, 3.4, 1.2 Hz, H-5), 2.64 (1H, d, *J* = 3.6 Hz, C6-O*H*), 2.29 (1H, dd, *J* = 6.7, 6.7 Hz, C4-O*H*); **^{13}C {^{1}H} NMR** (101 MHz; $CDCl_3$) δ 137.4 (C_q), 134.5 (C_q), 131.0 (CH), 129.1 (CH), 128.5 (CH), 128.3 (CH), 128.0 (CH), 127.6 (CH), 95.8 (d, $^1J_{C\text{-}F}$ = 189.5 Hz, C3), 87.0 (d, $^3J_{C\text{-}F}$ = 8.4 Hz, C1), 79.0 (d, $^3J_{C\text{-}F}$ = 6.6 Hz, C5), 77.7

(d, ${}^2J_{C\text{-}F}$ = 15.5 Hz, C2), 75.5 (d, ${}^4J_{C\text{-}F}$ = 4.1 Hz, CH_2 Bn), 66.7 (d, ${}^2J_{C\text{-}F}$ = 19.0 Hz, C4), 62.4 (d, ${}^4J_{C\text{-}F}$ = 2.0 Hz, C6); **^{19}F NMR** (376 MHz; $CDCl_3$) δ −195.7 (ddd, *J* = 49.0, 11.9, 5.4 Hz); **HRMS** (ESI) *m/z* found: $(M\text{-}H)^-$ 363.1071, $C_{19}H_{20}O_4FS$ requires 363.1072.

Methyl (phenyl 2-*O*-benzyl-3-deoxy-3-fluoro-1-thio-β-D-mannopyranoside) uronate (3)

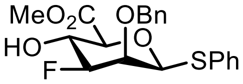

To a vigorously stirred solution of compound **2** (1.51 g, 4.13 mmol, 1.0 eq.) in CH_2Cl_2 (14.0 mL) and water (4.7 mL) at 0 °C was added TEMPO (13 mg, 0.08 mmol, 0.02 eq.), and BAIB (3.25 g, 10.1 mmol, 2.4 eq.). The mixture was allowed to warm to room temperature and was stirred for 3 h. The reaction mixture was quenched by the addition of 10% aqueous sodium thiosulfate and diluted with CH_2Cl_2 (50 mL). The aqueous layer was acidified to ~pH = 2 by the addition of conc. HCl and the organic layer was separated. The aqueous layer was extracted with CH_2Cl_2 (3 × 50 mL) and the combined organic layers were dried ($MgSO_4$) and concentrated *in vacuo* to afford a pale-yellow oil. This oil was then dried under high-vacuum for ~4 h. The oil was dissolved in DMF (5.0 mL) and cooled to 0 °C. K_2CO_3 (573 mg, 4.14 mmol, 1.0 eq.) and iodomethane (0.65 mL, 10.4 mmol, 2.5 eq.) were added and the mixture was stirred overnight while being allowed to warm to room temperature. The reaction mixture was concentrated *in vacuo*. The resulting residue was dissolved in CH_2Cl_2 (200 mL) and washed with water (100 mL) and brine (50 mL). The organic layer was dried ($MgSO_4$) and concentrated *in vacuo*. The resulting residue was purified by chromatography on silica gel (30% EtOAc/*n*-hexane) to afford the title compound **3** (998 mg, 2.55 mmol, 62%) as a white solid. R_f = 0.2 (30% EtOAc/*n*-hexane); m.p. 103-104 °C; **[α]**$_D^{23}$ −115 (c 1.0, $CHCl_3$); **^{1}H NMR** (400 MHz; $CDCl_3$) δ 7.44-7.43 (4H, m, Ar-H), 7.39-7.26 (6H. m, Ar-H), 4.96 (1H, d, *J* = 11.3 Hz, C*H*H Bn), 4.79 (1H, d, *J* = 11.2 Hz, C*H*H Bn), 4.79 (1H, dd, *J* = 1.4, 1.4 Hz, H-1), 4.46-4.42 (2H, m, H-3, H-4), 4.24 (1H, ddd, *J* = 5.4, 3.1, 1.1 Hz, H-2), 3.83 (3H, s, COOCH_3), 3.78 (1H, dd, *J* = 9.5, 1.3 Hz, H-5), 3.27 (1H, d, *J* = 2.4 Hz, O*H*); **^{13}C {^{1}H} NMR** (101 MHz; $CDCl_3$) δ 169.4 (d, ${}^4J_{C\text{-}F}$ = 3.3 Hz, C=O), 137.3 (C_q), 134.4 (C_q), 131.5 (CH), 129.1 (CH), 128.6 (CH), 128.3 (CH), 123.0 (CH), 127.8 (CH), 94.3 (d, ${}^1J_{C\text{-}F}$ = 192.1 Hz, C3), 88.0 (d, ${}^3J_{C\text{-}F}$ = 8.5 Hz, C1), 77.2-76.9 (m, C2, C5), 75.5 (d, ${}^4J_{C\text{-}F}$ = 4.2 Hz, CH_2 Bn), 67.2 (d, ${}^2J_{C\text{-}F}$ = 19.4 Hz, C4), 52.9 (CH_3); **^{19}F NMR** (376 MHz; $CDCl_3$) δ −195.64 (ddd, *J* = 46.8, 12.9, 5.6 Hz); **HRMS** (ESI) *m/z* found: $(M+Na)^+$ 415.0973, $C_{20}H_{21}O_5FS$ requires 415.0991.

Methyl (phenyl 4-*O*-levulinoyl-2-*O*-benzyl-3-deoxy-3-fluoro-1-thio-β-D-mannopyranoside) uronate (4)

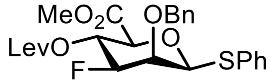

To a stirred solution of compound **3** (470 mg, 0.98 mmol, 1.0 eq.) in CH_2Cl_2 (2.8 mL) was added EDC·HCl (360 mg, 1.88 mmol, 1.9 eq.), levulinic acid (217 mg, 1.87 mmol, 1.9 eq.), DIPEA (0.33 mL, 1.89 mmol, 1.9 eq.) and DMAP (230 mg, 1.88 mmol, 1.9 eq.). The mixture was stirred at room temperature overnight and diluted with CH_2Cl_2 (25 mL). The organic layer was washed with water (2 × 10 mL) and concentrated *in vacuo*. The resulting residue was purified by chromatography on silica gel (40% EtOAc/*n*-hexane) to afford the title compound **4** as a white solid (487 mg, 0.842 mmol, 86%). R_f = 0.3 (40% EtOAc/*n*-hexane); m.p. 111-112 °C; **[α]**$_D^{23}$ −99 (c 1.0, $CHCl_3$); **^{1}H NMR** (400 MHz; $CDCl_3$) δ 7.50-7.43 (4H, m, Ar-H), 7.40-7.26 (6H, m, Ar-H), 5.70 (1H, ddd, *J* = 10.4, 9.7, 9.7 Hz, H-4), 4.98 (1H, d, *J* = 11.4 Hz, C*H*H Bn), 4.78 (1H, dd, *J* = 1.5, 1.5 Hz, H-1), 4.78 (1H, d, *J* = 11.5 Hz, C*H*H Bn), 4.64 (1H, ddd, *J* = 48.3, 9.5, 3.2 Hz, H-3), 4.27 (1H, ddd, *J* = 6.0, 3.3, 1.2 Hz, H-2), 3.90 (1H, dd, *J* = 9.8, 1.2 Hz, H-5), 3.76 (3H, s, COOC*H*$_3$), 2.79-2.72 (2H, m, CH_2 Lev), 2.64-2.57 (2H, m, CH_2 Lev), 2.18 (3H, s, CH_3 Lev); **^{13}C {^{1}H} NMR** (101 MHz; $CDCl_3$) δ 206.1 (C=O COMe Lev), 171.4 (C=O Lev), 166.9 (d, *J* = 3.1 Hz, *C*OOMe), 137.1 (C_q), 134.3 (C_q), 131.6 (CH), 129.1 (CH), 128.7 (CH), 128.3 (CH), 128.0 (CH), 127.9 (CH), 92.4 (d, *J* = 195.3 Hz, C3), 87.8 (d, *J* = 8.3 Hz, C1), 76.7-76.6 (m, C2), 76.1 (d, *J* = 7.0 Hz, C5), 75.3 (d, *J* = 4.3 Hz, CH_2 Bn), 67.7 (d, *J* = 19.8 Hz, C4), 52.9 (COO*C*H$_3$), 37.8 (CH_2 Lev), 29.8 (CH_3 Lev), 27.8 (CH_2 Lev); **^{19}F NMR** (376 MHz; $CDCl_3$) δ −194.3 (ddd, *J* = 48.5, 10.5, 6.0 Hz); **HRMS** (ESI) *m/z* found: $(M+NH_4)^+$ 508.1805, $C_{25}H_{27}O_7FS$ requires 508.1800.

Methyl (phenyl 4-*O*-acetyl-2-*O*-benzyl-3-deoxy-3-fluoro-1-thio-β-D-mannopyranoside) uronate (7)

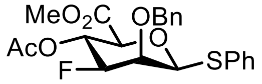

A solution of compound **3** (264 mg, 0.67 mmol, 1.0 eq.), Ac_2O (0.13 mL, 1.35 mmol, 2.0 eq.), DMAP (9 mg, 0.07 mmol, 0.1 eq.) and pyridine (0.11 mL, 1.35 mmol, 2.0 eq.) in CH_2Cl_2 (3.4 mL) was stirred at room temperature for 30 min. The reaction was quenched with saturated aqueous $NaHCO_3$ and diluted with CH_2Cl_2 (50 mL) and water (25 mL). The organic layer was separated and the aqueous layer extracted with CH_2Cl_2 (10 mL). The combined organic layers

were dried ($MgSO_4$) and concentrated *in vacuo*. The crude product was purified by chromatography on silica gel (25% EtOAc/*n*-hexane) to afford the title compound **7** (233 mg, 0.54 mmol, 80%) as a white solid. R_f = 0.2 (25% EtOAc/*n*-hexane); m.p. 167-168 °C; $[\alpha]_D^{19}$ −125 (c 1.0, $CHCl_3$); **^{1}H NMR** (400 MHz; $CDCl_3$) δ 7.49-7.45 (4H, m, Ar-H), 7.40-7.26 (6H, m, Ar-H), 5.69 (1H, ddd, *J* = 10.0, 10.0, 9.6 Hz, H-4), 4.98 (1H, d, *J* = 11.4 Hz, C*H*H Bn), 4.79 (1H, d, 10.9 Hz, C*H*H Bn), 4.78-4.77 (1H, m, H-1), 4.63 (1H, ddd, *J* = 48.3, 9.6, 3.3 Hz, H-3), 4.27 (1H, ddd, *J* = 5.8, 3.2, 1.0 Hz, H-2), 3.88 (1H, dd, *J* = 9.8, 1.2 Hz, H-5), 3.76 (3H, s, $COOCH_3$), 2.09 (3H, s, CH_3 Ac); **^{13}C {^{1}H} NMR** (101 MHz; $CDCl_3$) δ 169.5 (C=O Ac), 167.0 (d, *J* = 3.1 Hz, *C*OOMe), 137.1 (C_q), 134.3 (C_q), 131.6 (CH), 129.1 (CH), 128.7 (CH), 128.3 (CH), 128.0 (CH), 127.9 (CH), 92.3 (d, $^1J_{C\text{-}F}$ = 195.2 Hz, C-3), 87.8 (d, $^3J_{C\text{-}F}$ = 8.3 Hz, C-1), 76.7 (d, $^2J_{C\text{-}F}$ = 15.7 Hz, C-2), 76.2 (d, $^3J_{C\text{-}F}$ = 7.0 Hz, C-5), 75.4 (d, $^4J_{C\text{-}F}$ = 4.2 Hz, CH_2 Bn), 67.5 (d, $^2J_{C\text{-}F}$ = 19.7 Hz, C-4), 52.8 (COO*C*H_3), 20.7 (CH_3 Ac); **^{19}F NMR**: (376 MHz; $CDCl_3$) δ: −194.3 (ddd, *J* = 48.3, 10.4, 5.9 Hz); **^{13}C-GATED** (101 MHz, $CDCl_3$) 87.8 ($^1J_{C1\text{-}H1}$ = 154 Hz, C1); **HRMS** (ESI) *m/z* found: $(M+NH_4)^+$ 452.1538, $C_{22}H_{27}FNO_6S$ requires 452.1543.

1.3 Synthesis of Native ManA Thioglycoside 6

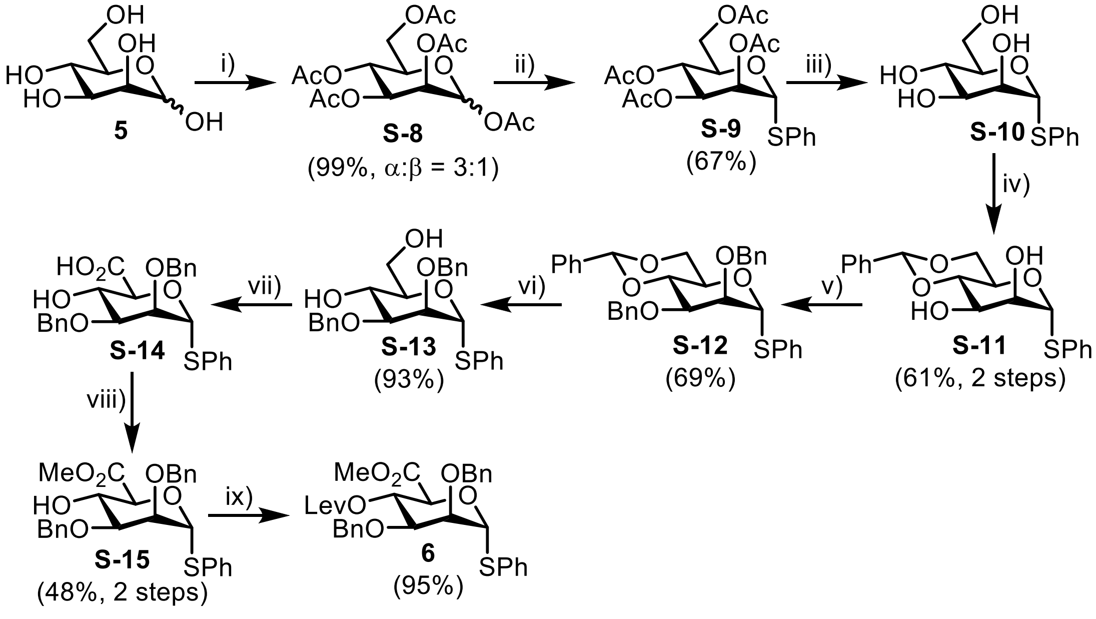

***Reaction conditions: i)** Ac_2O, NaOAc; **ii)** PhSH, $BF_3{\cdot}Et_2O$, CH_2Cl_2; **iii)** Na_2CO_3, MeOH; **iv)** $PhCH(OMe)_2$, $HBF_4{\cdot}OEt_2$, DMF; **v)** BnBr, NaH, DMF; **vi)** p-TsOH·H_2O, MeOH; **vii)** TEMPO, BAIB, H_2O, CH_2Cl_2; **viii)** MeI, K_2CO_3, DMF; **ix)** LevOH, EDC·HCl, DIPEA, DMAP, CH_2Cl_2.*

1,2,3,4,6-Penta-*O*-acetyl-α/β-D-mannopyranoside (S-8)

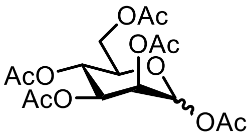

To a stirred mixture of α/β-D-mannose (**5**) (20 g, 111 mmol, 1 eq.) in acetic anhydride (105 mL) at 0 °C was added NaOAc (4.55 g, 55.5 mmol, 0.5 eq.). The mixture was heated to reflux for 10 min. and was then cooled to 0 °C. Water (500 mL) was added and the mixture was stirred for 1 h. The mixture was diluted with CH_2Cl_2 (500 mL). The organic layer was separated and the aqueous layer extracted with CH_2Cl_2 (250 mL). The combined organic layers were concentrated *in vacuo* to afford a red oil. A small sample was purified by chromatography on silica gel (3% Et_2O/CH_2Cl_2) to obtain solely the α-anomer for analysis. The remaining oil was filtered through a plug of silica, eluting with Et_2O to afford the title compound **S-8** as a pale yellow oil (42.9 g, 110 mmol, 99%, α:β = 3:1). R_f = 0.4 (40% EtOAc/*n*-hexane); **α-anomer:** ^{1}**H NMR** (400 MHz; $CDCl_3$) δ 6.09 (1H, d, *J* = 1.8 Hz, H-1), 5.37-5.33 (2H, m, H-3, H-4), 5.26 (1H, ddd, *J* = 2.5, 2.0, 0.7 Hz, H-2), 4.29 (1H, dd, *J* = 12.4, 4.9 Hz, H-6a), 4.11 (1H, dd, *J* = 12.4, 2.5 Hz, H-6b), 4.08-4.03 (1H, m, H-5), 2.18 (3H, s, CH_3 Ac), 2.18 (3H, s, CH_3 Ac), 2.10 (3H, s, CH_3 Ac), 2.06 (3H, s, CH_3 Ac), 2.01 (3H, s, CH_3 Ac); ^{13}**C {^{1}H} NMR** (101 MHz; $CDCl_3$) δ 170.7 (C=O), 170.0 (C=O), 169.8 (C=O), 169.6 (C=O), 168.1 (C=O), 90.6 (C1), 70.6 (C5), 68.8 (C3), 68.4 (C2), 65.6 (C4), 62.1 (C6), 20.9 (CH_3 OAc), 20.8 (CH_3 OAc), 20.7 (CH_3 OAc), 20.7 (CH_3 OAc), 20.7 (CH_3 OAc); **HRMS** (ESI) *m/z* found: $(M+Na)^+$ 413.1059, $C_{16}H_{22}NaO_{11}$ requires 413.1060. Data matched those previously reported.[4]

Phenyl 2,3,4,6-tetra-*O*-acetyl-1-thio-α-D-mannopyranoside (S-9)

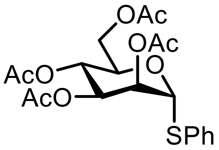

To a stirred solution of compound **S-8** (108 g, 275 mmol, 1.0 eq.) in anhydrous CH_2Cl_2 (42.2 mL) at 0 °C was slowly added $BF_3{\cdot}OEt_2$ (163 mL, 1.32 mol, 4.8 eq.) and thiophenol (42.2 mL, 413 mmol, 1.5 eq.). The mixture was stirred at 0 °C for 48 h after which time the reaction was quenched by the addition of saturated aqueous $NaHCO_3$. The organic layer was separated and sequentially washed with saturated aqueous $NaHCO_3$ (4 × 160 mL) and 2.0 M aqueous NaOH (5 × 140 mL) and brine (3 × 100 mL). The organic layer was dried ($MgSO_4$) and concentrated *in vacuo* to afford an orange solid. Recrystallisation of the crude product from EtOH (~300 mL) afforded the title compound **S-9** as a white solid (81.9 g, 184 mmol, 67%). R_f = 0.5 (40%

EtOAc/*n*-hexane); **^{1}H NMR** (400 MHz; $CDCl_3$) δ 7.56-7.46 (2H, m, Ar-H), 7.37-7.28 (3H, m, Ar-H), 5.61-5.38 (2H, m, H-1, H-3), 5.40-5.27 (2H, m, H-2, H-4), 4.55 (1H, ddt, *J* = 8.2, 5.8, 2.4 Hz, H-5), 4.31 (1H, dd, *J* = 12.3, 5.9 Hz, H-6a), 4.11 (1H, dd, *J* = 12.2, 2.4 Hz, H-6b), 2.15 (3H, s, CH_3 Ac), 2.08 (3H, s, CH_3 Ac), 2.05 (3H, s, CH_3 Ac), 2.02 (3H, s, CH_3 Ac); **^{13}C {^{1}H} NMR** (101 MHz; $CDCl_3$) δ 170.5 (C=O Ac), 169.9 (C=O Ac), 169.8 (C=O Ac), 169.7 (C=O Ac), 132.6 (C_q), 132.1 (CH), 129.2 (CH), 128.1 (CH), 85.7 (C1), 70.9 (C3), 69.5 (C5), 69.4 (C2), 66.4 (C4), 62.5 (C6), 20.9 (CH_3 Ac), 20.7 (CH_3 Ac), 20.7 (CH_3 Ac), 20.6 (CH_3 Ac); **HRMS** (ESI) *m/z* found: $(M+Na)^+$ 463.1038, $C_{20}H_{24}NaO_9S$ requires 463.1039. Data matched those previously reported.[5]

Phenyl 4,6-*O*-benzylidene-1-thio-α-D-mannopyranoside (S-11)

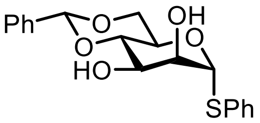

A solution of compound **S-9** (81.9 g, 185 mmol, 1.0 eq.) and sodium carbonate (1.97 g, 18.5 mmol, 0.1 eq.) in MeOH (466 mL) was stirred at room temperature overnight. The reaction mixture was neutralised by the addition of Amberlite IR120 (H^+) ion exchange resin. The mixture was filtered and concentrated *in vacuo* to afford the deacetylated product **S-10**. Compound **S-10** was dissolved in DMF (568 mL) and benzaldehyde dimethyl acetal (27.7 mL, 185 mmol, 1 eq.) added. The mixture was cooled to 0 °C and $HBF_4{\cdot}OEt_2$ (25.4 mL, 206 mmol, 1.1 eq.) was added. The mixture was then allowed to warm to room temperature and was stirred overnight. The reaction was quenched by the addition of NEt_3 and the mixture concentrated *in vacuo*. The resulting solid was recrystallised from hot EtOH to afford the title compound **S-11** (41.3 g, 115 mmol, 61%) as a white solid. R_f = 0.2 (40% EtOAc/*n*-hexane); m.p. 98-99 °C; **^{1}H NMR** (400 MHz; $CDCl_3$) δ 7.51-7.44 (4H, m, Ar-H), 7.43-7.28 (6H, m, Ar-H), 5.63 (1H, s, C*H*Ph), 5.61-5.53 (1H, m, O*H*), 5.47 (1H, d, *J* = 1.3 Hz, H-1), 5.30-5.19 (1H, m, O*H*), 4.11-4.04 (2H, m, H-5, H-6a), 4.03-3.99 (1H, m, H-2), 3.96 (1H, dd, *J* = 9.3, 9.3 Hz, H-4), 3.82-3.72 (2H, m, H-3, H-6b); **^{13}C {^{1}H} NMR** (101 MHz; $CDCl_3$) δ 138.3 (C_q), 134.1 (C_q), 131.8 (CH), 129.7 (CH), 129.3 (CH), 128.5 (CH), 127.9 (CH), 126.9 (CH), 101.7 (*C*HPh), 89.7 (C1), 78.9 (C4), 72.9 (C2), 68.5 (C3), 68.1 (C6), 65.8 (C5); **HRMS** (ESI) *m/z* found: $(M+Na)^+$ 383.0921, $C_{19}H_{20}NaO_5S$ requires 383.0929. Data matched those previously reported.[5]

Phenyl 2,3-di-*O*-benzyl-4,6-*O*-benzylidene-1-thio-α-D-mannopyranoside (S-12)

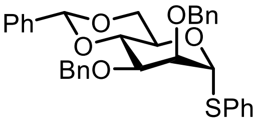

A stirred solution of compound **S-11** (41.3 g, 115 mmol, 1.0 eq.) in DMF (500 mL) was cooled to 0 °C and sodium hydride (60% dispersion in mineral oil, 12.3 g, 309 mmol, 2.7 eq.) was added. After 15 min benzyl bromide (36.6 mL, 307 mmol, 2.7 eq.) was slowly added and the mixture was stirred overnight while being allowed to warm to room temperature. The mixture was then cooled to 0 °C and quenched with MeOH. The reaction mixture was concentrated *in vacuo*, dissolved in CH_2Cl_2 (500 mL) and washed with water (500 mL). The aqueous layer was extracted with CH_2Cl_2 (250 mL) and the combined organic layers were concentrated *in vacuo* to afford a yellow solid. The solid was recrystallised from hot EtOH (15 mL) to afford the title compound **S-12** as a white solid (~6 g) which was filtered and kept aside. The filtrate was concentrated *in vacuo* and purified by chromatography on silica gel (5% EtOAc/*n*-hexane) afforded the title compound **S-12**. Both batches of product **S-12** were combined to afford the title compound **S-12** (42.9 g, 79.4 mmol, 69%) as a white solid. R_f = 0.6 (30% EtOAc/*n*-hexane); m.p. 82-83 °C; **^{1}H NMR** (400 MHz; $CDCl_3$) δ 7.55-7.48 (2H, m, Ar-H), 7.42-7.26 (18H, m, Ar-H), 5.65 (1H, s, C*H*Ph), 5.51 (1H, d, *J* = 1.5 Hz, H-1), 4.83 (1H, d, *J* = 12.2 Hz, C*H*H Bn), 4.72 (2H, s, CH_2 Bn), 4.65 (1H, d, *J* = 12.2 Hz, C*H*H Bn), 4.37-4.18 (3H, m, H-4, H-5, H-6a), 4.04 (1H, dd, *J* = 3.2, 1.5 Hz, H-2), 3.97 (1H, dd, *J* = 9.5, 3.2 Hz, H-3), 3.89 (1H, dd, *J* = 9.8, 9.8 Hz, H-6b); **^{13}C {^{1}H} NMR** (101 MHz; $CDCl_3$) δ 138.4 (C_q), 137.7 (C_q), 137.6 (C_q), 133.8 (C_q), 131.6 (CH), 129.1 (CH), 128.9 (CH), 128.4 (CH),128.4 (CH), 128.2 (CH), 128.1 (CH), 127.9 (CH), 127.7 (CH), 127.6 (CH), 126.1 (CH), 101.5 (*C*HPh), 87.1 (C1), 79.1 (C4), 78.1 (C2), 76.2 (C3), 73.1 (*C*H_2 Bn), 73.1 (*C*H_2 Bn), 68.5 (C6), 65.5 (C5); **HRMS** (ESI) *m/z* found: $(M+Na)^+$ 563.1871, $C_{33}H_{32}NaO_5S$ requires 563.1868. Data matched those previously reported.[6]

Phenyl 2,3-di-*O*-benzyl-1-thio-α-D-mannopyranoside (S-13)

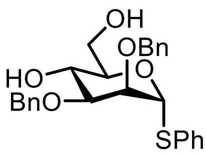

A stirred solution of compound **S-12** (13.5 g, 24.9 mmol, 1 eq.) and *p*-toluenesulfonic acid monohydrate (948 mg, 4.98 mmol, 0.2 eq.) in MeOH (101 mL) was heated to reflux for 2 h. The mixture was then cooled to 0 °C, neutralised with NEt_3 and concentrated *in vacuo*. The

resulting residue was dissolved in CH_2Cl_2 (500 mL) and washed with water (200 mL). The organic layer was concentrated *in vacuo* and the resulting residue purified by chromatography on silica gel (40% EtOAc/*n*-hexane) to afford the title compound **S-13** (10.5 g, 23.1 mmol, 93%) as a white solid. $R_f = 0.25$ (40% EtOAc/*n*-hexane); m.p. 95-96 °C; **^{1}H NMR** (400 MHz; $CDCl_3$) δ 7.42-7.37 (2H, m, Ar-H), 7.34-7.23 (13H, m, Ar-H), 5.53 (1H, d, *J* = 1.5 Hz, H-1), 4.63 (1H, d, *J* = 12.2 Hz, C*H*H Bn), 4.57-4.45 (3H, m, C*H*H Bn, CH_2 Bn), 4.17-4.02 (2H, m, H-4, H-5), 3.96 (1H, dd, *J* = 3.1, 1.6 Hz, H-2), 3.86-3.77 (2H, m, H-6a, H-6b), 3.67 (1H, dd, *J* = 8.9, 3.0 Hz, H-3), 3.17 (1H, d, *J* = 3.0 Hz, OH), 2.54-2.46 (1H, m, OH); **^{13}C {^{1}H} NMR** (101 MHz; $CDCl_3$) δ 137.7 (C_q), 137.6 (C_q), 133.9 (C_q), 131.7 (CH), 129.1 (CH), 128.5 (CH), 128.4 (CH), 128.0 (CH), 127.9 (CH), 127.9 (CH), 127.8 (CH), 127.6 (CH), 85.9 (C1), 79.5 (C3), 75.6 (C2), 73.4 (C4), 72.1 (CH_2 Bn), 71.8 (CH_2 Bn), 67.0 (C4), 62.3 (C6); **HRMS** (ESI) *m/z* found: $(M+Na)^+$ 475.1555, $C_{26}H_{28}NaO_5S$ requires 475.1555. Data matched those previously reported.[7]

Methyl (phenyl-2,3-di-*O*-benzyl-1-thio-α-D-mannopyranoside) uronate (S-15)

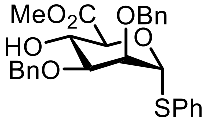

To a vigorously stirred solution of compound **S-13** (2.10 g, 4.64 mmol, 1 eq.) in CH_2Cl_2 (14.7 mL) and water (5.5 mL) at 0 °C was added TEMPO (14 mg, 0.09 mmol, 0.02 eq.), and BAIB (3.71 g, 11.5 mmol, 2.5 eq.). The mixture was allowed to warm to room temperature and was stirred for 1.5 h. The reaction mixture was quenched by the addition of 10% aqueous sodium thiosulfate and diluted with CH_2Cl_2 (50 mL). The aqueous layer was acidified to ~pH = 1 by the addition of conc. HCl and the organic layer was separated. The aqueous layer was extracted with CH_2Cl_2 (3 × 50 mL) and the combined organic layers were dried ($MgSO_4$) and concentrated *in vacuo* to afford an orange oil. This oil was then dried under high vacuum for ~4 h. K_2CO_3 (0.64 g, 4.63 mmol, 1 eq.) and iodomethane (0.71 mL, 11.4 mmol, 2.5 eq.) were added and the mixture was stirred overnight. The reaction mixture was concentrated *in vacuo*. The resulting residue was dissolved in CH_2Cl_2 (200 mL) and washed with water (100 mL) and brine (50 mL). The organic layer was dried ($MgSO_4$) and concentrated *in vacuo*. The crude product was purified by chromatography on silica gel (20%→30% EtOAc/*n*-hexane) to afford the title compound **S-15** as a colourless oil (1.06 g, 2.21 mmol, 48%). $R_f = 0.3$ (30% EtOAc/*n*-hexane); **^{1}H NMR** (400 MHz; $CDCl_3$) δ 7.47-7.42 (2H, m, Ar-H), 7.36-7.26 (13H, m, Ar-H), 5.61 (1H, d, *J* = 2.3 Hz, H-1), 4.74-4.53 (5H, m, 2 × CH_2 Bn, H-5), 4.38 (1H, ddd, *J* = 9.0, 9.0, 2.3 Hz, H-4), 3.95 (1H, dd, *J* = 2.7, 2.7 Hz, H-2), 3.78 (3H, s, COOC*H*$_3$), 3.71 (1H, dd, *J* = 9.0,

3.0 Hz, H-3), 2.96 (1H, d, *J* = 2.7 Hz, OH); **^{13}C {^{1}H} NMR** (101 MHz; $CDCl_3$) δ 169.3 (C=O), 136.9 (C_q), 136.6 (C_q), 132.7 (C_q), 130.5 (CH), 128.1 (CH), 127.5 (CH), 127.4 (CH), 127.0 (CH), 126.8 (CH), 126.8 (CH), 126.7 (CH), 85.2 (C1), 77.2 (C3), 74.6 (C2), 71.6 (C5), 71.5 (CH_2 Bn), 71.3 (CH_2 Bn), 67.5 (C4), 51.6 (CH_3); **HRMS** (ESI) *m/z* found: $(M+NH_4)^+$ 498.1931, $C_{27}H_{32}NO_6S$ requires 498.1950. Data matched those previously reported.[8]

Methyl (phenyl-4-*O*-levulinoyl-2,3-di-*O*-benzyl-1-thio-α-D-mannopyranoside) uronate (6)

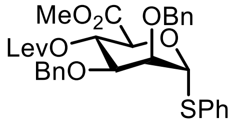

To a stirred solution of compound **S-15** (986 mg, 2.05 mmol, 1 eq.) in CH_2Cl_2 (5 mL) was added EDC·HCl (629 mg, 3.28 mmol, 1.6 eq.), levulinic acid (381 mg, 3.28 mmol, 1.6 eq.), DIPEA (0.572 mL, 3.28 mmol, 1.6 eq.) and DMAP (401 mg, 3.28 mmol, 1.6 eq.). The mixture was stirred at room temperature overnight and was then diluted with EtOAc (50 mL). The organic layer was washed with water (2 × 25 mL) and was concentrated *in vacuo*. The resulting residue was purified by chromatography on silica gel (30%→35% EtOAc/*n*-hexane) to afford the title compound **6** as a pale-yellow oil (1.12 g, 1.94 mmol, 95%). R_f = 0.4 (40% EtOAc/*n*-hexane); **^{1}H NMR** (400 MHz; $CDCl_3$) δ 7.59 (2H, d, *J* = 7.6 Hz, Ar-H), 7.33-7.21 (13H, m, Ar-H), 5.78 (1H, d, *J* = 7.2 Hz, H-1), 5.57 (1H, dd, *J* = 6.0, 4.5 Hz, H-4), 4.64 (1H, d, *J* = 11.9 Hz, C*H*H Bn), 4.61-4.50 (4H, m, 3 × C*H*H Bn, H-5), 3.86 (1H, dd, *J* = 6.0, 2.8 Hz, H-3), 3.75 (1H, dd, *J* = 7.6 Hz, 2.9 Hz, H-2), 3.59 (3H, s, COOC*H*$_3$), 2.79-2.66 (2H, m, CH_2 Lev), 2.62-2.47 (2H, m, CH_2 Lev); **^{13}C {^{1}H} NMR** (101 MHz; $CDCl_3$) δ 206.2 (*C*OMe Lev), 171.6 (OC=O Lev), 168.5 (*C*OOMe), 137.7 (C_q), 137.5 (C_q), 133.7 (C_q), 131.0 (CH), 128.9 (CH), 128.4 (CH), 128.1 (CH), 127.9 (CH), 127.8 (CH), 127.8 (CH), 127.2 (CH), 82.8 (br., C1), 74.2 (br., C2), 73.7 (br., C3), 72.8 (br., C5), 72.6 (CH_2 Bn), 72.4 (CH_2 Bn), 69.8 (C4), 52.4 (COO*C*H$_3$), 37.8 (CH_2 Lev), 29.9 (CH_3 Lev), 27.9 (CH_2 Lev); **HRMS** (ESI) *m/z* found: $(M+Na)^+$ 601.1862, $C_{32}H_{34}NaO_8S$ requires 601.1872; Data matched those previously reported.[8]

1.4 Synthesis of NPTFA Imidate Donors S-17 and S-19

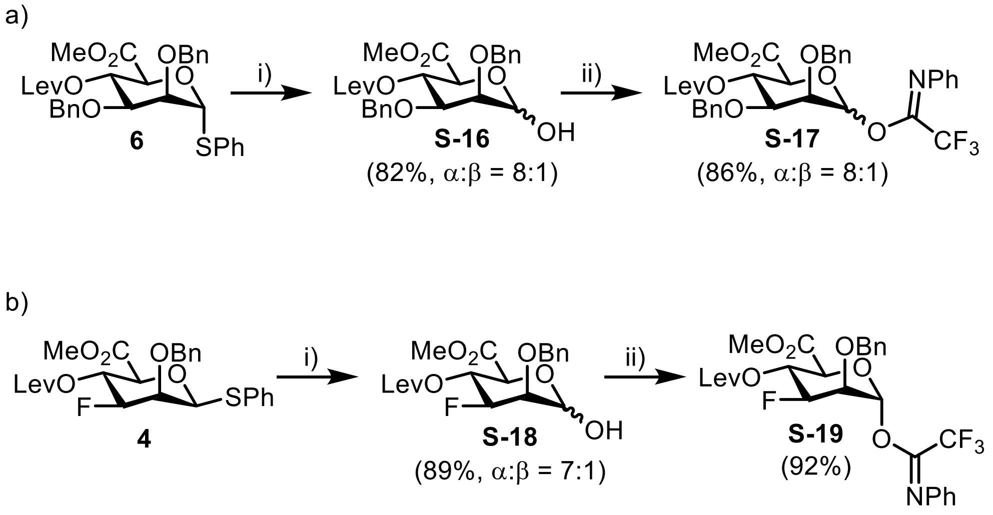

Reaction conditions: i) *NIS, TFA, CH_2Cl_2:MeOH (1:1);* ***ii)*** *2,2,2-Trifluoro-N-phenylacetimidoyl chloride, K_2CO_3, acetone:H_2O (20:1).*

Methyl (4-*O*-levulinoyl-2,3-di-*O*-benzyl-α/β-D-mannopyranoside) uronate (S-16)

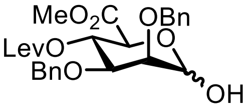

To a stirred solution of thioglycoside **6** (420 mg, 0.73 mmol, 1.0 eq.) and NIS (163 mg, 0.73 mmol, 1.0 eq.) in 10:1 CH_2Cl_2:H_2O (8.11 mL) at 0 °C was slowly added trifluoroacetic acid (0.54 mL, 0.70 mmol, 1.0 eq.). The mixture was stirred vigorously for 2.5 h and then saturated aqueous sodium thiosulfate (14 mL) was added. The mixture was stirred for a further 30 min and was diluted with EtOAc (100 mL). The organic layer was separated and the aqueous layer extracted with EtOAc (50 mL). The combined organic layers were washed with saturated aqueous $NaHCO_3$ (50 mL), dried ($MgSO_4$) and concentrated *in vacuo*. Purification by chromatography on silica gel (60% EtOAc/pet. ether) afforded the title compound **S-16** (290 mg, 0.60 mmol, 82%, α:β = 8:1) as a colourless oil. R_f = 0.3 (60% EtOAc/pet. ether); **α anomer: ^{1}H NMR** (400 MHz; $CDCl_3$) δ 7.37-7.23 (10H, m, Ar-H), 5.57-5.50 (2H, m, H-1, H-4), 4.75 (1H, d, *J* = 12.2 Hz, C*H*H Bn), 4.67 (1H, d, *J* = 12.1 Hz, C*H*H Bn), 4.61 (1H, d, *J* = 12.0 Hz, C*H*H Bn), 4.57 (1H, d, *J* = 12.0 Hz, C*H*H Bn), 4.46 (1H, d, *J* = 5.0 Hz, H-5), 3.93 (1H, dd, *J* = 6.3, 2.9 Hz, H-3), 3.63 (1H, dd, *J* = 5.6, 2.9 Hz, H-2), 3.60 (3H, s, $COOCH_3$), 2.75-2.68 (2H, m, CH_2 Lev), 2.58-2.51 (2H, m, CH_2 Lev), 2.18 (3H, s, CH_3 Lev); **^{13}C {^{1}H} NMR** (101 MHz; $CDCl_3$) δ 206.3 (*C*OMe Lev), 171.7 (OC=O Lev), 169.0 (*C*OOMe), 138.2 (C_q), 137.8 (C_q), 128.3 (CH), 128.3 (CH), 127.9 (CH), 127.7 (CH), 127.7 (CH), 127.7 (CH), 92.6 (C1), 75.4 (C2), 75.0 (C3), 72.9 (CH_2 Bn), 72.6 (CH_2 Bn), 72.1 (C5), 69.7 (C4), 52.4

(COO*C*H_3), 37.8 (CH_2 Lev), 29.8 (CH_3 Lev), 28.0 (CH_2 Lev); **HRMS** (ESI) *m/z* found: $(M+NH_4)^+$ 504.2233, $C_{26}H_{34}NO_9$ requires 504.2234. Data matched those previously reported.[9]

Methyl (4-*O*-levulinoyl-2,3-di-*O*-benzyl-1-*O*-(*N*-[phenyl]trifluoroacetimidoyl)-α/β-D-mannopyranoside) uronate (S-17)

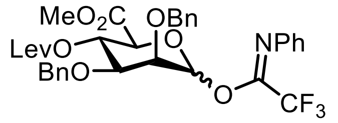

To a stirred suspension of hemiacetal **S-16** (525 mg, 1.08 mmol, 1.0 eq.) and K_2CO_3 (180 mg, 1.30 mmol, 1.2 eq.) in 20:1 acetone:water (11.4 mL) at 0 °C was slowly added 2,2,2-trifluoro-*N*-phenylacetimidoyl chloride (0.26 mL, 1.62 mmol, 1.5 eq.). The mixture was allowed to warm to room temperature and was stirred overnight. The reaction mixture was then concentrated *in vacuo* and redissolved in EtOAc (200 mL) and water (50 mL). The organic layer was separated, dried ($MgSO_4$) and concentrated *in vacuo*. Purification by chromatography on neutralised silica gel (30% EtOAc/*n*-hexane + 0.1% NEt_3) afforded the title compound **S-17** (522 mg, 0.79 mmol, 74%, α:β = 8:1) as a colourless oil. R_f = 0.3 (33% EtOAc/pet. ether); **α anomer: ^{1}H NMR** (400 MHz; $CDCl_3$) δ 7.40-7.21 (12H, m, Ar-H), 7.14-7.09 (1H, m, Ar-H NPh), 6.78 (2H, d, *J* = 7.4 Hz, Ar-H NPh), 6.44 (1H, br. s, H-1), 5.59 (1H, dd, *J* = 7.4, 7.4 Hz, H-4), 4.83-4.50 (4H, m, 2 × CH_2 Bn), 4.39 (1H, d, *J* = 7.1 Hz, H-5), 3.90 (1H, dd, *J* = 7.7, 3.0 Hz, H-3), 3.80-3.77 (1H, m, H-2), 3.67 (3H, s, $COOCH_3$), 2.76-2.69 (2H, m, CH_2 Lev), 2.62-2.53 (2H, m, CH_2 Lev), 2.18 (3H, s, CH_3 Lev); **^{13}C {^{1}H} NMR** (101 MHz; $CDCl_3$) δ 206.2 (*C*OMe Lev), 171.6 (OC=O Lev), 143.2 (C_q NPh), 142.3 (d, $^2J_{C-F}$ = 36.2 Hz, *C*=NPh), 137.4 (C_q), 128.8 (CH), 128.4 (CH), 128.4 (CH), 128.1 (CH), 128.0 (CH), 128.0 (CH), 127.9 (CH), 124.5 (CH NPh), 119.5 (CH NPh), 115.9 (d, $^1J_{C-F}$ = 286.3 Hz, CF_3), 94.3 (C1), 74.7 (C3), 73.1 (C2), 72.9 (CH_2 Bn), 72.9 (CH_2 Bn), 72.9 (CH_2 Bn), 72.9 (C5), 68.9 (C4), 52.7 (COO*C*H_3), 37.8 (CH_2 Lev), 29.8 (CH_3 Lev), 27.9 (CH_2 Lev); **^{19}F NMR** (376 MHz; $CDCl_3$) δ -71.9 (s); **HRMS** (ESI) *m/z* found: $(M+Na)^+$ 680.2069, $C_{34}H_{34}F_3NNaO_9$ requires 680.2083.

Methyl (4-*O*-levulinoyl-2-*O*-benzyl-3-deoxy-3-fluoro-α/β-D-mannopyranoside) uronate (S-18)

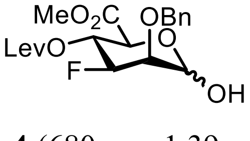

To a stirred solution of thioglycoside **4** (680 mg, 1.39 mmol, 1.0 eq.) and NIS (303 mg, 1.35 mmol, 1.0 eq.) in 10:1 CH_2Cl_2:H_2O (15.5 mL) was slowly added trifluoroacetic acid (1.24 mL,

1.62 mmol, 1.2 eq.). The mixture was stirred vigorously for 2.5 h and then saturated aqueous sodium thiosulfate (25 mL) and saturated aqueous $NaHCO_3$ (25 mL) were added. The mixture was stirred for a further 30 min and was then diluted with CH_2Cl_2 (100 mL). The organic layer was separated and the aqueous layer was extracted with CH_2Cl_2 (50 mL). The combined organic extracts were dried ($MgSO_4$) and concentrated *in vacuo*. Purification by chromatography on silica gel (50% EtOAc/*n*-hexane) afforded the title compound **S-18** (497 mg, 1.24 mmol, 89%, α:β = 7:1) as a colourless oil. R_f = 0.2 (80% Et_2O/pet. ether); **α anomer:** **^{1}H NMR** (400 MHz; $CDCl_3$) δ 7.38-7.27 (5H, m, Ar-H), 5.71-5.59 (1H, m, H-4), 5.37 (1H, dd, *J* = 3.4, 3.4 Hz, H-1), 4.91 (1H, ddd, *J* = 48.6, 8.7, 3.1 Hz, H-3), 4.80 (1H, dd, *J* = 12.1, 2.2 Hz, C*H*H Bn), 4.68 (1H, dd, *J* = 12.1, 2.2 Hz, C*H*H Bn), 4.43 (1H, d, *J* = 8.7 Hz, H-5), 3.90 (1H, ddd, *J* = 10.3, 2.9, 2.9 Hz, H-2), 3.76-3.74 (3H, m, COOC*H*$_3$), 2.83-2.71 (2H, m, CH_2 Lev), 2.68-2.58 (2H, m, CH_2 Lev), 2.19 (3H, s, CH_3 Lev); **^{13}C {^{1}H} NMR** (101 MHz; $CDCl_3$) δ 206.3 (s, C=O COMe Lev), 171.6 (s, C=O Lev), 168.6 (d, *J* = 2.5 Hz, *C*OOMe), 137.5 (C_q), 128.4 (CH), 127.9 (CH), 127.9 (CH), 93.4 (d, *J* = 8.2 Hz, C-1), 89.2 (d, *J* = 188.2 Hz, C-3), 74.9 (d, *J* = 15.6 Hz, C-2), 73.4 (d, *J* = 2.3 Hz, CH_2 Bn), 69.6 (d, *J* = 5.8 Hz, C-5), 68.3 (d, *J* = 21.5 Hz, C-5), 52.9 (s, COO*C*H$_3$), 37.8 (s, CH_2 Lev), 29.9 (s, CH_3 Lev), 27.8 (s, CH_2 Lev); **^{13}C-GATED** (101 MHz, $CDCl_3$) 93.4 ($^1J_{C1-H1}$ = 173 Hz, C1); **^{19}F NMR** (376 MHz; $CDCl_3$) δ -204.7 (dddd, *J* = 48.7, 10.3, 10.3, 4.0 Hz); **HRMS** (ESI) *m/z* found: $(M+NH_4)^+$ 416.1721, $C_{19}H_{27}FNO_8$ requires 416.1721.

Methyl (4-*O*-levulinoyl-2-*O*-benzyl-3-deoxy-3-fluoro-1-*O*-(*N*-[phenyl]trifluoroacetimidoyl)-α-D-mannopyranoside) uronate (S-19)

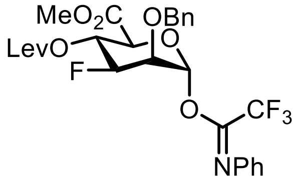

To a stirred suspension of hemiacetal **S-18** (337 mg, 0.85 mmol, 1.0 eq.) and K_2CO_3 (140 mg, 1.01 mmol, 1.2 eq.) in 20:1 acetone:water (11.4 mL) at 0 °C was slowly added 2,2,2-trifluoro-*N*-phenylacetimidoyl chloride (0.20 mL, 1.25 mmol, 1.5 eq.). The mixture was allowed to warm to room temperature and was stirred for 3 h. The reaction mixture was then concentrated *in vacuo* and redissolved in EtOAc (200 mL) and water (50 mL). The organic layer was separated, dried ($MgSO_4$) and concentrated *in vacuo*. Purification by chromatography on neutralised silica gel (30% EtOAc/*n*-hexane + 0.1% NEt_3) afforded the title compound **S-19** (442 mg, 0.78 mmol, 92%) as a colourless oil. R_f = 0.3 (30% EtOAc/pet. ether); **$[\alpha]_D^{23}$** +15 (c 1.0, $CHCl_3$); **^{1}H NMR** (400 MHz; $CDCl_3$) δ 7.37-7.27 (7H, m, Ar-H), 7.17-7.07 (1H, m, Ar-

H), 6.78-6.74 (2H, m, Ar-H), 6.38 (1H, br. s, H-1), 5.70 (1H, dd, J = 9.5, 9.5 Hz, H-4), 4.90 (1H, ddd, J = 48.4, 9.2, 3.3 Hz, H-3), 4.78 (1H, d, J = 12.2 Hz, C*H*H Bn), 4.71 (1H, d, J = 12.2 Hz, C*H*H Bn), 4.33 (1H, d, J = 9.4 Hz, H-5), 4.12-4.04 (1H, m, H-2), 3.78 (3H, s, CO_2Me), 2.85-2.72 (2H, m, CH_2 Lev), 2.71-2.59 (2H, m, CH_2 Lev), 2.20 (3H, s, CH_3 Lev); **^{13}C {^{1}H} NMR** (101 MHz; $CDCl_3$) δ 206.0 (C=O COMe Lev), 171.5 (C=O Lev), 167.2 (d, J = 2.3 Hz, *C*O_2Me), 142.8 (C_q NPh), 141.9 (q, J = 37.0 Hz, *C*=NPh), 136.8 (C_q), 128.8 (CH), 128.5 (CH), 128.1 (CH), 128.1 (CH), 124.8 (CH), 119.4 (CH), 120.0-111.8 (m, CF_3), 94.9-94.4 (m, C1), 88.4 (d, $^1J_{C\text{-}F}$ = 192.1 Hz, C3), 73.5 (d, $^4J_{C\text{-}F}$ = 1.9 53.1 (s, CO_2CH_3), 37.7 (CH_2 Lev), 29.8 (CH_3 Lev), 27.8 (CH_3 Lev); **^{13}C-GATED** (101 MHz, $CDCl_3$) 94.6 ($^1J_{C1\text{-}H1}$ = 189 Hz, C1); **^{19}F NMR** (376 MHz; $CDCl_3$) δ −65.7 (br. s, CF_3); −204.2 (d, J = 49.9 Hz).

S2. Anomeric Triflate Formation and Degradation

2.1 General experimental

NMR spectra were recorded on Bruker AV-I-500 NMR spectrometer. The multiplicity of signals is indicated by the following abbreviations: s (singlet), d (doublet), ddd (doublet of doublets of doublets), m (multiplet), J coupling values in **^{1}H NMR** spectra are provided to one decimal place.

2.2 Experimental procedure

Methyl (trifluormethylsulfonyl 4-*O*-acetyl-2-*O*-benzyl-3-deoxy-3-fluoro-α-D-mannopyranoside) uronate (8)

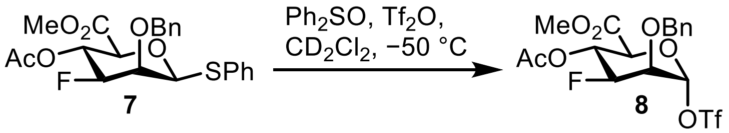

Thioglycoside donor **7** (13 mg, 0.03 mmol, 1.0 eq.) and diphenyl sulfoxide (8 mg, 0.039 mmol, 1.3 eq.) were co-evaporated with toluene and then dissolved in deuterated dichloromethane (0.6 mL) and transferred to an NMR tube under an atmosphere of N_2. The tube was cooled to −80 °C and Tf_2O (6.6 μL, 0.039 mmol, 1.3 eq.) was added. The NMR tube was placed in the NMR spectrometer and allowed to warm up to −70 °C.[10] ^{1}H and ^{19}F NMR spectra were then taken and the sample was allowed to gradually warm up with further ^{1}H and ^{19}F NMR spectra taken in 10 °C increments until degradation of the triflate was observed at −20 °C. **^{1}H NMR** (500 MHz; CD_2Cl_2, −50 °C) δ 6.07 (1H, d, J = 3.4 Hz), 5.50 (1H, ddd, J = 9.4, 9.3, 9.3 Hz, H-4), 4.93 (1H, ddd, J = 47.7, 9.1, 3.3 Hz, H-3), 4.78 (1H, d, J = 11.6 Hz, C*H*H Bn), 4.68 (1H, d, J = 11.5 Hz, C*H*H Bn), 4.34 (1H, d, J = 9.3 Hz, H-5), 4.16-4.03 (1H, m, H-2), 3.70 (3H, s,

CO_2CH_3), 2.10 (3H, s, CH_3 OAc); **^{19}F {^{1}H} NMR** (471 MHz; CD_2Cl_2, −50 °C) δ −75.5 (CF_3), −205.1 (CHF).

2.3 Stacked ^{1}H NMR Spectra of Formation and Degradation of Triflate 8

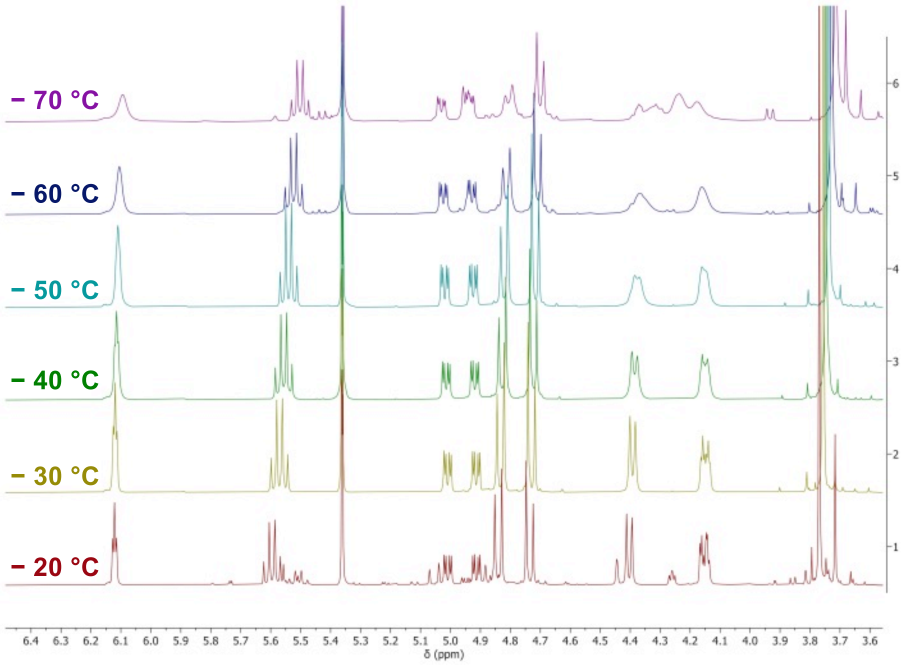

Figure S1. *Stacked ^{1}H NMR spectra (500 MHz, CD_2Cl_2) of the reaction mixture of thioglycoside 7, Ph_2SO and Tf_2O from −70 °C and increasing temperature in 10 °C increments. Complete conversion of thioglycoside 7 to triflate* ***8*** *was observed at −50 °C and degradation of triflate* ***8*** *was observed at −20 °C.*

S3. Exchange NMR analysis of triflate 8

Chemical Exchange Saturation Transfer (CEST) NMR spectroscopy. CEST NMR was utilized in ^{1}H and ^{19}F NMR spectroscopy. CEST NMR spectra were recorded by incrementing the saturation frequency offset over a domain of interest. For ^{1}H CEST NMR a window of δ_H = 7.0 to 5.0 ppm was chosen; for ^{19}F CEST NMR a window of typically δ_F = −73 to −79 ppm was used. Before each experiment, the 90°-degree pulse was calibrated. Saturation field strengths were chosen to be 20, 30, 40, and 60 Hz for ^{1}H CEST and 50, 75, 100, and 150 Hz for ^{19}F CEST to be fitted to the set of Bloch-McConnell equations. The saturation was achieved either by CW saturation on a Bruker AV-500 NMR instrument (500 MHz and 471 MHz for ^{1}H and ^{19}F respectively). Finally, saturation time was set to 3 seconds; the relaxation delay was set one second longer than the saturation time (4 seconds); number of scans was set to 4 per saturation frequency. CEST profiles were constructed using 1D NMR spectra with saturation at variable positions. A spectrum with no saturation is required as reference named $M_z(0)$. The

signal intensity for the α-triflate resonances were determined for all individual spectra after phasing and applying baseline correction. The signal intensity of every spectrum was divided by the signal intensity of the unaltered spectrum ($M_z(0)$) to obtain the relative signal intensity of the main observable species as function of saturation frequency. Plotting the relative intensity of the α-triflate signal as function of the saturation frequency gives corresponding CEST profile.

Fitting ^{1}H and ^{19}F CEST NMR Profiles to the Bloch-McConnell Equations. Experimental ^{1}H and ^{19}F CEST profiles were quantitatively analyzed using a two-site chemical exchange model governed by the Bloch–McConnell equations under continuous-wave (CW) irradiation. The exchanging system was described by a major observable pool (A, in this case corresponding to the $^4C_1$ conformer of the α-triflate) and a minor exchanging pool (B, in this case corresponding to the $^1C_4$ conformer of the α-triflate). The time evolution of the magnetization components for pools A and B under CW saturation is described by:

$$\begin{cases} \frac{dM_x^A(t)}{dt} = -\Delta\Omega^A M_y^A(t) - R_2^A M_x^A(t) + k_{B\to A} M_x^B(t) - k_{A\to B} M_x^A(t) \\ \frac{dM_x^B(t)}{dt} = -\Delta\Omega^B M_y^B(t) - R_2^B M_x^B(t) + k_{A\to B} M_x^A(t) - k_{B\to A} M_x^B(t) \\ \frac{dM_y^A(t)}{dt} = +\Delta\Omega^A M_x^A(t) - R_2^A M_y^A(t) - \Omega_1 M_z^A(t) + k_{B\to A} M_y^B(t) - k_{A\to B} M_y^A(t) \\ \frac{dM_y^B(t)}{dt} = +\Delta\Omega^B M_x^B(t) - R_2^B M_y^B(t) - \Omega_1 M_z^B(t) + k_{A\to B} M_y^A(t) - k_{B\to A} M_y^B(t) \\ \frac{dM_z^A(t)}{dt} = +\Omega_1 M_y^A(t) - R_1^A(M_z^A(t) - M_z^A(0)) + k_{B\to A} M_z^B(t) - k_{A\to B} M_z^A(t) \\ \frac{dM_z^B(t)}{dt} = +\Omega_1 M_y^B(t) - R_1^B(M_z^B(t) - M_z^B(0)) + k_{A\to B} M_z^A(t) - k_{B\to A} M_z^B(t) \end{cases}$$

In these equations, R_1^i and R_2^i denote the longitudinal and transverse relaxation rate constants of pool i, defined as the inverse of the corresponding relaxation times ($R_1^i = 1/T_1^i$ and $R_2^i = 1/T_2^i$). The applied frequency field strength is expressed as the angular frequency Ω_1, which is related to the experimentally applied saturation field B_1 (in Hz) by $\Omega_1 = 2\pi B_1$ (in rad·s^{-1}). The frequency offset of each exchanging pool relative to the applied saturation frequency is given by $\Delta\Omega^B = 2\pi(\upsilon_{sat} - \upsilon_i)$, where υ_{sat} is the irradiation frequency and υ_i is the resonance frequency of pool i. The parameters $k_{A\to B}$ and $k_{B\to A}$ represent the forward and backward chemical exchange rate constants between pools A and B, respectively.

Under continuous-wave saturation, the above set of coupled differential equations was solved using the analytic steady-state solution for a two-pool exchange system as implemented in the `cest.sources` MATLAB toolbox.[11] For each applied saturation offset and saturation field strength, the longitudinal magnetization of pool A after the experimental saturation period ($t_{sat} = 3.0$ seconds) was calculated. The experimentally measured CEST profile corresponds to the normalized longitudinal magnetization of pool A:

$$Z(\upsilon_{sat}) = \frac{M_z^A(t_{sat}, \upsilon_{sat})}{M_z^A(0)}$$

where $M_z^A(0)$ represents the equilibrium longitudinal magnetization in the absence of saturation, experimentally obtained from the reference spectrum. For each nucleus (^{1}H and ^{19}F), CEST datasets recorded at multiple saturation field strengths were fitted simultaneously to a single parameter set (global multi-B_1 fit). The saturation field strengths used were 20, 30, 40, and 60 Hz for ^{1}H CEST and 50, 75, 100, and 150 Hz for ^{19}F CEST as described above. During fitting, the experimentally applied B_1 values were converted to angular frequencies ($\Omega_1 = 2\pi B_1$) and explicitly included in the simulation.

Longitudinal relaxation rates (R_1^A and, where applicable, R_1^B) were fixed to values determined independently from inversion-recovery experiments. Because the minor pool (pool B) was typically not directly observable in conventional 1D spectra, its longitudinal relaxation rate (R_1^B) could not be determined independently by inversion–recovery experiments. Therefore, R_1^B was approximated using the experimentally measured R_1^A value. This approximation is justified because the two conformers are structurally closely related and expected to exhibit similar dipolar relaxation behavior. Moreover, sensitivity analysis showed that variation of R_1^B within a physically reasonable range had a negligible influence on the fitted exchange rate constants and population ratios. Consequently, R_1^B was fixed during the global fitting procedure.

The remaining parameters were optimized by nonlinear least-squares minimization across all saturation offsets and all B_1 datasets simultaneously. The fitted parameters comprised the forward and backward exchange rate constants ($k_{A\to B}$ and $k_{B\to A}$), the transverse relaxation rates (R_2^A and R_2^B), the chemical shift difference between the exchanging pools ($\Delta\Omega = \Omega^B - \Omega^A$), and the relative equilibrium magnetization of pool B ($M_z^B(0)$). From this, the equilibrium ratio (f_B) follows directly as follows.

$$\frac{M_z^B(0)}{M_z^A(0)} = \frac{k_{A\rightarrow B}}{k_{B\rightarrow A}} = f_B$$

Selective 1D ^{19}F EXSY NMR. Before each experiment, the 90°-degree pulse was calibrated and then the selective excitation offset was set to the resonance of interest. The gaussian shaped selection pulse was set to span ± 100 Hz (± 0.2 ppm) and was on resonance with the glycosyl α-triflate (δ_F = −75.7 ppm). The power levels of the excitation pulse were calculated against the actual 90°-degree pulse and the selection window. Relaxation delays were typically set to $5T_1$ of the triflates, and the number of scans were set to 8. The longest mix times were determined empirically so that they fit within the initial rate approximation (± 10% conversion to exchanging partner). For experiments where the shortest mix time was larger than 80 ms, a pulse sequence that contains a Z-gradient element to crush zero-quantum magnetization and clean up artifacts was used (selnogp). When the shortest mix time was much lower than 80 ms, the versions of the above experiments without the Z-gradient element were utilized.

Initial Rate Approximation for ^{19}F EXSY Experiments. In general, the α-triflate dissociates to form a triflate anion (OTf^-) in two possible mechanisms: S_N1-like leaving of the triflate anion or getting expelled by an incoming triflate in an S_N2-like manner. The rate law of the corresponding reactions is described in equation S1 and S2 for the intramolecular glycosyl stabilization and intermolecular glycosyl stabilization respectively. Both processes can proceed simultaneously; hence the overall rate law could be a combination of both (Eq. S3).

$$R_{\alpha\rightarrow OTf^-,inter} = \frac{d[OTf^-]}{dt} = -\frac{d[\alpha]}{dt} = k_{inter}\cdot[\alpha] \qquad \text{(S1)}$$

$$R_{\alpha\rightarrow OTf^-,intra} = \frac{d[OTf^-]}{dt} = -\frac{d[\alpha]}{dt} = k_{intra}\cdot[\alpha]\cdot[OTf^-] \qquad \text{(S2)}$$

$$R_{\alpha\rightarrow OTf^-} = \frac{d[OTf^-]}{dt} = -\frac{d[\alpha]}{dt} = k_{inter}\cdot[\alpha] + k_{intra}\cdot[\alpha]\cdot[OTf^-] \qquad \text{(S3)}$$

Selective 1D ^{19}F EXSY NMR is a suitable method to study the exchange. By applying a selective excitation pulse on the α-triflate resonance, formation of triflate anion can be measured despite the high population triflate already present in the reaction mixture. This is possible for two main reasons: i) the resonances of both the α-triflate and triflate anion are sufficiently separated to selectively excite the α-triflate in ^{19}F NMR, and ii) only excited-state nuclei are detected in EXSY NMR. Varying the delay (mix time, τ_m) between the excitation of the α-triflate resonance and the spectrum acquisition allows to measure different degrees of

conversion for the α-triflate into the triflate anion resonance. The initial triflate formation is linear and kinetics can be described according to the initial rate approximation. Over the initial linear interval, the reaction rate is equal to the α-triflate consumption and triflate anion formation (Eq. S4 and S5). Herein, $[\alpha]_t$ = concentration excited state α-triflate a set mix time after applying the excitation pulse on the α-triflate resonance; $[\alpha]_0$ = concentration excited state α-triflate directly after applying a selective excitation pulse on the α-triflate resonance; $[OTf^-]_t$ = concentration excited state OTf^- a set mix time after applying the excitation pulse on the α-triflate resonance; $[OTf^-]_0$ = concentration excited state OTf^- directly after applying a selective excitation pulse on the α-triflate resonance (hence, $[OTf^-]_0 = 0$). Substituting equation S4 into equation S2 gives the concentration excited state α-triflate in terms of concentration and mix time (Eq. S6).

$$-R_{\alpha \rightarrow OTf^-} = \frac{[\alpha]_t - [\alpha]_0}{\tau_m} \quad \text{(S4)}$$

$$R_{\alpha \rightarrow OTf^-} = \frac{[OTf^-]_t - [OTf^-]_0}{\tau_m} \quad \text{(S5)}$$

$$[\alpha]_t = [\alpha]_0 - (k_{inter} \cdot [\alpha] + k_{intra} \cdot [\alpha] \cdot [OTf^-]) \cdot \tau_m \quad \text{(S6)}$$

At the very start of the reaction, the concentration α-triflate deviates only marginally compared to the starting concentration ($[\alpha]_0$). In accordance with the initial rate approximation, equation S6 becomes equation S7. Additionally, within the initial rate approximation, only an α-triflate conversion of about ± 10% is recorded. Therefore, the concentration excited state triflate ($[OTf^-]$) is sufficiently small (especially compared to the bulk concentration non-excited triflate anion ($[OTf^-]$)) such that the backwards reaction can be neglected. Subsequently, dividing the equation by $[\alpha]_0$ simplifies the equation to S8.

$$[\alpha]_t = [\alpha]_0 - (k_{inter} \cdot [\alpha]_0 + k_{intra} \cdot [\alpha]_0 \cdot [OTf^-]) \cdot \tau_m \quad \text{(S7)}$$

$$\frac{[\alpha]_t}{[\alpha]_0} = 1 - (k_{inter} + k_{intra} \cdot [OTf^-]) \cdot \tau_m \quad \text{(S8)}$$

Within NMR spectroscopy, the concentration is proportional (with a constant, c) to the absolute integral ($\int I_x$) of the observed resonances (Eq. S9 and S10). Substituting equation S8 with S9 and S10 gives the absolute integral of the excited state α-triflate resonance as function of mix time (Eq. S11).

$$\int I_{\alpha,t} = c \cdot [\alpha]_t \quad \text{(S9)}$$

$$\int I_{\alpha,0} = c \cdot [\alpha]_0 \quad \text{(S10)}$$

$$\frac{\int I_{\alpha,t}}{\int I_{\alpha,0}} = 1 - (k_{inter} + k_{intra} \cdot [OTf^-]) \cdot \tau_m \quad \text{(S11)}$$

One complication is that T_1 relaxation occurs during the mixing time, which will reduce the absolute integral or intensity of the selected and exchanged resonances over time and plotting S11 will result in a multiexponential decay process if $k \leq T_1^{-1}$. This can be easily considered if the T_1 is known for each in the absence of exchange. However, if the T_1 for the species are very similar and thus experience similar rates of relaxation, then an approximation can be made where the integral of the α-triflate at t=0 ($I_{\alpha,0}$) is the sum of the integrals of the α-triflate and triflate anion at a given mix time (Eq. S12). This then allows Equation S13 to be rewritten in an internally consistent manner where the decay is normalized by the measurable peaks for each given mix time. The slope of the plot (Eq. S14) is directly related to the rate constants of both exchange processes.

$$\int I_{\alpha,0} = \int I_{\alpha,t} + \int I_{OTf^-,t} \quad \text{(S12)}$$

$$\frac{\int I_{\alpha,t}}{\int I_{\alpha,t} + \int I_{OTf^-,t}} = 1 - (k_{inter} + k_{intra} \cdot [OTf^-]) \cdot \tau_m \quad \text{(S13)}$$

$$Slope = k_{inter} + k_{intra} \cdot [OTf^-] \quad \text{(S14)}$$

The rates measured and determined by selective ^{19}F EXSY spectroscopy is, as described above, directly equal to the slope of the normalized absolute integral of the α-triflate ($\int I_{\alpha,t}$) versus mixing time. Hence, equation S14 is in the main text referred to as $\boldsymbol{R_{\alpha \rightarrow OTf, EXSY}}$ (Eq. S15).

$$R_{\alpha \rightarrow OTf^-,EXSY} = Slope = k_{inter} + k_{intra} \cdot [OTf^-] \quad \text{(S15)}$$

General Methods. All chemicals (Acros, Fluka, Merck, and Sigma-Aldrich) were used as received unless stated otherwise. Dichloromethane was stored over activated 4 Å molecular sieves (beads, 8-12 mesh, Sigma-Aldrich). Before use traces of water present in the donor, diphenyl sulfoxide (Ph_2SO) and tri-*tert*-butylpyrimidine (TTBP) were removed by co-

evaporation with dry toluene. The acceptors were stored in stock solutions (DCM, 0.5 M) over activated 3 Å molecular sieves (rods, size 1/16 in., Sigma-Aldrich). Trifluoromethanesulfonic anhydride (Tf_2O) was distilled over P_2O_5 and stored at −20 °C under a nitrogen atmosphere. Overnight temperature control was achieved by an FT902 Immersion Cooler (Julabo). Column chromatography was performed on silica gel 60 Å (0.04 − 0.063 mm, Screening Devices B.V.). TLC-analysis was conducted on TLC Silica gel 60 (Kieselgel 60 F254, Merck) with UV detection by (254 nm) and by spraying with 20% sulfuric acid in ethanol followed by charring at ± 150 °C or by spraying with a solution of $(NH_4)_6Mo_7O_{24} \cdot H_2O$ (25 g/l) and $(NH_4)_4Ce(SO_4)_4 \cdot 2H_2O$ (10 g/l) in 10% aq. sulfuric acid followed by charring at ± 250 °C. High-resolution mass spectra were recorded on a Thermo Finnigan LTQ Orbitrap mass spectrometer equipped with an electrospray ion source in positive mode (source voltage 3.5 kV, sheath gas flow 10, capillary temperature 275 °C) with resolution R=60.000 at m/z = 400 (mass range = 150 − 4000). ^{1}H and ^{13}C NMR spectra were recorded on a Bruker AV-300 NMR instrument (300 and 75 MHz respectively), a Bruker AV-400 NMR instrument (400 and 101 MHz respectively), a Bruker AV-500 NMR instrument (500 and 126 MHz respectively), a Bruker AV-600 NMR instrument (600 and 151 MHz respectively), or a Bruker AV-850 NMR instrument (850 and 214 MHz respectively). For samples measured in $CDCl_3$ chemical shifts (δ) are given in ppm relative to tetramethylsilane (TMS) as an internal standard or the residual signal of the deuterated solvent. Coupling constants (*J*) are given in Hz. To get better resolution of signals with small coupling constants or overlapping signals a gaussian window function (LB ± −1 and GB ± 0.5) was used on the ^{1}H NMR spectrum. All given ^{13}C-APT spectra are proton decoupled. NMR peak assignment was made using HH-COSY, and HSQC. If necessary additional HH-NOESY, HMBC and HMBC-GATED experiments were used to elucidate the structure further. The anomeric product ratios were based on the integration of ^{1}H NMR spectra of the purified products and was checked with the spectra of the crude reaction mixture.

General Procedure for Sample Preparation for VT-NMR. Thioglycoside donor **7** (10.9 mg, 25.0 µmol), Ph_2SO (5.6 mg, 27.5 µmol, 1.1 equiv.) and TTBP (15.5 mg, 62.5 µmol, 2.5 equiv.) were dissolved in anhydrous DCM-d_2 (500 µL, 0.05 M) and transferred to an oven-dried NMR tube. Three activated 3 Å molecular sieves (rods, size 1/16 in.) were added to the NMR tube and a pre-activation spectrum was recorded at r.t. Thereafter, the NMR tube was cooled to −80 °C using a dry ice/acetone bath. To the cooled solution, Tf_2O (5.5 µL, 32.5 µmol, 1.3 equiv.) was added and the sample was shaken quickly and re-cooled three times. Then, the sample was wiped dry and quickly inserted into the NMR probe which was being held at −80 °C after

which a list of kinetic and characterization experiments was conducted at intervals of 10 °C ranging from −90 °C to −30 °C. The tube is stationary throughout aquisition.

Selective 1D ^{19}F EXSY NMR of Mannuronosyl Triflate 8

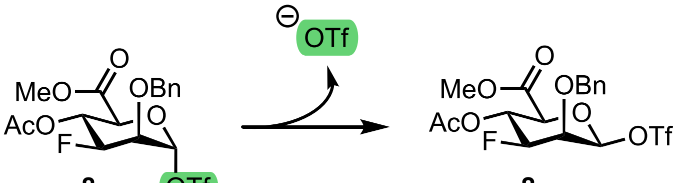

8$_{\alpha OTf}$ **8**$_{\beta OTf}$

Table S1 | ^{19}F EXSY Data for Mannuronosyl Triflate **8** at −60 °C.

Mixing Time [s]	α-OTf Integral	OTf⁻ Integral	Sum	Normalized α-OTf	Normalized OTf⁻
0.05	839327	79	839406	0.9999	**0.0001**
0.1	783046	159	783205	0.9998	**0.0002**
0.15	679797	324	680121	0.9995	**0.0005**
0.2	638622	456	639078	0.9993	**0.0007**
0.25	566450	528	566978	0.9991	**0.0009**
0.3	483499	680	484179	0.9986	**0.0014**
0.35	437299	732	438031	0.9983	**0.0017**
0.4	386173	834	387007	0.9978	**0.0022**
0.45	344486	976	345462	0.9972	**0.0028**
0.5	312181	1038	313219	0.9967	**0.0033**

Fitting of the data gives a triflate dissociation rate $\mathbf{R_{\alpha \rightarrow OTf,EXSY}}$ of **0.0066 ± 0.0005** s^{-1}

Table S2 | ^{19}F EXSY Data for Mannuronosyl Triflate **8** at −50 °C.

Mixing Time [s]	α-OTf Integral	OTf⁻ Integral	Sum	Normalized α-OTf	Normalized OTf⁻
0.05	1539680	3178	1542858	0.9979	**0.0021**
0.1	1391610	2509	1394119	0.9982	**0.0018**
0.15	1234860	3520	1238380	0.9972	**0.0028**
0.2	1128640	4593	1133233	0.9959	**0.0041**
0.25	1047110	3468	1050578	0.9967	**0.0033**
0.3	942329	6186	948515	0.9935	**0.0065**
0.35	841494	6543	848037	0.9923	**0.0077**
0.4	752382	3995	756377	0.9947	**0.0053**
0.45	697720	5193	702913	0.9926	**0.0074**
0.5	634503	7304	641807	0.9886	**0.0114**

Fitting of the data gives a triflate dissociation rate $\mathbf{R_{\alpha \rightarrow OTf,EXSY}}$ of **0.0183 ± 0.0025** s^{-1}

Table S3 | ^{19}F EXSY Data for Mannuronosyl Triflate **8** at −40 °C.

Mixing Time [s]	α-OTf Integral	OTf⁻ Integral	Sum	Normalized α-OTf	Normalized OTf⁻
**0.05**	1865190	2971	1868161	0.9984	0.0016
**0.1**	1718250	5200	1723450	0.9970	0.0030
**0.15**	1580120	9341	1589461	0.9941	0.0059
**0.2**	1460120	8433	1468553	0.9943	0.0057
**0.25**	1332520	12113	1344633	0.9910	0.0090
**0.3**	1224740	13635	1238375	0.9890	0.0110
**0.35**	1114990	14847	1129837	0.9869	0.0131
**0.4**	1037170	13955	1051125	0.9867	0.0133
**0.45**	953289	17398	970687	0.9821	0.0179
**0.5**	884624	15536	900160	0.9827	0.0173

Fitting of the data gives a triflate dissociation rate $\mathbf{R_{\alpha\rightarrow OTf,EXSY}}$ of **0.0368 ± 0.0018** s^{-1}

Table S4 | ^{19}F EXSY Data for Mannuronosyl Triflate **8** at −30 °C.

Mixing Time [s]	α-OTf Integral	OTf⁻ Integral	Sum	Normalized α-OTf	Normalized OTf⁻
0.05	1673400	7160	1680560	0.9957	**0.0043**
0.1	1536250	13856	1550106	0.9911	**0.0089**
0.15	1405960	19780	1425740	0.9861	**0.0139**
0.2	1295530	22818	1318348	0.9827	**0.0173**
0.25	1191310	27116	1218426	0.9777	**0.0223**
0.3	1102930	27714	1130644	0.9755	**0.0245**
0.35	1020020	33882	1053902	0.9679	**0.0321**
0.4	950185	33216	983401	0.9662	**0.0338**
0.45	868558	39815	908373	0.9562	**0.0438**
0.5	800452	35856	836308	0.9571	**0.0429**

Fitting of the data gives a triflate dissociation rate $\mathbf{R_{\alpha\rightarrow OTf,EXSY}}$ of **0.0893 ± 0.0033** s^{-1}

Noteworthy is that all of these exchange rates are much lower than the interconversion observed between the two ^{1}H and ^{19}F CEST exchanging partners, especially at −90 °C, where the 1D ^{19}F EXSY exchange is unmeasurably slow. Therefore, we conclude that this exchange is a conformational change of the mannuronosyl triflate from $^4C_1$ to $^1C_4$, as opposed to being a triflate anomerisation, forming an equatorial triflate in that manner.

Chemical Exchange Saturation Transfer NMR Profiles

Table S5 | ^{1}H CEST NMR Profiles of Mannuronosyl Triflate **8** at −90 °C. Fitting to the Bloch-McConnell equations gave f_B = 0.157 ± 0.051 and $k_{A\rightarrow B}$ = 627.5 ± 45.6 s^{-1}.

Saturation Freq. [ppm]	B_1 = 0.47 T	B_1 = 0.70 T	B_1 = 0.93 T	B_1 = 1.40 T
7	0.988	0.991	0.992	0.959
6.96	0.991	0.976	0.964	0.918

6.92	0.987	0.959	0.951	0.889
6.88	0.980	0.953	0.936	0.871
6.84	0.974	0.938	0.917	0.835
6.8	0.971	0.922	0.894	0.806
6.76	0.960	0.905	0.874	0.760
6.72	0.949	0.883	0.843	0.714
6.68	0.943	0.840	0.793	0.648
6.64	0.916	0.784	0.732	0.578
6.6	0.894	0.735	0.680	0.504
6.56	0.863	0.671	0.611	0.424
6.52	0.817	0.581	0.519	0.340
6.48	0.753	0.488	0.426	0.265
6.44	0.664	0.391	0.332	0.197
6.4	0.563	0.296	0.242	0.137
6.36	0.444	0.207	0.168	0.073
6.32	0.333	0.132	0.082	0.030
6.28	0.239	0.061	0.032	0.008
6.24	0.155	0.027	0.010	0.006
6.2	0.096	0.007	0.009	0.005
6.16	0.068	0.005	0.004	0.001
6.12	0.045	0.002	0.001	0.000
6.08	0.001	0.000	0.000	0.000
6.04	0.003	0.001	0.002	0.001
6	0.082	0.005	0.004	0.002
5.96	0.213	0.083	0.062	0.032
5.92	0.363	0.165	0.127	0.072
5.88	0.538	0.271	0.217	0.113
5.84	0.667	0.381	0.322	0.180
5.8	0.764	0.503	0.434	0.248
5.76	0.817	0.609	0.537	0.341
5.72	0.866	0.692	0.623	0.427
5.68	0.906	0.759	0.701	0.516
5.64	0.934	0.809	0.762	0.597
5.6	0.946	0.851	0.810	0.665
5.56	0.962	0.881	0.845	0.723
5.52	0.968	0.905	0.883	0.777
5.48	0.971	0.926	0.905	0.810
5.44	0.979	0.942	0.925	0.846
5.4	0.985	0.957	0.947	0.875
5.36	0.989	0.961	0.954	0.910
5.32	0.989	0.967	0.960	0.925
5.28	0.990	0.973	0.974	0.943
5.24	0.993	0.980	0.975	0.955
5.2	0.994	0.984	0.979	0.962
5.16	0.997	0.989	0.984	0.973
5.12	0.998	0.993	0.992	0.985
5.08	1.000	0.990	0.998	0.993
5.04	0.996	0.996	1.000	0.998
5	1.000	1.000	0.996	1.000

Table S6 | ^{19}F CEST NMR Profiles of Mannuronosyl Triflate **8** at −90 °C. Fitting to the Bloch-McConnell equations gave f_B = 0.152 ± 0.014 and $k_{A\rightarrow B}$ = 529.85 ± 66.1 s^{-1}.

Saturation Freq. [ppm]	B_1 = 1.25 T	B_1 = 1.87 T	B_1 = 2.50 T	B_1 = 3.75 T
-73	0.960	0.891	0.840	0.759
-73.12	0.929	0.844	0.777	0.635
-73.24	0.879	0.790	0.716	0.541
-73.36	0.817	0.694	0.611	0.436
-73.48	0.722	0.573	0.493	0.323
-73.6	0.582	0.439	0.363	0.233
-73.72	0.409	0.284	0.234	0.149
-73.84	0.252	0.175	0.145	0.099
-73.96	0.174	0.119	0.098	0.070

-74.08	0.161	0.103	0.084	0.055
-74.2	0.180	0.110	0.085	0.051
-74.32	0.179	0.106	0.080	0.043
-74.44	0.138	0.075	0.054	0.026
-74.56	0.075	0.040	0.029	0.015
-74.68	0.022	0.011	0.009	0.005
-74.8	0.067	0.034	0.026	0.014
-74.92	0.232	0.139	0.105	0.054
-75.04	0.442	0.306	0.241	0.130
-75.16	0.618	0.461	0.384	0.211
-75.28	0.745	0.598	0.522	0.319
-75.4	0.828	0.707	0.639	0.435
-75.52	0.878	0.790	0.737	0.526
-75.64	0.921	0.852	0.797	0.634
-75.76	0.928	0.875	0.840	0.700
-75.88	0.946	0.909	0.883	0.762
-76	0.960	0.920	0.899	0.802
-76.12	0.961	0.941	0.915	0.847
-76.24	0.977	0.956	0.936	0.867
-76.36	0.974	0.968	0.951	0.901
-76.48	0.965	0.964	0.949	0.907
-76.6	0.981	0.973	0.960	0.921
-76.72	0.988	0.986	0.969	0.937
-76.84	0.989	0.978	0.971	0.939
-76.96	0.992	0.995	0.976	0.962
-77.08	0.996	0.999	0.984	0.972
-77.2	0.998	1.000	0.986	0.992
-77.32	1.000	0.998	0.982	0.985
-77.44	0.996	0.996	0.980	0.977
-77.56	0.989	0.980	0.977	0.977
-77.68	0.993	0.997	0.969	0.977
-77.8	0.995	0.995	0.971	0.972
-77.92	1.000	0.999	0.976	0.992
-78.04	0.996	1.000	0.984	0.985
-78.16	0.989	0.998	0.986	0.977
-78.28	0.993	0.996	0.982	0.977
-78.4	0.995	0.980	0.980	0.977
-78.52	1.000	0.997	0.977	0.979
-78.64	0.996	0.997	0.997	0.977
-78.76	0.989	1.000	0.993	0.992
-78.88	0.993	0.997	1.000	1.000
-79	0.995	0.989	0.996	0.996

S4. Conformational Energy Landscapes

The workflow for the generation of computational energy landscapes (CEL)[12] was adapted for computations in ORCA5.04.[13–15] Initial geometries were constructed by a constrained relaxed potential energy surface scan using the AM1 semi-empirical method and the VerySlowConv keyword. All bond distances of directly connected atoms (i.e. C1-H1 O5-C1, C2-X2, etc.) were constrained. The C1-C2-C3-C4, C3-C4-C5-O5 and the C5-O5-C1-C2 dihedral angles were scanned from –60 to 60 degrees with 15-degree steps, generating 729 unique structures. The subsequent output geometries were then used as starting geometries for the CEL computations

with only dihedral constraints on the C1-C2-C3-C4, C3-C4-C5-O5 and the C5-O5-C1-C2 dihedral angles. The CEL computations were then performed by re-optimization of the AM1 geometries, placing constraints on the mentioned dihedral angles, with DFT using the keywords TightSCF, DEFGRID2 and SlowConv at SMD(dichloromethane)-revDSD-PBEP86-D4-def2TZVPP//PCM(dichloromethane)-B3LYP-D3BJ-def2TZVPP.[16–25] This method has been shown to yield a significant improvement in accuracy over the previously used CEL[12] methodology.[26] Slicing of the CEL maps was done in an identical way to previous work,[27] and they were visualized using a Matplotlib-based python script which generates contour plots.

Figure S2 shows the CEL map generated for the 3F ManA oxocarbenium ion. For the computations the OBn and OLev substituents have been replaced with OMe groups to reduce computational costs. The lowest energy conformation was a $^1C_4$ chair in which bridging occurred between the C5 methyl ester and C1 to form a C1-C5 dioxolanium ion. Nucleophilic attack on this intermediate could presumably proceed *via* an S_N2-like mechanism to afford an α-linked product, however empirical data shows unmodified ManA donors undergo remarkable β-selective glycosylation, indicating that such bridged structures do not contribute significantly to this glycosylation continuum. A $^1C_4$ intermediate is close in conformational space to $^3H_4$ which can likely form easily.

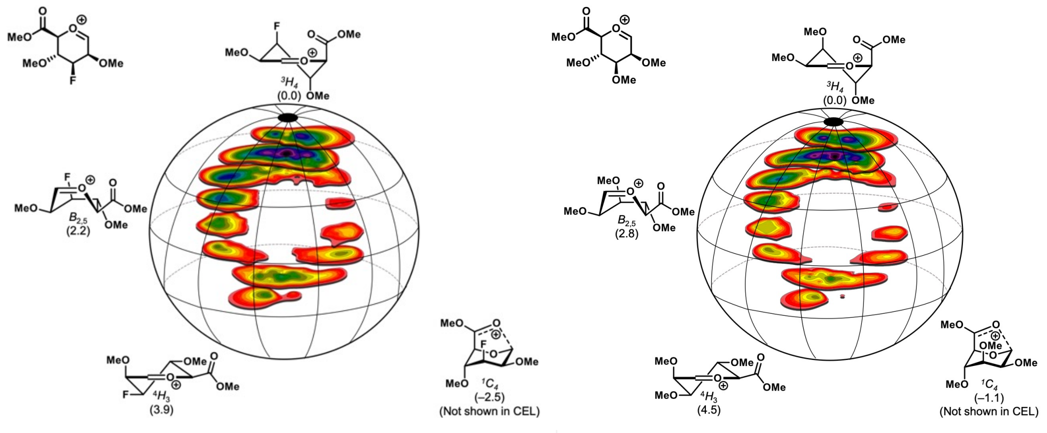

Figure S2 Conformational energy landscape (CEL) maps of 3-F-ManA (left) and ManA (right) oxocarbenium ions, in which the local minima identified are shown with their respective energy. Energies of all conformations in the CEL are computed at SMD(CH_2Cl_2)-revDSD-PBEP86-D4-def2TZVPP//PCM(CH_2Cl_2)-B3LYP-D3(BJ)-def2TZVPP and expressed as relative Gibbs free energy (T = 193.15K) in kcal mol^{-1}. Energies given are relative to the individual lowest energy oxocarbenium ion conformer.

S5. Solution-phase glycosylation reactions of donor 7

General Procedure for Tf_2O/Ph_2SO Mediated Glycosylations. Donor (0.10 mmol), Ph_2SO (26 mg, 0.13 mmol, 1.3 equiv.) and TTBP (62 mg, 0.25 mmol, 2.5 equiv.) were co-evaporated twice with dry toluene (4 Å molecular sieves) and dissolved in anhydrous DCM (2.0 mL, 0.05 M donor). Activated 3 Å molecular sieves (rods, size 1/16 in.) were added and the reaction mixture was stirred for 15 min at room temperature. The solution was cooled to −80 °C and Tf_2O (22 µL, 0.13 mmol, 1.3 eq.) was slowly added. The reaction mixture was allowed to warm to −60 °C in approximately 45 min, whereafter acceptor (0.20 mmol, 2.0 equiv.) in DCM (0.40 mL, 0.5 M) was added slowly. The reaction mixture was stirred at −60 °C for 16 hours and consequently quenched by the addition of sat. aq. $NaHCO_3$ solution (1.0 mL) at −60 °C. The mixture was then diluted with DCM and H_2O and transferred to a separatory funnel, partitioned, and the aqueous phase was extracted twice with DCM. The combined organic layers were dried over $MgSO_4$, filtered, and concentrated under reduced pressure. Flash column chromatography afforded the glycosylation product as a mixture of anomers.

Experimental Procedures

Methyl (ethyl 4-O-acetyl-2-*O*-benzyl-2-deoxy-2-fluoro-α/β-D-mannopyranosyl uronate) (7A)

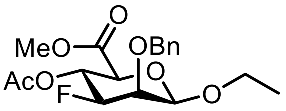

The title compound was prepared from donor **7** and ethanol using the general procedure for Tf_2O/Ph_2SO mediated glycosylations. Flash column chromatography (70:30 to 0:100, *n*-Pentane:Et_2O, v:v) yielded glycosylation product **7A** (34 mg, 91 µmol, 91%, α:β = <2:98) as a white solid. TLC: R_f 0.48, (30:70, *n*-Pentane:Et_2O, v:v); ^1H NMR (850 MHz, $CDCl_3$, HH-COSY, HSQC, HMBC): δ 7.43 – 7.40 (m, 2H, CH_{arom}), 7.34 – 7.31 (m, 2H, CH_{arom}), 7.29 – 7.25 (m, 1H, CH_{arom}), 5.62 (dt, *J* = 10.6, 9.3 Hz, 1H, H-4), 4.92 (d, *J* = 12.4 Hz, 1H, C*H*H Bn), 4.82 (d, *J* = 12.4 Hz, 1H, CH*H* Bn), 4.53 (ddd, *J* = 48.2, 9.3, 3.3 Hz, 1H, H-3), 4.49 (d, *J* = 0.8 Hz, 1H, H-1), 4.02 (dq, *J* = 9.5, 7.1 Hz, 1H, C*H*HCH3 Et), 3.99 (ddd, *J* = 8.4, 3.3, 1.0 Hz, 1H, H-2), 3.86 (dd, *J* = 9.3, 1.2 Hz, 1H, H-5), 3.75 (s, 3H, CH_3 OMe), 3.52 (dq, *J* = 9.3, 7.0 Hz, 1H, CH*H*CH_3 Et), 2.08 (s, 3H, CH_3 Ac), 1.26 (t, *J* = 7.1 Hz, 3H, $CH_2C\textit{H}_3$ Et); ^{13}C-APT NMR (214 MHz, $CDCl_3$, HSQC, HMBC): δ 169.6 (C=O Ac), 167.7 (d, *J* = 3.2 Hz, C-6), 138.1 (C_q), 128.3, 128.3, 127.7 (CH_{arom}), 100.4 (d, *J* = 10.7 Hz, C-1), 90.5 (d, *J* = 193.7 Hz, C-3), 74.7 (d, *J* = 15.8 Hz, C-2), 74.4 (d, *J* = 1.6 Hz, CH_2 Bn), 72.6 (d, *J* = 7.3 Hz, C-5), 68.2 (d, *J* = 20.5 Hz,

C-4), 66.1 (*C*H_2CH_3 Et), 52.9 (CH_3 OMe), 20.9 (CH_3 Ac), 15.1 (CH_2*C*H_3 Et); ^{13}C-GATED NMR (214 MHz, $CDCl_3$): δ 100.4 ($J_{C\text{-}1,H\text{-}1}$ = 155.5 Hz, C-1 β);

Methyl (2-monofluoroethyl 4-O-acetyl-2-*O*-benzyl-2-deoxy-2-fluoro-α/β-ᴅ-mannopyranosyl uronate) (7B)

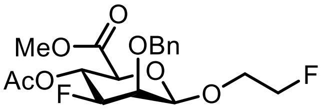

The title compound was prepared from donor **7** and 2-monofluoroethanol using the general procedure for Tf_2O/Ph_2SO mediated glycosylations. Flash column chromatography (70:30 to 0:100, *n*-Pentane:Et_2O, v:v) yielded glycosylation product **7B** (34 mg, 88 µmol, 88%, α:β = <2:98) as a white solid. TLC: R_f 0.42, (30:70, *n*-Pentane:Et_2O, v:v); ^{1}H NMR (850 MHz, $CDCl_3$, HH-COSY, HSQC, HMBC): δ 7.42 – 7.40 (m, 2H, CH_{arom}), 7.34 – 7.31 (m, 2H, CH_{arom}), 7.29 – 7.26 (m, 1H, CH_{arom}), 5.63 (dt, *J* = 10.5, 9.1 Hz, 1H, H-4), 4.91 (d, *J* = 12.2 Hz, 1H, C*H*H Bn), 4.82 (d, *J* = 12.2 Hz, 1H, CH*H* Bn), 4.66 (dddd, *J* = 48.9, 10.5, 8.2, 2.2 Hz, 1H, CH_2C*H*HF), 4.60 (d, *J* = 1.6 Hz, 1H, H-1), 4.55 (ddd, *J* = 48.1, 9.1, 3.3 Hz, 1H, H-3), 4.54 (dddd, *J* = 47.5, 10.5, 3.7, 2.4 Hz, 1H, CH_2CH*H*F), 4.11 (dddd, *J* = 36.4, 12.3, 3.7, 2.2 Hz, 1H, C*H*HCH_2F), 4.06 (ddd, *J* = 8.9, 3.3, 1.1 Hz, 1H, H-2), 3.88 (dd, *J* = 9.2, 1.1 Hz, 1H, H-5), 3.85 (dddd, *J* = 22.9, 11.7, 7.9, 2.1 Hz, 1H, CH*H*CH_2F), 3.75 (s, 3H, CH_3 OMe), 2.09 (s, 3H, CH_3 Ac); ^{13}C-APT NMR (214 MHz, $CDCl_3$, HSQC, HMBC): δ 169.6 (C=O Ac), 167.5 (d, *J* = 3.0 Hz, C-6), 137.9 (C_q), 128.3, 128.3, 127.8 (CH_{arom}), 100.6 (d, *J* = 10.6 Hz, C-1), 90.2 (d, *J* = 193.6 Hz, C-3), 82.9 (d, *J* = 169.6 Hz, CH_2*C*H_2F), 74.5 (d, *J* = 1.5 Hz, CH_2 Bn), 74.5 (d, *J* = 16.1 Hz, H-2), 72.5 (d, *J* = 7.0 Hz, H-5), 69.1 (d, *J* = 19.5 Hz, *C*H_2CH_2F), 68.1 (d, *J* = 20.8 Hz, H-4), 52.9 (CH_3 OMe), 20.8 (CH_3 Ac); ^{13}C-GATED NMR (214 MHz, $CDCl_3$): δ 100.6 ($J_{C\text{-}1,H\text{-}1}$ = 157.14 Hz, C-1 β).

Methyl (2,2-difluoroethyl 4-O-acetyl-2-*O*-benzyl-2-deoxy-2-fluoro-α/β-ᴅ-mannopyranosyl uronate) (7C)

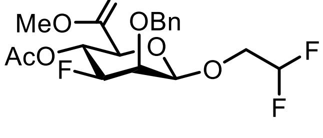

The title compound was prepared from donor **7** and 2,2-difluoroethanol using the general procedure for Tf_2O/Ph_2SO mediated glycosylations. Flash column chromatography (70:30 to 0:100, *n*-Pentane:Et_2O, v:v) yielded glycosylation product **7C** (33 mg, 82 µmol, 82%, α:β = <2:98) as a white solid. TLC: R_f 0.49, (30:70, *n*-Pentane:Et_2O, v:v); ^{1}H NMR (850 MHz,

$CDCl_3$, HH-COSY, HSQC, HMBC): δ 7.40 – 7.38 (m, 2H, CH_{arom}), 7.35 – 7.32 (m, 2H, CH_{arom}), 7.30 – 7.27 (m, 1H, CH_{arom}), 5.95 (dddd, *J* = 56.7, 53.9, 5.6, 2.9 Hz, 1H, $CH_2C\textit{H}F_2$), 5.64 (dt, *J* = 10.3, 8.8 Hz, 1H, H-4), 4.85 (d, *J* = 12.2 Hz, 1H, C*H*H Bn), 4.80 (d, *J* = 12.2 Hz, 1H, CH*H* Bn), 4.62 (t, *J* = 1.5 Hz, 1H, H-1), 4.56 (ddd, *J* = 48.0, 8.9, 3.2 Hz, 1H, H-3), 4.09 (dddd, *J* = 23.2, 11.7, 8.9, 2.6 Hz, 1H, $C\textit{H}HCHF_2$), 4.04 (ddd, *J* = 10.0, 3.2, 1.3 Hz, 1H, H-2), 3.91 (dd, *J* = 8.8, 1.0 Hz, 1H, H-5), 3.78 (dd, *J* = 22.8, 11.7, 8.7, 5.5 Hz, 1H, $CH\textit{H}CHF_2$), 3.75 (s, 3H, CH_3 OMe), 2.09 (s, 3H, CH_3 Ac); ^{13}C-APT NMR (214 MHz, $CDCl_3$, HSQC, HMBC): δ 169.6 (C=O Ac), 167.3 (d, *J* = 2.9 Hz, C-6), 137.7 (Cq), 128.4, 128.3, 127.9 (CH_{arom}), 114.2 (dd, *J* = 242.5, 239.7 Hz, $CH_2\textit{C}HF_2$), 100.3 (d, *J* = 10.2 Hz, C-1), 89.8 (d, *J* = 193.5 Hz, C-3), 74.4 (d, *J* = 1.5 Hz, CH_2 Bn), 74.0 (d, *J* = 16.3 Hz, C-2), 72.4 (d, *J* = 6.7 Hz, C-5), 68.6 (dd, *J* = 31.6, 25.4 Hz, $\textit{C}H_2CHF_2$), 67.9 (d, *J* = 21.4 Hz, C-4), 53.0 (CH_3 OMe), 20.8 (CH_3 Ac); ^{13}C-GATED NMR (214 MHz, $CDCl_3$): δ 100.3 ($J_{C\text{-}1,H\text{-}1}$ = 157.7 Hz, C-1 β).

Methyl (2,2,2-trifluoroethyl 4-O-acetyl-2-*O*-benzyl-2-deoxy-2-fluoro-α/β-ᴅ-mannopyranosyl uronate) (7D)

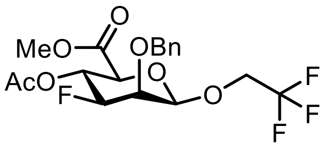

The title compound was prepared from donor 7 and 2,2,2-trifluoroethanol using the general procedure for Tf2O/Ph2SO mediated glycosylations. Flash column chromatography (70:30 to 0:100, n-Pentane:Et2O, v:v) yielded glycosylation product 7D (36 mg, 85 µmol, 85%, α:β = <2:98) as a white solid. TLC: Rf 0.58, (70:30, n-Pentane:Et2O, v:v); 1H NMR (850 MHz, CDCl3, HH-COSY, HSQC, HMBC): δ 7.40 – 7.38 (m, 2H, CH_{arom}), 7.35 – 7.32 (m, 2H, CH_{arom}), 7.30 – 7.27 (m, 1H, CH_{arom}), 5.65 (dt, J = 10.3, 8.6 Hz, 1H, H-4), 4.87 (d, J = 12.1 Hz, 1H, CHH Bn), 4.80 (d, J = 12.1 Hz, 1H, CHH Bn), 4.69 (t, J = 1.6 Hz, 1H, H-1), 4.57 (ddd, J = 47.9, 8.8, 3.2 Hz, 1H, H-3), 4.25 (dq, J = 12.6, 8.8 Hz, 1H, CHHCF3), 4.06 (ddd, J = 10.5, 3.2, 1.4 Hz, 1H, H-2), 3.97 (dq, J = 12.7, 8.3 Hz, 1H, $CH\textit{H}CF_3$), 3.93 (dd, *J* = 8.5, 0.9 Hz, 1H, H-5), 3.75 (s, 3H, CH_3 OMe), 2.09 (s, 3H, CH_3 Ac); ^{13}C-APT NMR (214 MHz, $CDCl_3$, HSQC, HMBC): δ 169.5 (C=O Ac), 167.2 (d, *J* = 2.6 Hz, C-6), 137.5 (C_q), 128.4, 128.3, 127.9 (CH_{arom}), 123.7 (q, *J* = 278.6 Hz, $CH_2\textit{C}F_3$), 99.8 (d, *J* = 10.0 Hz, C-1), 89.6 (d, *J* = 193.5 Hz, C-3), 74.4 (d, *J* = 1.4 Hz, CH_2 Bn), 73.7 (d, *J* = 16.4 Hz, C-2), 72.5 (d, *J* = 6.5 Hz, C-5), 67.9 (d, *J* = 21.6 Hz, C-4), 66.1 (q, *J* = 34.9 Hz, $\textit{C}H_2CF_3$), 52.9 (CH_3 OMe), 20.7 (CH_3 Ac); ^{13}C-GATED NMR (214 MHz, $CDCl_3$): δ 99.8 ($J_{C\text{-}1,H\text{-}1}$ = 158.3 Hz, C-1 β).

Table S7 Model glycosylation reaction results for the 3-deoxy-3-fluoro mannuronosyl donor, **7**. The stereoselectivity of the reaction is expressed as α:β and based on ^{1}H-NMR of purified α/β-product mixtures. Pre-activation-based glycosylation conditions were used: donor **7** (1.0 equiv.), Tf_2O (1.3 equiv.), Ph_2SO (1.3 equiv.), TTBP (2.5 equiv.), DCM (0.05 M), −80 to −60 °C, then add nucleophile (2.0 equiv.).

	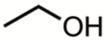	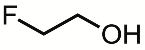	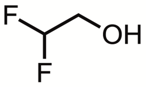	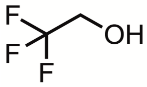
**Donor**	α:β (yield)	α:β (yield)	α:β (yield)	α:β (yield)
7 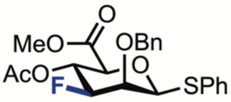	**7A** <2:98 (91%)	**7B** <2:98 (88%)	**7C** <2:98 (82%)	**7D** <2:98 (85%)

>90:10	>75:25	>60:40	50:50	<40:60	<25:75	<10:90

S6. Automated Glycan Assembly

6.1 General Experimental

AGA was performed on a commercially available Glyconeer 3.1 synthesiser at the Max Planck Institute of Colloids and Interfaces, Potsdam, Germany.[28] HPLC grade solvents were used for washing lines and as the reaction solvent. For building block, activator, TMSOTf and capping solutions, solvents obtained from a J.C. Meyer anhydrous solvent system were used. Building blocks were dried overnight under high vacuum prior to preparation of building block solutions. Oven-heated, argon-flushed flasks were used to prepare all moisture-sensitive solutions. The yields of products obtained by AGA were calculated relative to the theoretical maximum based on the resin loading. Resin loading was previously determined within the department following the procedure of Gude *et al.*[29] Photocleavage used a Vapourtec UV-150 photochemical reactor with a mercury vapour lamp. Lyophilisation was conducted using a Christ Alpha 2-4 LD freeze-drier. NMR spectra were recorded on a Varian NMR 600 MHz Premium Shielded NMR spectrometer, Bruker Avance NEO 600 MHz NMR spectrometer or a Bruker 700 MHz Ascend NMR spectrometer. MALDI-TOF analysis was conducted using an autoflex TM (Bruker). HRMS data was obtained using a Waters Xevo QToF mass spectrometer.

6.2 AGA solution preparation

Building block solution: Donor **S-17** or **S-19** (0.0625 mmol) was dissolved in CH_2Cl_2 (1 mL) under an atmosphere of argon.

Lev deprotection solution: Hydrazine acetate (725 mg, 7.87 mmol) was added to a pyridine:AcOH:H_2O (52.5 mL, *v*/*v*, 32:8:2) mixture under an atmosphere of argon . The mixture was sonicated until the hydrazine acetate had completely dissolved.

TMSOTf solution: TMSOTf (0.9 mL, 5.0 mmol) was added to CH_2Cl_2 (80 mL) under an atmosphere of argon .

Pre-capping solution: Pyridine (10 mL) was added to DMF (90 mL) under an atmosphere of argon.

Capping solution: Ac_2O (6 mL) and methanesulfonic acid (1.2 mL) were added to CH_2Cl_2 (50 mL) under an atmosphere of argon.

6.3 AGA modules[30–32]

Module A: Resin preparation

Syntheses were performed on 0.0125 mmol scale with respect to resin loading. Resin functionalised with linker **S-20** (0.30 mmol/g) was added to the reaction vessel . The resin was swollen for 20 min at room temperature with CH_2Cl_2 whilst the reagent lines to be used during the synthesis were washed. When swelling was complete, the resin was washed with DMF (3 × 3 mL, 15 seconds each) and CH_2Cl_2 (3 × 3 mL, 15 seconds each).

Module B: Acidic wash with TMSOTf solution

The resin was swollen in CH_2Cl_2 (2 mL) and the temperature of the reaction vessel adjusted to −35 °C. **TMSOTf solution** (1 mL) was then added dropwise to the reaction vessel which was incubated for 3 min. The solution was then drained from the reaction vessel and the resin was washed with CH_2Cl_2 (3 mL, 10 seconds).

Action	Cycles	Solution	Amount	T (°C)	Incubation time
**Cooling**	-	-	-	−35	
**Deliver**	1	CH_2Cl_2	2 mL	−35	-
**Deliver**	1	TMSOTf solution	1 mL	−35	3 min
**Wash**	1	CH_2Cl_2	3 mL	−35	10 sec

Module C: Imidate glycosylation

The required **Building block solution** (1 mL) was added to the reaction vessel followed by **TMSOTf solution** (1 mL). The reaction temperature was held at −35 °C for 30 min. The reaction temperature was then increased to −10 °C for 5 min after which the reaction vessel was drained. The resin was then washed with CH_2Cl_2 (3 mL for 15 seconds *then* 2 × 2 mL, 10 seconds each).

Action	Cycles	Solution	Amount	T (°C)	Incubation time
**Cooling**	-	-	-	−35	-
**Deliver**	1	BB solution	1 mL	−35	-
**Deliver**	1	TMSOTf solution	1 mL	−35	-
**Reaction time**	1			−35<br>−10	30 min<br>5 min
**Wash**	1	CH_2Cl_2	3 mL	−10	15 sec
**Wash**	2	CH_2Cl_2	2 mL	−10	10 sec

Module D: Capping

The reaction vessel temperature was increased to 30 °C and the resin was washed with DMF (2 × 3 mL, 15 seconds each). **Pre-capping solution** was added to the reaction vessel and was incubated for 1 minute after which the vessel was drained. The resin was then washed with CH_2Cl_2 (3 × 3 mL, 25 seconds each). **Capping solution** (2 mL) was added to the reaction vessel and after 10 min incubation the vessel was drained. Further **Capping solution** (2 mL) was added to the reaction vessel and was again incubated for 10 min and drained. The resin was then washed with CH_2Cl_2 (3 × 2 mL, 25 seconds each) and once with DMF for 1 minute.

Action	Cycles	Solution	Amount	T (°C)	Incubation time
**Heating**	-	-	-	30	-
**Wash**	2	DMF	3 mL	30	15 sec
**Deliver**	1	Pre-capping solution	2 mL	30	1 min
**Wash**	3	CH_2Cl_2	3 mL	30	25 sec
**Deliver**	2	Capping Solution	2 mL	30	10 min
**Wash**	3	CH_2Cl_2	2 mL	30	25 sec
**Wash**	1	DMF	2 mL	30	1 min

Module E: Lev deprotection

The temperature of the reaction vessel was increased to 40 °C and the resin was washed with CH_2Cl_2 (3 × 2 mL, 15 seconds each). CH_2Cl_2 (0.6 mL) was added to the reaction vessel followed by **Lev deprotection** solution (2 mL). After 10 min incubation, the vessel was drained, and the resin was washed with CH_2Cl_2 (3 mL, 10 seconds). This process was repeated

a further two times after which the resin was washed with DMF (3 × 2 mL, 25 seconds each) and CH_2Cl_2 (5 × 2 mL, 25 seconds each).

Action	Cycles	Solution	Amount	T (°C)	Incubation time
**Heating**	-	-	-	40	-
**Wash**	3	CH_2Cl_2	2 mL	40	15 sec
**Deliver**	3	CH_2Cl_2<br>Lev depr. solution	0.6 mL<br>2 mL	40<br>40	10 min
**Wash**		CH_2Cl_2	3 mL		10 sec
**Wash**	3	DMF	2 mL	40	25 sec
**Wash**	5	DCM	2 mL	40	25 sec

6.4 Post AGA modules

Module F: Photocleavage

Cleavage of the resulting oligosaccharides from the resin was achieved in a continuous flow photoreactor according to the previously reported procedure of Hurevich *et al.*[33] 10% MeOH in CH_2Cl_2 was used as the reaction solvent.

Module G: Normal phase purification

Compounds cleaved from the solid support were analysed using analytical normal phase HPLC (Agilent 1200 series, Method 1). The purification of the crude protected trisaccharide en-route to **9** was conducted using normal phase HPLC (Agilent 1200 series, Method 2)

- Method 1: (YMC-Diol-300 column, 150 × 4.6 mm), flow rate of 1.0 mL/min with hexane and EtOAc as eluents [isocratic 20% EtOAc (5 min), linear gradient to 100% EtOAc (30 min)].
- Method 2: (YMC-Diol-300 column, 150 × 20 mm), flow rate of 15 mL/min with hexane and EtOAc as eluents [isocratic 20% EtOAc (5 min), linear gradient to 100% EtOAc (30 min)].

Module H: Saponification

The protected oligosaccharide was dissolved in THF (0.4 mL) and 0.45 M KOH (0.09 mL) was added.[9] The reaction was stirred at room temperature for 2.5 h followed by neutralisation with Amberlite IR120 (H^+) ion exchange resin, filtration and concentration *in vacuo*.

Module I: Hydrogenolysis

In a 15 mL sample vial the crude compound obtained from **Module H** was dissolved in 2.5 mL of EtOAc:*t*-BuOH:H_2O (0.5:1:1). $Pd(OH)_2$ (100% by weight) was added and the vial was placed in a pressurised reactor. The reaction was placed under an atmosphere of hydrogen with 60 psi pressure and was stirred at room temperature overnight. The reaction mixture was then filtered through a syringe filter, washing with MeCN and H_2O, and was concentrated *in vacuo*.

Module J: Reverse phase HPLC purification

Trisaccharides **9** and **10** were initially analysed by analytical HPLC (Agilent 1200 Series, **Method 1**) and then purified by reverse phase HPLC (Agilent 1200 Series, **Method 2**).

- **Method 1**: (Hypercarb column, ThermoFisher Scientific, 150 × 4.6 mm, 3 µm) flow rate of 0.7 mL/min with H_2O (0.1% formic acid) and MeCN as eluents [isocratic (5 min), linear gradient to 30% MeCN (30 min), linear gradient to 100% MeCN (5 min)].
- **Method 2**: (Hypercarb column, ThermoFisher Scientific, 150 × 10 mm, 5 µm) flow rate of 3 mL/min with H_2O (0.1% formic acid) and MeCN as eluents [isocratic (5 min), linear gradient to 30% MeCN (30 min), linear gradient to 100% MeCN (5 min)].

Module K: Size exclusion chromatography

Hexasaccharides **11**, **12** and **13** were purified by size exclusion chromatography. A glass column was filled with a slurry of Sephadex LH-20 Size Exclusion resin in 1:1 MeOH:H_2O. The oligosaccharide was loaded onto the column as a solution in the minimum volume of water. The column was then run under gravity with fractions collected using a Bio-Rad Model 2110 Fraction Collector. The fractions were then analysed by HRMS (QTOF) and those found to contain the desired product were concentrated and analysed by ^{1}H, HSQC and ^{19}F NMR spectroscopy. Fractions containing the desired product with no observed deletion sequences were combined and lyophilised.

6.5 Oligosaccharide Synthesis

β-D-Mannopyranosyl uronate-(1→4)-β-D-mannopyranosyl uronate-(1→4)- 5-aminopentyl-α-D-mannopyranosyl uronate (9)

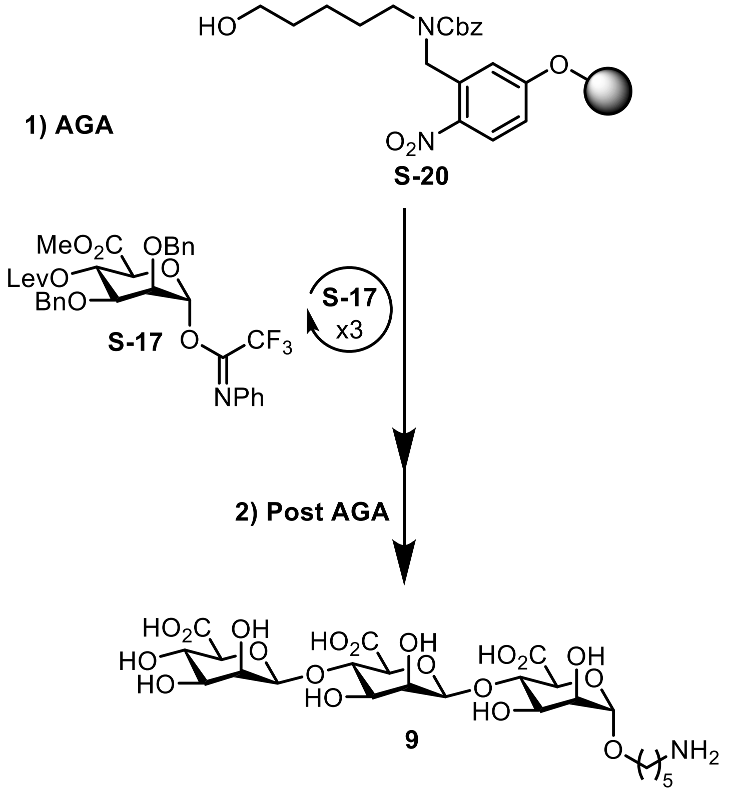

Step	BB	Modules
**AGA**	(**S-17**) × 3	**A**<br>(**B**, **C**, **D**, **E**) × 3
**Post-AGA**		**F**, **G**, **H**, **I**,<br>**J** (Method 2: 26.50 min)

Following AGA, photocleavage, global deprotection and purification, trisaccharide **9** (0.8 mg, 1.27 µmol, 10%) was isolated as a white solid. **^{1}H NMR** (700 MHz; D_2O) δ 4.88 (1H, d, *J* = 2.1 Hz, H-1), 4.68 (1H, s, H-1'), 4.63 (1H, s, H-1''), 4.03 (1H, d, *J* = 8.9 Hz), 4.01-3.99 (2H, m), 3.95 (1H, d, *J* = 3.3 Hz), 3.93-3.87 (3H, m), 3.81 (1H, d, *J* = 9.8 Hz), 3.79-3.70 (4H, m), 3.62 (1H, dd, *J* = 9.6, 3.4 Hz), 3.58-3.54 (1H, m, $OC\mathit{H}HC_4H_8NH_2$), 2.96 (2H, t, *J* = 7.5 Hz, $OC_4H_8C\mathit{H}_2NH_2$), 1.71-1.54 (4H, m, 2 × CH_2 linker), 1.48-1.37 (2H, m, CH_2 linker); **^{13}C {^{1}H} NMR** (177 MHz; $CDCl_3$) δ 174.8 (C=O), 174.3 (C=O), 174.0 (C=O), 100.1 (C1' or C1''), 99.8 (C1' or C1''), 99.5 (C1), 78.1, 77.9, 75.2, 75.1, 72.3, 72.0, 71.3, 70.2, 69.8, 69.2, 68.0, 68.0 ($O\mathit{C}H_2C_4H_8NH_2$), 39.3 ($OC_4H_8\mathit{C}H_2NH_2$), 28.0 (CH_2 linker), 26.3 (CH_2 linker), 22.3 (CH_2 linker); **HRMS** (Q-TOF) *m/z* found: $(M+H)^+$ 632.2046, $C_{23}H_{38}NO_{19}$ requires 632.2038.

β-D-Mannopyranosyl uronate-(1→4)-3-deoxy-3-fluoro-β-D-mannopyranosyl uronate-(1→4)-5-aminopentyl-α-D-mannopyranosyl uronate (10)

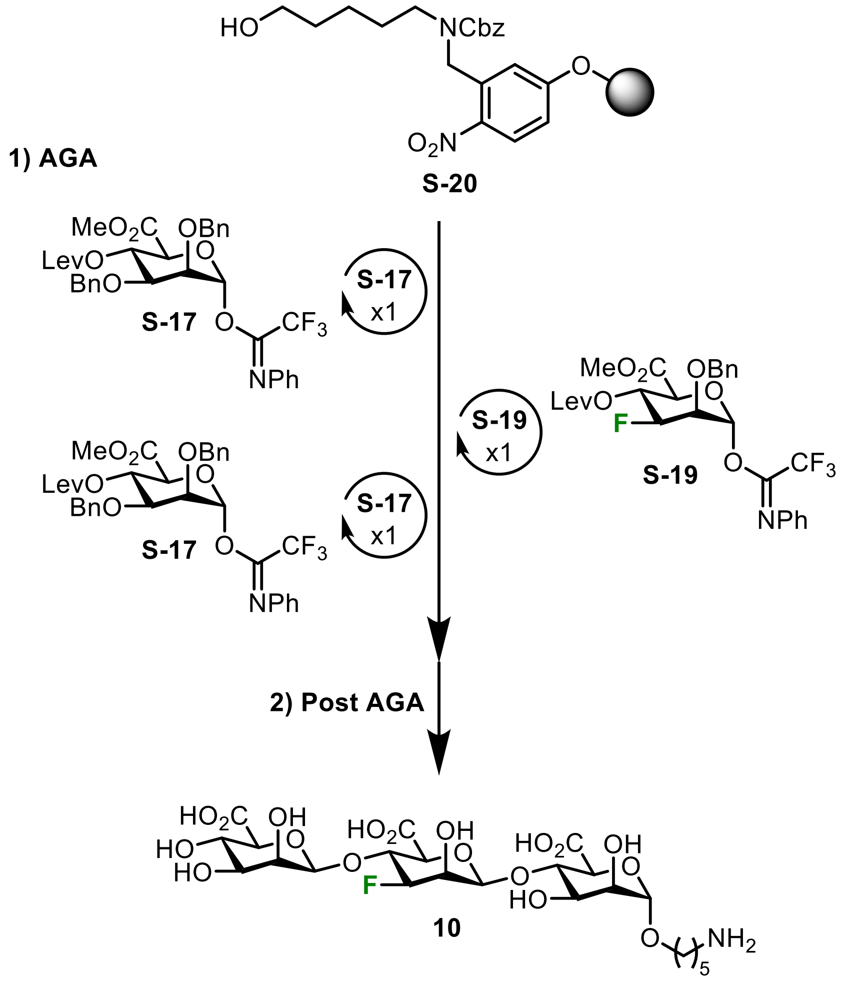

Step	BB	Modules
AGA		**A**
	S-17	**B**, **C**, **D**, **E**
	S-19	**B**, **C**, **D**, **E**
	S-17	**B**, **C**, **D**, **E**
Post-AGA		**F**, **H**, **I**, **J** (Method 2: 26.05 min)

Following AGA, photocleavage, global deprotection and purification, trisaccharide **10** (0.5 mg, 0.790 μmol, 6%) was isolated as a white solid. **^{1}H NMR** (600 MHz; D_2O) δ 4.91 (1H, d, J = 2.2 Hz, H-1), 4.71 (1H, ddd, J = 47.1, 9.5, 3.3 Hz, H-3'), 4.71 (1H, br. s, H-1' or H-1''), 4.66 (1H, br. s, H-1' or H-1''), 4.28 (1H, dd, J = 8.0, 3.4 Hz, H-2'), 4.21 (1H, ddd, J = 10.1, 10.1, 10.1 Hz, H-4'), 4.03-3.92 (5H, m), 3.81-3.68 (3H, m), 3.68-3.63 (2H, m), 3.61 (1H, dt, J = 10.4, 5.6 Hz, OC*H*HC$_4$H$_8$NH$_2$), 3.02 (2H, t, J = 7.5 Hz, OC$_4$H$_8$C*H*$_2$NH$_2$), 1.76-1.59 (4H, m, 2

× CH_2 linker), 1.54-1.42 (2H, m, CH_2 linker); **^{19}F {^{1}H} NMR** (565 MHz; D_2O) δ −196.9 (s); **HRMS** (Q-TOF) *m/z* found: $(M{-}H)^{-}$ 632.1833, $C_{23}H_{35}FNO_{18}$ requires 632.1838.

β-D-Mannopyranosyl uronate-(1→4)-β-D-mannopyranosyl uronate-(1→4)-β-D-mannopyranosyl uronate-(1→4)-β-D-mannopyranosyl uronate-(1→4)-β-D-mannopyranosyl uronate-(1→4)-5-aminopentyl-α-D-mannopyranosyl uronate (11)

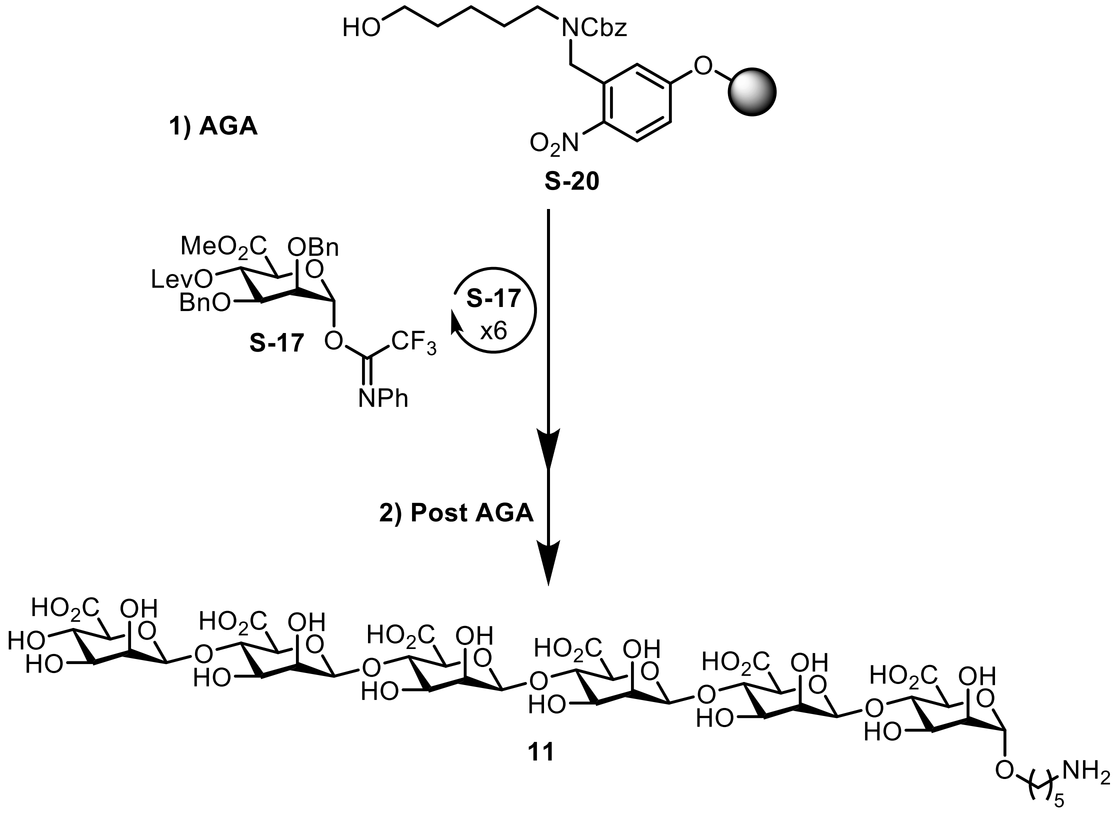

Step	BB	Modules
**AGA**	**(S-20)** × 6	A
		B, C, D, E
**Post-AGA**		F, H, I, K

Following AGA, photocleavage, global deprotection and purification, hexasaccharide **11** (1.8 mg, 1.55 µmol, 12%) was isolated as a white solid. **^{1}H NMR** (600 MHz; D_2O) δ 4.91 (1H, d, *J* = 2.1, α-H-1), 4.69 (1H, br. s, β-H-1), 4.65 (3H, br. s, 3 × β-H-1), 4.63 (1H, br. s, β-H-1), 4.05-4.02 (4H, m), 4.01-3.96 (4H, m), 3.95-3.87 (7H, m), 3.79-3.74 (9H, m), 3.69-3.65 (3H, m), 3.61 (1H, dt, *J* = 10.5, 5.6 Hz, OC*H*H$C_4H_8NH_2$), 3.02 (2H, td, *J* = 7.6, 1.7 Hz, OC_4H_8C*H*$_2NH_2$), 1.75-1.59 (4H, m, 2 × CH_2 linker), 1.55-1.43 (2H, m, CH_2 linker); **^{13}C {^{1}H} NMR** (151 MHz; $CDCl_3$) δ 175.9 (C=O), 175.3 (C=O), 175.1 (C=O), 100.1 (β-C1), 100.0 (β-C1), 99.8 (β-C1), 99.6 (α-C1), 78.0, 78.0, 75.9, 75.8, 72.7, 72.6, 71.4, 71.3, 70.4, 70.0, 69.9,

69.9, 69.5, 69.2, 68.5, 67.7 (O*C*$H_2C_4H_8NH_2$), 39.4 (OC_4H_8*C*H_2NH_2), 28.1 (CH_2 linker), 26.4 (CH_2 linker), 22.4 (CH_2 linker); **HRMS** (Q-TOF) *m/z* found: $(M-H)^-$ 1158.283, $C_{41}H_{60}NO_{37}$ requires 1158.284.

β-D-Mannopyranosyl uronate-(1→4)-β-D-mannopyranosyl uronate-(1→4)-3-deoxy-3-fluoro-β-D-mannopyranosyl uronate-(1→4)-β-D-mannopyranosyl uronate-(1→4)-β-D-mannopyranosyl uronate-(1→4)-5-aminopentyl-α-D-mannopyranosyl uronate (12)

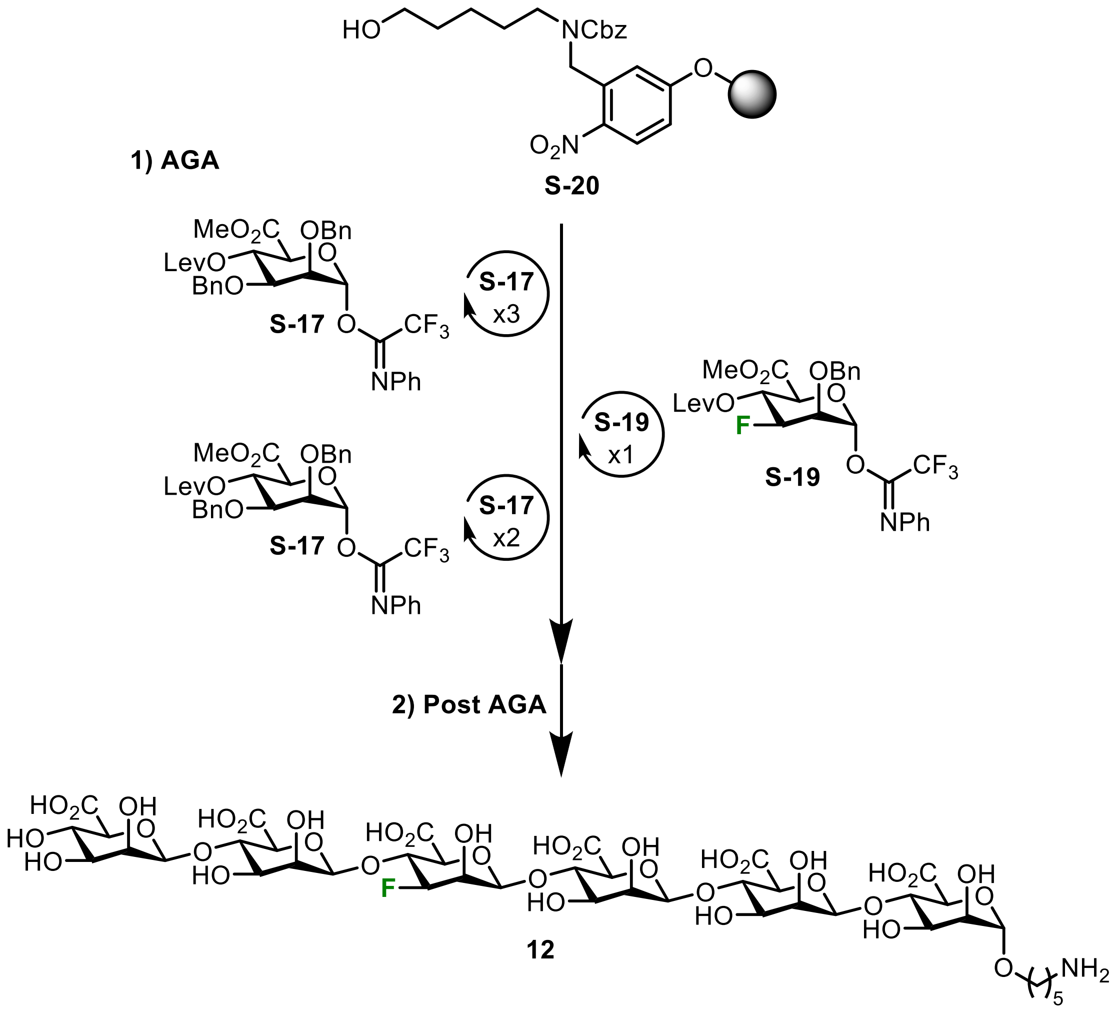

Step	BB	Modules
		A
	**(S-17)** × 3	B, C, D, E
**AGA**	**S-19**	B, C, D, E
	**(S-17)** × 2	B, C, D, E
**Post-AGA**		F, H, I, K

Following AGA, photocleavage, global deprotection and purification, hexasaccharide **12** (0.8 mg, 0.689 µmol, 6%) was isolated as a white solid. **^{1}H NMR** (600 MHz; D_2O) δ 4.91 (1H, d, J = 2.2 Hz, α-H-1), 4.69 (1H, s, β-H-1), 4.67 (1H, s, β-H-1), 4.66 (1H, s, β-H-1), 4.65 (1H, s, β-H-1), 4.63 (1H, s, β-H-1), 4.30-4.25 (1H, m, H-2’’’), 4.17 (1H, ddd, J = 10.1, 10.1, 10.1 Hz,

H-4'''), 4.06-4.01 (3H, m), 4.01-3.96 (4H, m), 3.95-3.88 (4H, m), 3.79-3.59 (13H, m), 3.02 (2H, td, J = 7.6, 1.9 Hz, $OC_4H_8CH_2NH_2$), 1.78-1.59 (4H, m, 2 × CH_2 linker), 1.53-1.44 (2H, m, CH_2 linker); **^{19}F NMR** (564 MHz; D_2O) δ −196.6 (ddd, J = 47.9 Hz); **HRMS** (Q-TOF) *m/z* found: $(M-H)^-$ 1160.277, $C_{41}H_{59}FNO_{36}$ requires 1160.280.

β-D-Mannopyranosyl uronate-(1→4)-3-deoxy-3-fluoro-β-D-mannopyranosyl uronate-(1→4)-β-D-mannopyranosyl uronate-(1→4)-β-D-mannopyranosyl uronate-(1→4)-3-deoxy-3-fluoro-β-D-mannopyranosyl uronate-(1→4)-5-aminopentyl-α-D-mannopyranosyl uronate (13)

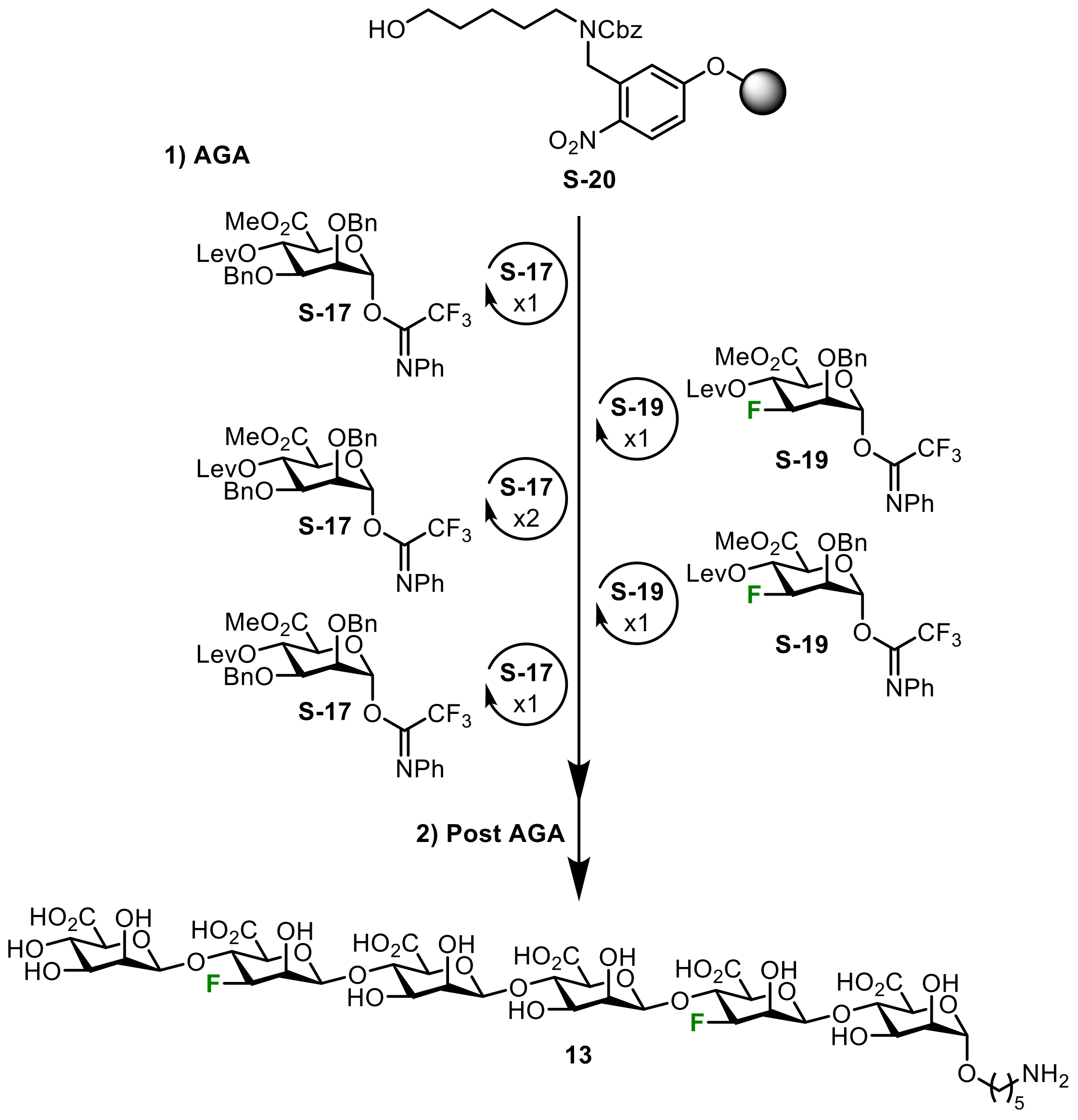

Step	BB	Modules
AGA		A
	**S-17**	B, C, D, E
	**S-19**	B, C, D, E
	**(S-17)** × 2	B, C, D, E
	**S-19**	B, C, D, E
	**S-17**	B, C, D, E
Post-AGA		F, H, I, K

Following AGA, photocleavage, global deprotection and purification, hexasaccharide **13** (0.3 mg, 0.259 µmol, 2%) was isolated as a white solid. **^{1}H NMR** (700 MHz; D_2O) δ 4.87 (1H, d, *J* = 2.2 Hz, α-H-1), 4.72-4.62 (6H, m, 2 × CHF, 5 × β-H-1), 4.26-4.21 (2H, m, H-2', H-2'''), 4.15 (2H, ddd. *J* = 10.1, 10.1, 10.0 Hz, H-4', H-4'''), 4.04-3.82 (10H, m), 3.76-3.65 (10H, m), 3.63-3.52 (4H, m), 2.98 (2H, td, *J* = 7.6, 2.4 Hz, $OC_4H_8CH_2NH_2$), 1.71-1.55 (4H, m, 2× CH_2 linker), 1.50-1.41 (2H, m, CH_2 linker); **^{19}F NMR** (564 MHz; D_2O) δ −196.5 (br. d, *J* = 31.1 Hz); **HRMS** (Q-TOF) *m/z* found: $(M-H)^-$ 1162.270, $C_{41}H_{58}F_2NO_{35}$ requires 1162.276.

S7. Anomerisation kinetics for compound 7a

General Procedure for Sample Preparation for Anomerisation Kinetics. Compound **7A** (ethanol glycosylation product from mannuronosyl donor 7) (9.2 mg, 25 µmol) was dissolved in anhydrous DCM-d_2 (500 µL, 0.05 M) and transferred to an oven-dried NMR tube. Three activated 3 Å molecular sieves (rods, size 1/16 in.) were added to the NMR tube and a pre-anomerisation spectrum was recorded at r.t. Thereafter, the tube was taken out of the magnet and cooled to −40 °C using an acetone/LN_2 bath. To the NMR tube, TMS−OTf (4.8 µL, 25 µmol, 1.0 equiv.) was added and the sample was quickly shaken and re-cooled three times before being inserted back into the cooled magnet at −40 °C. The sample was then quickly shimmed and the time between addition of the TMS−OTf and the start of the kinetics measurement was carefully recorded as the 'dead-time' of the experiment. In this case, in accordance with the AGA protocol, the sample was kept at −40 °C for 30 minutes, after which warming to −10 °C took place. Thereafter, the sample was kept at −10 °C until full conversion was observed. The results were plotted as a function of normalized concentration $(\mathbf{C}_i(t)/\mathbf{C}_i(0))$ over time.

Anomerisation Kinetics Measurement

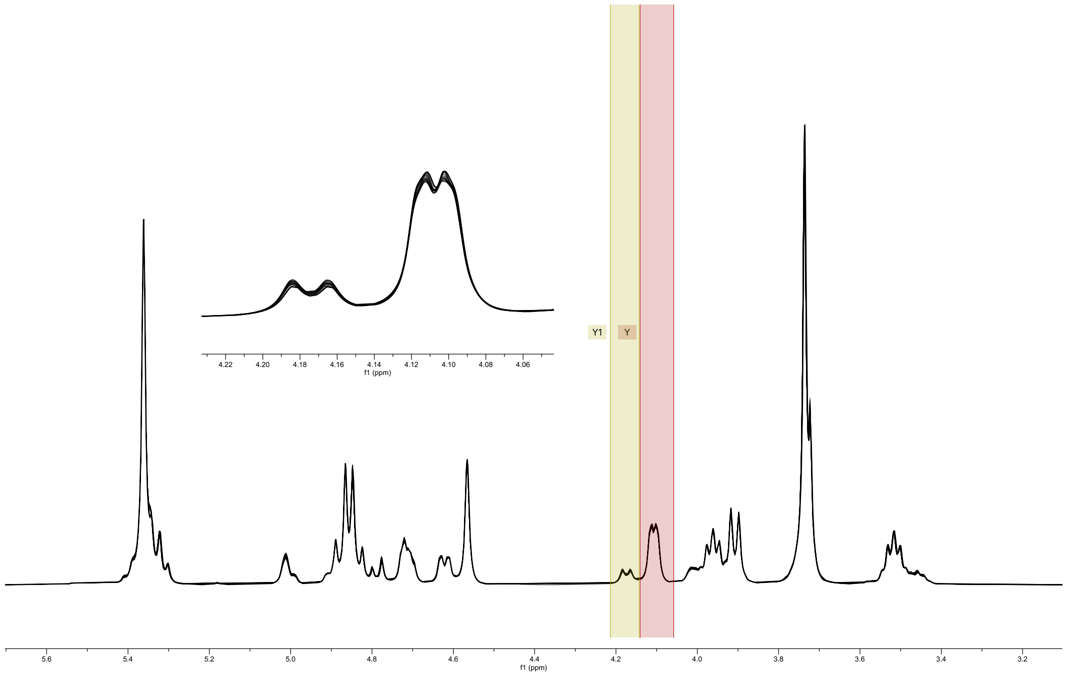

Figure S3. ^{1}H-Kinetics Experiment at −40 °C, 500 MHz, CD_2Cl_2 of compound **7A** with 1.0 equiv. TMS−OTf. The H-2 resonance integrals is used for plotting as these are not convoluted with other signals.

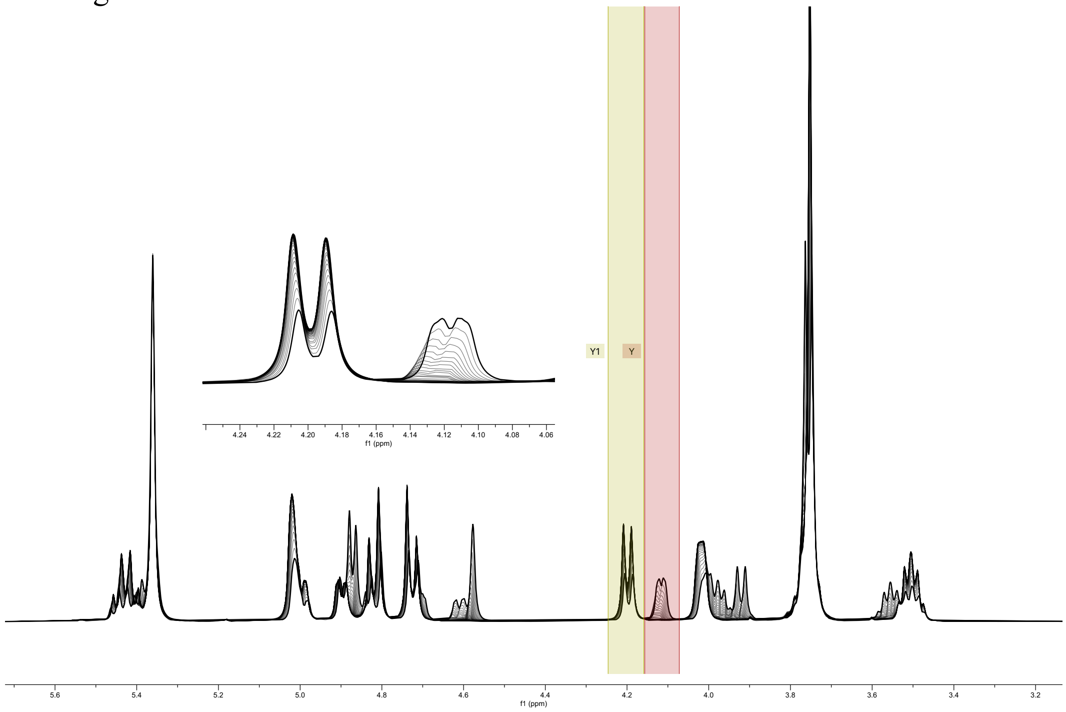

Figure S4. ^{1}H-Kinetics Experiment at −10 °C, 500 MHz, CD_2Cl_2 of compound **7A** with 1.0 equiv. TMS−OTf. The H-2 resonance integrals is used for plotting as these are not convoluted with other signals.

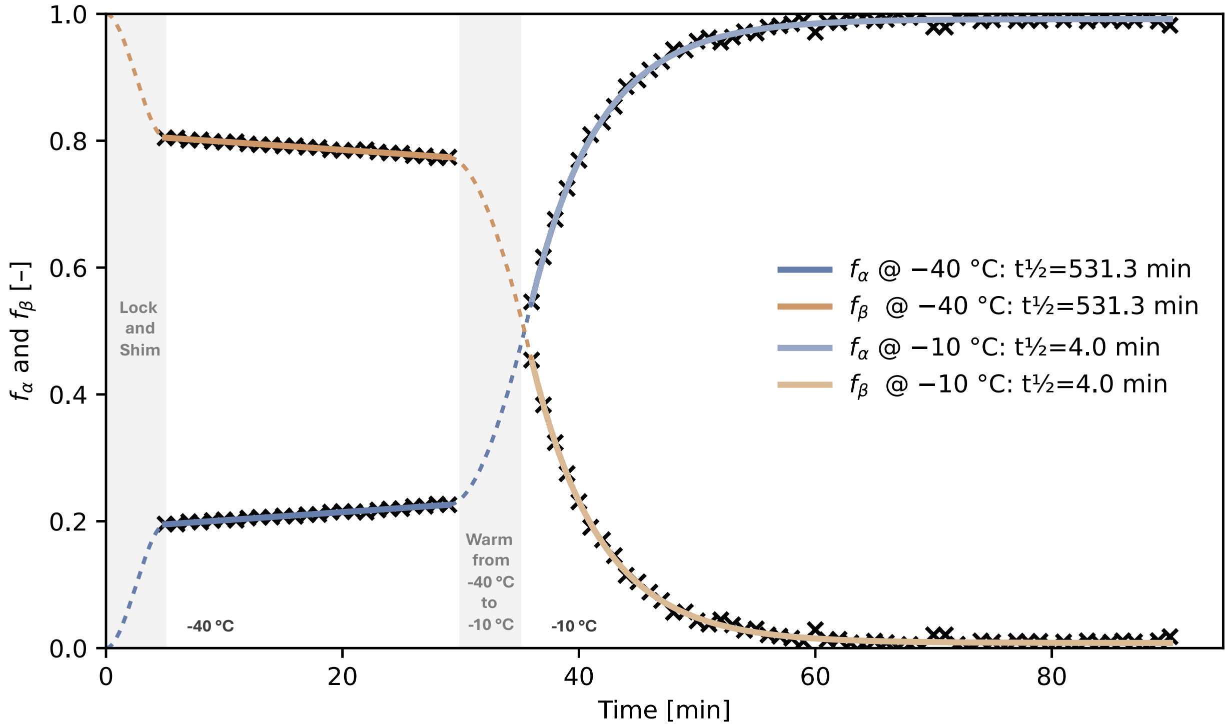

Figure S5. [1]H-Kinetics Experiment at −40 °C to −10 °C. Normalized Concentrations of both anomers are plotted as a function of time. Exponential functions were fitted to the data to calculate half-times of the species at each temperature.

S8.1D NMR spectra

3-deoxy-3-fluoro-1,2:5,6-diisopropylidene-α-D-glucofuranose (S-2)
^{1}H NMR (400 MHz, $CDCl_3$)

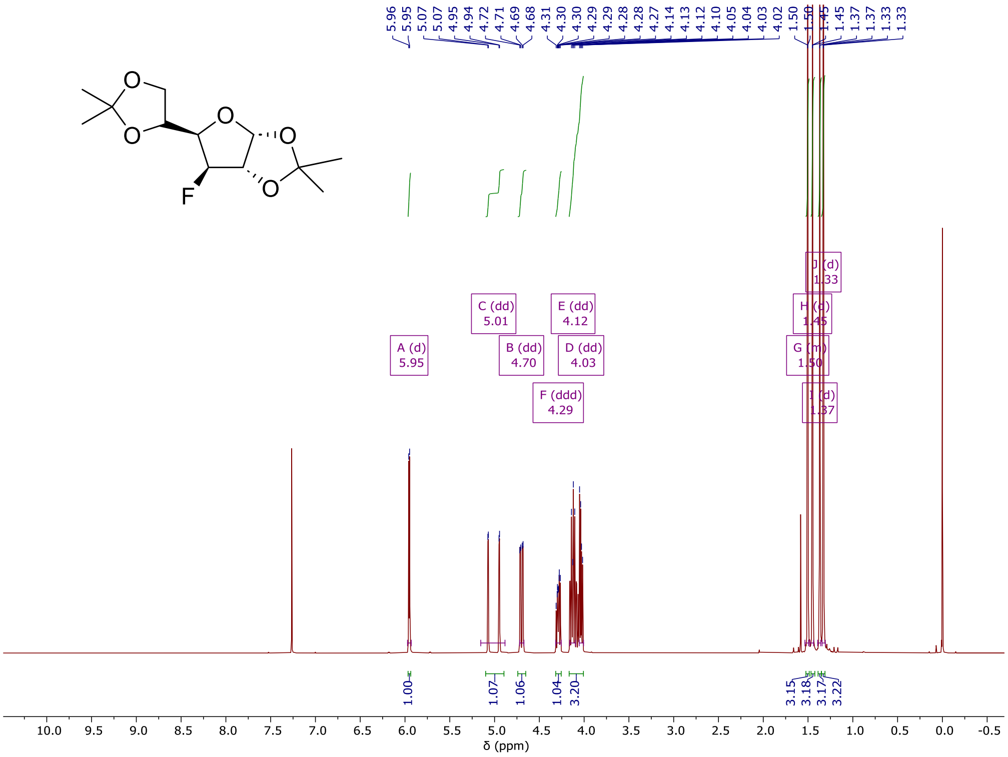

3-deoxy-3-fluoro-1,2:5,6-diisopropylidene-α-D-glucofuranose (S-2)
^{13}C NMR (101 MHz, $CDCl_3$)

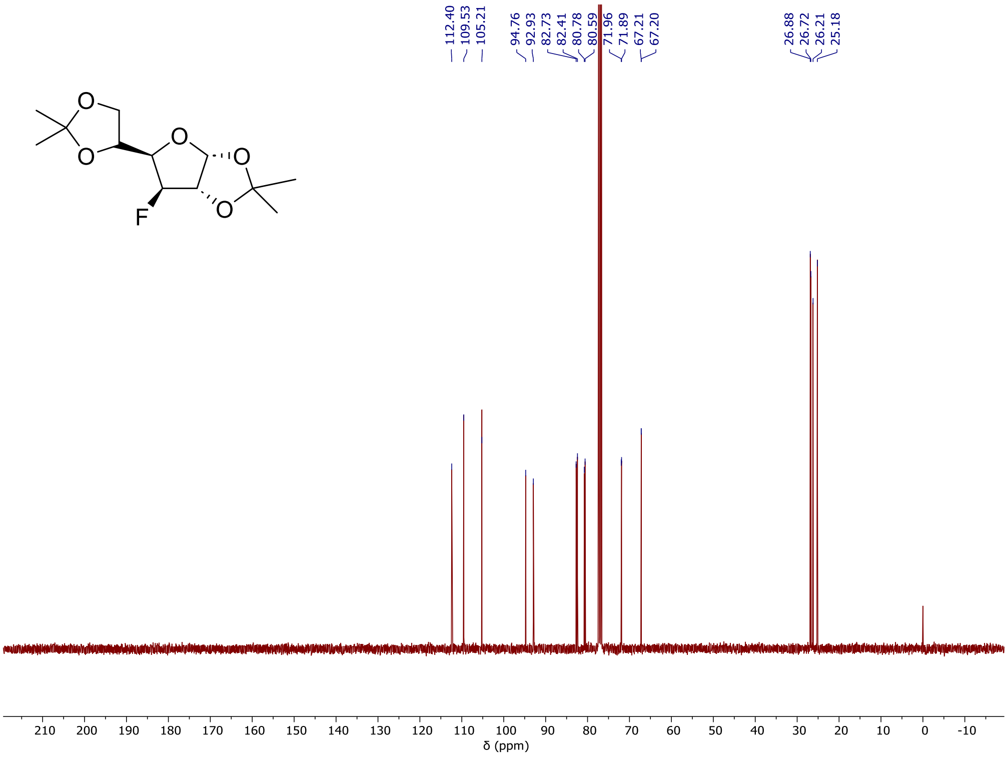

1,2,4,6-Tetra-*O*-acetyl-3-deoxy-3-fluoro-β-D-glucopyranose (S-3)
^{1}H NMR (400 MHz, CDCl$_3$)

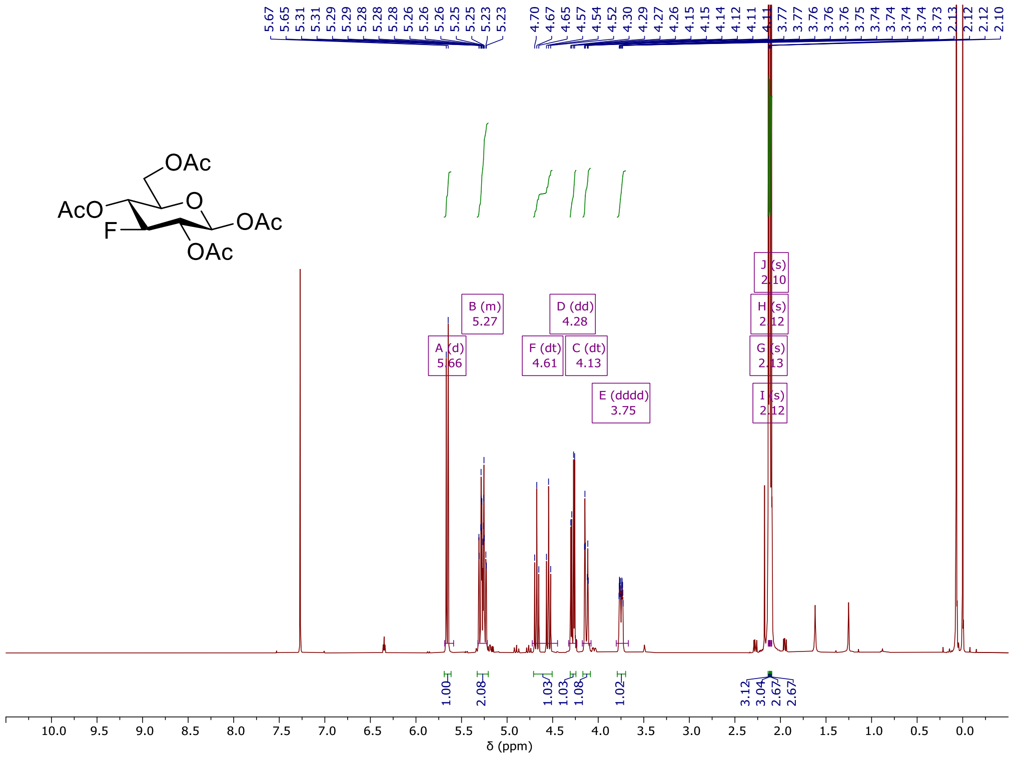

1,2,4,6-Tetra-*O*-acetyl-3-deoxy-3-fluoro-β-D-glucopyranose (S-3)
^{13}C NMR (101 MHz, CDCl$_3$)

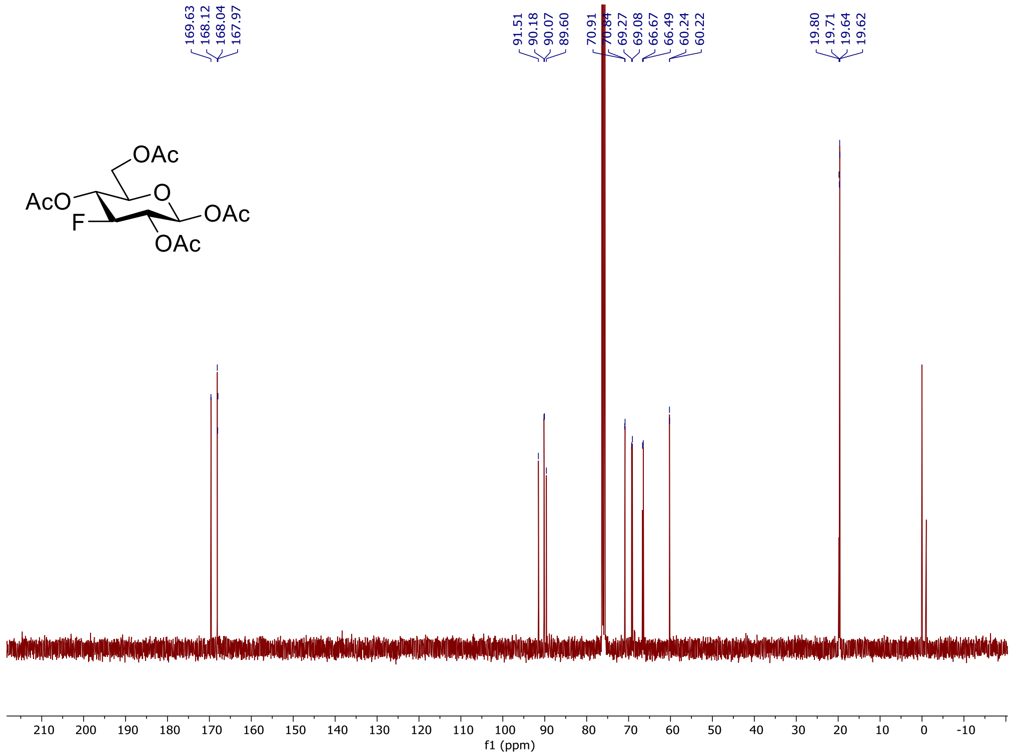

Phenyl 2,4,6-tri-*O*-acetyl-3-deoxy-3-fluoro-1-thio-β-D-glucopyranoside (S-4), ^1^H NMR (400 MHz, CDCl~3~)

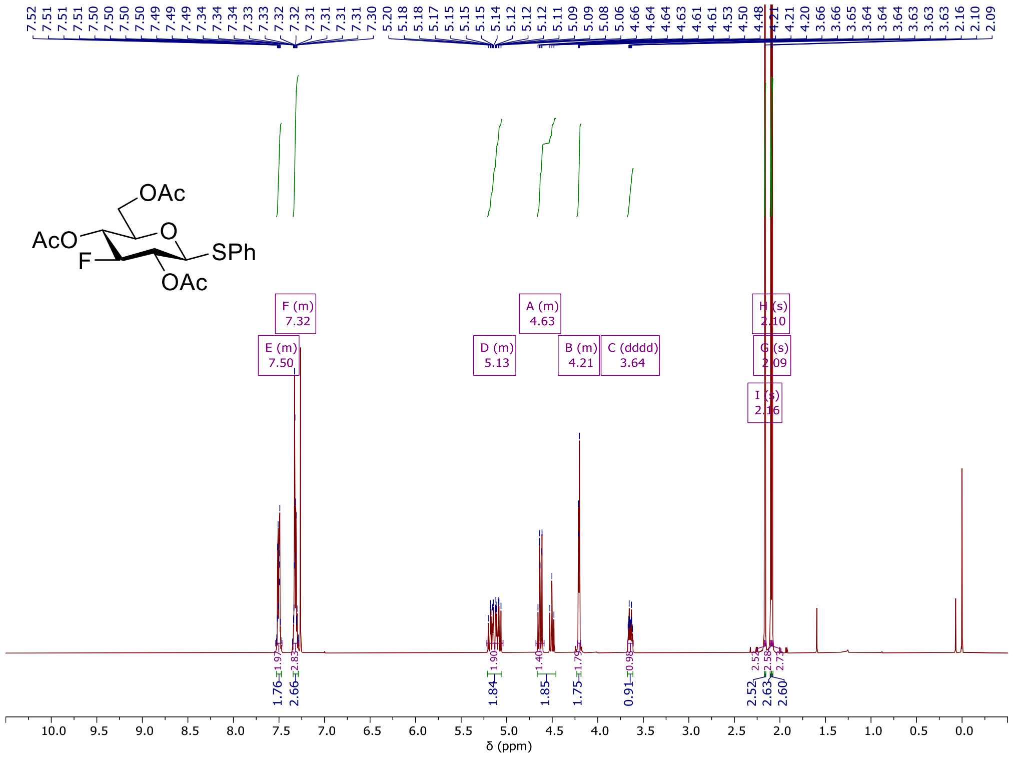

Phenyl 2,4,6-tri-*O*-acetyl-3-deoxy-3-fluoro-1-thio-β-D-glucopyranoside (S-4), ^13^C NMR (101 MHz, CDCl~3~)

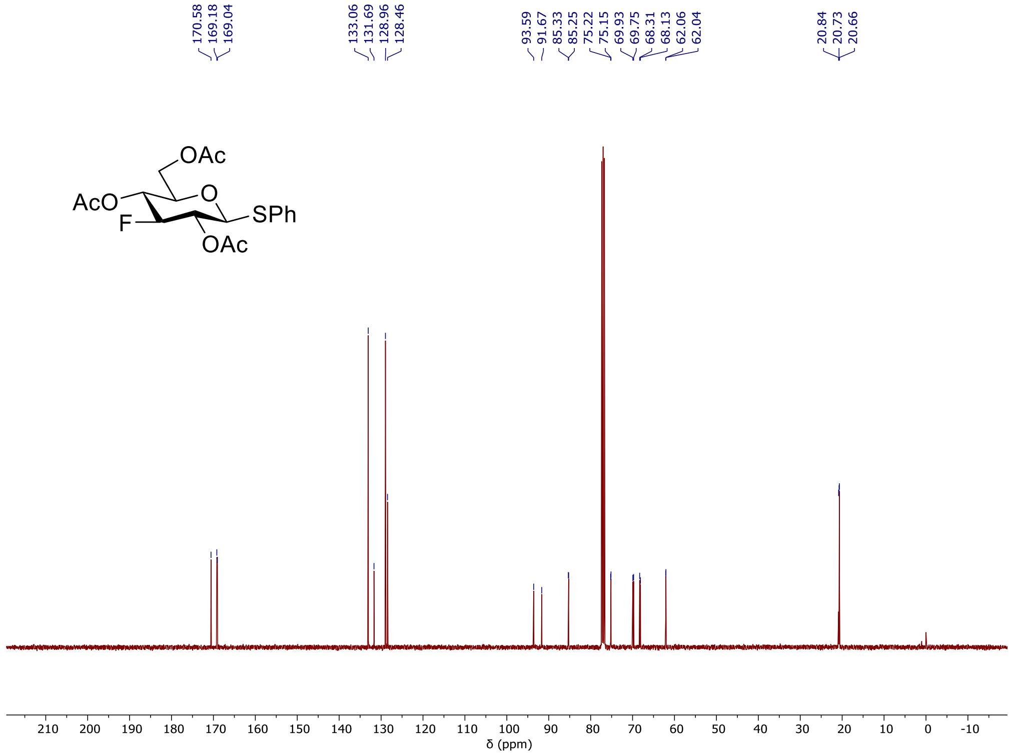

Phenyl 3-deoxy-3-fluoro-1-thio-β-D-glucopyranoside (S-5), ^{1}H NMR (400 MHz, MeOD)

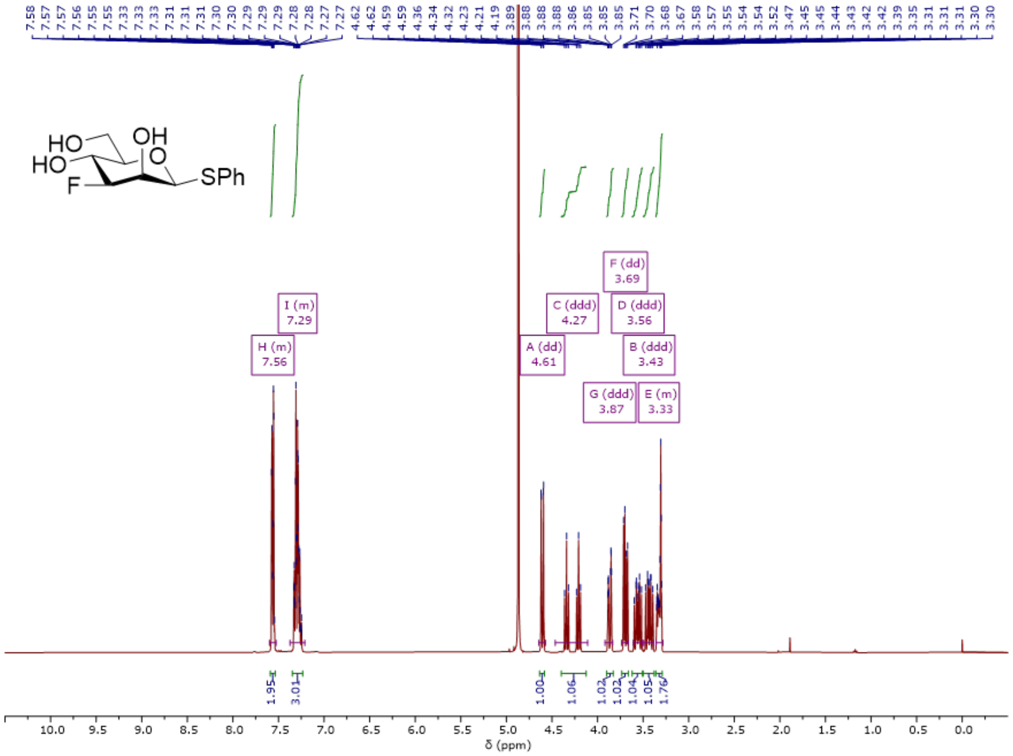

Phenyl 3-deoxy-3-fluoro-1-thio-β-D-glucopyranoside (S-5), ^{1}H NMR (400 MHz, MeOD)

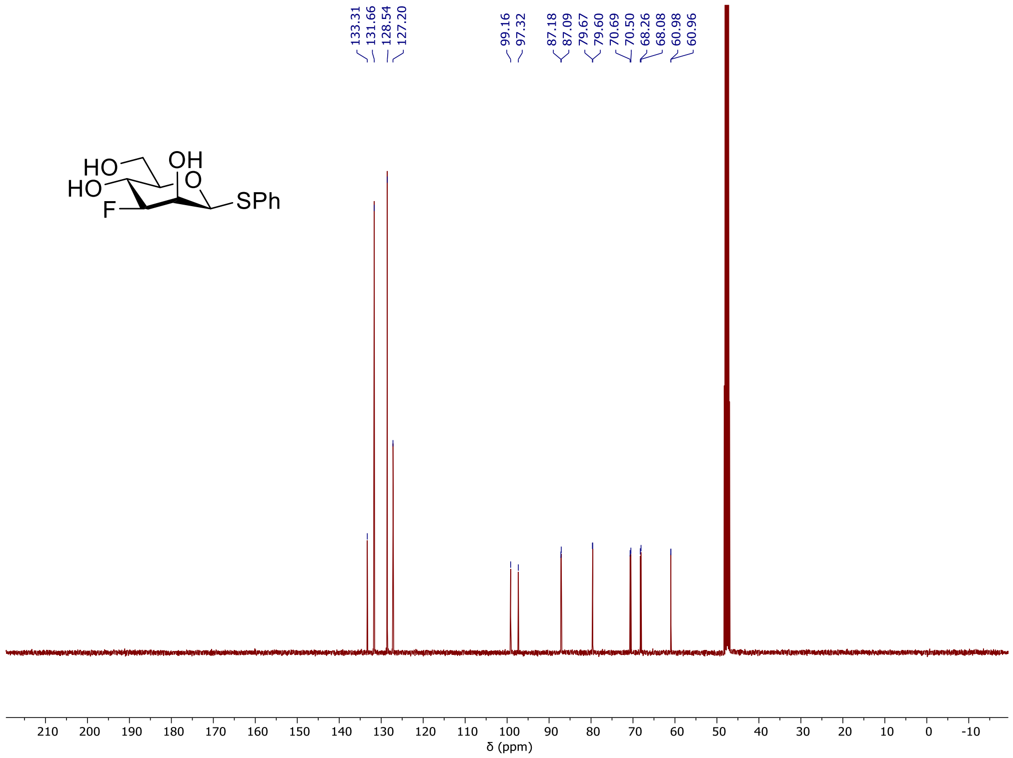

Phenyl 4,6-*O*-benzylidene-3-deoxy-3-fluoro-1-thio-β-D-glucopyranoside (S-6), ^{1}H NMR (400 MHz, $CDCl_3$)

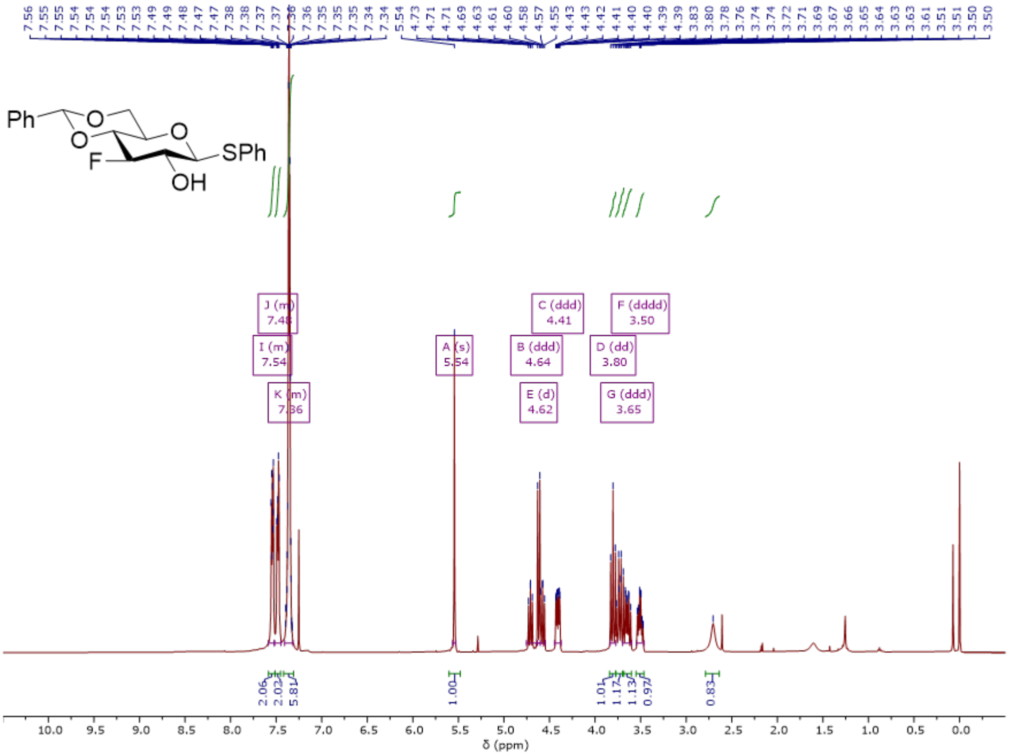

Phenyl 4,6-*O*-benzylidene-3-deoxy-3-fluoro-1-thio-β-D-glucopyranoside (S-6), ^{13}C NMR (101 MHz, $CDCl_3$)

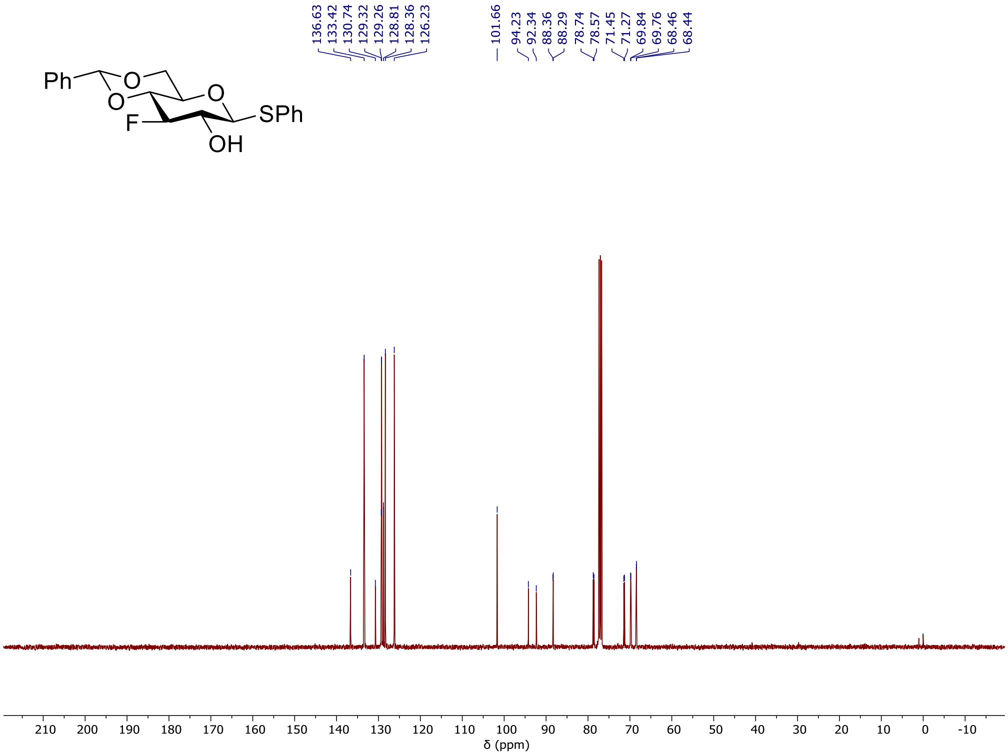

Phenyl 4,6-*O*-benzylidene-3-deoxy-3-fluoro-1-thio-β-D-mannopyranoside (S-7), ^{1}H NMR (400 MHz, CDCl$_3$)

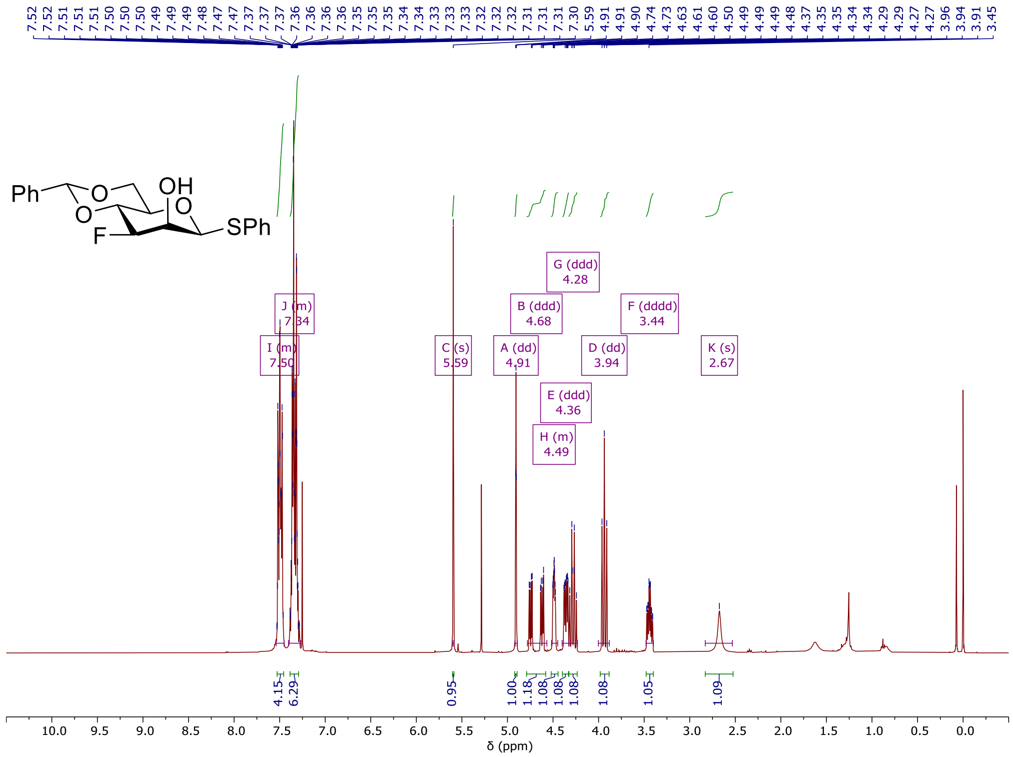

Phenyl 4,6-*O*-benzylidene-3-deoxy-3-fluoro-1-thio-β-D-mannopyranoside (S-7), ^{13}C NMR (101 MHz, CDCl$_3$)

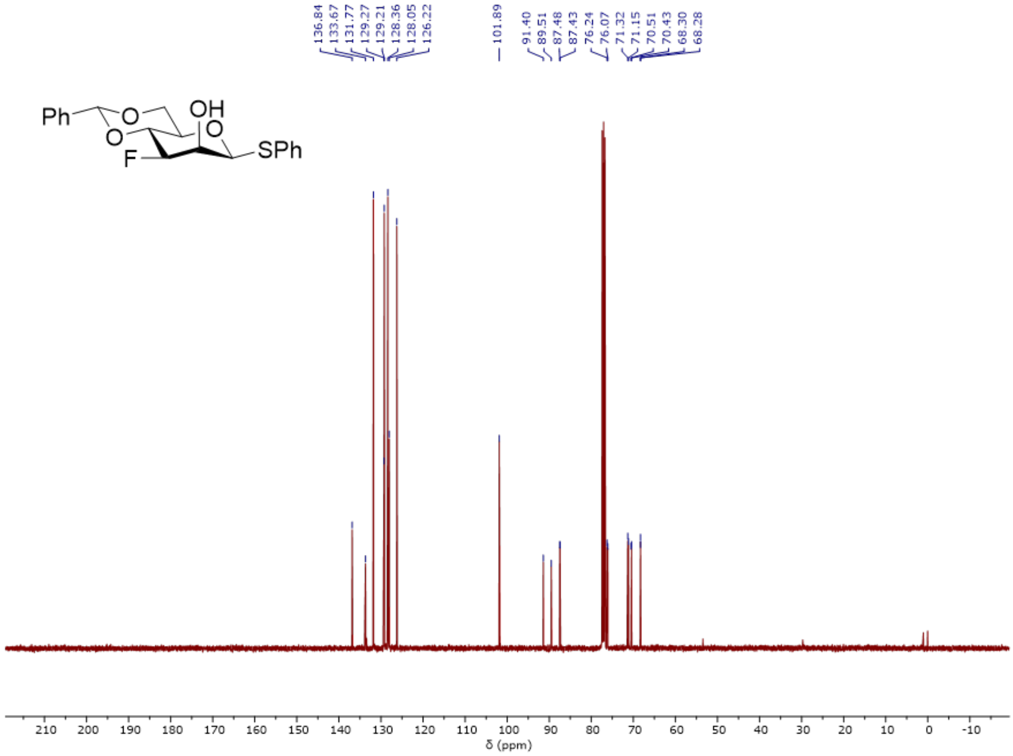

Phenyl 2-*O*-benzyl-4,6-*O*-benzylidene-3-deoxy-3-fluoro-1-thio-β-D-mannopyranoside (1), ^{1}H NMR (400 MHz, CDCl$_3$)

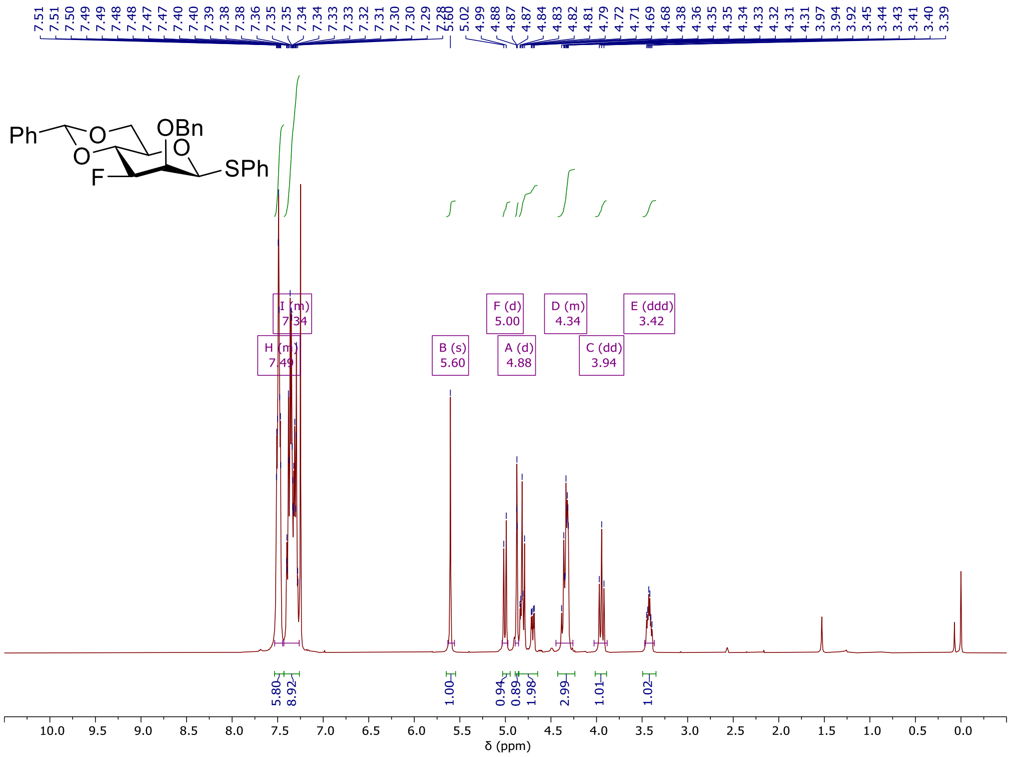

Phenyl 2-*O*-benzyl-4,6-*O*-benzylidene-3-deoxy-3-fluoro-1-thio-β-D-mannopyranoside (1), ^{13}C NMR (101 MHz, CDCl$_3$)

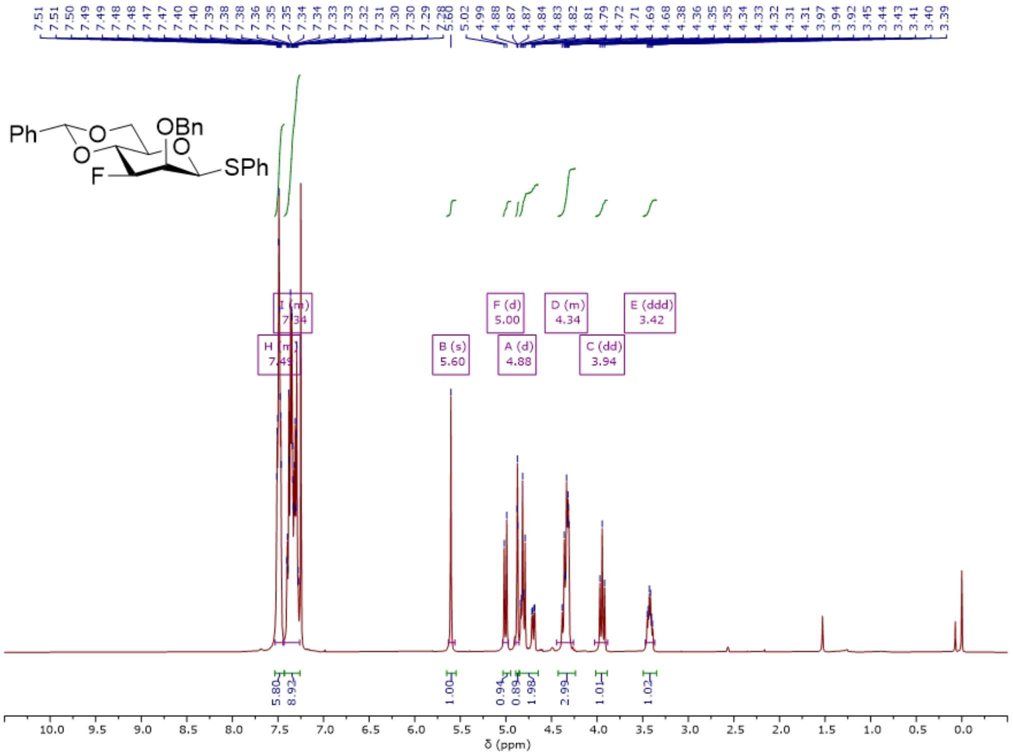

Phenyl 2-*O*-benzyl-3-deoxy-3-fluoro-1-thio-β-D-mannopyranoside (2), ^{1}H NMR (400 MHz, CDCl$_3$)

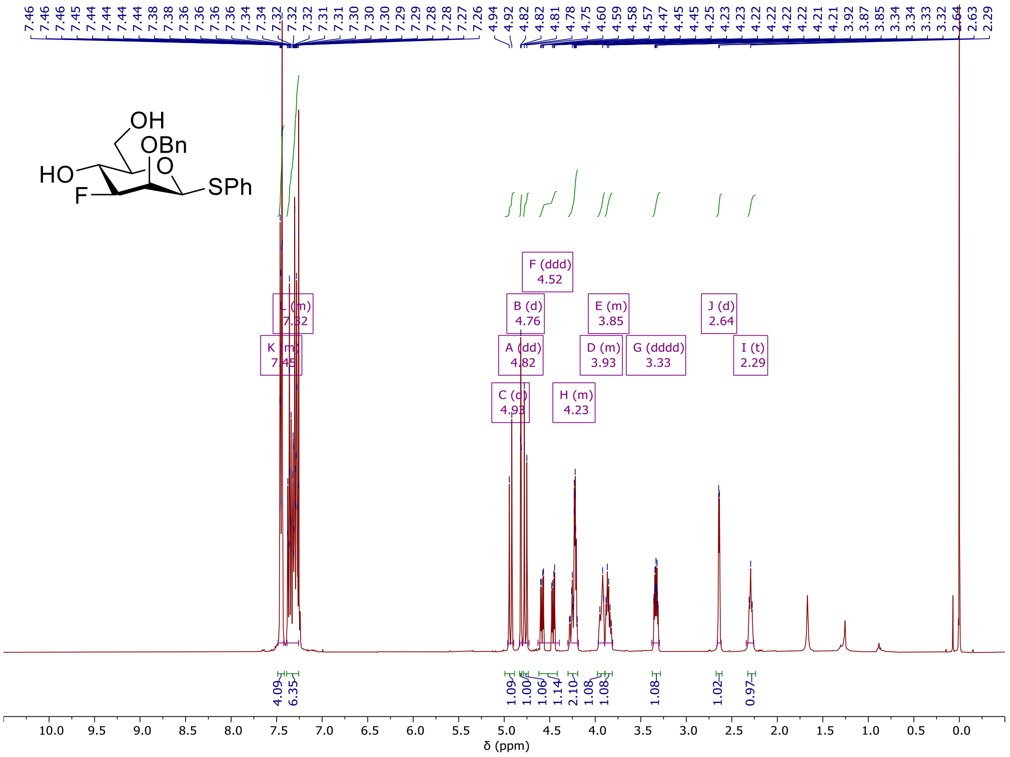

Phenyl 2-O-benzyl-3-deoxy-3-fluoro-1-thio-β-D-mannopyranoside (2), ^{13}C NMR (101 MHz, CDCl$_3$)

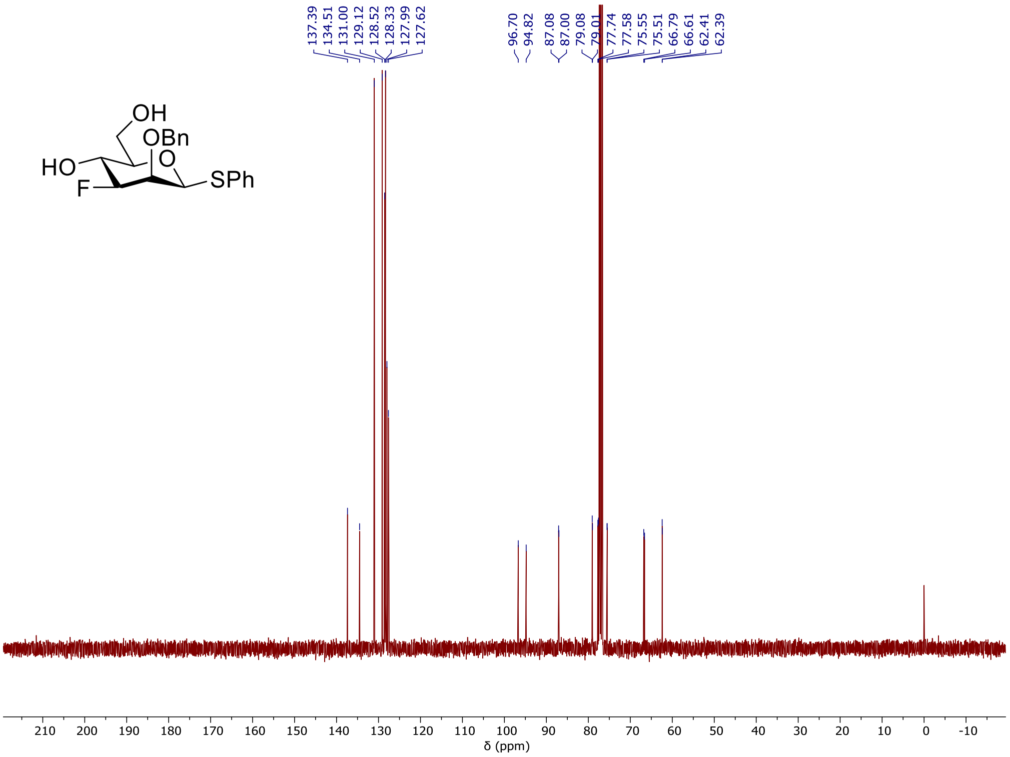

Methyl (phenyl 2-*O*-benzyl-3-deoxy-3-fluoro-1-thio-β-D-mannopyranoside) uronate (3), ^{1}H NMR (400 MHz, $CDCl_3$)

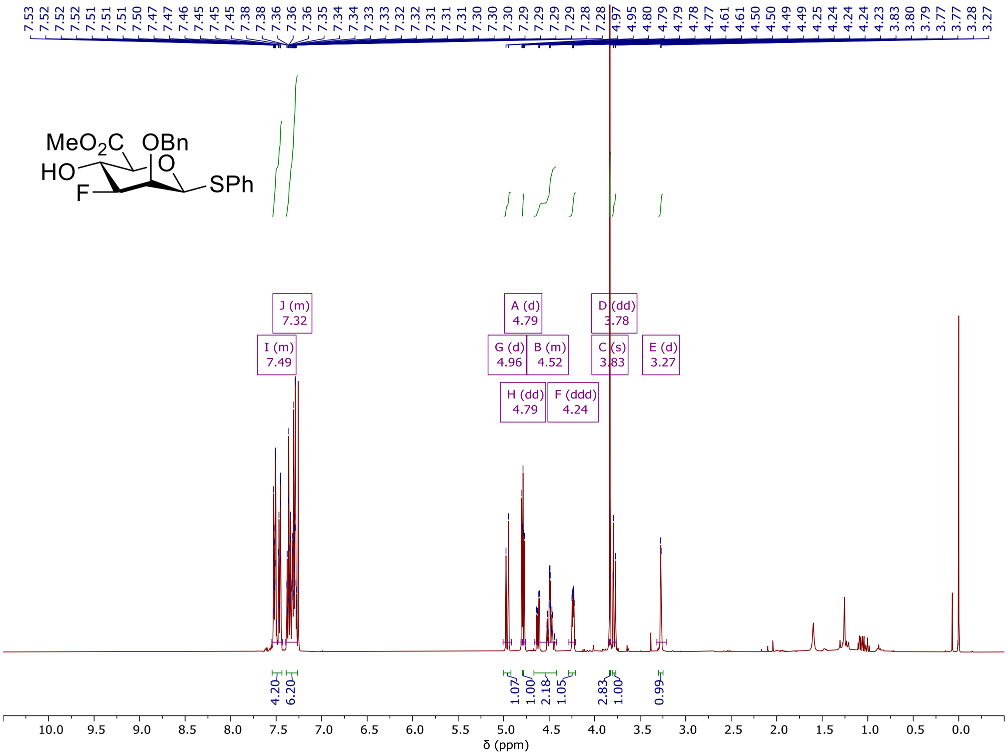

Methyl (phenyl 2-*O*-benzyl-3-deoxy-3-fluoro-1-thio-β-D-mannopyranoside) uronate (3), ^{13}C NMR (101 MHz, $CDCl_3$)

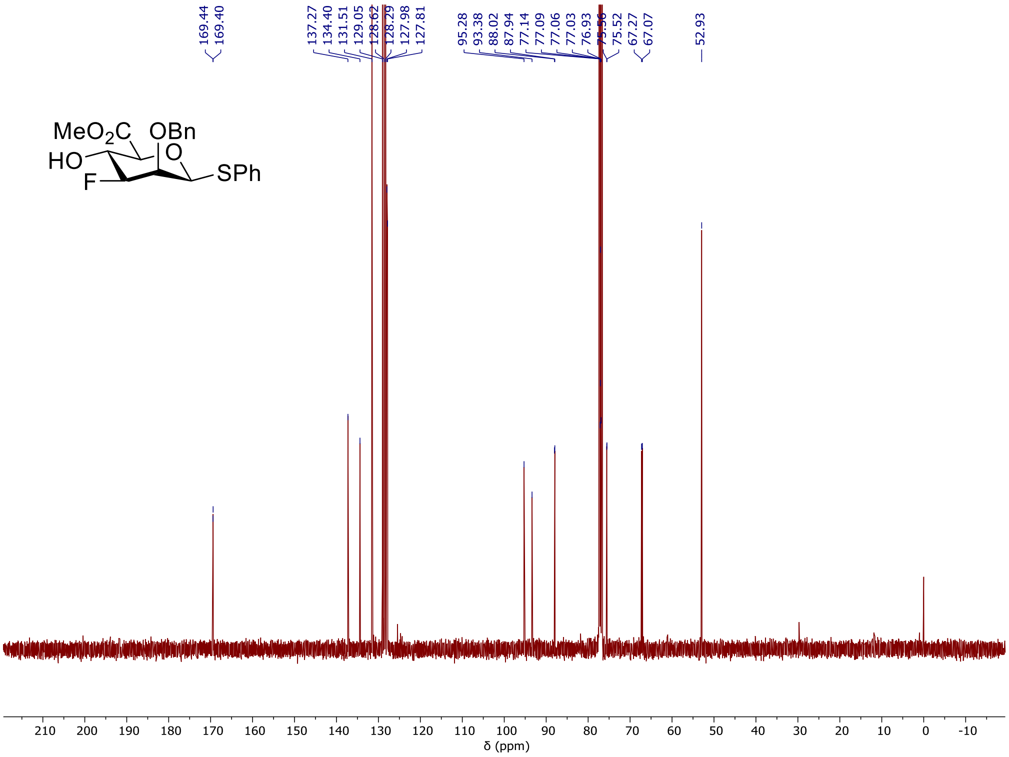

Methyl (phenyl 4-*O*-levulinoyl-2-*O*-benzyl-3-deoxy-3-fluoro-1-thio-β-D-mannopyranoside) uronate (4), ^{1}H NMR (400 MHz, CDCl3)

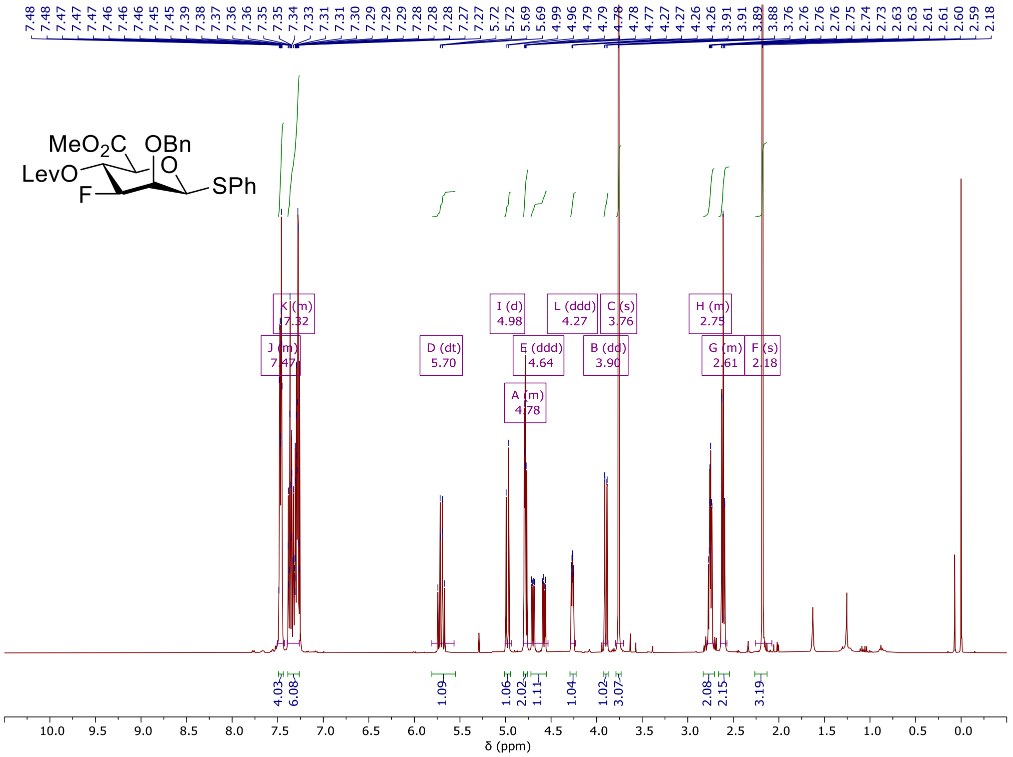

Methyl (phenyl 4-*O*-levulinoyl-2-*O*-benzyl-3-deoxy-3-fluoro-1-thio-β-D-mannopyranoside) uronate (4), ^{13}C NMR (101 MHz, CDCl3)

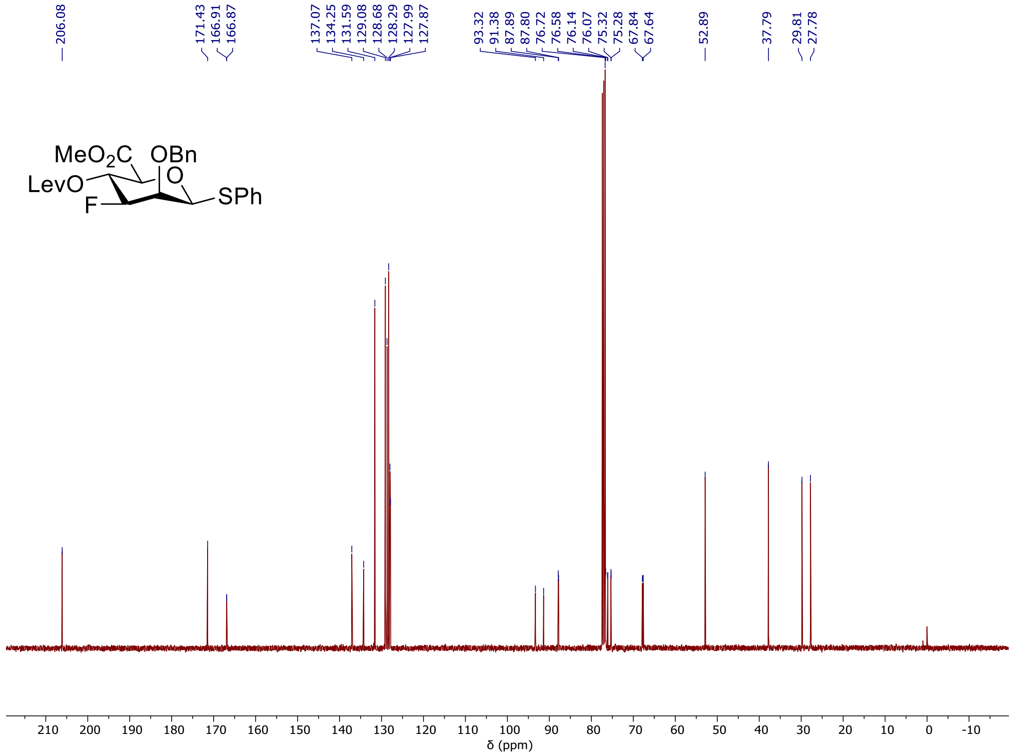

Methyl (phenyl 4-*O*-acetyl-2-*O*-benzyl-3-deoxy-3-fluoro 1-thio-β-D-mannopyranoside) uronate (7), ^{1}H NMR (400 MHz, CDCl$_3$)

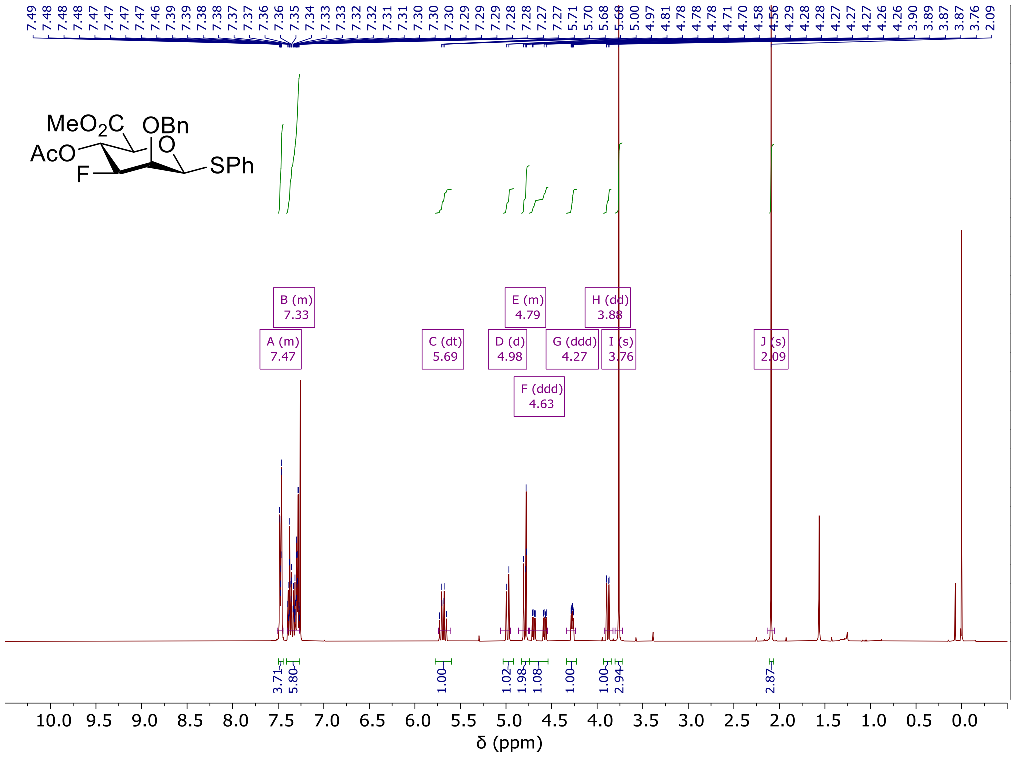

Methyl (phenyl 4-*O*-acetyl-2-*O*-benzyl-3-deoxy-3-fluoro-1-thio-β-D-mannopyranoside) uronate (7), ^{13}C NMR (400 MHz, CDCl$_3$)

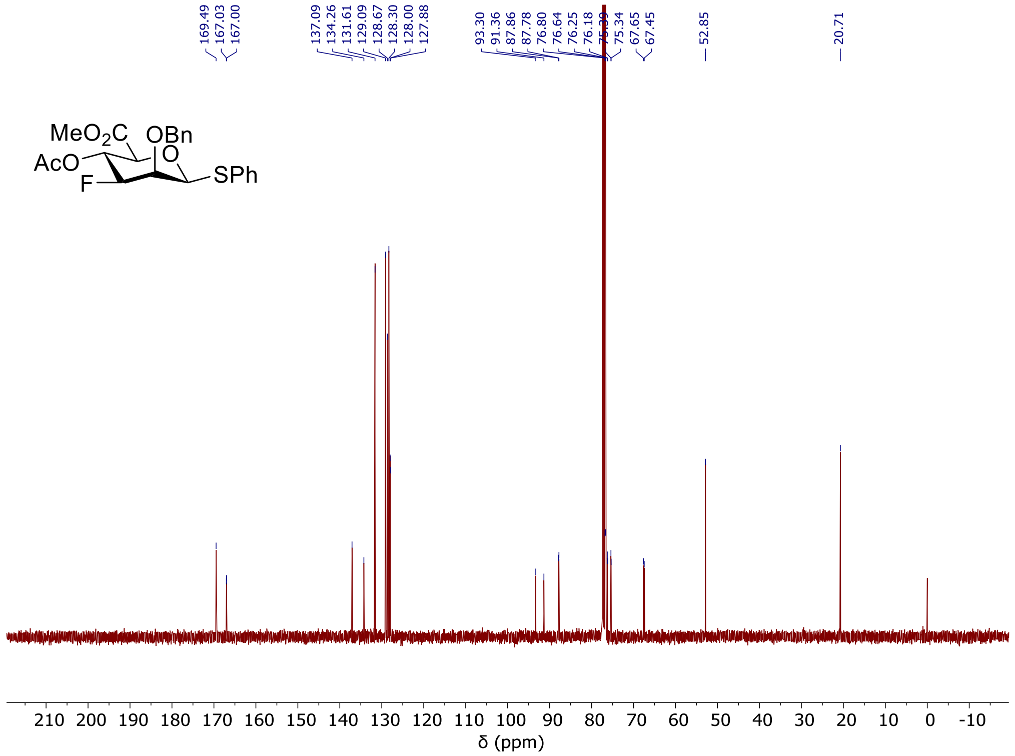

1,2,3,4,6-Penta-*O*-acetyl-α/β-D-mannopyranoside (S-8), ^{1}H NMR (400 MHz, CDCl3)

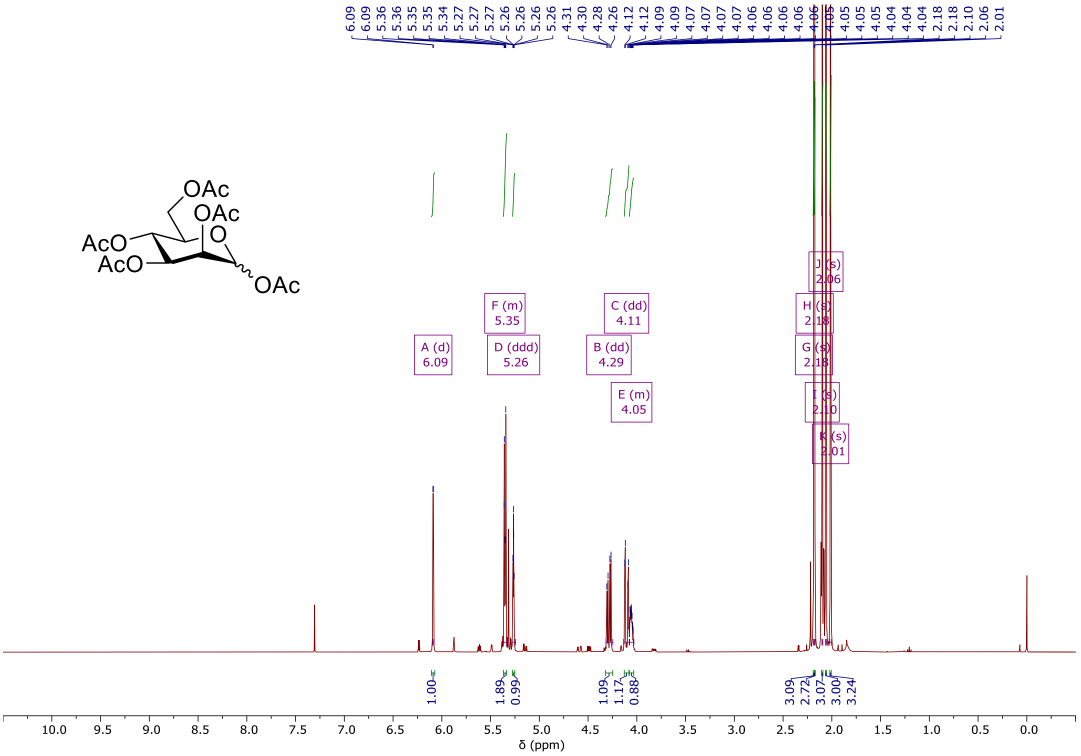

1,2,3,4,6-Penta-*O*-acetyl-α/β-D-mannopyranoside (S-8), ^{13}C NMR (101 MHz, CDCl3)

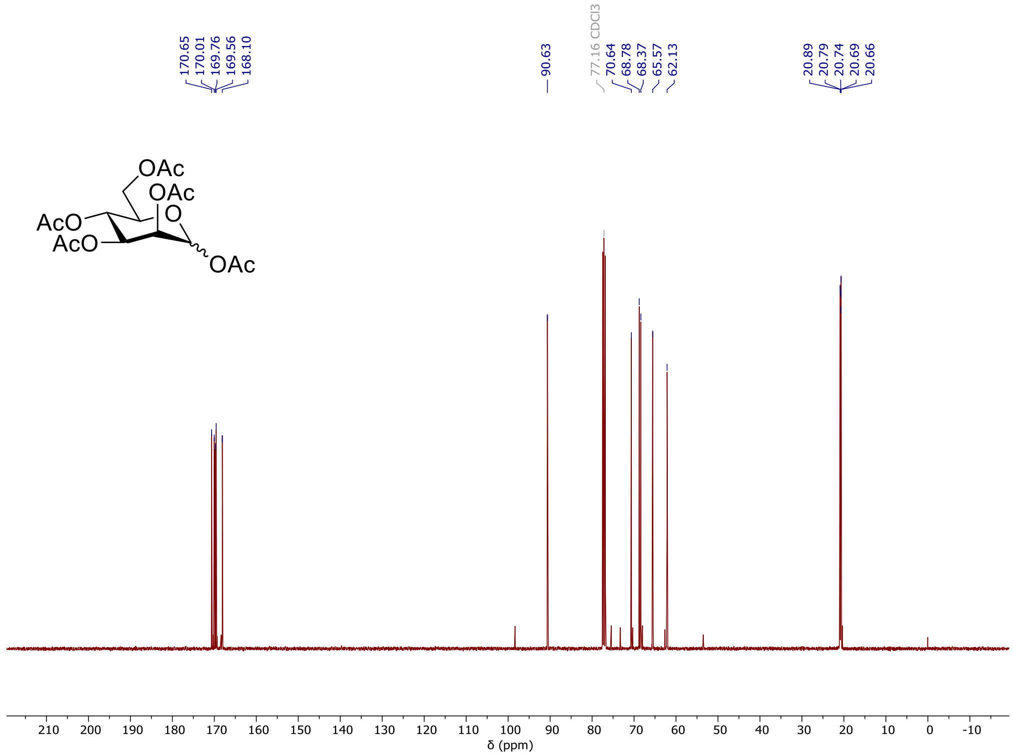

Phenyl 2,3,4,6-tetra-*O*-acetyl-1-thio-α-D-mannopyranoside (S-9), ^{1}H NMR (400 MHz, $CDCl_3$)

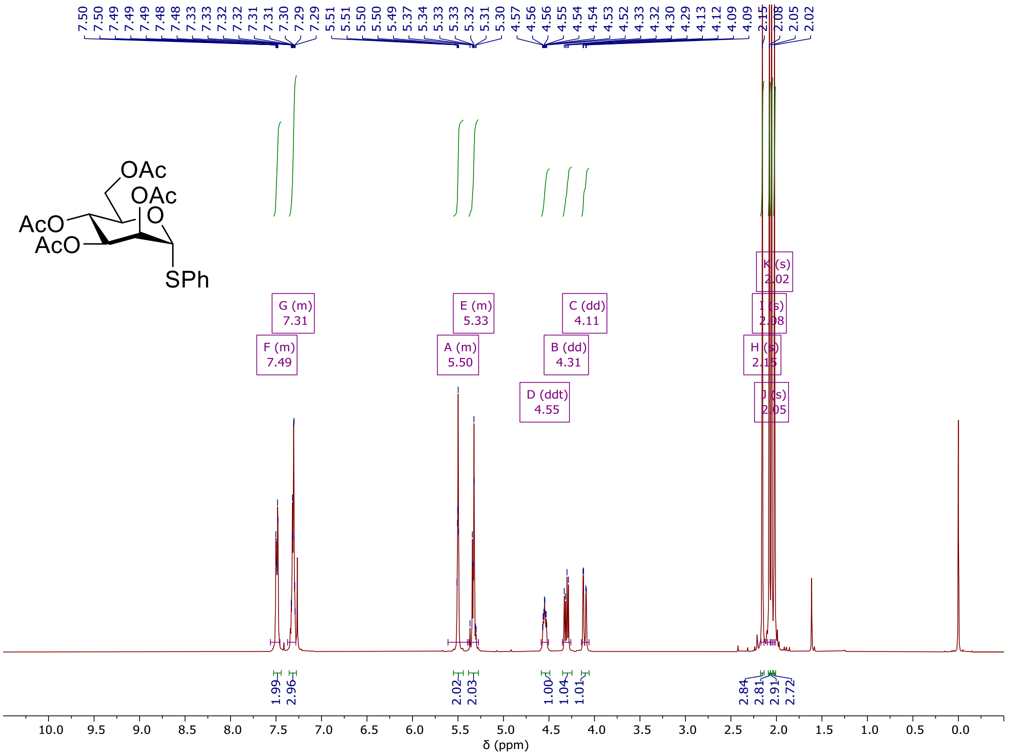

Phenyl 2,3,4,6-tetra-*O*-acetyl-1-thio-α-D-mannopyranoside (S-9), ^{13}C NMR (101 MHz, $CDCl_3$)

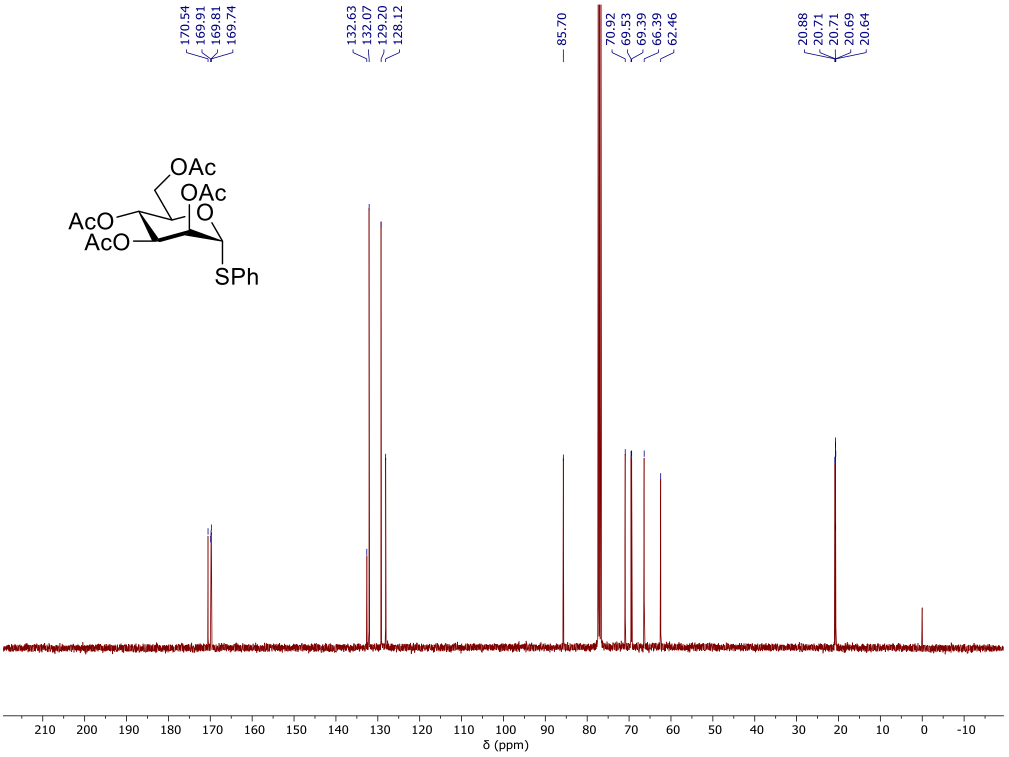

Phenyl 4,6-*O*-benzylidene-1-thio-α-D-mannopyranoside (S-11), ^{1}H NMR (400 MHz, $CDCl_3$)

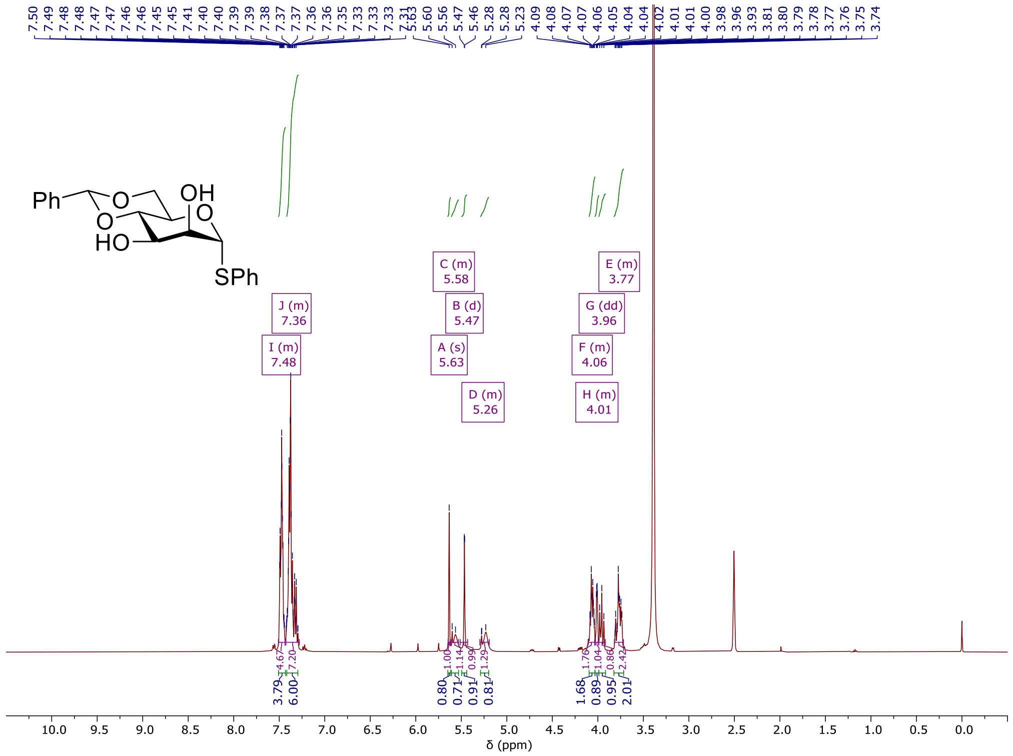

Phenyl 4,6-*O*-benzylidene-1-thio-α-D-mannopyranoside (S-11), ^{13}C NMR (101 MHz, $CDCl_3$)

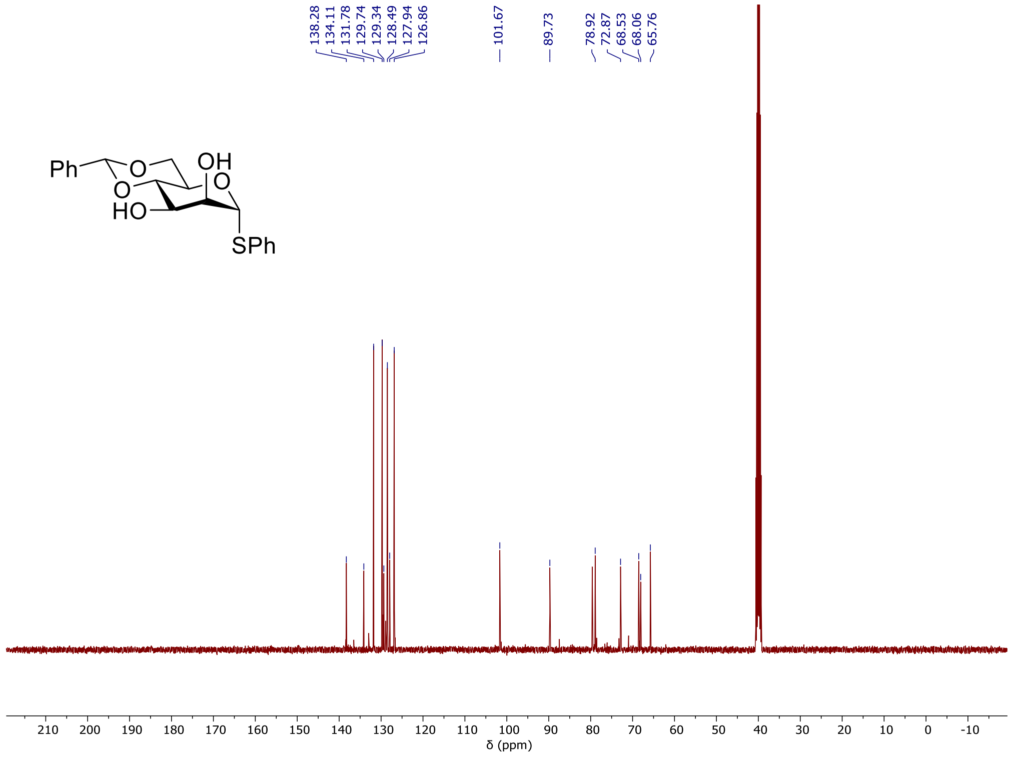

Phenyl 2,3-di-*O*-benzyl-4,6-*O*-benzylidene-1-thio-α-D-mannopyranoside (S-12), ^{1}H NMR (400 MHz, $CDCl_3$)

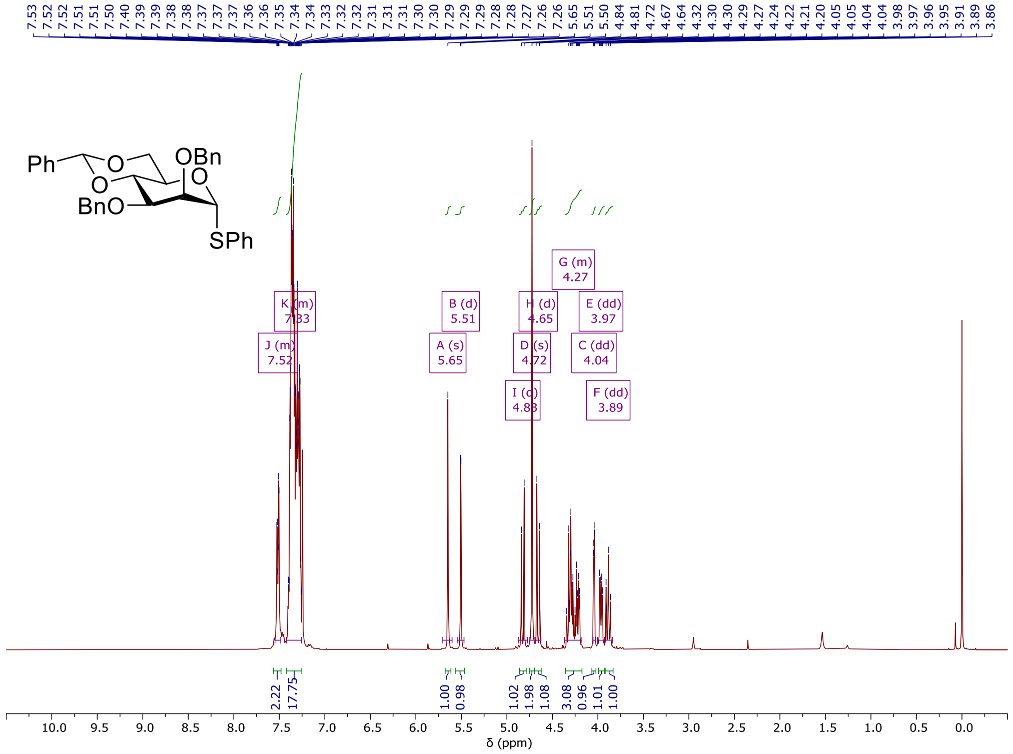

Phenyl 2,3-di-*O*-benzyl-4,6-*O*-benzylidene-1-thio-α-D-mannopyranoside (S-12), ^{13}C NMR (101 MHz, $CDCl_3$)

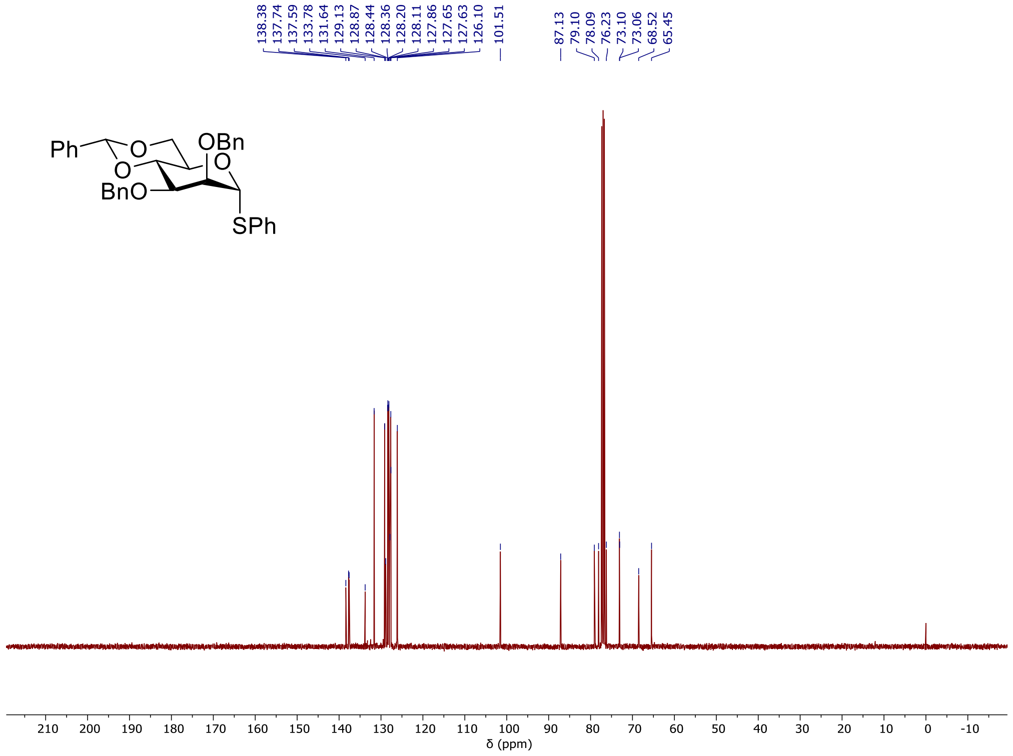

Phenyl 2,3-di-O-benzyl-1-thio-α-D-mannopyranoside (S-13), ^{1}H NMR (400 MHz, $CDCl_3$)

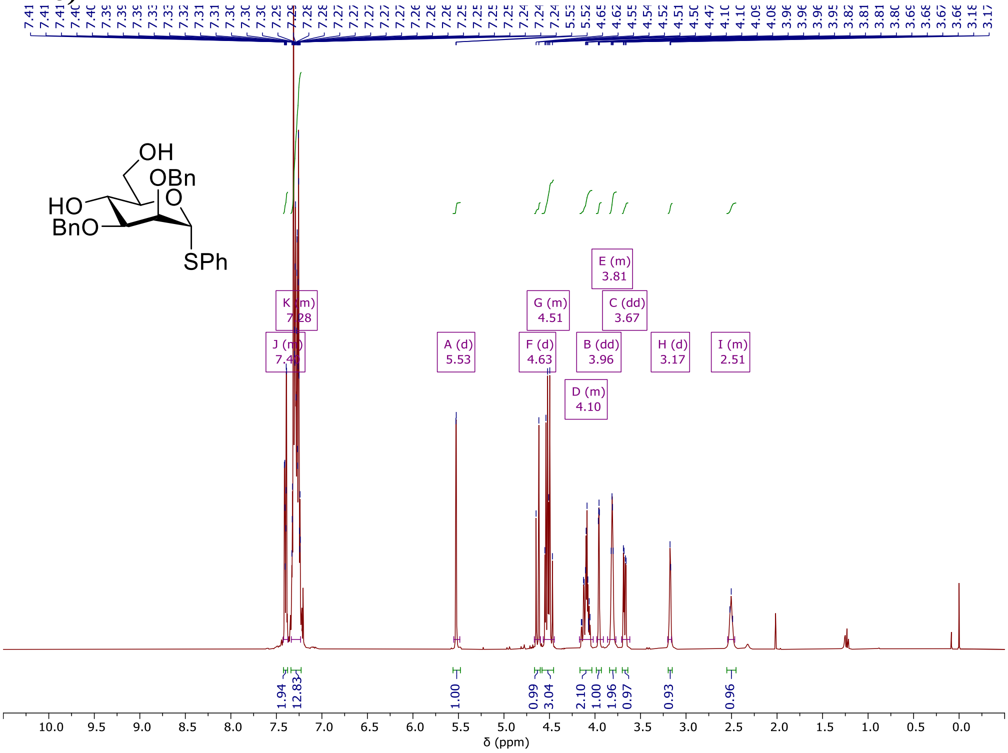

Phenyl 2,3-di-O-benzyl-1-thio-α-D-mannopyranoside (S-13), ^{13}C NMR (101 MHz, $CDCl_3$)

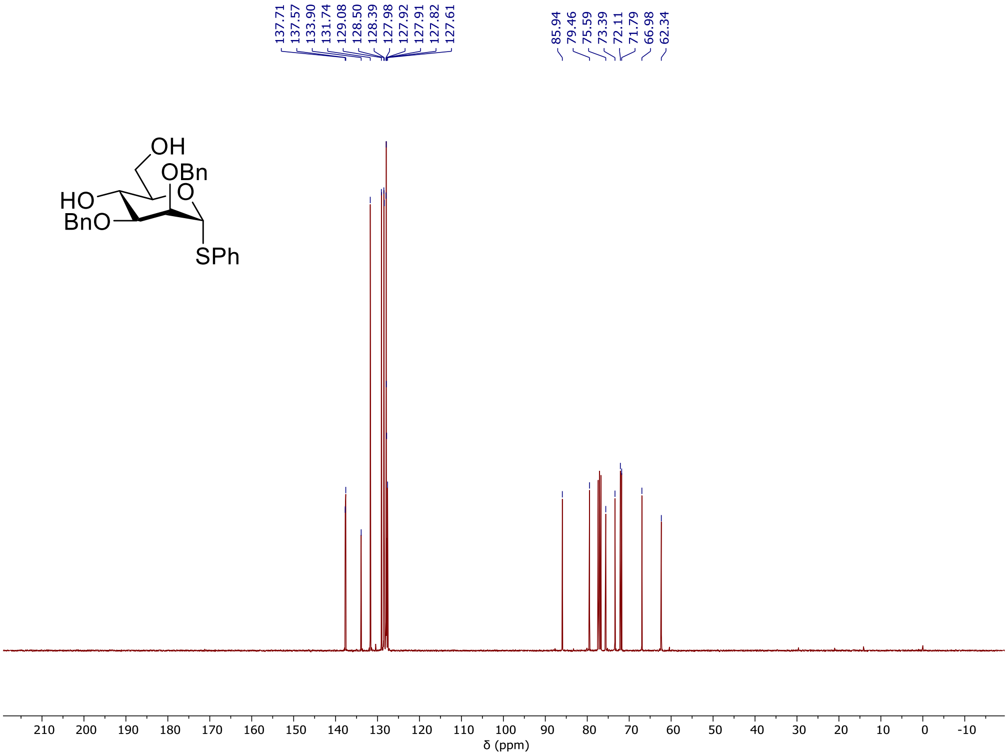

Methyl (phenyl-2,3-di-*O*-benzyl-1-thio-α-D-mannopyranoside) uronate (S-15), ^{1}H NMR (400 MHz, $CDCl_3$)

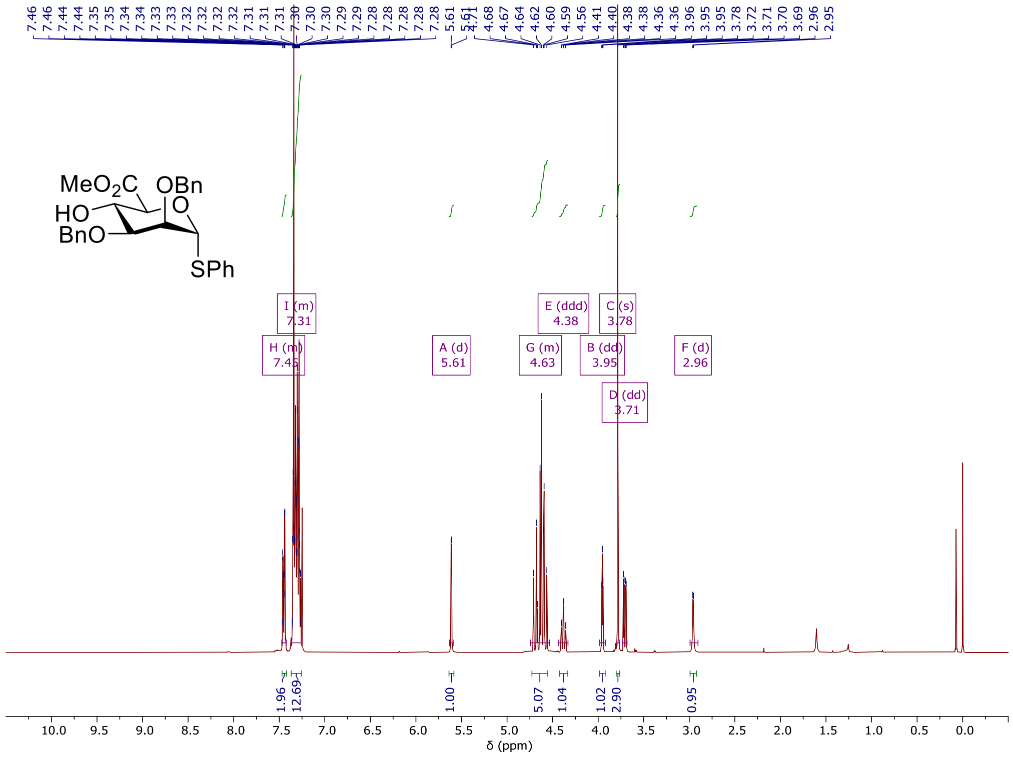

Methyl (phenyl-2,3-di-*O*-benzyl-1-thio-α-D-mannopyranoside) uronate (S-15), ^{13}C NMR (101 MHz, $CDCl_3$)

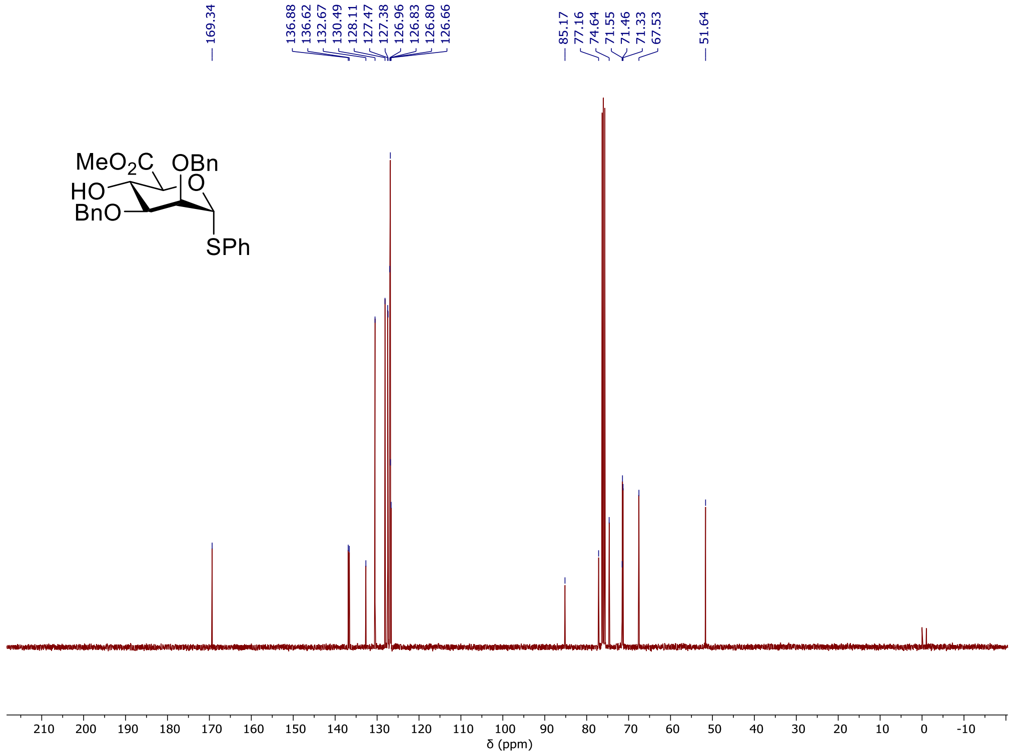

Methyl (phenyl-4-*O*-levulinoyl-2,3-di-*O*-benzyl-1-thio-α-D-mannopyranoside) uronate (6), ^{1}H NMR (400 MHz, $CDCl_3$)

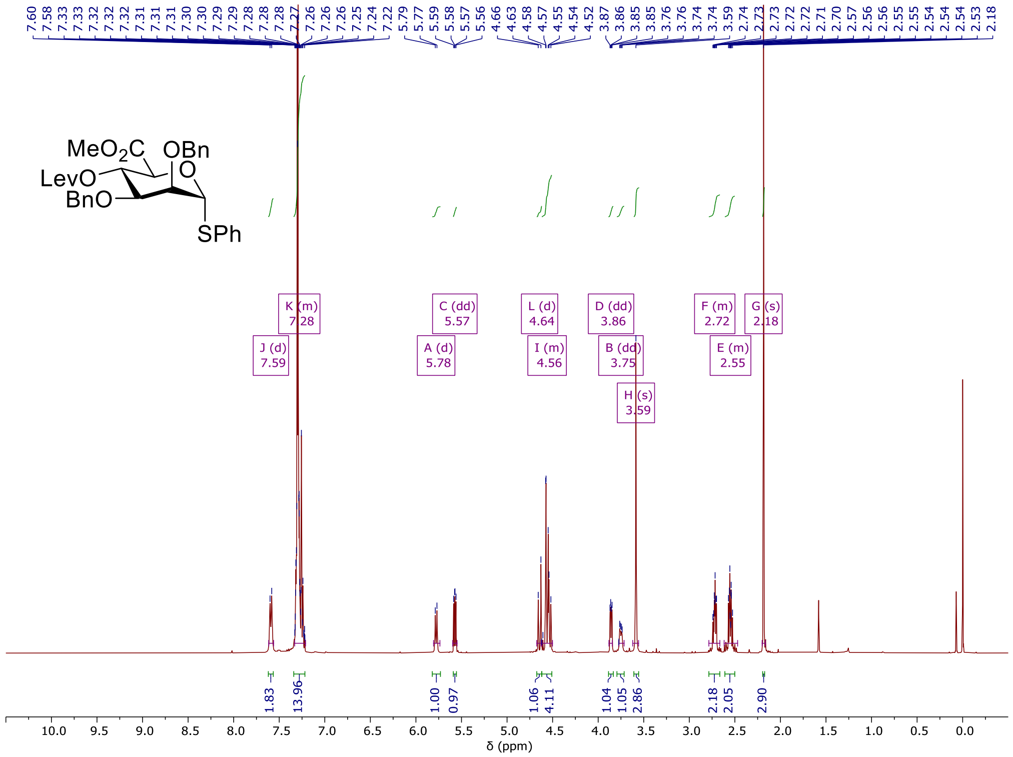

Methyl (phenyl-4-*O*-levulinoyl-2,3-di-*O*-benzyl-1-thio-α-D-mannopyranoside) uronate (6), ^{13}C NMR (101 MHz, $CDCl_3$)

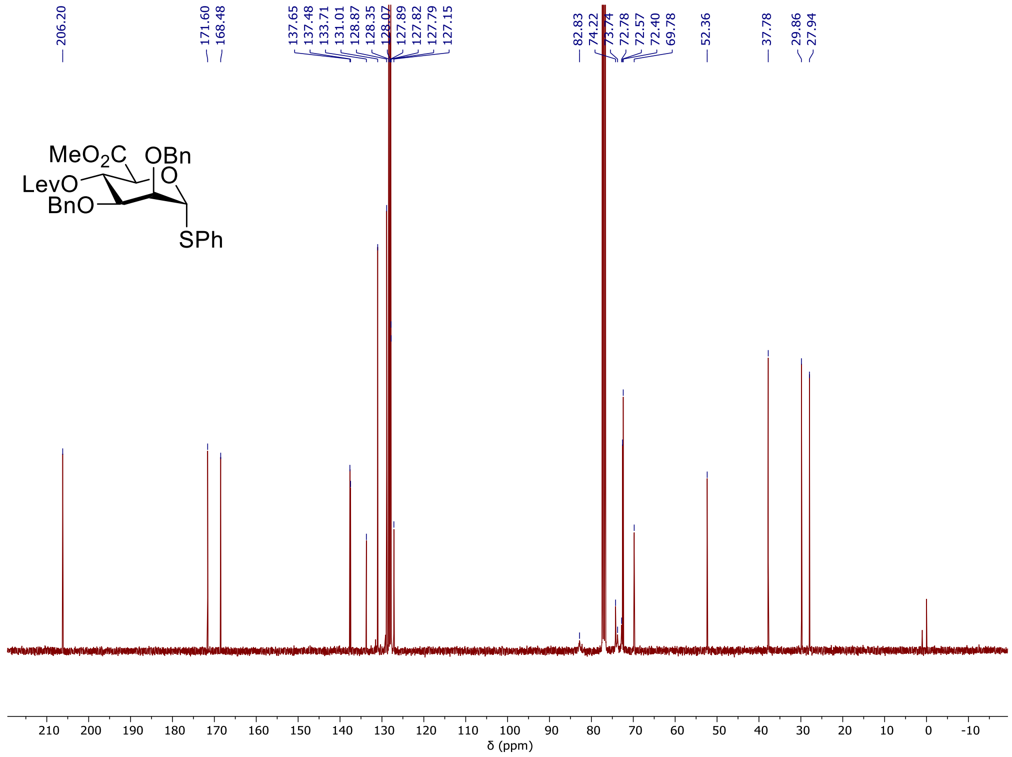

Methyl (4-*O*-levulinoyl-2,3-di-*O*-benzyl-α/β-D-mannopyranoside) uronate (S-16), ¹H NMR (400 MHz, CDCl₃)

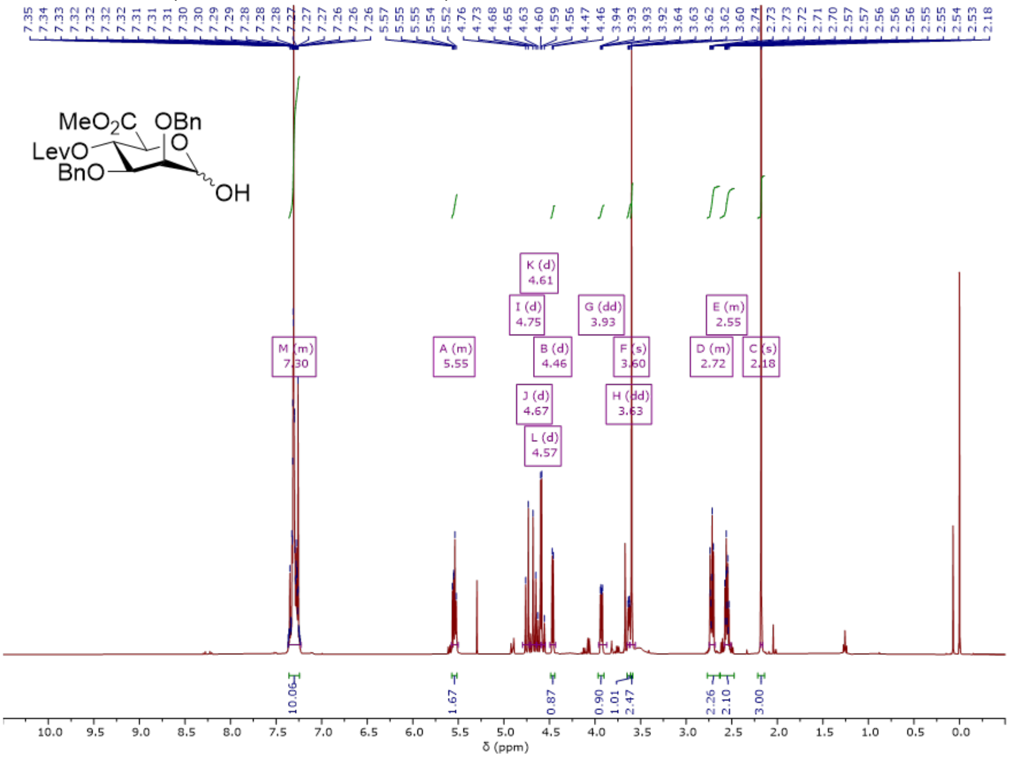

Methyl (4-*O*-levulinoyl-2,3-di-*O*-benzyl-α/β-D-mannopyranoside) uronate (S-16), ¹³C NMR (101 MHz, CDCl₃)

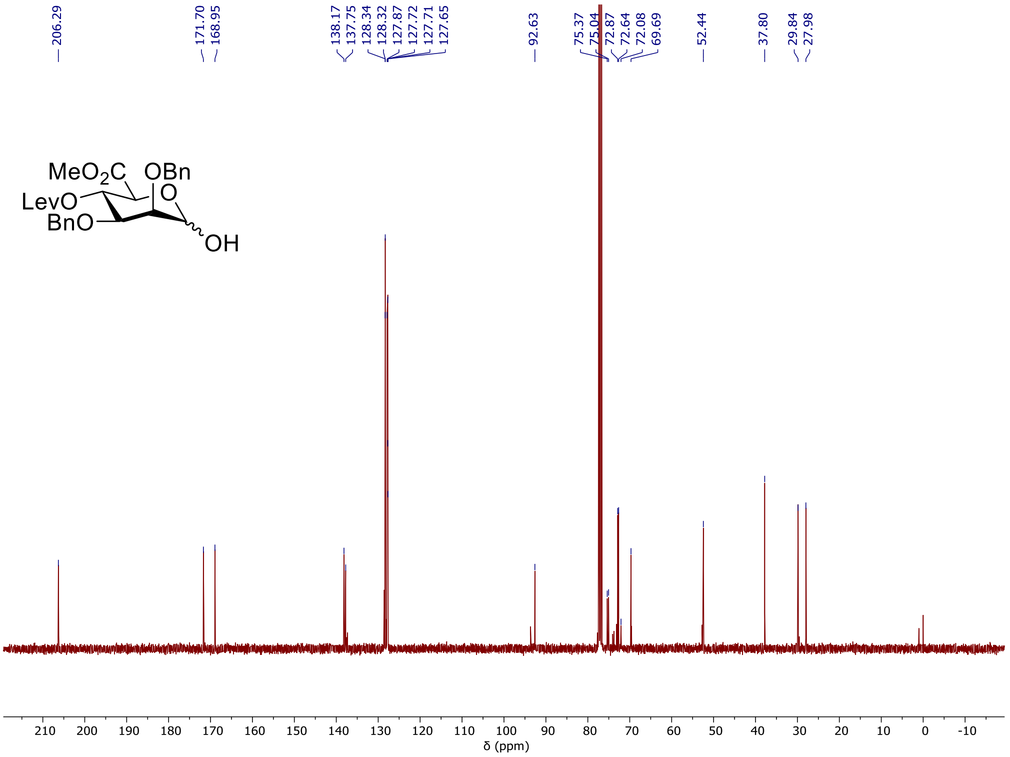

Methyl (4-*O*-levulinoyl-2,3-di-*O*-benzyl-1-*O*-(*N*-[phenyl]trifluoroacetimidoyl)-α/β-D-mannopyranoside) uronate (S-17), ^{1}H NMR (400 MHz, CDCl$_3$)

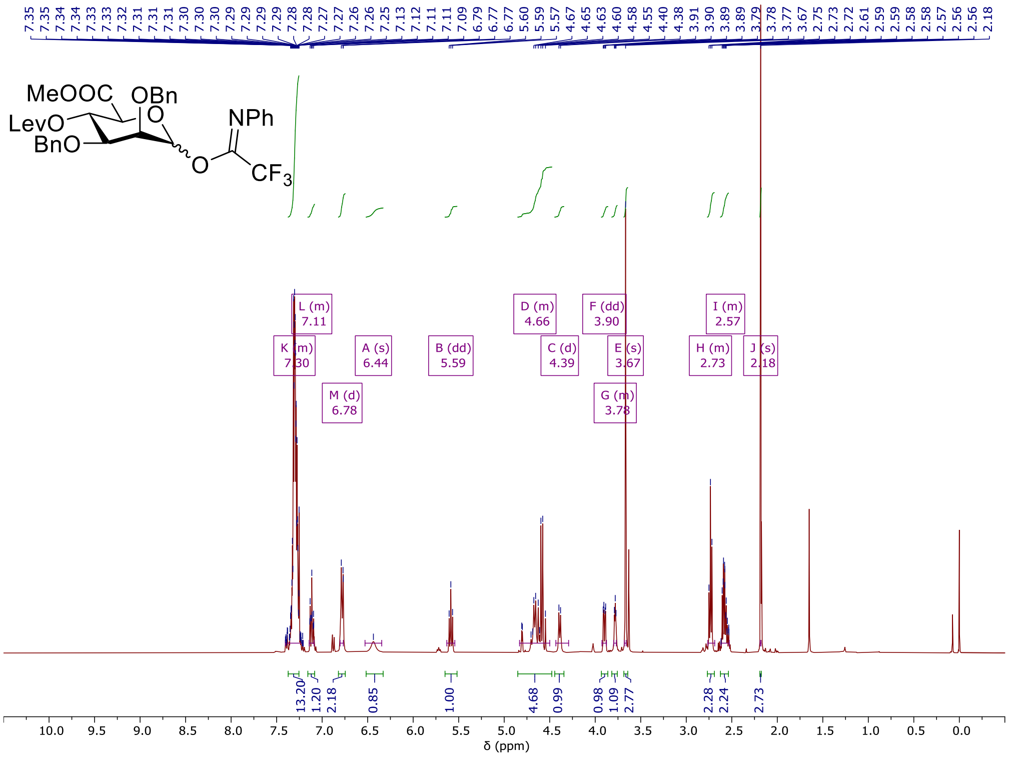

Methyl (4-*O*-levulinoyl-2,3-di-*O*-benzyl-1-*O*-(*N*-[phenyl]trifluoroacetimidoyl)-α/β-D-mannopyranoside) uronate (S-17), ^{13}C NMR (101 MHz, CDCl$_3$)

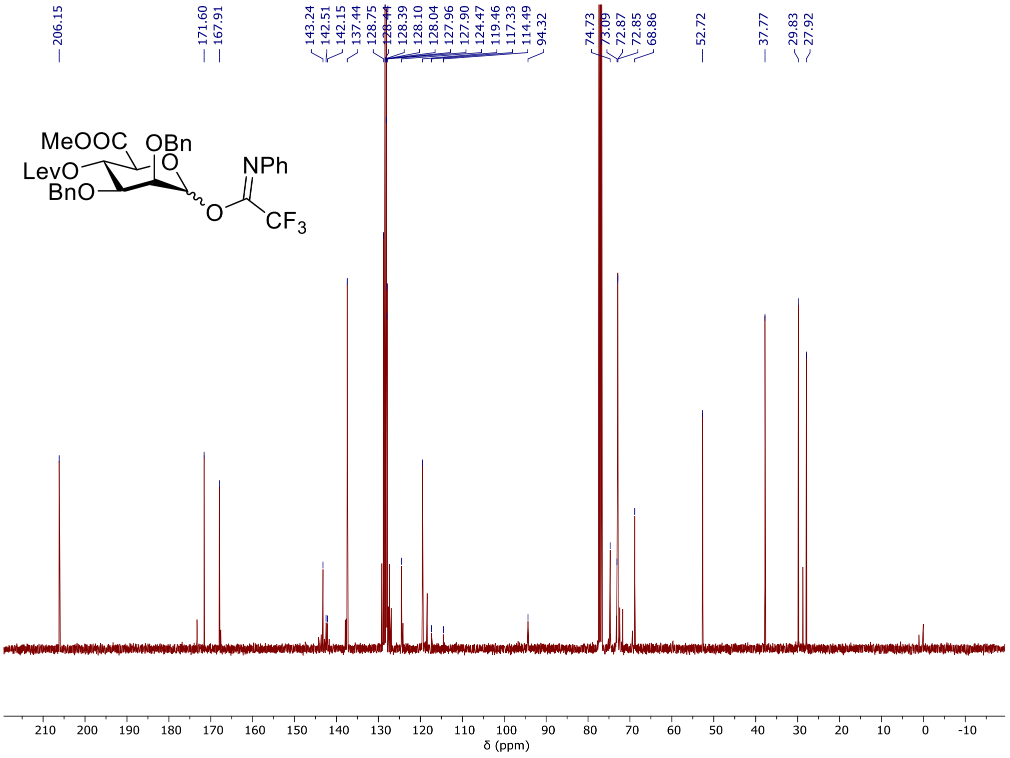

Methyl (4-*O*-levulinoyl-2-*O*-benzyl-3-deoxy-3-fluoro-α/β-D-mannopyranoside) uronate (S-18), ^{1}H NMR (400 MHz, $CDCl_3$)

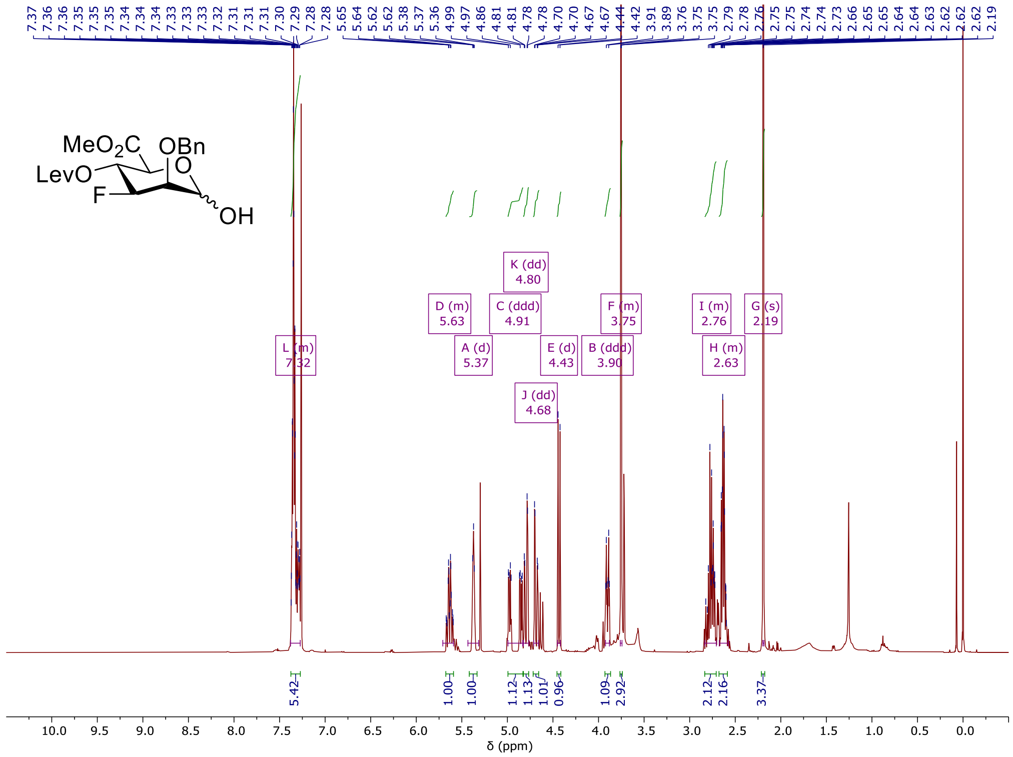

Methyl (4-*O*-levulinoyl-2-*O*-benzyl-3-deoxy-3-fluoro-α/β-D-mannopyranoside) uronate (S-18), ^{13}C NMR (101 MHz, $CDCl_3$)

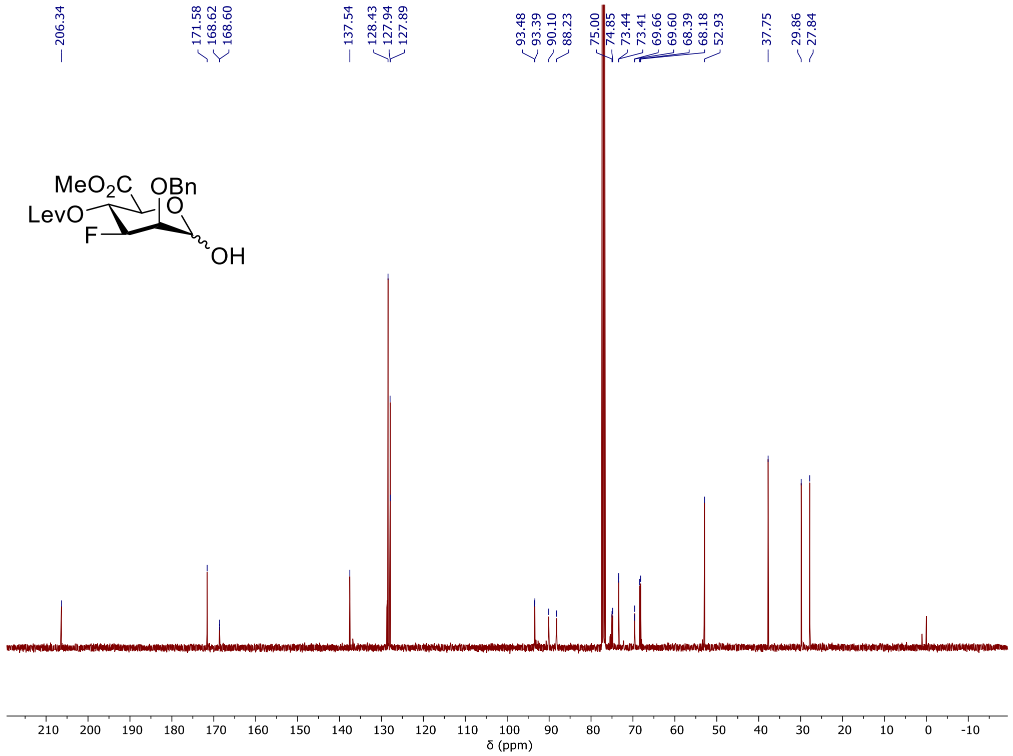

Methyl (4-*O*-levulinoyl-2-*O*-benzyl-3-deoxy-3-fluoro-1-*O*-(*N*-[phenyl]trifluoroacetimidoyl)-α-D-mannopyranoside) uronate (S-19), ^{1}H NMR (400 MHz, CDCl$_3$)

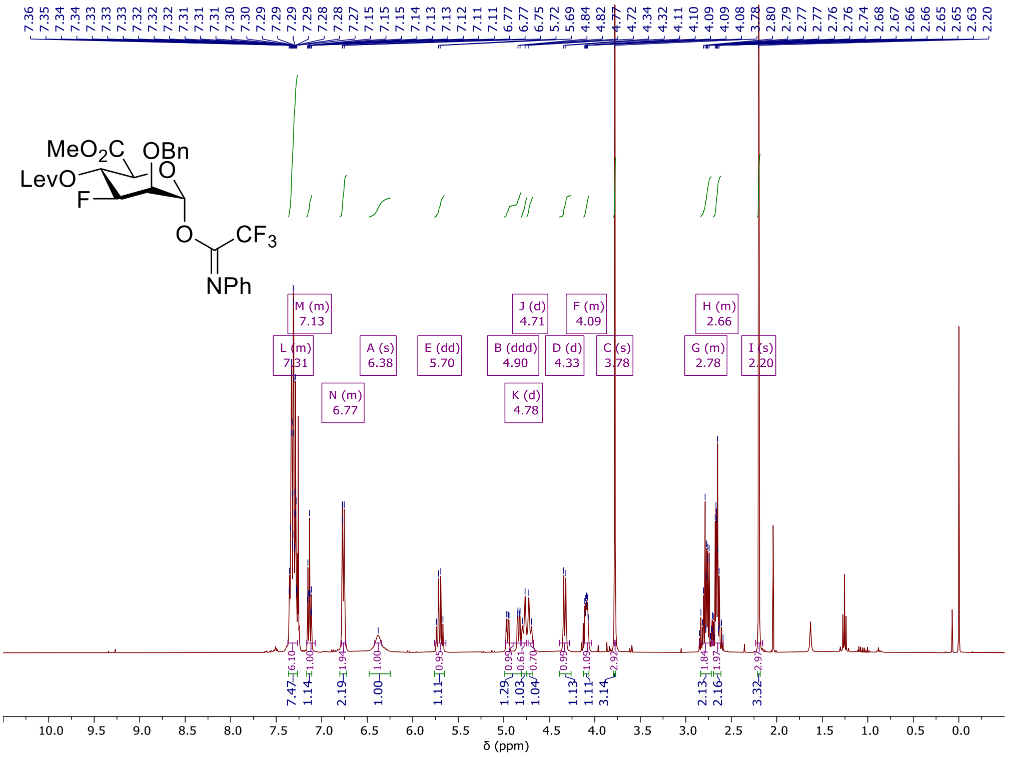

Methyl (4-*O*-levulinoyl-2-*O*-benzyl-3-deoxy-3-fluoro-1-*O*-(*N*-[phenyl]trifluoroacetimidoyl)-α-D-mannopyranoside) uronate (S-19), ^{13}C NMR (101 MHz, CDCl$_3$)

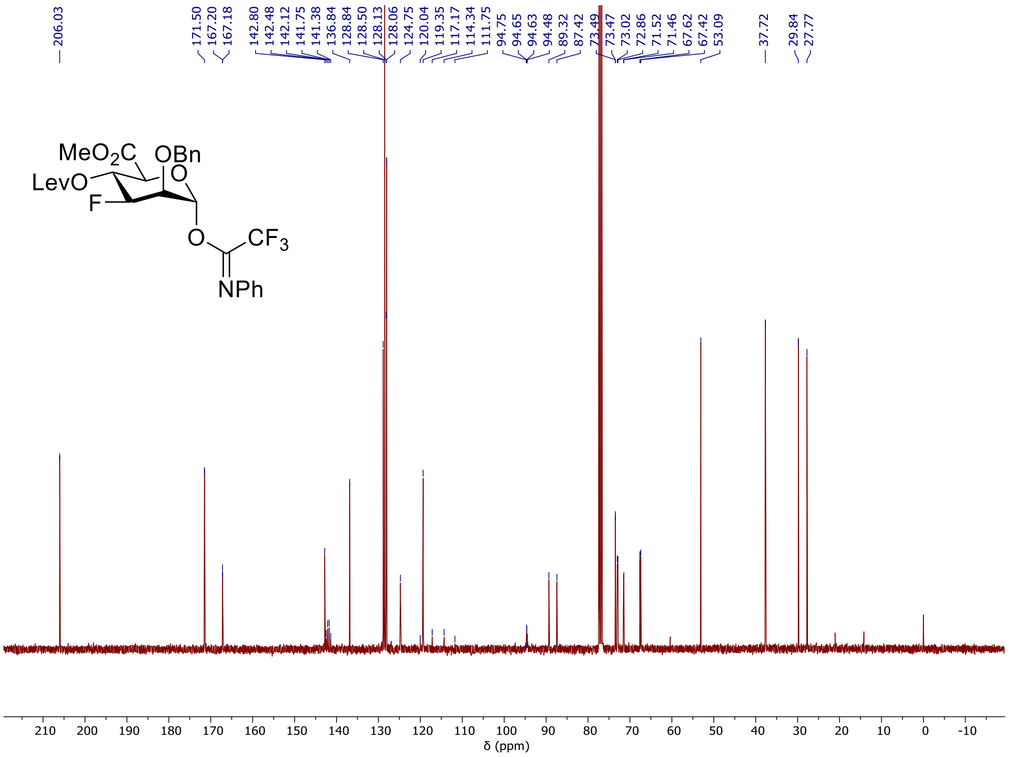

Methyl (ethyl 4-O-acetyl-2-O-benzyl-2-deoxy-2-fluoro-α/β-ᴅ-mannopyranosyl uronate) (7A), ^{1}H NMR (850 MHz, $CDCl_3$)

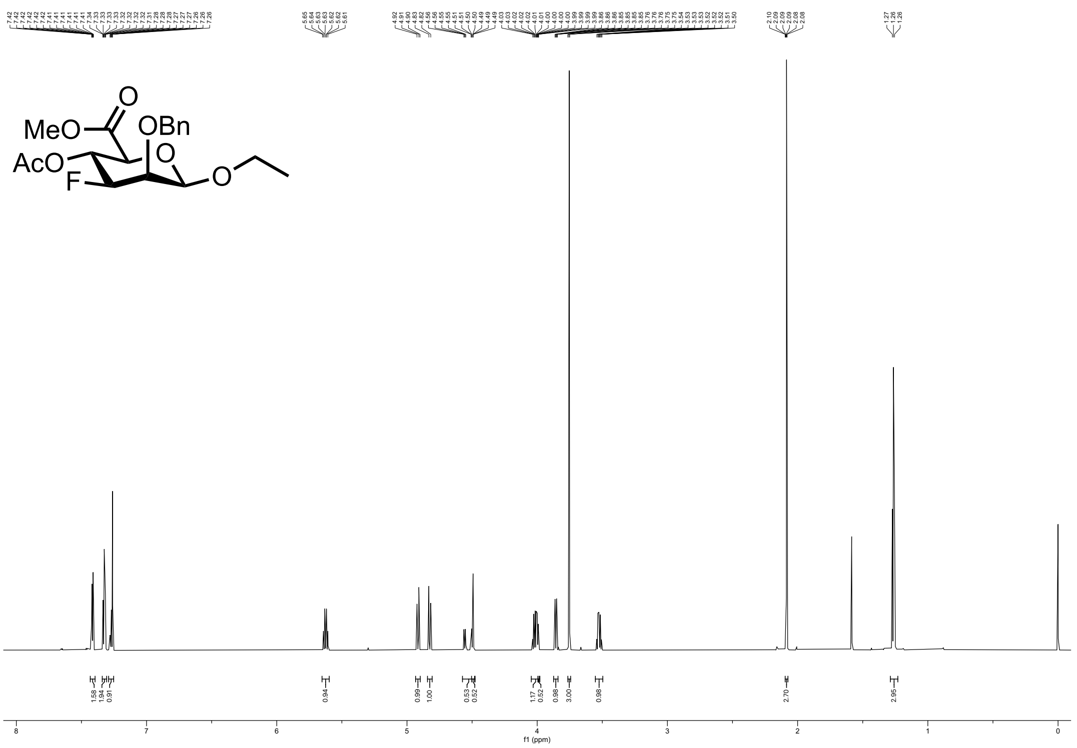

Methyl (ethyl 4-O-acetyl-2-O-benzyl-2-deoxy-2-fluoro-α/β-ᴅ-mannopyranosyl uronate) (7A), ^{13}C NMR (214 MHz, $CDCl_3$)

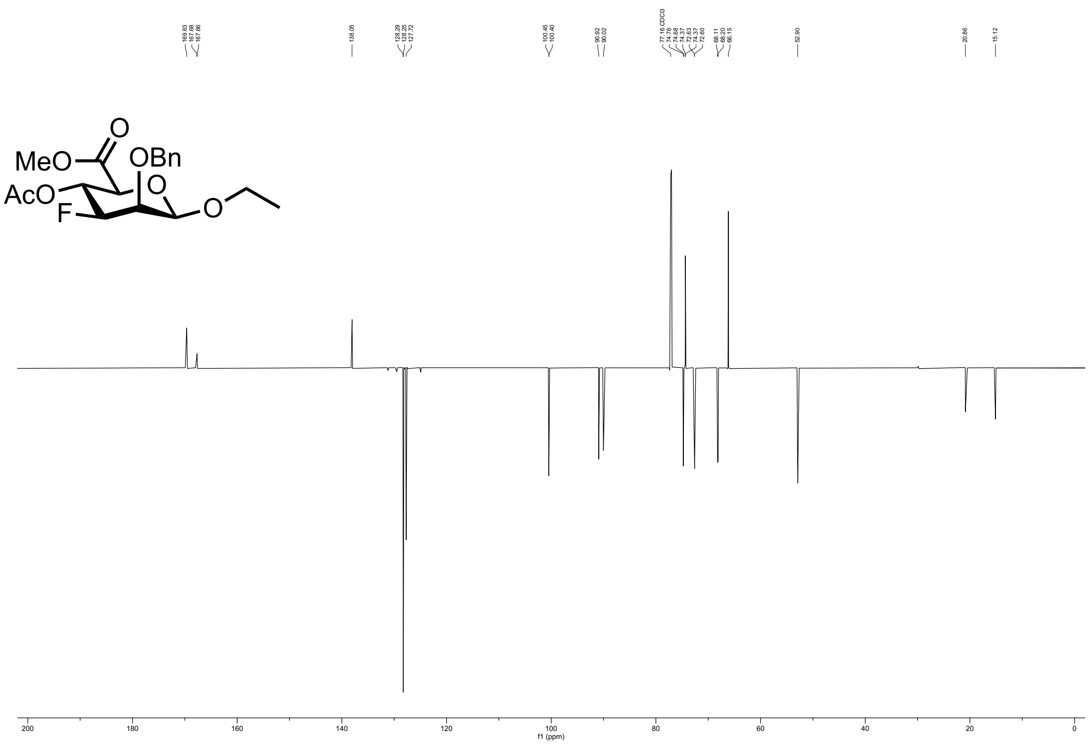

Methyl (2-monofluoroethyl 4-*O*-acetyl-2-*O*-benzyl-2-deoxy-2-fluoro-α/β-D-mannopyranosyl uronate) (7B), ^{1}H NMR (850 MHz, $CDCl_3$)

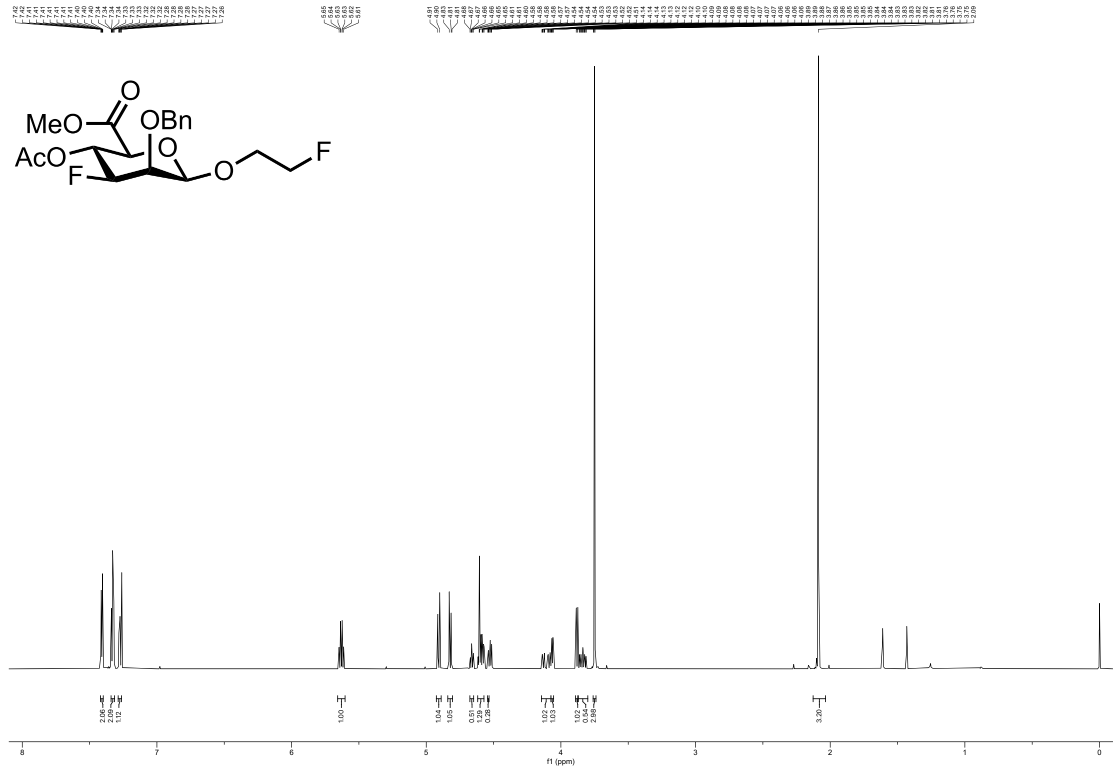

Methyl (2-monofluoroethyl 4-*O*-acetyl-2-*O*-benzyl-2-deoxy-2-fluoro-α/β-D-mannopyranosyl uronate) (7B), ^{13}C NMR (214 MHz, $CDCl_3$)

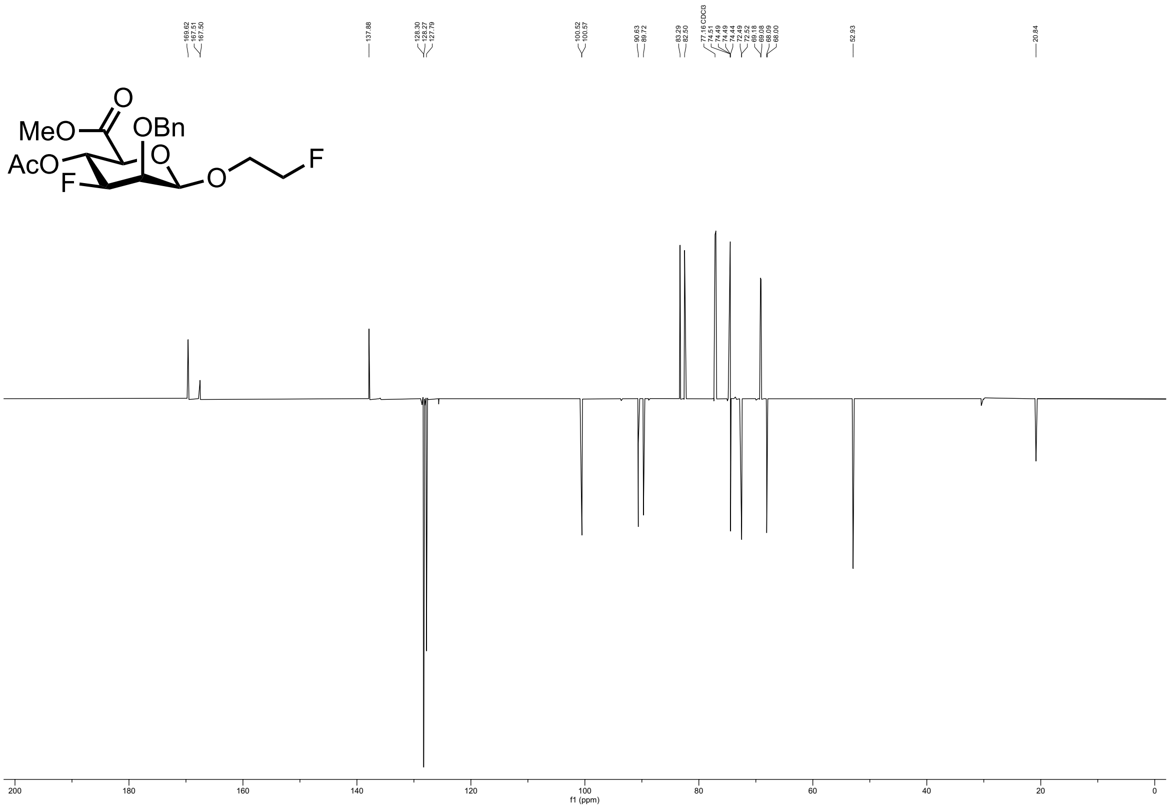

Methyl (2,2-difluoroethyl 4-*O*-acetyl-2-*O*-benzyl-2-deoxy-2-fluoro-α/β-D-mannopyranosyl uronate) (7C), ^{1}H NMR (850 MHz, $CDCl_3$)

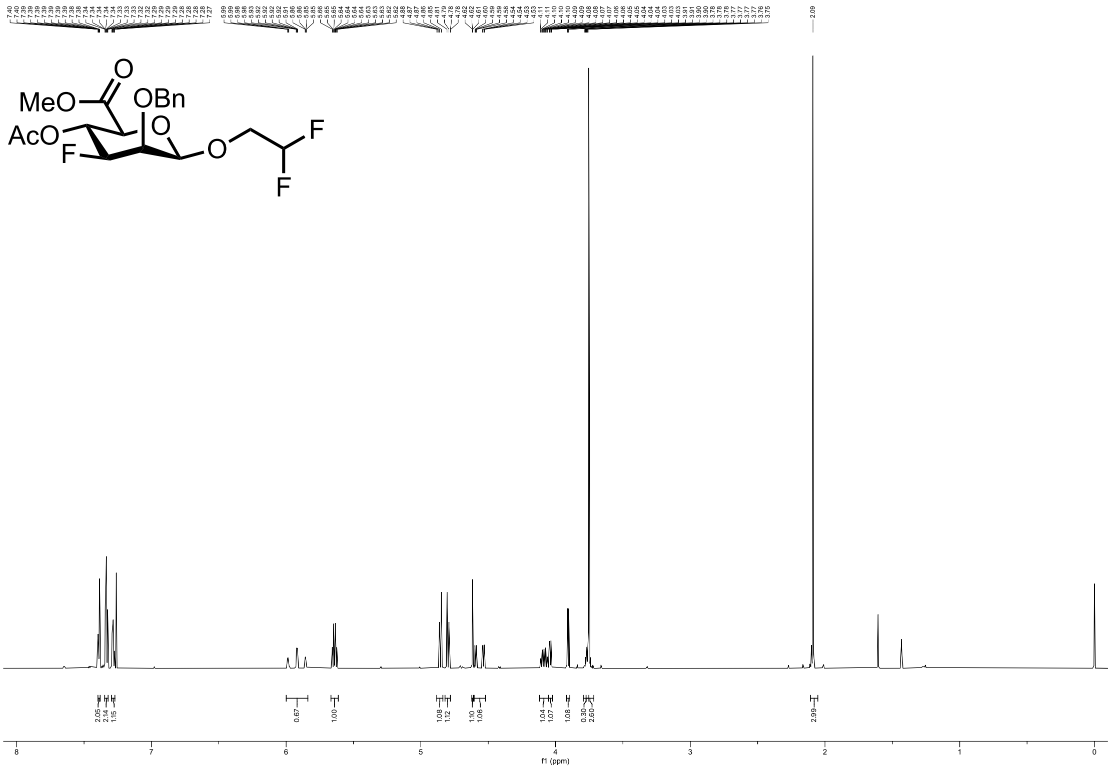

Methyl (2,2-difluoroethyl 4-*O*-acetyl-2-*O*-benzyl-2-deoxy-2-fluoro-α/β-D-mannopyranosyl uronate) (7C), ^{13}C NMR (214 MHz, $CDCl_3$)

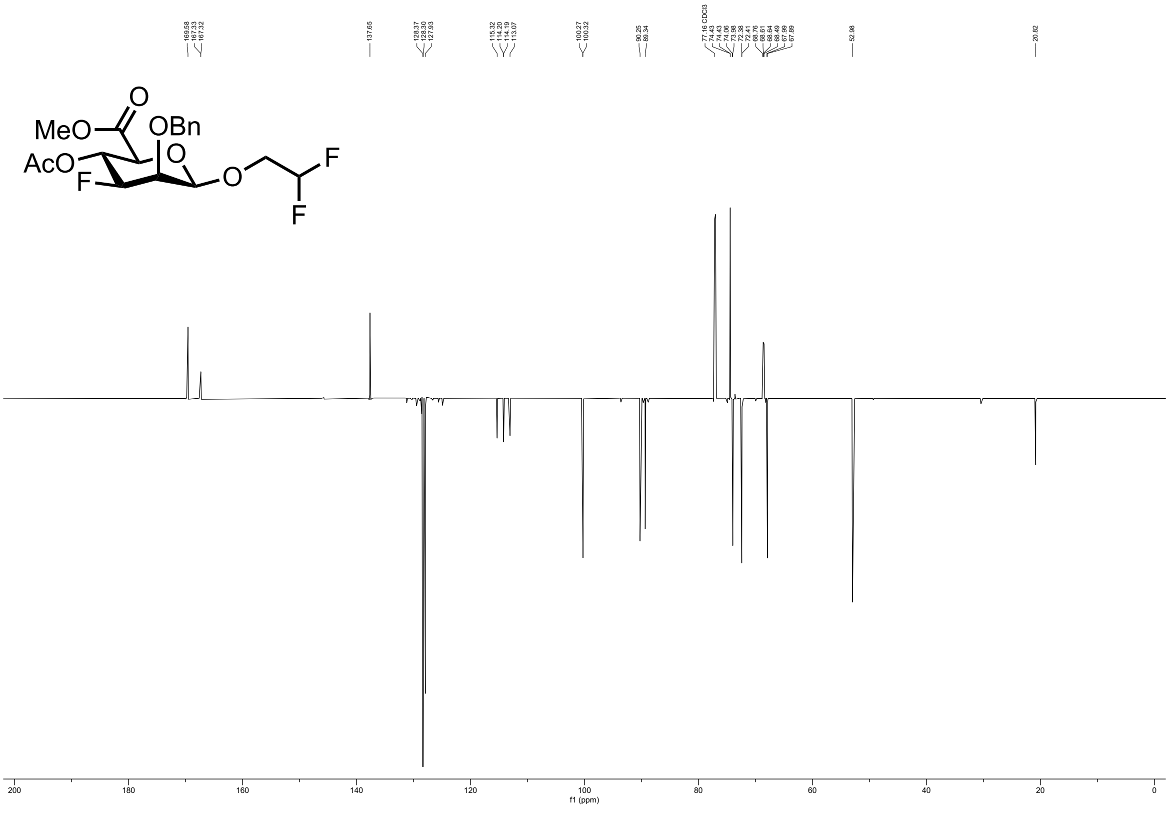

Methyl (2,2,2-trifluoroethyl 4-O-acetyl-2-*O*-benzyl-2-deoxy-2-fluoro-α/β-ᴅ-mannopyranosyl uronate) (7D), ^{1}H NMR (850 MHz, CDCl₃)

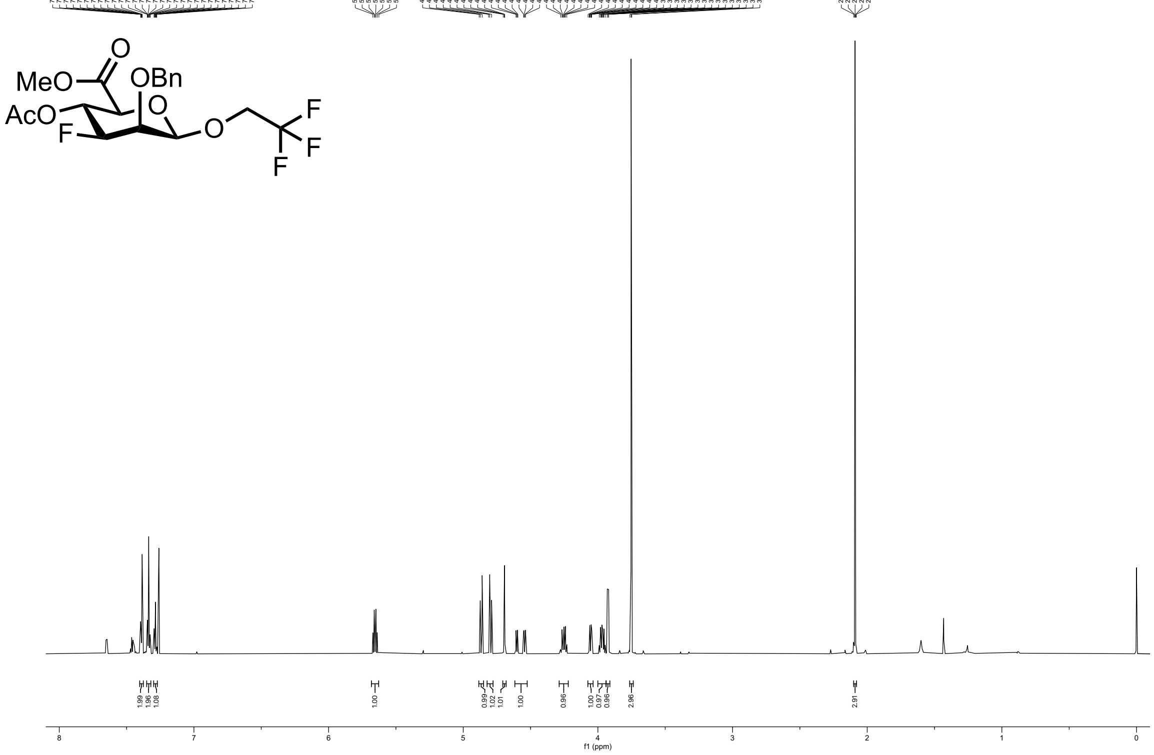

Methyl (2,2,2-trifluoroethyl 4-O-acetyl-2-*O*-benzyl-2-deoxy-2-fluoro-α/β-ᴅ-mannopyranosyl uronate) (7D), ^{13}C NMR (214 MHz, CDCl₃)

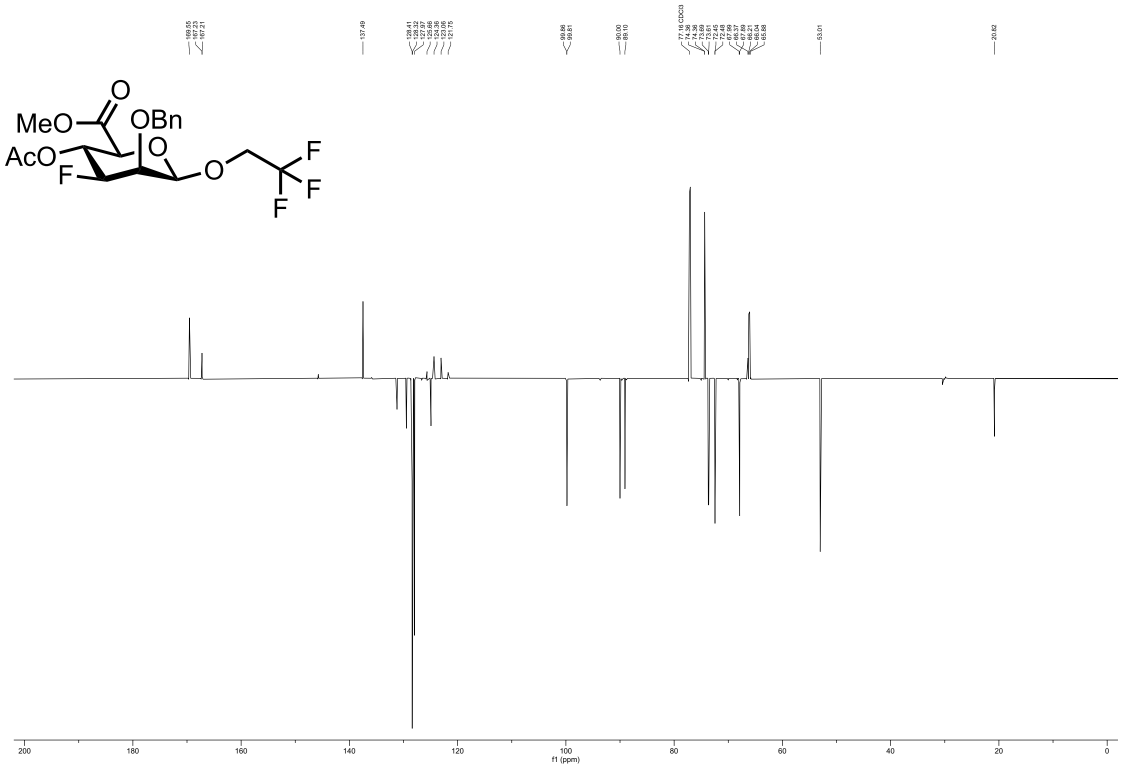

β-D-Mannopyranosyl uronate-(1→4)-β-D-mannopyranosyl uronate-(1→4)- 5-aminopentyl-α-D- mannopyranosyl uronate (9), ^{1}H NMR (700 MHz, D_2O)

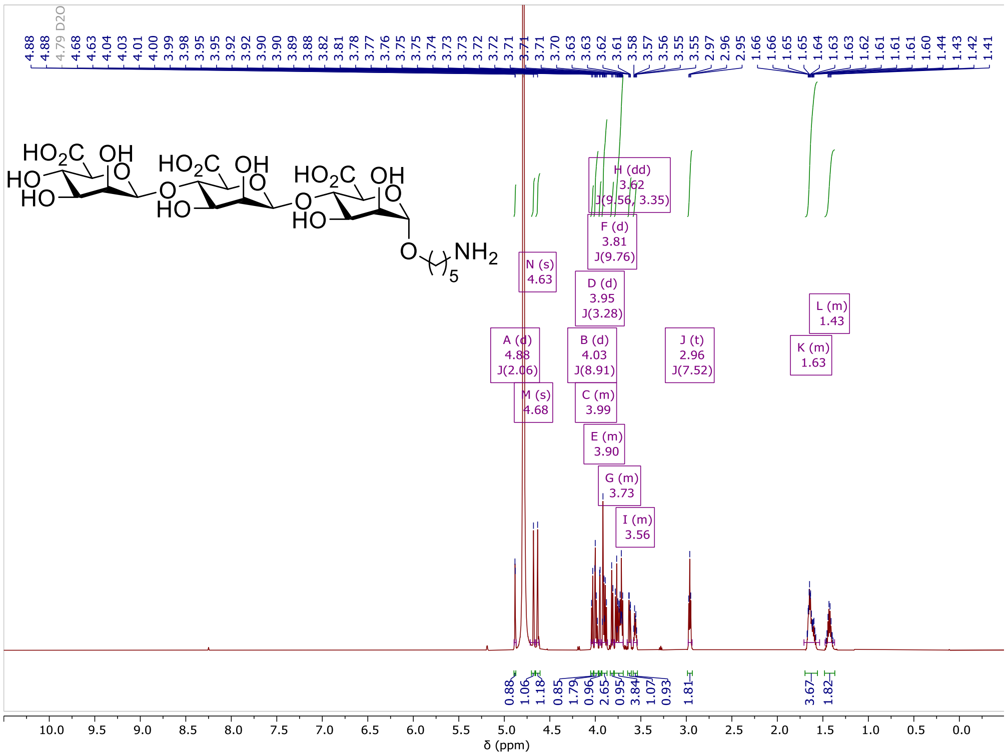

β-D-Mannopyranosyl uronate-(1→4)-β-D-mannopyranosyl uronate-(1→4)- 5-aminopentyl-α-D-mannopyranosyl uronate (9), ^{13}C NMR (176 MHz, D_2O)

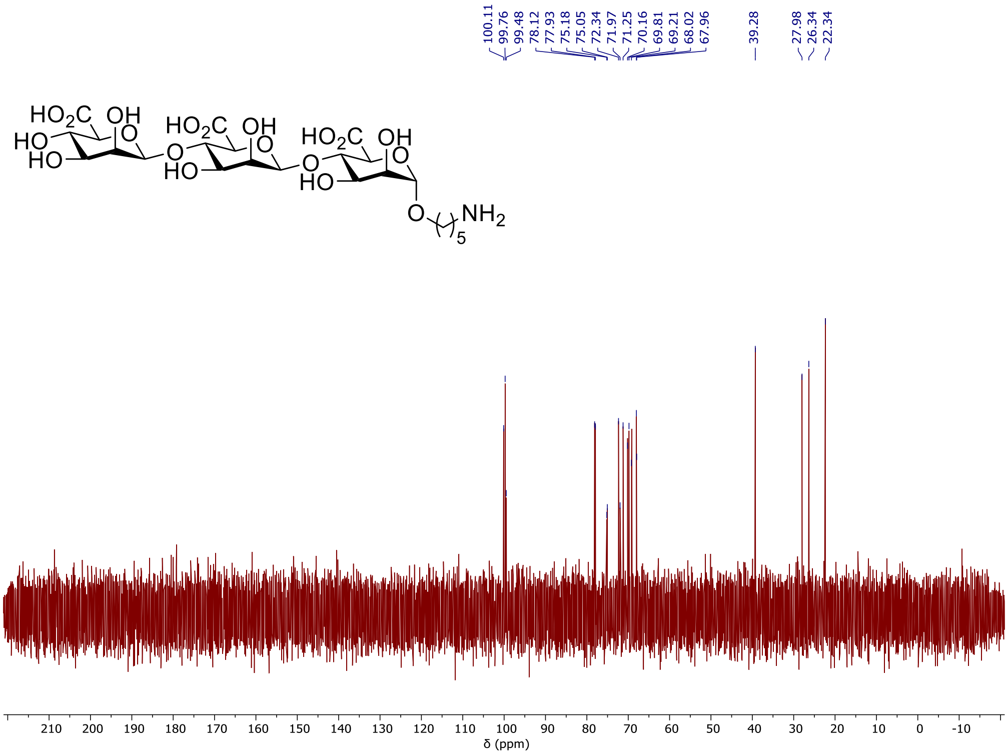

β-D-Mannopyranosyl uronate-(1→4)-3-deoxy-3-fluoro-β-D-mannopyranosyl uronate-(1→4)-5-aminopentyl-α-D-mannopyranosyl uronate (10), ^{1}H NMR (600 MHz, D_2O)

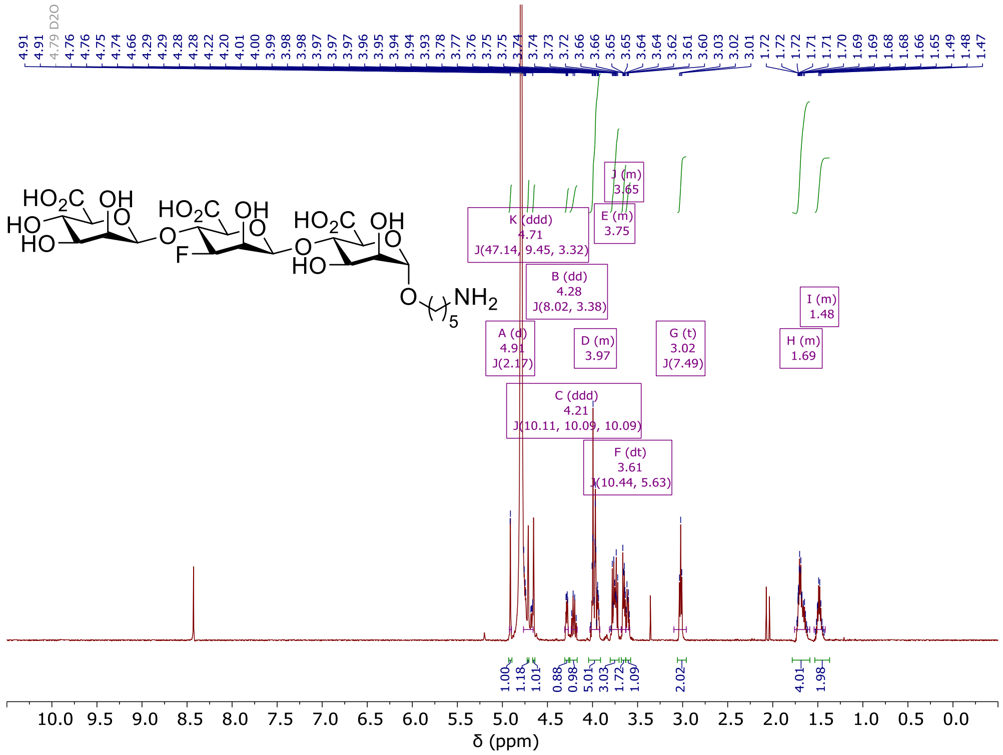

β-D-Mannopyranosyl uronate-(1→4)-3-deoxy-3-fluoro-β-D-mannopyranosyl uronate-(1→4)-5-aminopentyl-α-D-mannopyranosyl uronate (10), ^{19}F {^{1}H} NMR (565 MHz, D_2O)

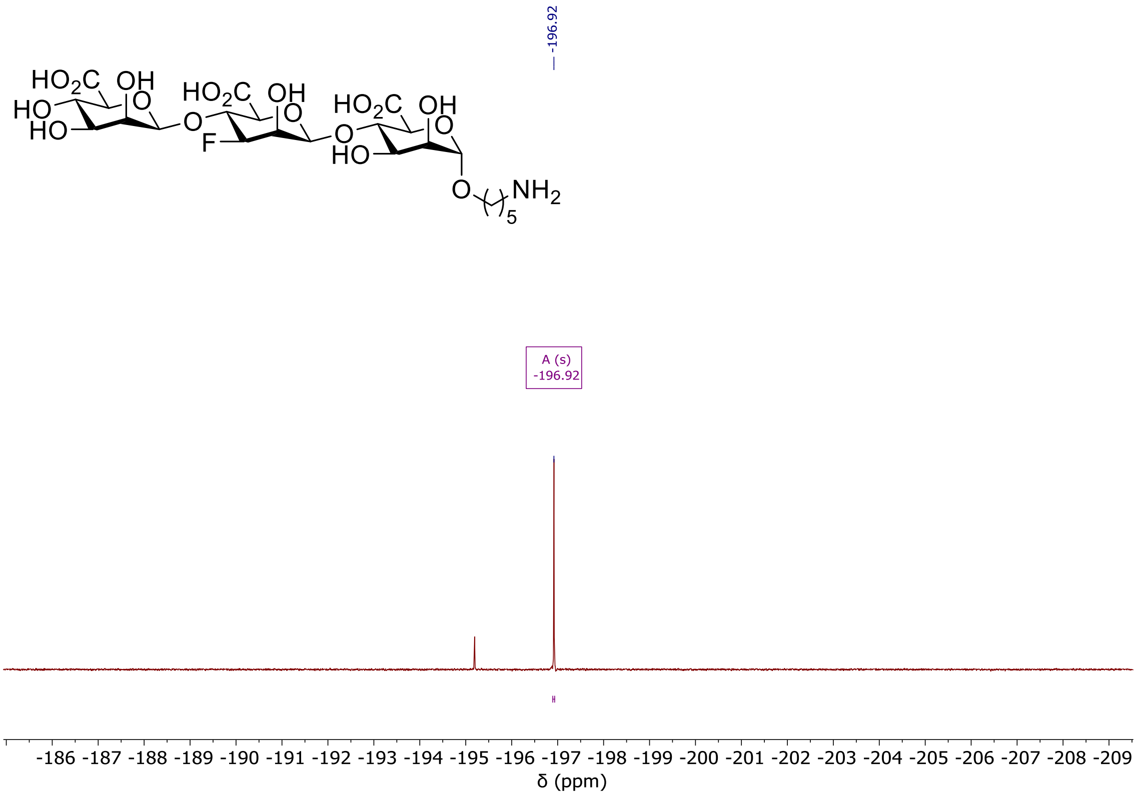

β-D-Mannopyranosyl uronate-(1→4)-β-D-mannopyranosyl uronate-(1→4)-β-D-mannopyranosyl uronate-(1→4)-β-D-mannopyranosyl uronate-(1→4)-β-D-mannopyranosyl uronate-(1→4)-5-aminopentyl-α-D-mannopyranosyl uronate (11), ^{1}H NMR (600 MHz, D_2O)

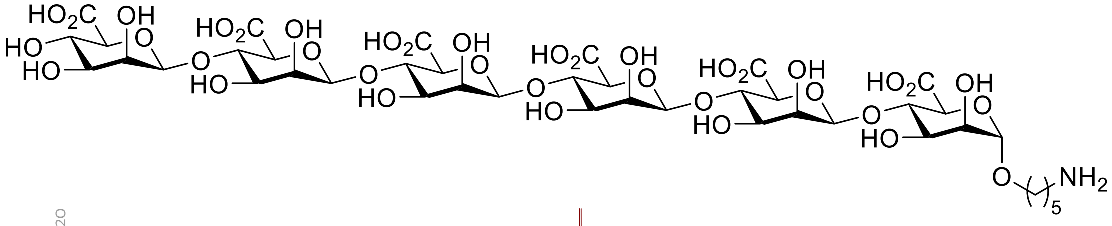

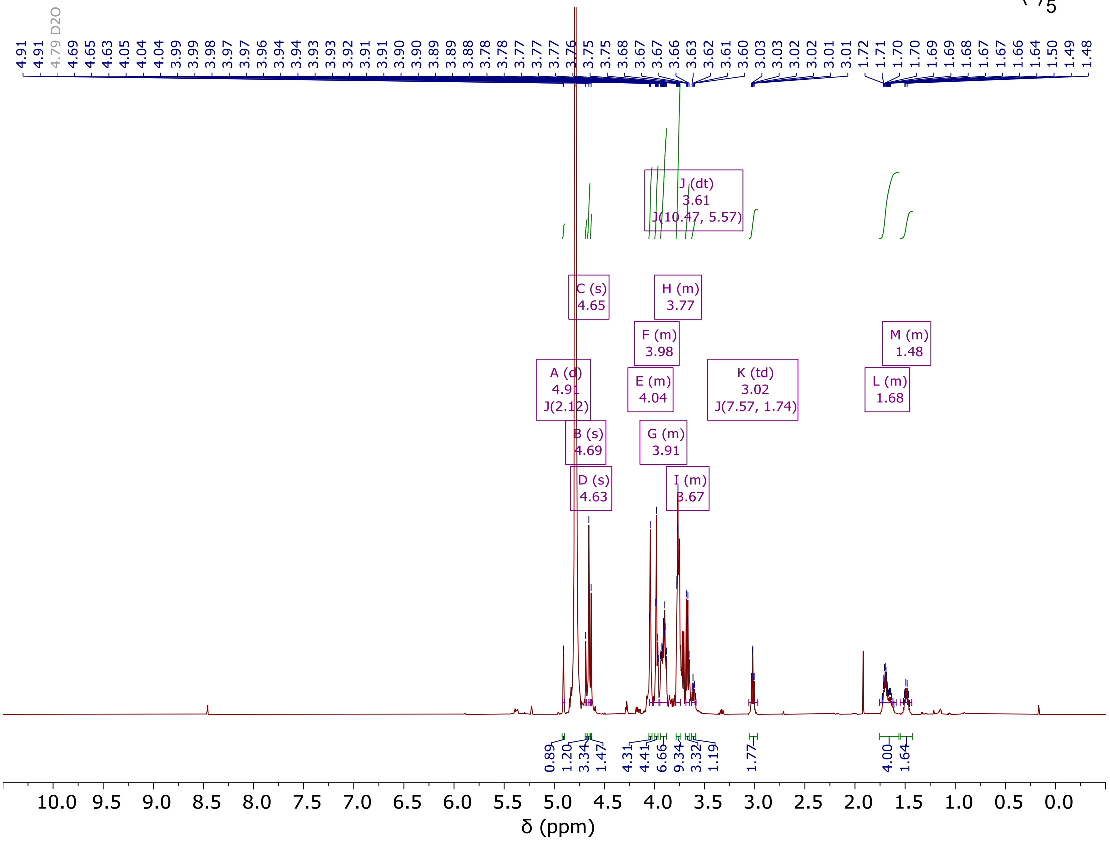

β-D-Mannopyranosyl uronate-(1→4)-β-D-mannopyranosyl uronate-(1→4)-β-D-mannopyranosyl uronate-(1→4)-β-D-mannopyranosyl uronate-(1→4)-β-D-mannopyranosyl uronate-(1→4)-5-aminopentyl-α-D-mannopyranosyl uronate (11), ^{13}C NMR (151 MHz, D_2O)

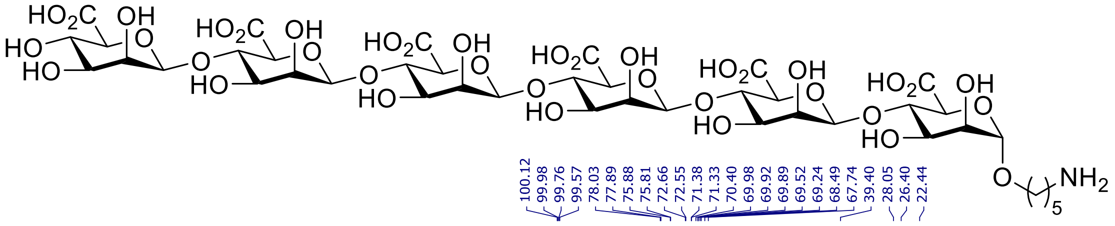

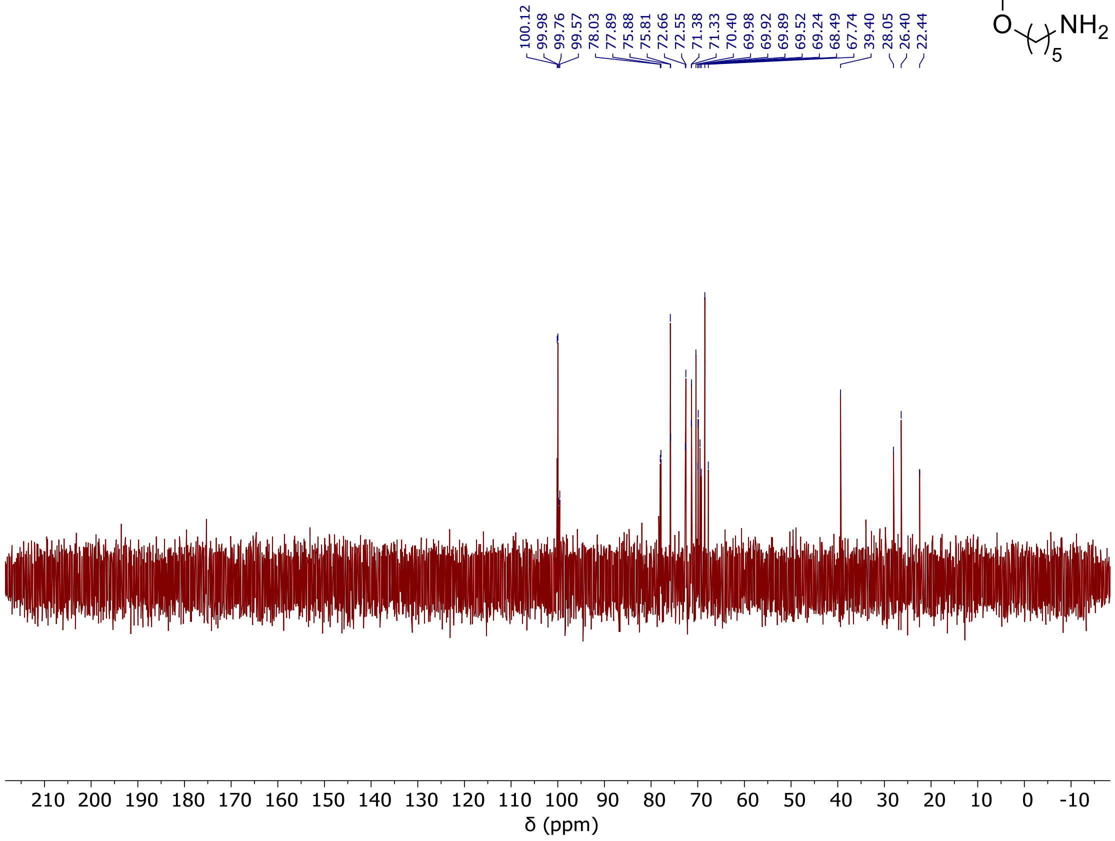

β-D-Mannopyranosyl uronate-(1→4)-β-D-mannopyranosyl uronate-(1→4)-3-deoxy-3-fluoro-β-D-mannopyranosyl uronate-(1→4)-β-D-mannopyranosyl uronate-(1→4)-β-D-mannopyranosyl uronate-(1→4)-5-aminopentyl-α-D-mannopyranosyl uronate (12), ^{1}H NMR (600 MHz, D_2O)

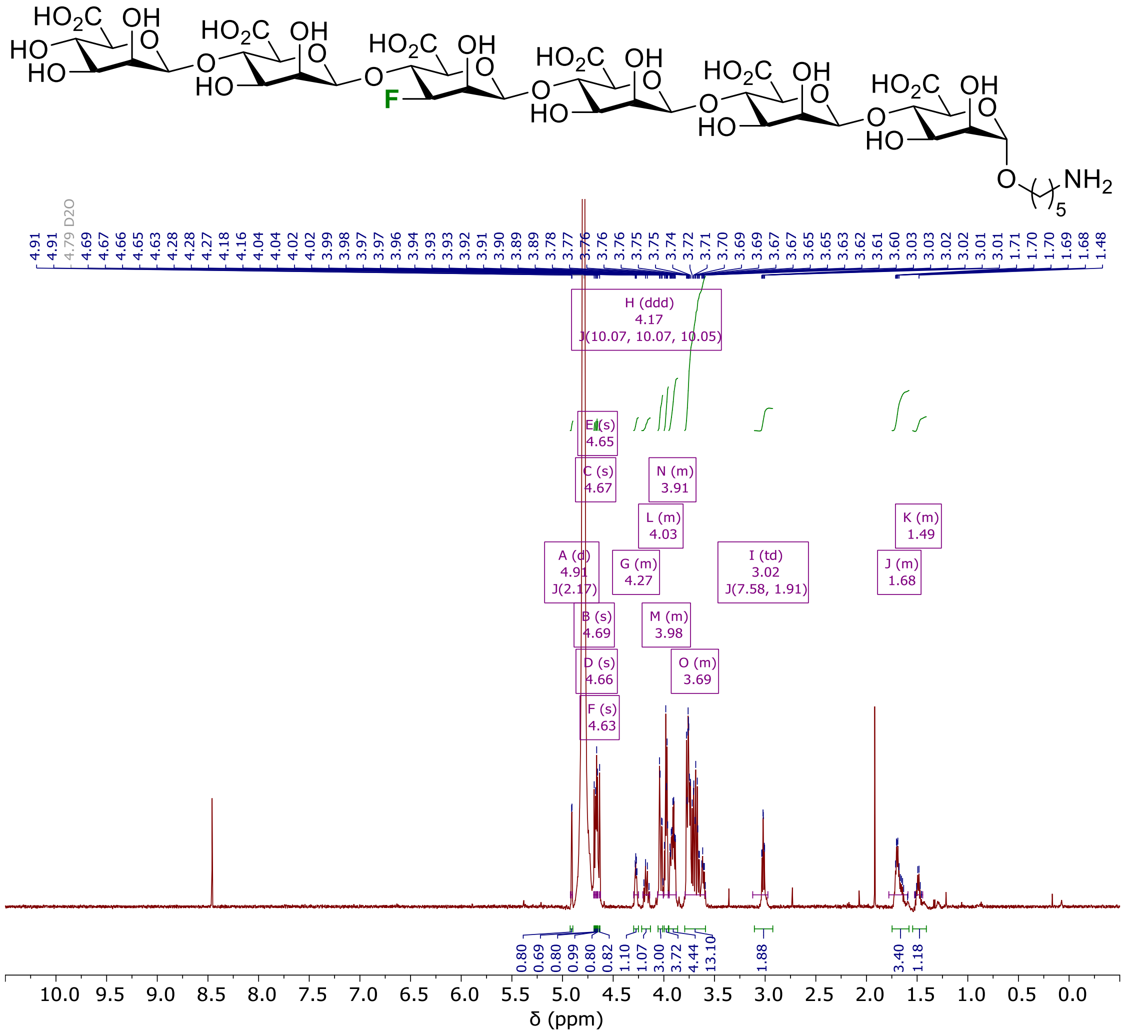

β-D-Mannopyranosyl uronate-(1→4)-β-D-mannopyranosyl uronate-(1→4)-3-deoxy-3-fluoro-β-D-mannopyranosyl uronate-(1→4)-β-D-mannopyranosyl uronate-(1→4)-β-D-mannopyranosyl uronate-(1→4)-5-aminopentyl-α-D-mannopyranosyl uronate (12), ^{19}F NMR (564 MHz, D_2O)

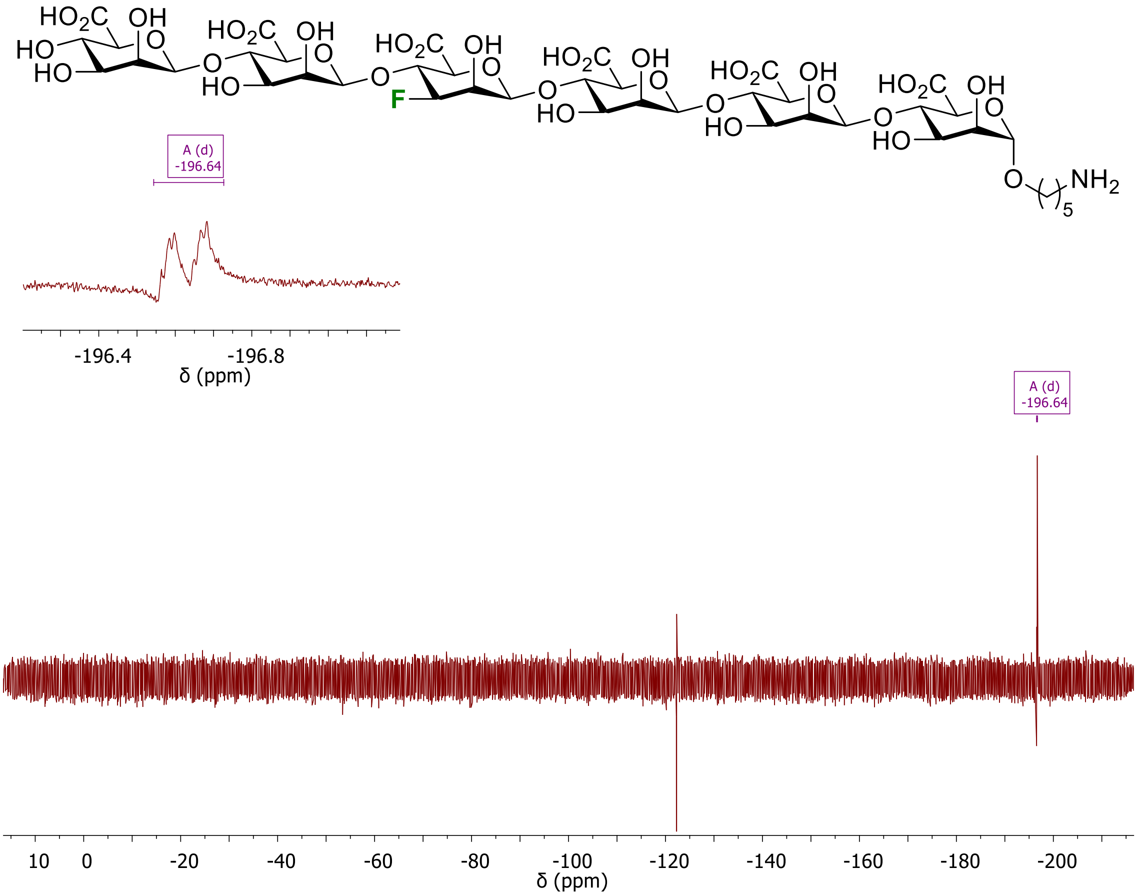

β-D-Mannopyranosyl uronate-(1→4)-3-deoxy-3-fluoro-β-D-mannopyranosyl uronate-(1→4)-β-D-mannopyranosyl uronate-(1→4))-β-D-mannopyranosyl uronate-(1→4)-3-deoxy-3-fluoro-β-D-mannopyranosyl uronate-(1→4)-5-aminopentyl-α-D-mannopyranosyl uronate (13), ^{1}H NMR (700 MHz, D_2O)

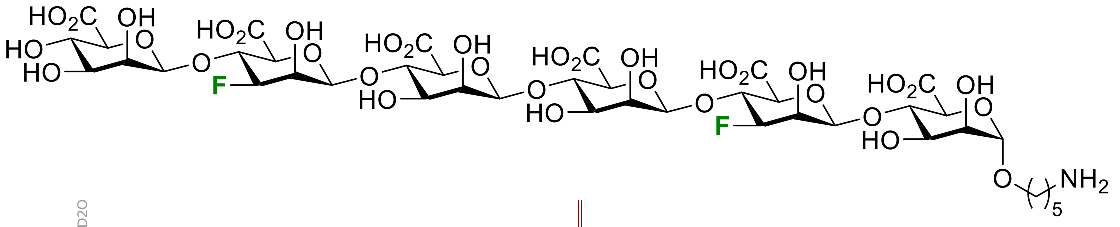

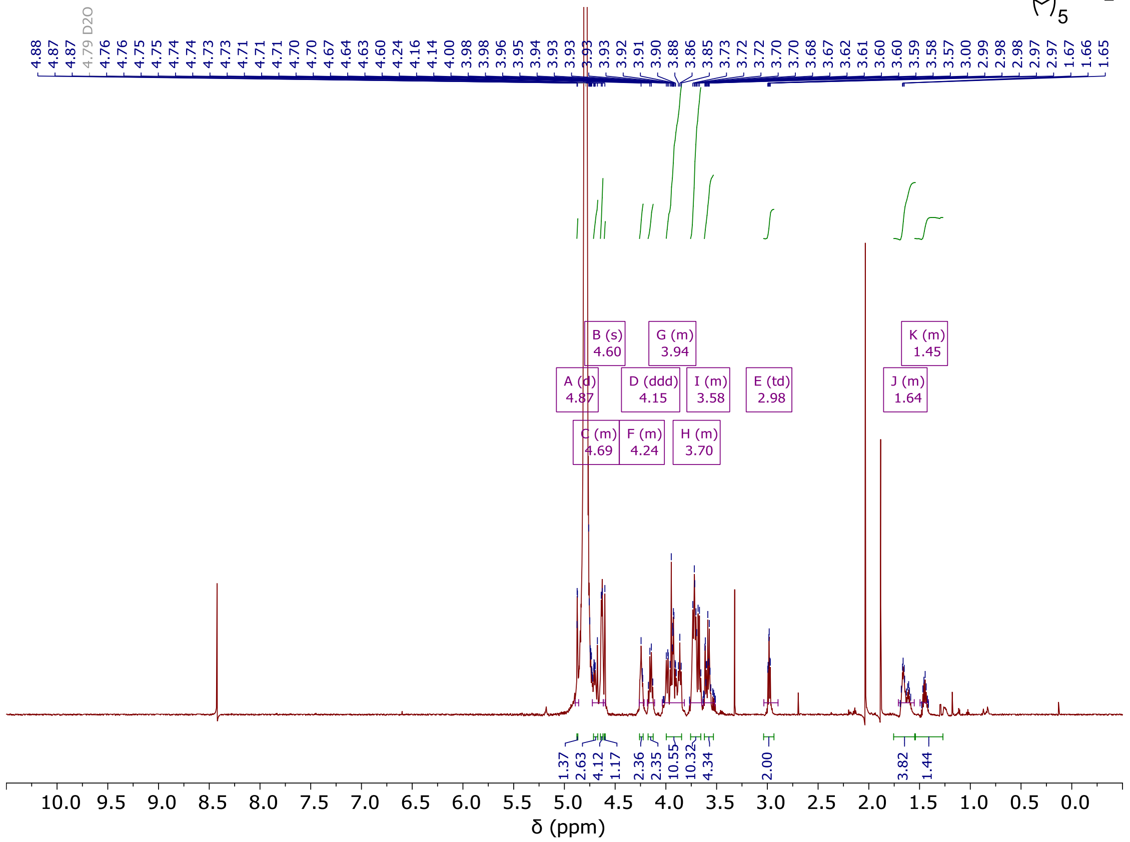

β-D-Mannopyranosyl uronate-(1→4)-3-deoxy-3-fluoro-β-D-mannopyranosyl uronate-(1→4)-β-D-mannopyranosyl uronate-(1→4))-β-D-mannopyranosyl uronate-(1→4)-3-deoxy-3-fluoro-β-D-mannopyranosyl uronate-(1→4)-5-aminopentyl-α-D-mannopyranosyl uronate (13), ^{19}F NMR (564 MHz, D_2O)

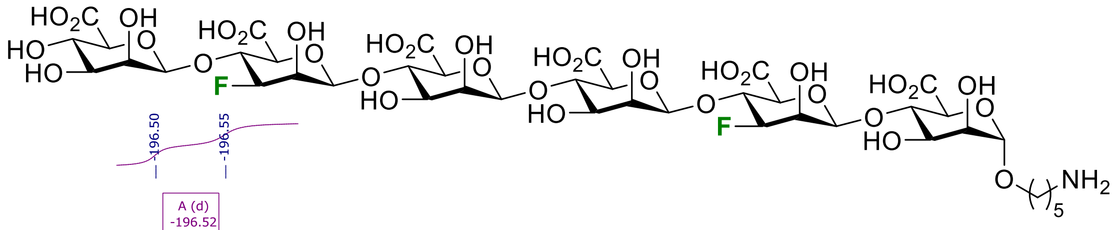

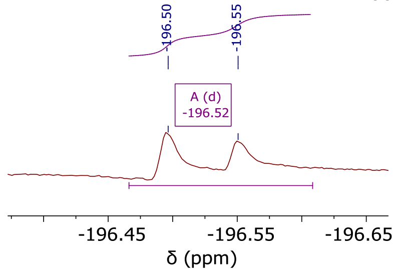

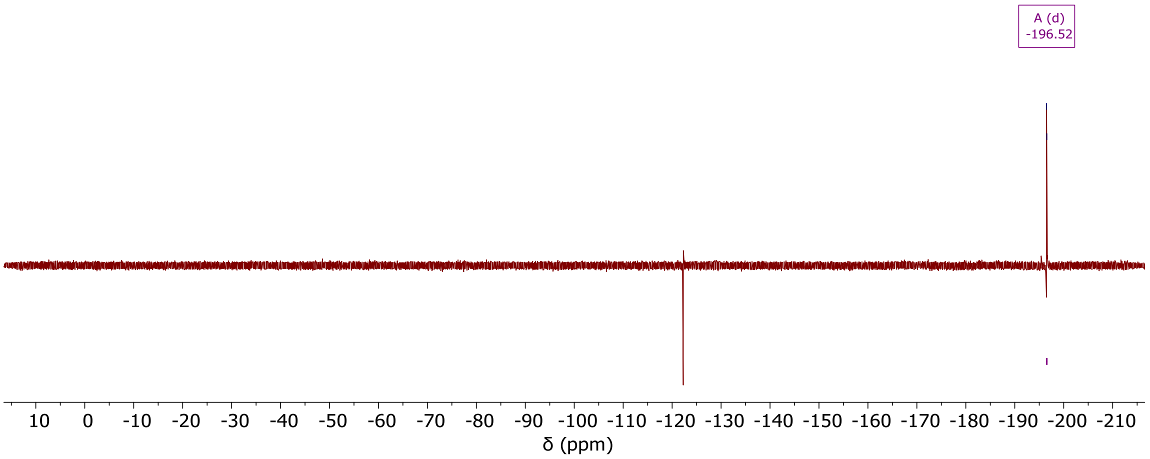

S9. 2D NMR Spectra of Oligosaccharides and Conformational Analysis

9.1 General Experimental

Following synthesis of ManA oligosaccharides, initial HSQC NMR spectra were acquired using a Varian NMR 600 MHz Premium Shielded NMR spectrometer or a Bruker 700 MHz Ascend NMR spectrometer. Later NMR experiments were performed to support the structural assignment and to carry out conformational analysis of the oligomeric compounds. These spectra were acquired on two instruments: Bruker Avance III HD 800 MHz spectrometer equipped with a TCI cryogenically cooled probe, and Bruker Avance NEO 600 MHz spectrometer equipped with TXI and SEF room temperature probes for the ^{19}F-NMR experiments. Samples were dissolved in D_2O, and measurements were conducted at temperatures ranging from 293 to 298 K. Compound concentrations ranged from 0.4 to 1.0 mM. NMR experiments for assignment and conformational analysis procedures were performed in PBS buffer/D_2O, while $CaCl_2$ titration experiments were carried out in TRIS-d_{11} buffer/D_2O, using TSP-d_4 and trifluoroethanol as ^{1}H and ^{19}F chemical shift reference, respectively. The following 2D experiments were recorded: COSY, TOCSY, NOESY, ROESY, HSQC, HSQC-TOCSY and HMBC, using standard Bruker pulse sequences. Acquisition parameters typically included 1024-2048 × 256-512 complex points and 8–48 scans per increment. Spectral widths were set to 12 ppm for ^{1}H and 160 ppm for ^{13}C. NOESY mixing times ranged from 200 to 400 ms, depending on the particular compound, while the ROESY spinlock mixing time was set to 300ms. For the fluorinated analogues, ^{1}H-^{19}F-HSQC-TOCSY type spectra were obtained using an *in house* written 2D sequence: F-relay-[H]HTOCSY: LONG_RANGE_CORRELATION, specifically designed to relay magnetization from fluorine to proton networks. NOE cross-peaks provided key information on spatial proximities. Strong intraresidue NOEs between H1 and H3/H5 typical of $^4C_1$ chair conformations, and interresidue NOEs between H1(i) and H4(i+1), were indicative of extended conformations across the β(1→4) linkage. The fluorinated analogues exhibited characteristic ^{13}C chemical shift features at the modified positions, reflecting changes in electronic environment and conformational preferences. In particular, the fluorinated residues (obviously lacking the OH3i–O5i+a hydrogen bond) showed deshielding at C5. For the provided HSQC NMR spectra, the assignment of cross peaks is indicated. For the provided ROESY and NOESY NMR spectra the key NOE cross peaks are highlighted.

9.2 2D NMR Spectra

^{1}H-^{13}C HSQC NMR spectrum of trisaccharide 9 (700 MHz, D_2O)

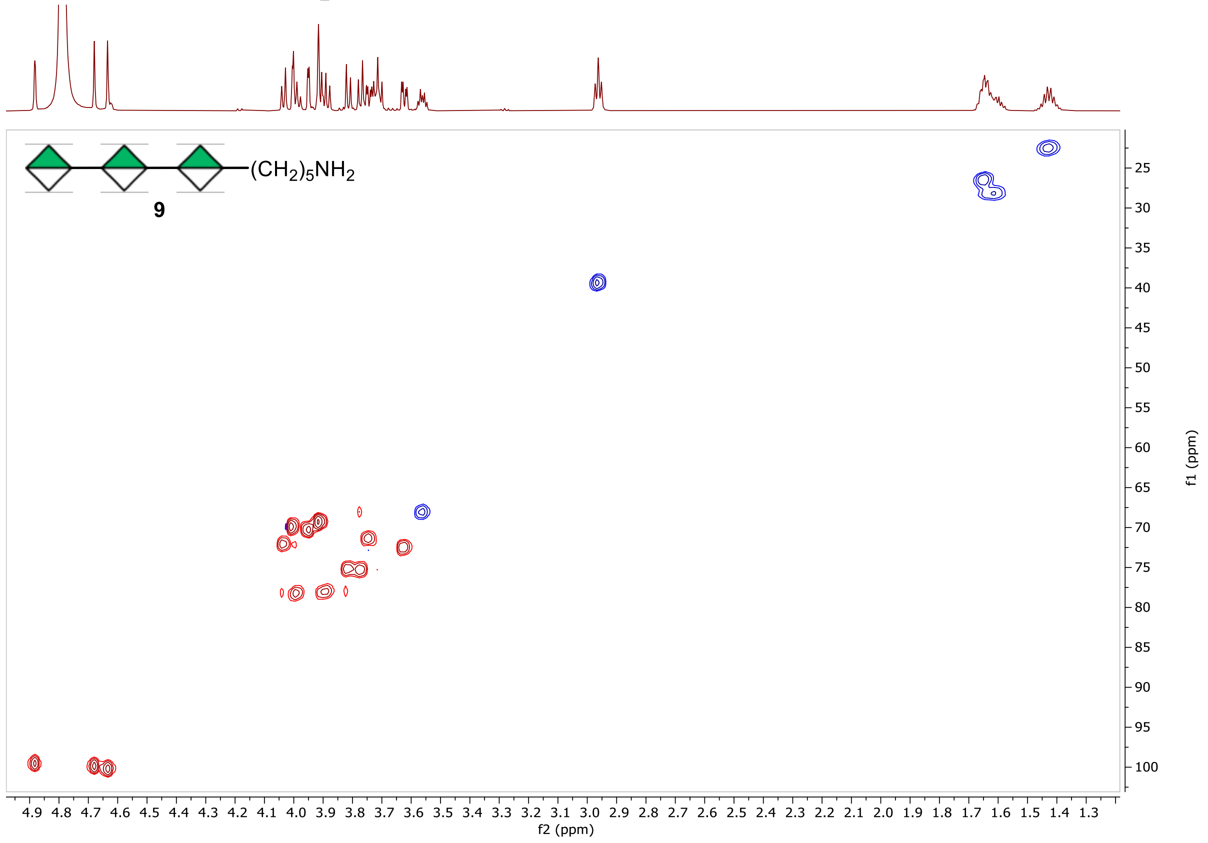

^{1}H-^{13}C HSQC NMR spectrum of trisaccharide 10 (600 MHz, D_2O)

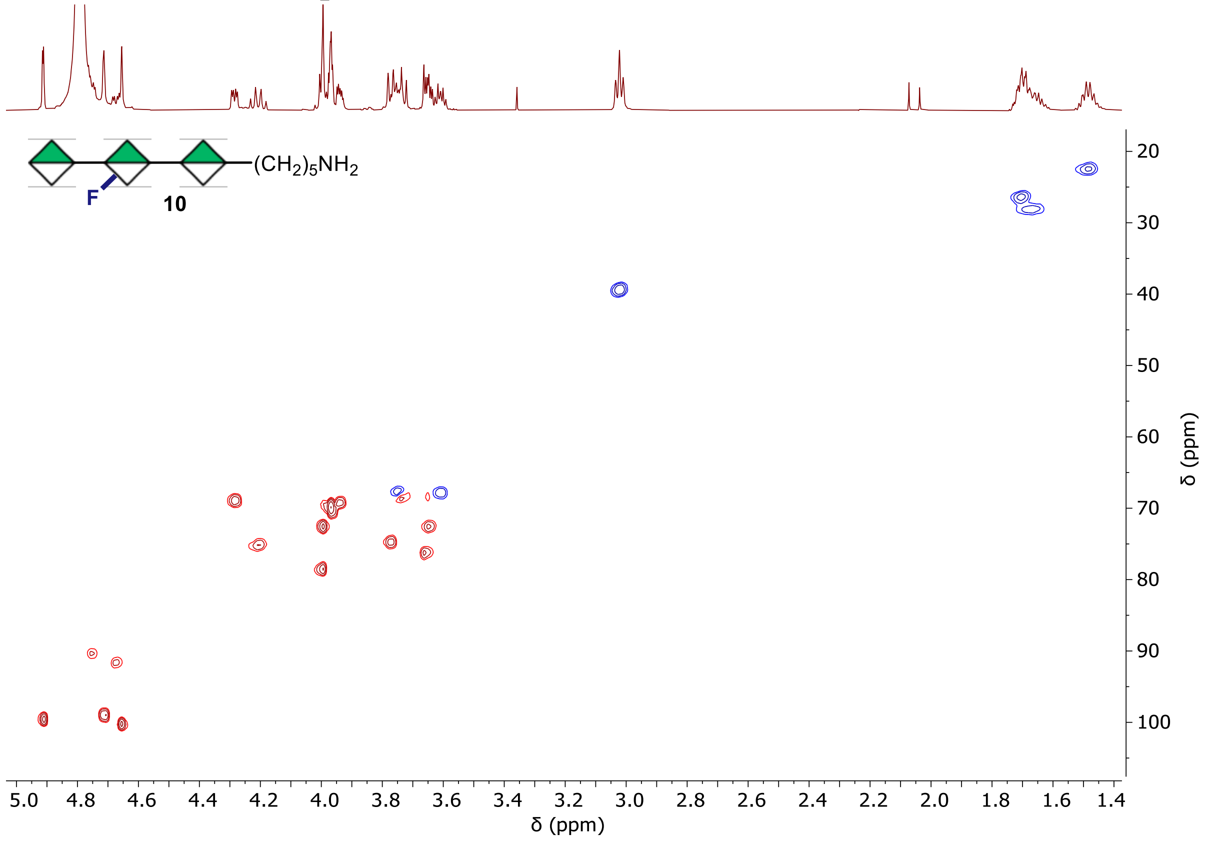

^{1}H-^{13}C HSQC NMR spectrum of hexasaccharide 11 (600 MHz, D_2O)

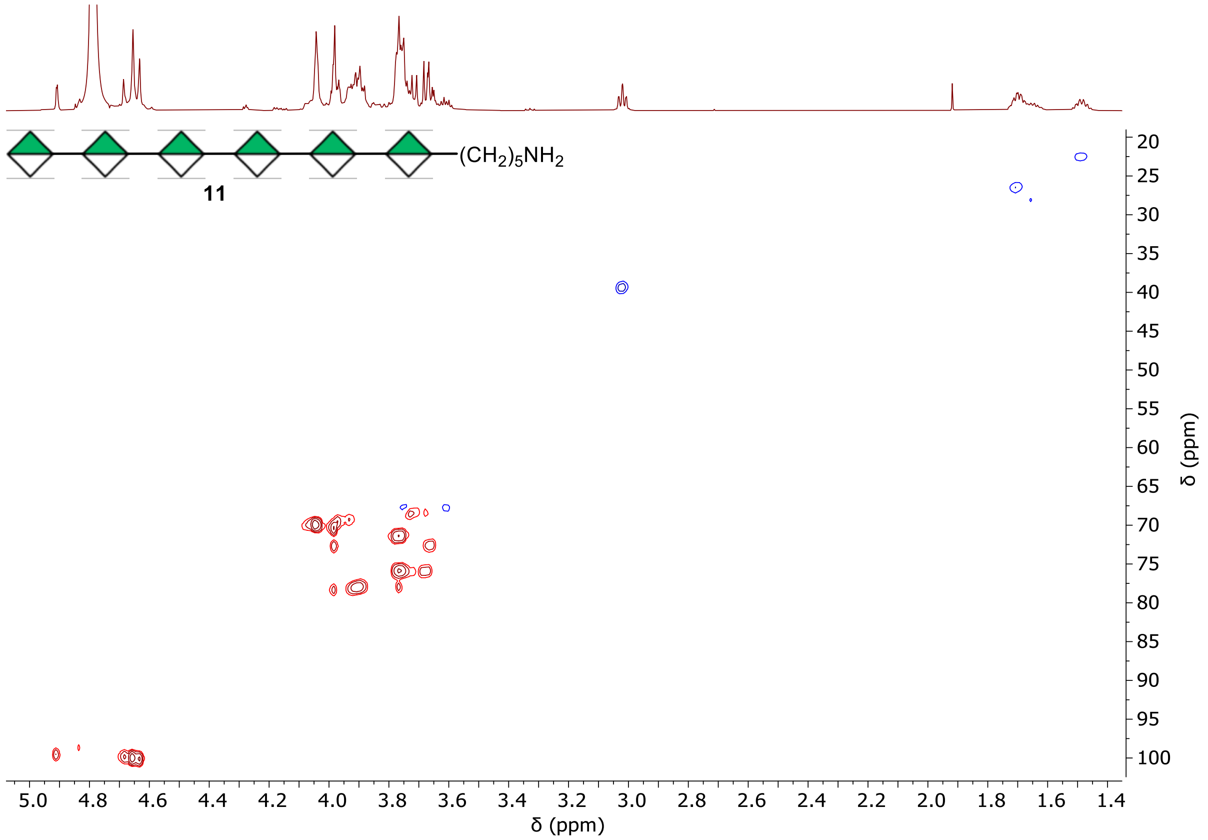

^{1}H-^{13}C HSQC NMR spectrum of hexasaccharide 12 (600 MHz, D_2O)

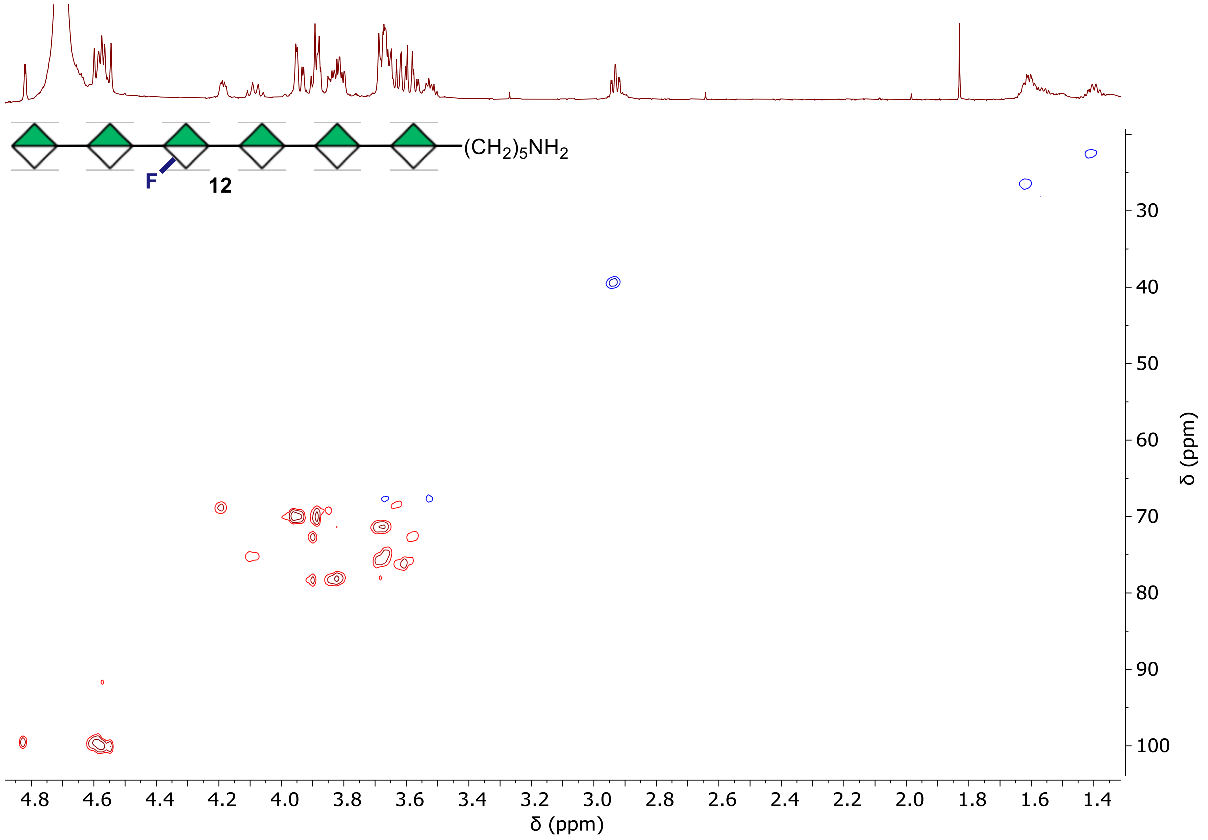

^{1}H-^{13}C HSQC NMR spectrum of hexasaccharide 13 (700 MHz, D_2O)

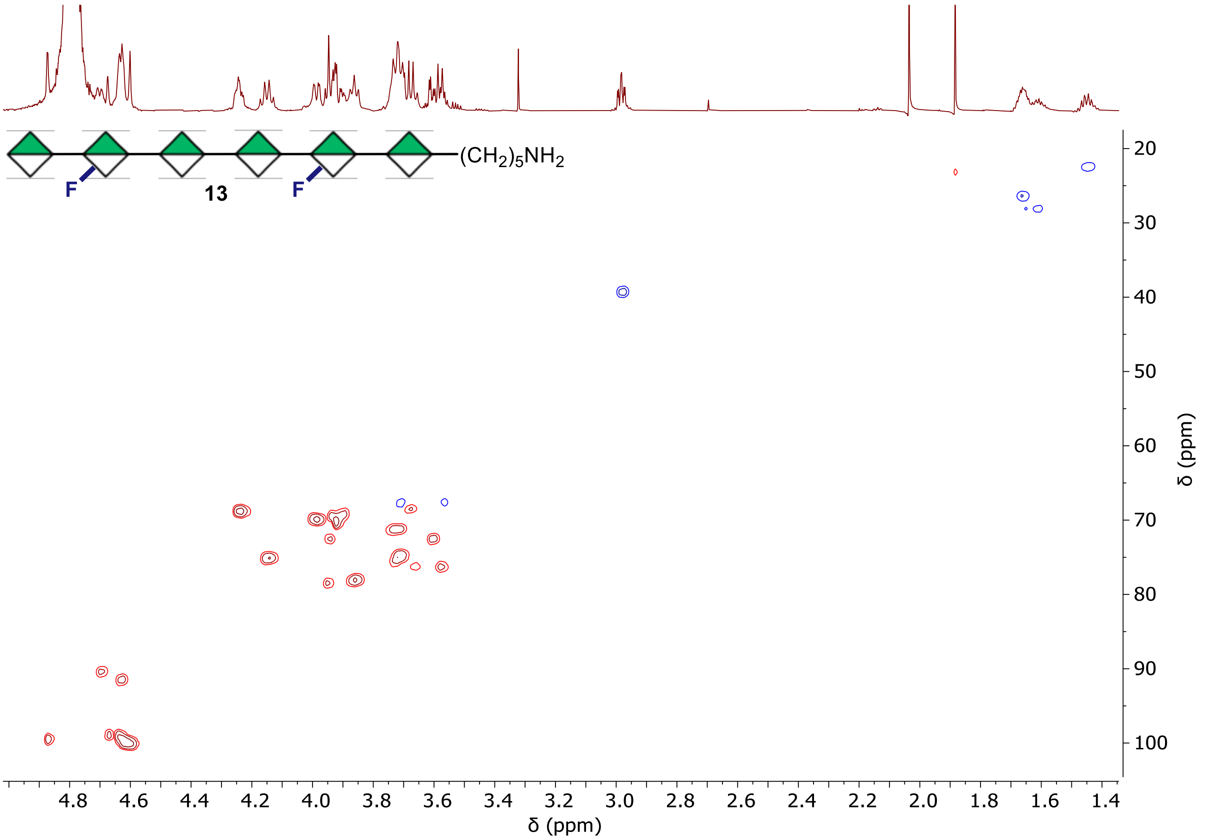

^{1}H-^{13}C HSQC NMR spectrum of trisaccharide 9 (800 MHz, D_2O), assigned

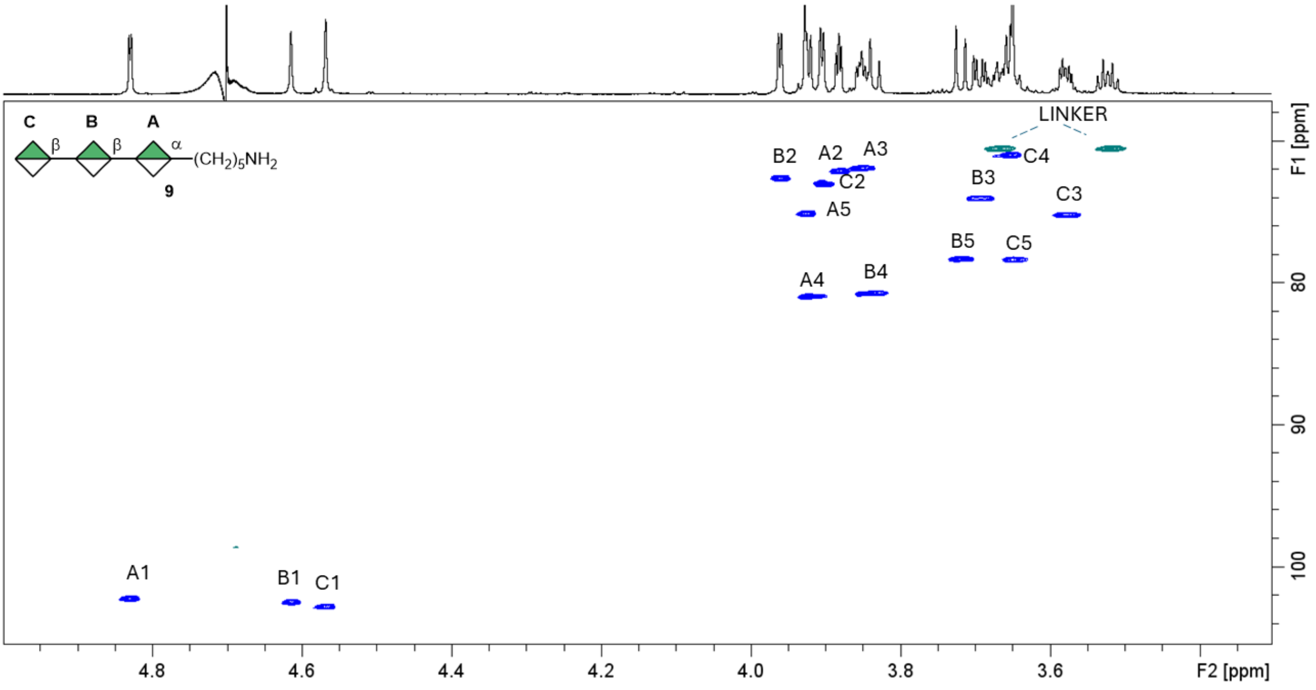

^{1}H-^{13}C HSQC NMR spectrum of trisaccharide 10 (800 MHz, D_2O), assigned

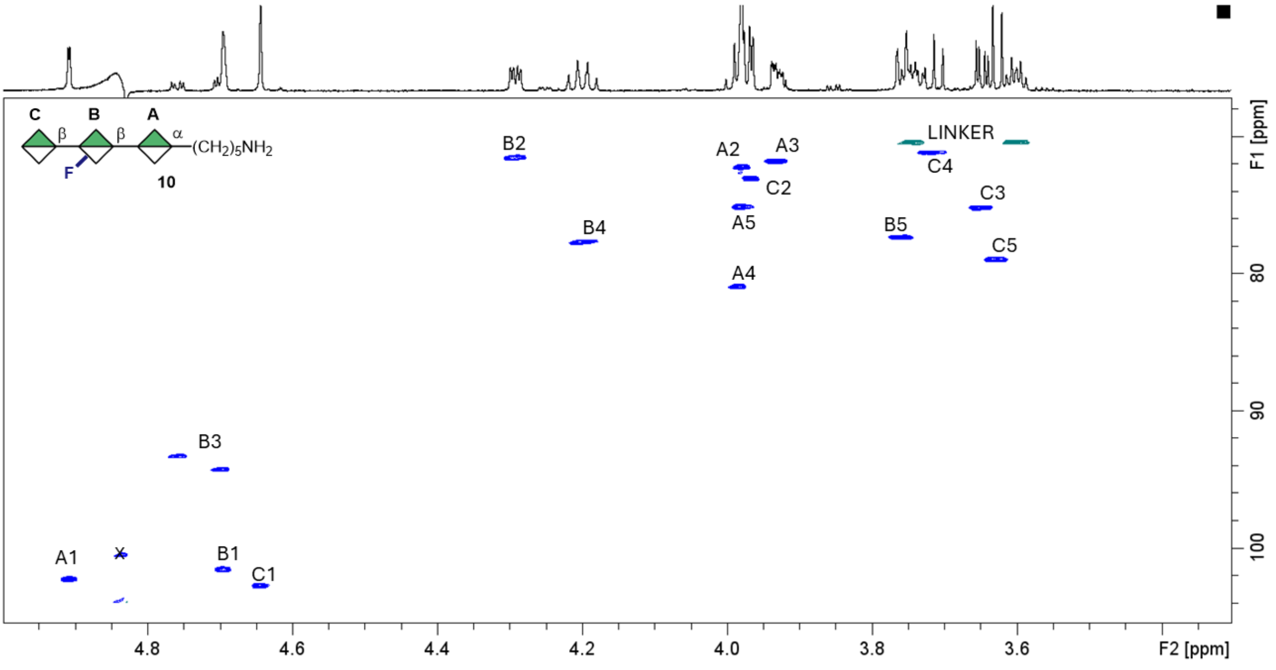

^{1}H-^{13}C HSQC NMR spectrum of hexasaccharide 11, (800 MHz, D_2O), assigned

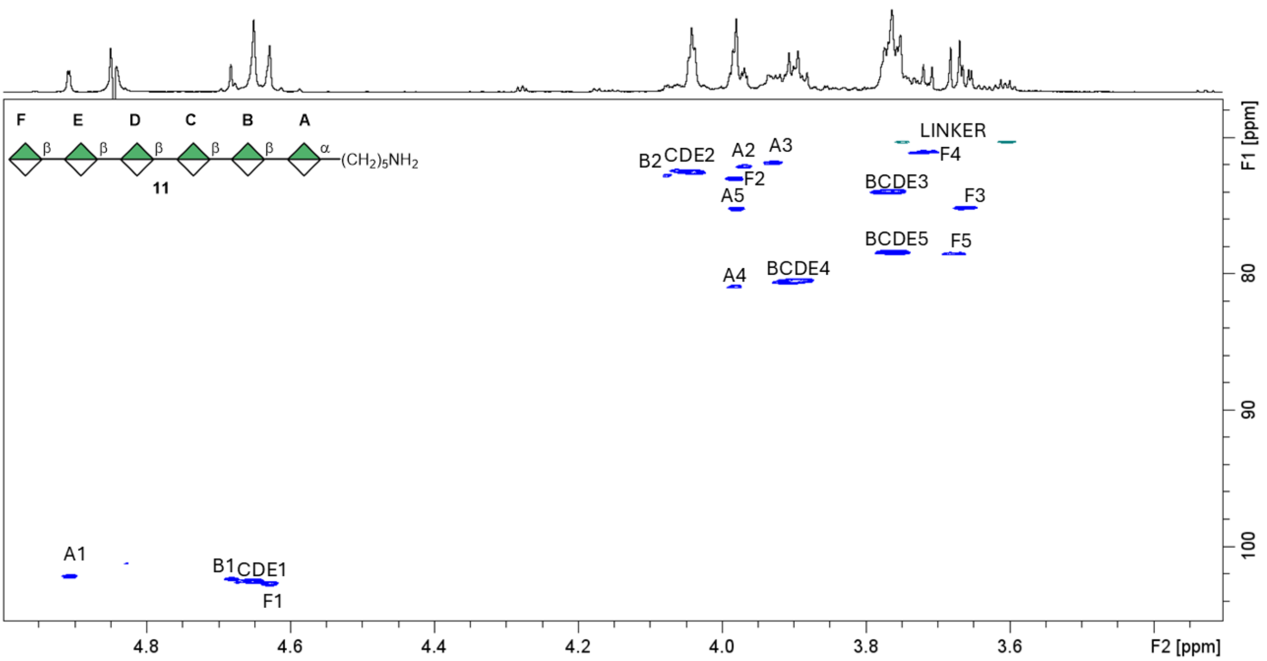

^{1}H-^{13}C HSQC NMR spectrum of hexasaccharide 12, (800 MHz, D_2O), assigned

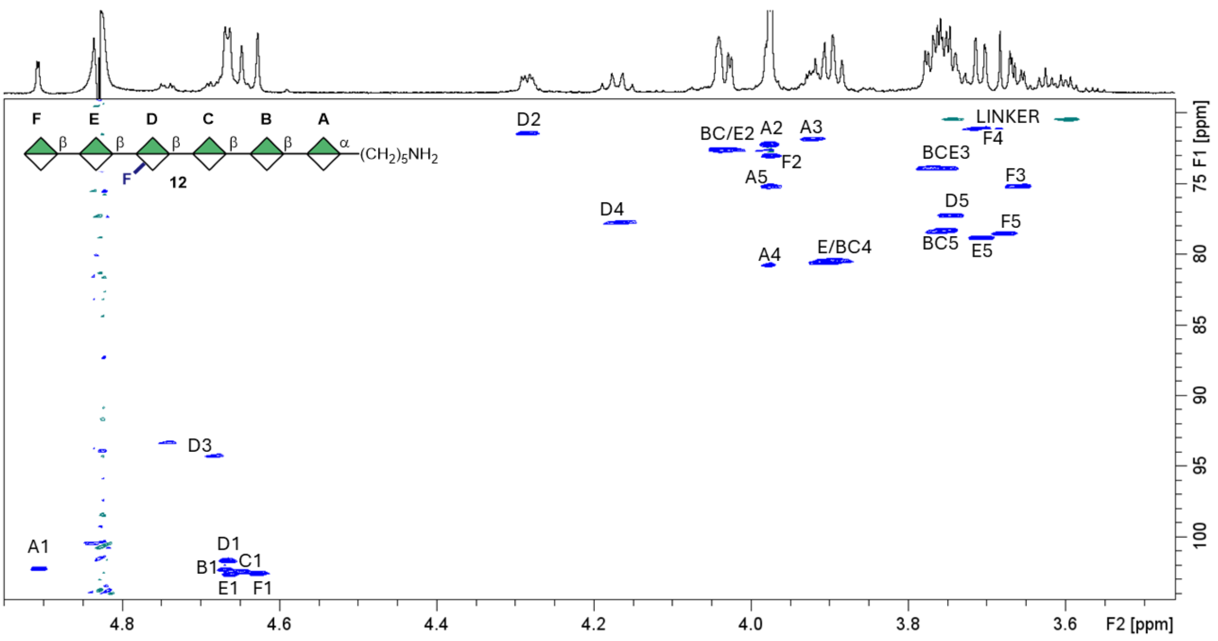

^{1}H-^{13}C HSQC NMR spectrum of hexasaccharide 13, (800 MHz, D_2O), assigned

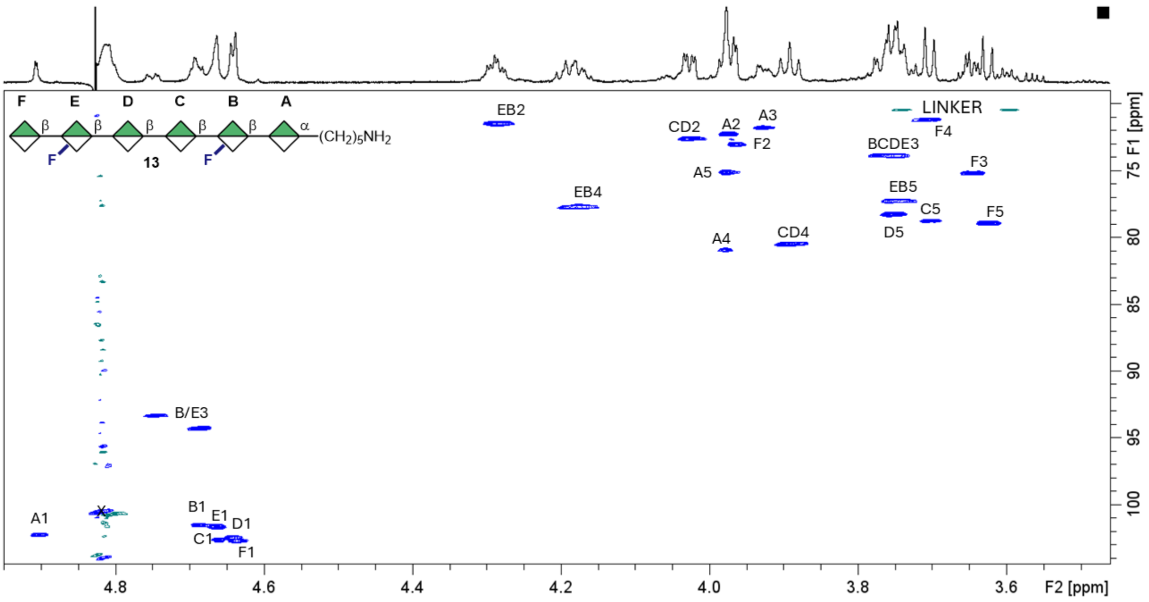

ROESY NMR Spectrum of Trisaccharide 9

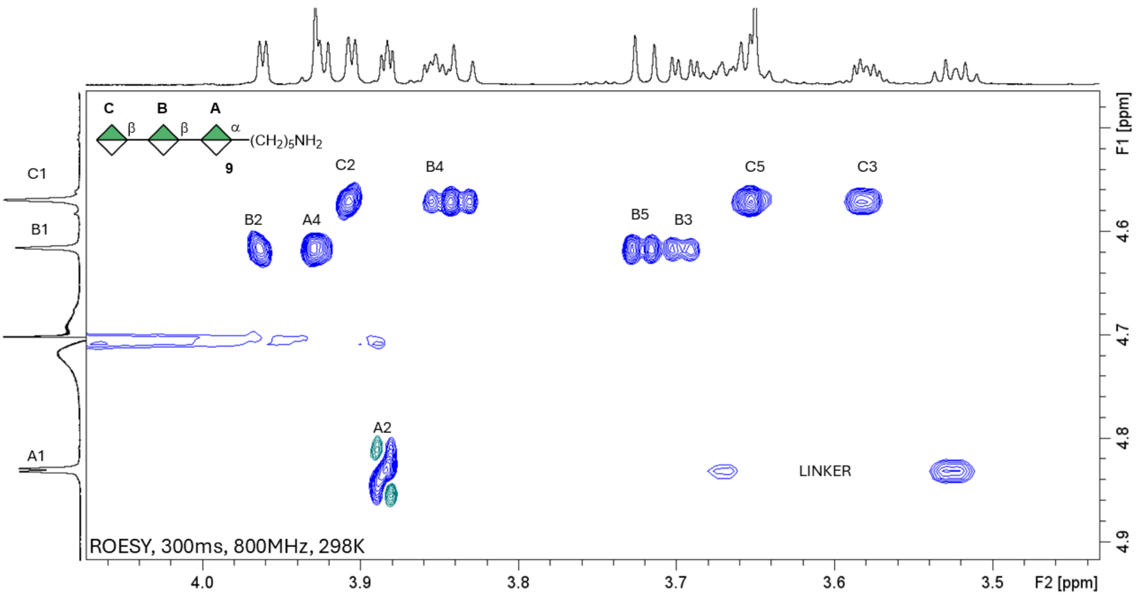

ROESY NMR Spectrum of Trisaccharide 10

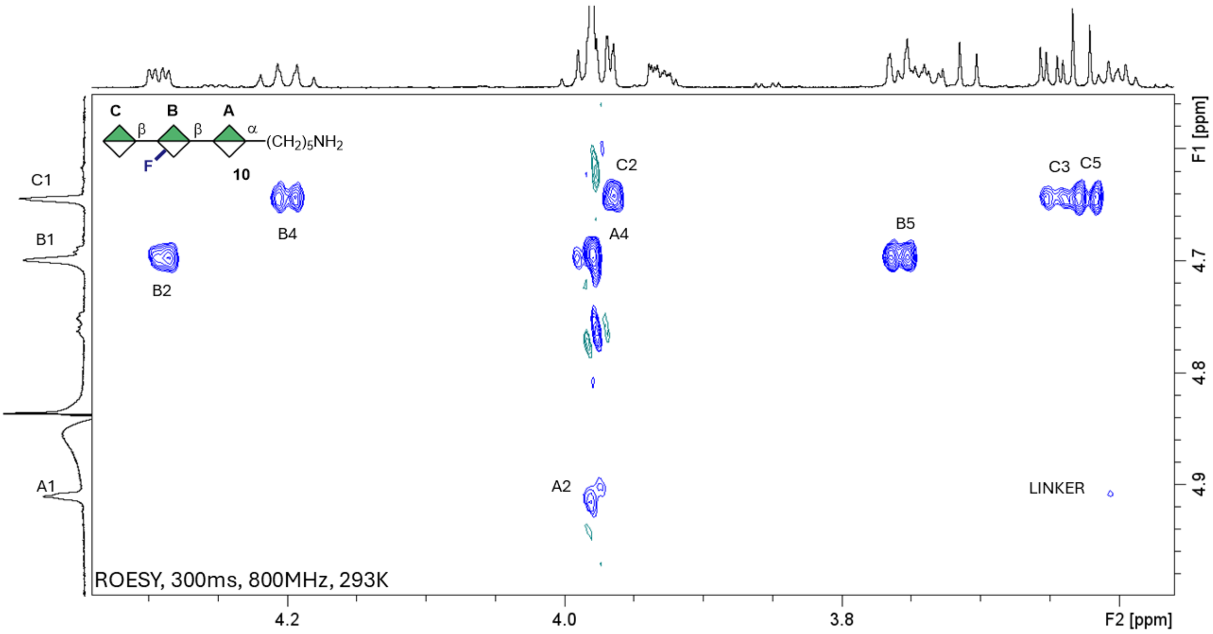

NOESY NMR Spectra of Hexasaccharide 11

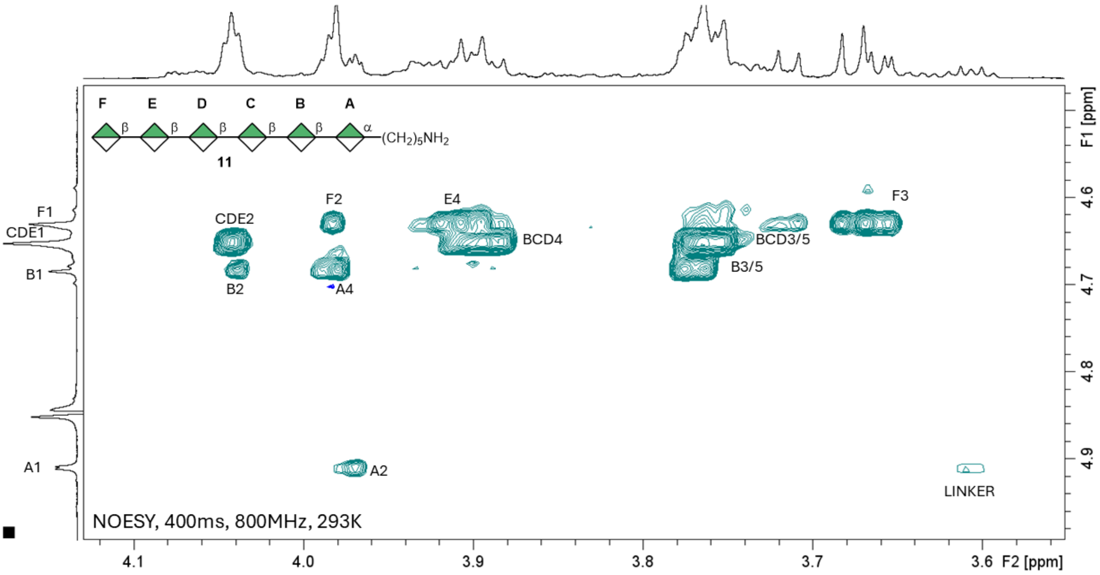

NOESY NMR Spectra of Hexasaccharide 12

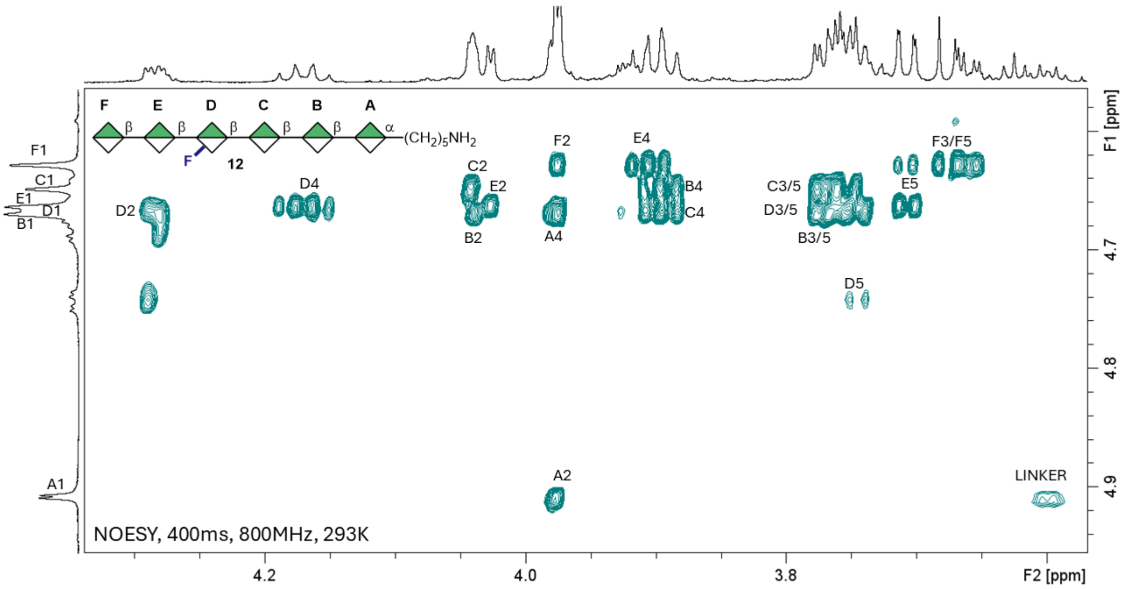

NOESY NMR Spectra of Hexasaccharide 12

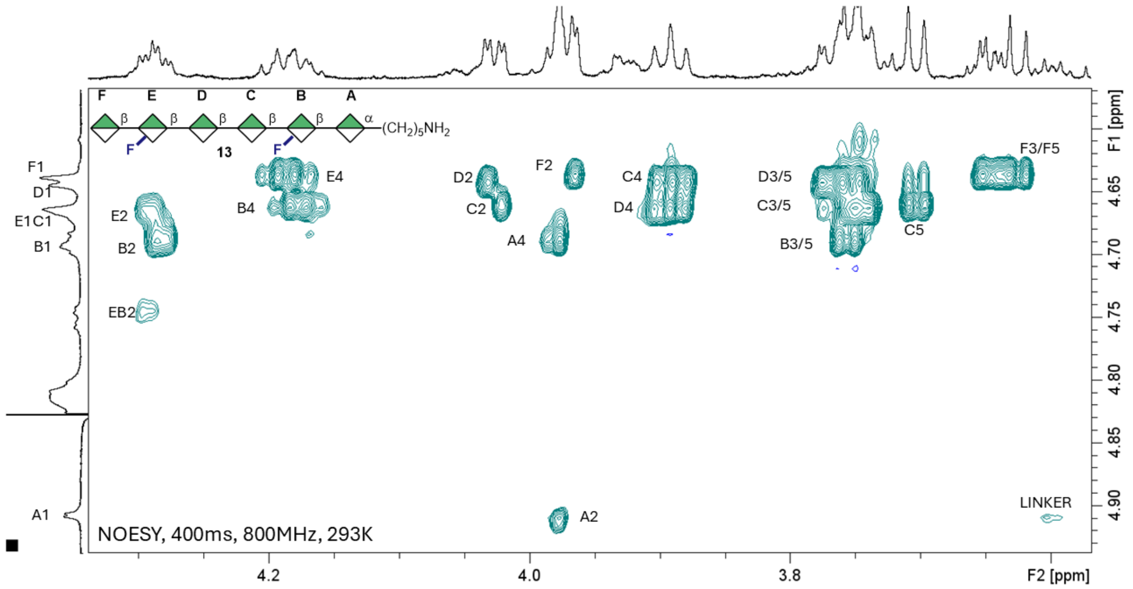

9.3 Computational Methods

Initial molecular models were constructed using the GLYCAM Web tool (http://glycam.org; DOI: [10.1101/2025.05.08.652828]), employing standard carbohydrate templates and β(1→4) glycosidic linkages (except for the α reducing end). Hexameric oligomers of β-D-mannuronic acid were generated using two structurally equivalent approaches. In the first, hexamers of β-D-mannopyranose were built, and the primary alcohol group at C6 (CH_2OH) of each residue was manually converted to a carboxylic acid (COOH) using Macromodel within the Maestro interface (Schrödinger Suite), preserving the stereochemistry at all other positions. In the second approach, hexamers of β-D-glucuronic acid were constructed, which naturally contain a carboxylic acid at C6 and an equatorial hydroxyl group at C2. To match the stereochemistry of mannuronic acid, the C2 hydroxyl group was inverted to an axial orientation in Macromodel, yielding β-D-mannuronic acid residues. Fluorinated analogs were generated by substituting the hydroxyl group at ring position 3 with fluorine in the selected compounds (**10**, **12**, and **13**). All structural modifications were performed in Maestro, followed by geometry optimization using the OPLS_2005 force field and the GB/SA water solvation model. Conformational sampling was carried out using the Monte Carlo Multiple Minimum (MCMM) method to explore the accessible conformational space. Low-energy conformers were selected for further analysis.

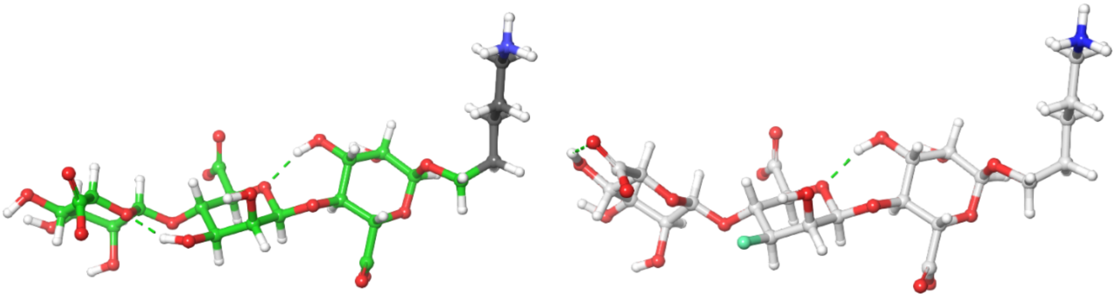

9 (green), **10** (grey)

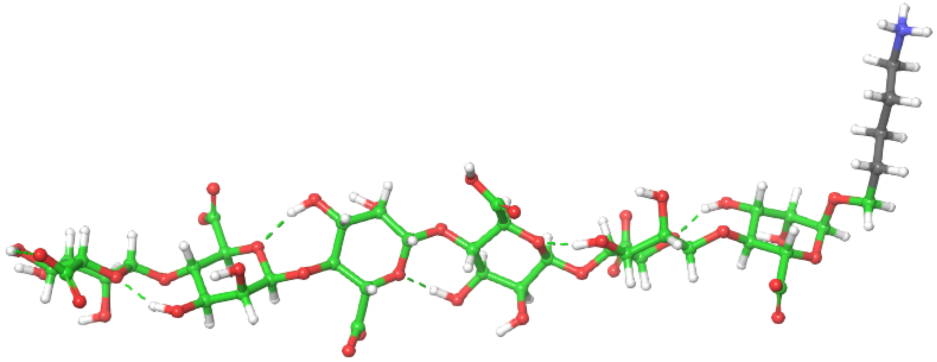

11

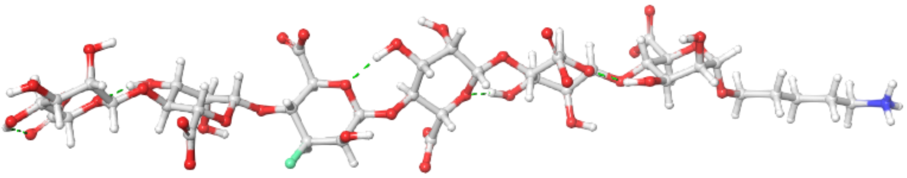

12

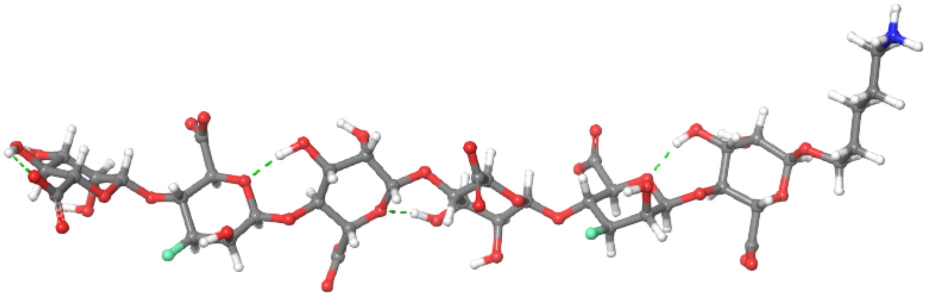

13.

S10. References

1 P. Zihlmann, M. Silbermann, T. Sharpe, X. Jiang, T. Mühlethaler, R. P. Jakob, S. Rabbani, C. P. Sager, P. Frei, L. Pang, T. Maier and B. Ernst, *Chem. Eur. J.*, 2018, **24**, 13049–13057.

2 Y. Lee, X. Hou, R. Chen, J. Feng, X. Liu, M. W. Ruszczycky, J. Gao, B. Wang, J. Zhou and H. Liu, *J. Am. Chem. Soc.*, 2022, **144**, 4478–4486.

3 D. Crich and L. Li, *J. Org. Chem.*, 2007, **72**, 1681–1690.

4 E. S. Cavalli, T. Mies, H. S. Rzepa, A. J. P. White, P. J. Parsons and A. G. M. Barrett, *Org. Lett.*, 2022, **24**, 8931–8935.

5 M. Huang, H. Tran, L. Bohé and D. Crich, in *Carbohydrate Chemistry Proven Synthetic Methods, Volume 2*, ed. G. van der Marel, J. Codee, CRC Press, Boca Raton, 2014, ch. 19, pp. 175-182.

6 V. Gannedi, A. Ali, P. P. Singh and R. A. Vishwakarma, *J. Org. Chem.*, 2020, **85**, 7757–7771.

7 D. Crich and M. Smith, *J. Am. Chem. Soc.*, 2002, **124**, 8867–8869.

8 L. J. van Den Bos, J. Dinkelaar, H. S. Overkleeft and G. A. van der Marel, *J. Am. Chem. Soc.*, 2006, **128**, 13066–13067.

9 M. T. C. Walvoort, H. van den Elst, O. J. Plante, L. Kröck, P. H. Seeberger, H. S. Overkleeft, G. A. van der Marel and J. D. C. Codée, *Angew. Chem. Int. Ed.*, 2012, **51**, 4393–4396.

10 J. Rönnols, M. T. C. Walvoort, G. A. van der Marel, J. D. C. Codée and G. Widmalm, *Org. Biomol. Chem.*, 2013, **11**, 8127–8134.

11 M. Zaiss and P. Bachert, *NMR Biomed.*, 2013, **26**, 507–518.

12 T. Hansen, L. Lebedel, W. A. Remmerswaal, S. van der Vorm, D. P. A. Wander, M. Somers, H. S. Overkleeft, D. V. Filippov, J. Désiré, A. Mingot, Y. Bleriot, G. A. van der Marel, S. Thibaudeau and J. D. C. Codée, *ACS Cent. Sci.*, 2019, **5**, 781–788.

13 F. Neese, *WIREs Comput. Mol. Sci.*, 2012, **2**, 73–78.

14 F. Neese, *WIREs Comput. Mol. Sci.*, 2022, **12**, e1606.

15 F. Neese, F. Wennmohs, U. Becker and C. Riplinger, *J. Chem. Phys.*, 2020, **152**, 224108.

16 G. Santra, N. Sylvetsky and J. M. L. Martin, *J. Phys. Chem. A*, 2019, **123**, 5129–5143.

17 E. Caldeweyher, C. Bannwarth and S. Grimme, *J. Chem. Phys.*, 2017, **147**, 034112.

18 A. D. Becke, *J. Chem. Phys.*, 1993, **98**, 5648–5652.

19 C. Lee, W. Yang and R. G. Parr, *Phys. Rev. B*, 1988, **37**, 785–789.

20 S. H. Vosko, L. Wilk and M. Nusair, *Can. J. Phys.*, 1980, **58**, 1200–1211.

21 P. J. Stephens, F. J. Devlin, C. F. Chabalowski and M. J. Frisch, *J. Phys. Chem.*, 1994, **98**, 11623–11627.

22 S. Grimme, J. Antony, S. Ehrlich and H. Krieg, *J. Chem. Phys.*, 2010, **132**, 154104.

23 S. Grimme, S. Ehrlich and L. Goerigk, *J. Comput. Chem.*, 2011, **32**, 1456–1465.

24 F. Weigend and R. Ahlrichs, *Phys. Chem. Chem. Phys.*, 2005, **7**, 3297-3305.

25 F. Weigend, *Phys. Chem. Chem. Phys*, 2006, **8**, 1057-1065.

26 K. M. Demkiw, W. A. Remmerswaal, T. Hansen, G. A. van der Marel, J. D. C. Codée and K. A. Woerpel, *Angew. Chem. Int. Ed.*, 2022, **61**, e202209401.

27 T. Hansen, H. Elferink, J. M. A. van Hengst, K. J. Houthuijs, W. A. Remmerswaal, A. Kromm, G. Berden, S. van der Vorm, A. M. Rijs, H. S. Overkleeft, D. V. Filippov, F. P. J. T. Rutjes, G. A. van der Marel, J. Martens, J. Oomens, J. D. C. Codée and T. J. Boltje, *Nat. Commun.*, 2020, **11**, 2664.

28 J. Danglad-Flores, E. T. Sletten, E. E. Reuber, K. Bienert, H. Riegler and P. H. Seeberger, *Device*, 2024, **2**, 100499.

29 M. Gude, J. Ryf and P. D. White, *Lett. Pept. Sci.*, 2002, **9**, 203-206.
30 G. Fittolani, T. Tyrikos-Ergas, A. Poveda, Y. Yu, N. Yadav, P. H. Seeberger, J. Jiménez-Barbero and M. Delbianco, *Nat. Chem.*, 2023, **15**, 1461–1469.
31 J. Huang and M. Delbianco, *Org. Biomol. Chem.*, 2024, **22**, 7133–7137.
32 G. Fittolani, D. Vargová, P. H. Seeberger, Y. Ogawa and M. Delbianco, *J. Am. Chem. Soc.*, 2022, **144**, 12469–12475.
33 M. Hurevich, J. Kandasamy, B. M. Ponnappa, M. Collot, D. Kopetzki, D. T. McQuade and P. H. Seeberger, *Org. Lett.*, 2014, **16**, 1794–1797.
